# Terrestrial ecosystem restoration increases biodiversity and reduces its variability, but not to reference levels: A global meta‐analysis

**DOI:** 10.1111/ele.14025

**Published:** 2022-05-12

**Authors:** Joe Atkinson, Lars A. Brudvig, Max Mallen‐Cooper, Shinichi Nakagawa, Angela T. Moles, Stephen P. Bonser

**Affiliations:** ^1^ Evolution & Ecology Research Centre School of Biological, Earth and Environmental Sciences UNSW Sydney Kensington New South Wales Australia; ^2^ Department of Plant Biology and Program in Ecology, Evolution, and Behavior Michigan State University East Lansing Michigan USA

**Keywords:** biodiversity, ecological restoration, lnCVR, meta‐analysis, terrestrial, variability

## Abstract

Ecological restoration projects often have variable and unpredictable outcomes, and these can limit the overall impact on biodiversity. Previous syntheses have investigated restoration effectiveness by comparing average restored conditions to average conditions in unrestored or reference systems. Here, we provide the first quantification of the extent to which restoration affects both the mean and variability of biodiversity outcomes, through a global meta‐analysis of 83 terrestrial restoration studies. We found that, relative to unrestored (degraded) sites, restoration actions increased biodiversity by an average of 20%, while decreasing the variability of biodiversity (quantified by the coefficient of variation) by an average of 14%. As restorations aged, mean biodiversity increased and variability decreased relative to unrestored sites. However, restoration sites remained, on average, 13% below the biodiversity of reference (target) ecosystems, and were characterised by higher (20%) variability. The lower mean and higher variability in biodiversity at restored sites relative to reference sites remained consistent over time, suggesting that sources of variation (e.g. prior land use, restoration practices) have an enduring influence on restoration outcomes. Our results point to the need for new research confronting the causes of variability in restoration outcomes, and close variability and biodiversity gaps between restored and reference conditions.

## Graphical abstract

For the first time, we assess the response of terrestrial biodiversity to restoration measures considering variability alongside mean response. We find that on average, restoration increases mean biodiversity and decreases variability of biodiversity. However, reference sites consistently display both the highest mean and least variable levels of biodiversity.

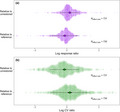

## INTRODUCTION

Efficient and effective ecological restoration is urgently needed to counteract the negative consequences of habitat destruction and degradation for biodiversity and the functioning of natural ecosystems (Suding et al., 2015). Yet, progress towards ambitious global targets (Fagan et al., 2020) is challenged by ineffectiveness at achieving restoration goals (Benayas et al., 2009; Jones et al., 2018) and inconsistent restoration outcomes, hindering the efficient use of limited financial resources (Brudvig et al., 2017). Generally, restoration enhances biodiversity compared with unrestored levels (Crouzeilles et al., 2017; Huang et al., 2019; Jones et al., 2018; Meli et al., 2017). However, the variability of biodiversity among restoration sites within projects is not well understood (Brudvig et al., 2017; Brudvig & Catano, 2021). Unrestored sites typically show substantial variability in biodiversity, owing to the variety and severity of degrading processes that can act on ecosystems, including altered disturbance regimes, invasive species, and land‐use conversion (Crouzeilles et al., 2016; Meli et al., 2017). Since the goal of restoration is generally to guide various degraded conditions toward reference conditions, variation in biodiversity outcomes among restoration projects (hereafter ‘among‐restoration variation’) is likely to be highest where degradation was also highly variable. Alternatively, if the dominant forms of disturbance pre‐restoration are homogeneous, then restoration is likely to produce more predictable and less variable biodiversity outcomes. In this study, we consider how restoration influences both variability and overall levels of biodiversity following ecological restoration, with respect to both degraded and reference conditions.

Understanding how biodiversity outcomes change over time following restoration is crucial for accurately setting restoration targets and optimising management decisions. On short timescales (<5 years), restoration projects often take divergent trajectories due to a strong influence of local environmental gradients, successional dynamics and stochastic variation, even when on average moving towards reference levels (Matthews & Spyreas, 2010). Over longer time scales, the variation in biodiversity among restoration replicates can decrease due to a predominating influence of a common set of factors (e.g. climate, disturbance; Matthews & Spyreas, 2010), whereas overall biodiversity accumulates (Holl, 2020). Here, we evaluate the extent to which age of restoration moderates overall levels and the variability of biodiversity.

The magnitude of degradation at an unrestored site usually delimits the goal of any given restoration project, and therefore, the outcomes of restoration (Brudvig et al., 2017; Crouzeilles et al., 2017; Groves et al., 2020; Prach et al., 2020). Land‐use history is a major driver of biodiversity outcomes, for example, a history of low‐intensity disturbance (e.g. selective logging) can lead to more desirable biodiversity outcomes in restored forests compared with high‐intensity disturbance regimes (Crouzeilles et al., 2016). Understanding how among‐restoration variation in biodiversity differs across historical land uses will enable more accurate predictions of variability in biodiversity outcomes. Because sites with the most intensive historical land use generally exhibit the highest alteration from baseline conditions, demanding the most complex restoration interventions (Hobbs & Norton, 1996; Prach et al., 2020), we expect that they will have the highest levels of among‐restoration variability in biodiversity. Additionally, many of the restoration methods applied in less degraded starting conditions (e.g. burning, thinning, mowing) could select for specific suites of species adapted to these treatments (Pausas & Bradstock, 2007; Rainsford et al., 2021; Shryock et al., 2014), as well as being broadly more homogenous treatments compared with those employed in complex interventions, resulting in less variable outcomes. These particular low‐degradation methods may also provide more suitable environmental conditions for species found in reference ecosystems and, therefore, result in more consistently successful restoration. However, if restoration failure takes time – that is, it takes a long time for a failed project to become apparent, or sites decline in target metrics of biodiversity over time – then variability may instead increase at longer timescales. Lastly, it may be that intense land‐use histories may cause very homogenous disturbance to a given area, reducing the variability of outcomes in these environments.

The scale of a restoration site can strongly affect its success in meeting desired outcomes (Ager et al., 2017), but little is known about the moderating influence of scale on variability in these outcomes (Brudvig et al., 2017). There are complex logistical, political and financial intricacies associated with scaling up ecological restoration while maintaining biodiversity benefits (Murcia et al., 2016), yet large‐scale goals are often perceived as the ideal (Ehrenfeld, 2000; Sexton & Emery, 2020). It may be that larger restoration sites are likely to display greater among‐restoration variation in biodiversity due to increased variety in abiotic factors (evapotranspiration, topography, soil), biotic inputs (species pool, soil seed bank) and historical contingencies acting on restorations conducted at landscape scales (Buffam et al., 2007; Cohen et al., 2016). Conversely, historical contingencies such as stochastic dispersal or biotic inputs may have a stronger influence on community assembly at relatively fine scales, leading to highly variable outcomes at smaller sites (Benincà et al., 2008; Stark et al., 2008). In terms of mean biodiversity levels, we expect that the same challenges (e.g. landscape heterogeneity, logistical and financial barriers) could limit overall biodiversity increases following restoration.

In summary, the aim of our study is to use a global meta‐analysis of terrestrial ecological restoration studies to ask: (1) What is the effect of ecological restoration on both variability in biodiversity and average biodiversity with respect to both unrestored and reference conditions?; (2) Does biodiversity increase with time since restoration, and do sites become more or less variable?; (3) Does biodiversity decrease with spatial extent of restoration, and do sites become more or less variable? and (4) How does land‐use history moderate the effect of restoration on overall levels and variability of biodiversity?

## METHODS

Two databases – Web of Science Core Collection and Scopus – were searched for all studies published up until March 25, 2020, using the following search string: (((restoration or restored) and (eco*) and (monitor* or success* or evaluat* or assess*)) and (biodiversity) and (taxonomic richness or taxonomic diversity or species diversity or species richness or functional diversity or functional richness or phylogenetic diversity)). To narrow the search to projects with goals of enhancing biodiversity and returning ecosystem integrity (following Society for Ecological Restoration (SER) definitions, https://www.ser‐rrc.org), we did not explicitly include terms such as reforestation, reclamation or rehabilitation in our search (Wortley et al., 2013), although we did not exclude studies using these terms during screening of results. This yielded 1796 results from Web of Science and 697 results from Scopus. An additional three studies were identified in literature databases of previous meta‐analyses. The total number of records was reduced to 2277 after 219 duplicates were excluded. After the removal of irrelevant studies, 584 studies remained for full‐text screening. Following the full‐text screening, 83 studies had data extracted for analysis (see Appendix S1 for details).

The literature search protocol was informed by the PRISMA (Preferred Reporting Items for Systematic Reviews and Meta‐Analyses) statement (Liberati et al., 2009; Moher et al., 2009), and we have provided a PRISMA‐EcoEvo checklist (O’Dea et al., 2021; archived at https://osf.io/4aucp/).

### Screening and extraction

From each study, we extracted mean biodiversity (as well as the metric of biodiversity measurement), standard deviation, coefficient of variation, standard error, past land status, sample size *(N*), ‘treatment’ (restored, unrestored, reference), age of restoration (years), approximate scale of restoration project (ha) and restoration method. For studies where the values were condensed into categories (e.g. ‘young’ and ‘old’ restoration sites), or where the history of restoration was not well known and given only as a range, we used median values. The categories used for past land use were forestry, mining, agriculture, urban, semi‐natural. ‘Semi‐natural’ was adopted as a category for studies of sites that were not necessarily heavily degraded in the classic sense, but where the cessation of a disturbance produced an undesired state transition (e.g. woody encroachment where the restoration treatment to return desired conditions was thinning and burning). We scored restoration method across three categories: 1 – natural restoration (cessation of the degrading process to allow natural recovery), 2 – assisted restoration (active remediation of substrate, reintroduction of species, invasive species management) and 3 – reconstructive restoration (a combination of both strategies with reintroduction of a major proportion of desired biota) (Atkinson & Bonser, 2020; Gann et al., 2019). Studies that only measured structural changes such as abundance or cover, or physical and chemical attributes of soil, were not included. Additionally, we noted the focal organisms of the study, the category of restoration method, the approximate location, and the size of the quadrats where applicable. For all available diversity variables in a study, we extracted the mean and standard deviation (SD), often calculated from raw data or other measures of spread. Where values were not available in text, we extracted them from figures using WebPlotDigitizer (Rohatgi, 2020) and the RStudio package metaDigitise (Pick et al., 2019).

Cross‐study biodiversity syntheses are prone to error and bias by comparing across multiple spatial scales or units of replication (Spake et al., 2020). Biodiversity restoration studies generally aim to understand the effect of restoration methods across multiple independent ‘efforts’. We considered each restoration effort as the unit of replication (*N*) in our calculations of standard deviation. For example, the variability of biodiversity among restorations is the variance reported within a single restoration treatment replicated across several restoration efforts in a single study (see *Scale dependency* section of Appendix S1 for more details).

### Effect sizes

Effect size calculations were completed using the *escalc* function in the ‘metafor’ R package (Viechtbauer, 2020). Due to the diversity of restoration methods used worldwide, effect sizes are calculated from both experimental and observational comparisons.

To quantify the effect of restoration on the relative variability of biodiversity we used the natural logarithm of the ratio between the coefficients of variation (lnCVR) (Nakagawa et al., 2015; Senior et al., 2020). Sánchez‐Tójar et al. (2020) suggest that lnCVR is preferable over the variability ratio (lnVR; Nakagawa et al., 2015) when there is a strong mean‐variance relationship, as it can account for the simultaneous difference between group mean and variance (Cohen & Xu, 2015; Nakagawa & Schielzeth, 2012). That is, in terms of our research questions, it would be unsurprising that restoration increases variability given it is also known to generally increase mean biodiversity (Huang et al., 2019; Jones et al., 2018; Meli et al., 2017); Figure S1), so the use of a relative measure of variability such as lnCVR is important. We assess heterogeneity in our models using *I* ^2^ (Higgins et al., 2003) and present these alongside meta‐analysis plots in Appendix S1 (Figure S3).

For mean differences, we used the log response ratio (lnRR; Hedges et al., 1999). Advantages of lnRR over the standardised mean difference (Cohen's *d*) include its ease of interpretability as a percentage response and its resilience to influence by heteroscedasticity (Sánchez‐Tójar et al., 2020). We calculated lnCVR and lnRR for two sets of models, the first comparing unrestored levels with restorations and the second comparing restorations with reference levels.

The data set comparing unrestored sites with restored sites consisted of 734 effect sizes from 59 studies, and for the comparison of restored sites and reference sites, there were 739 effect sizes from 66 studies. Thirty‐nine studies presented data for unrestored, restored and reference conditions.

### Meta‐analyses and meta‐regression

For lnCVR and lnRR, we specified restored sites as the numerator and the unrestored group as the denominator, so that positive values correspond to increased biodiversity variability at restored sites, and vice versa for negative values.

To account for the effect of the multi‐level structure in our data (e.g. repeated measurements within a study) on heterogeneity, we ran multilevel meta‐analytic models for both lnCVR and lnRR to test the variability and mean effects of restoration on biodiversity respectively (Nakagawa & Santos, 2012). A meta‐analytic (intercept) model was used to calculate the overall effect of restoration in the absence of moderators. We then used one meta‐regression model to investigate the influence of moderators that were available for all studies (age of restoration, past land use, measure of biodiversity) and a second model with a reduced sample size to determine the moderating effect of scale. The meta‐analytic and meta‐regression models included two random effects: study and plot, which accounted for repeated measures over time and across biodiversity metrics. To account for correlations between diversity metrics measured in the same plots, we constructed a variance‐covariance matrix that was used as the variance parameter of all mixed models (Noble et al., 2017). Marginal *R* ^2^ and conditional *R* ^2^ values (Nakagawa & Schielzeth, 2013) were calculated using the *r2_ml* from the *orchaRd* package (Nakagawa et al., 2020). We also tested our intercept models for evidence of publication bias, time‐lag bias and scale dependency (Appendix S1; Figures S8–S11; Tables S7–S10). Lastly, we ran meta‐analysis of overall variability and mean responses as well as the moderating effects of restoration site age separately for the organism categories (plants, invertebrates, vertebrates, microbes, fungi, and amoebae), to check the sensitivity of our results across broad taxonomic groups. Pairwise comparisons of groups (taxon) was conducted using the *multcomp* package (Hothorn et al., 2008) for any models for which there was evidence of differences in taxon.

All analysis was conducted using R version 4.1.2 (RStudio Team, 2022). All data and code for running the analysis and data visualisation are available from OSF: https://osf.io/4aucp/.

## RESULTS

**FIGURE 1:**
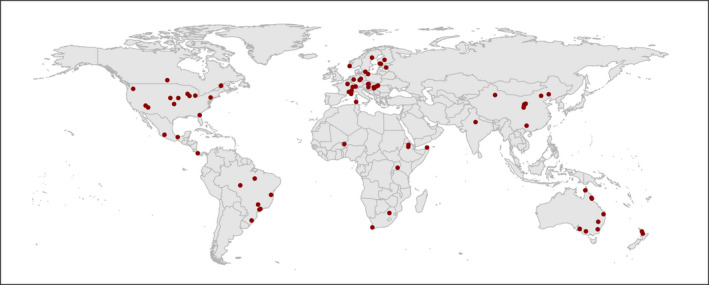
Global distribution of studies included in the meta‐analyses (*N* = 83)

The majority of biodiversity data (for all data sets) were derived from plants (*N* = 608, 61.4%) and invertebrates (*N* = 280, 28.3%), with the remaining values deriving from vertebrates (*N* = 42, 4.2%), fungi (*N* = 41, 4.1%), soil microbes (*N* = 6, 0.6%) and testate amoebae (*N* = 12, 1.2%). Measures of biodiversity included taxonomic (*N* = 489, 49.9%), functional (*N* = 243, 24.5%), phylogenetic (*N* = 9, 0.9%) and other biodiversity indices (*N* = 248, 25%). Our data set includes studies conducted in Europe, North America, South America, Asia, Oceania, Australasia and Africa (Figure 1). Restoration sites ranged in size from 0.001 to 2300 ha, with an average size of 76 ha, and in age from 0.1 to 54.5 years old (median = 7, mean = 10). Restorations were somewhat evenly spread between woody (583, 59%) and non‐woody (406, 41%) ecosystems. Twenty‐two studies assessed natural (or ‘passive’) restoration, with 64 studies assessing assisted restoration methods and six studies assessing ecosystem reconstruction (note some studies included sites of multiple categories).

**FIGURE 2:**
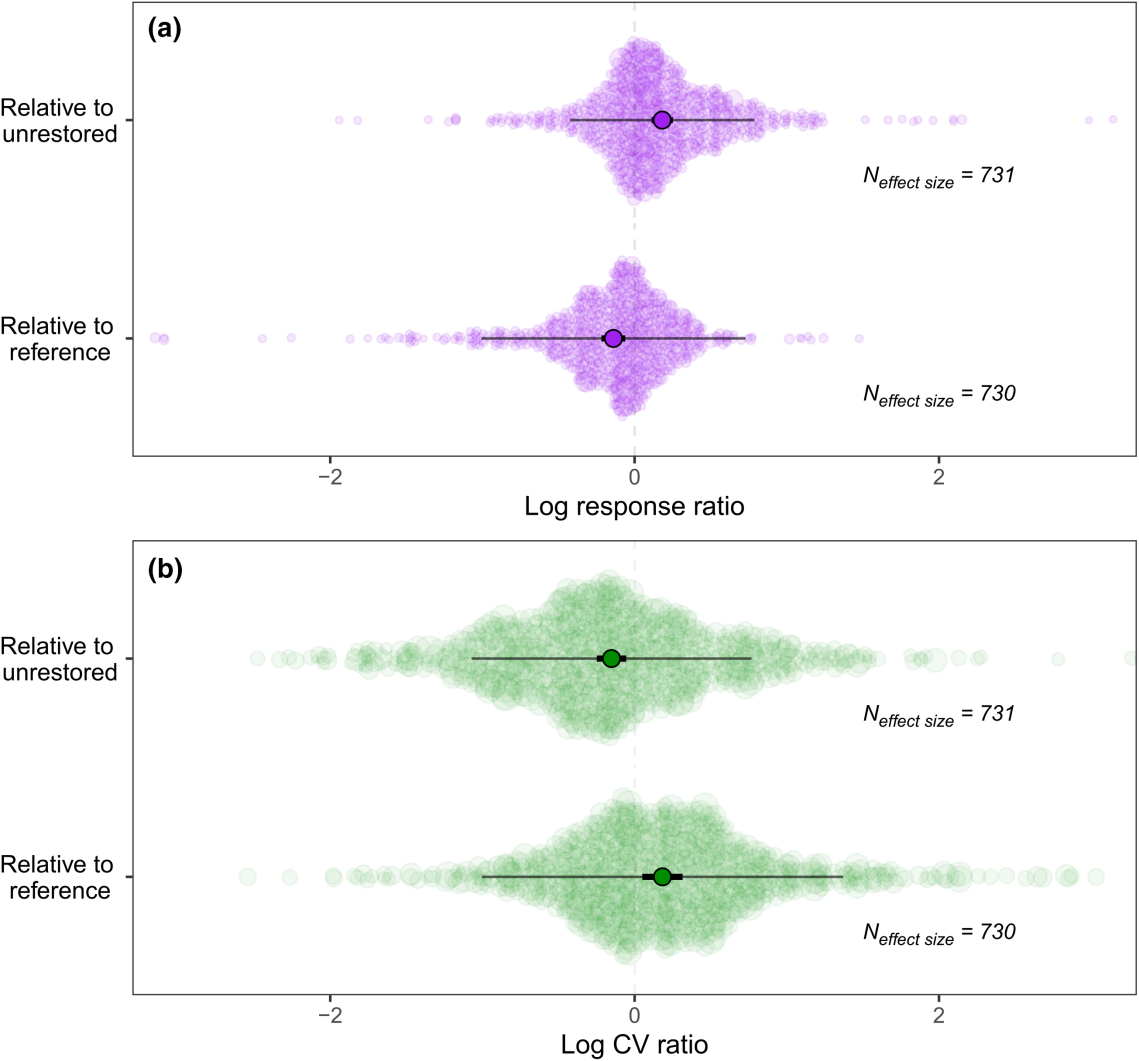
Meta‐analytic model results comparing the (a) mean (LnRR) and (b) variability (LnCVR) of biodiversity in restored sites with either unrestored or reference sites (central points represent model estimated means, thick bars represent 95% confidence intervals, and thin bars 95% prediction intervals). Each background point is an effect size, and its size is scaled by the precision of that estimate (1/SE). Note that a small number of outliers have not been shown here (those <‐3 or >3) but are visible on Figure 5

Restored sites had 20% higher mean biodiversity than did unrestored sites (LnRR = 0.18, 95% confidence interval (hereafter CI) = 0.11–0.25, *p* < 0.001; Figure 2). Variation in biodiversity was 14% lower among restorations than among unrestored sites (LnCVR = −0.16, *CI* = −0.25–−0.06, *p* = 0.002; Figure 2b). However, restored site biodiversity was on average 13% lower (LnRR = −0.14, CI = −0.22–−0.06, *p* < 0.001), and variation in biodiversity 20% higher, than reference sites (LnCVR = 0.18, CI = 0.05–0.31, *p* = 0.007; Figure 2b). Subgroup analysis at the taxon level showed no significant variation between taxon responses in any model (Table S15).

**FIGURE 3:**
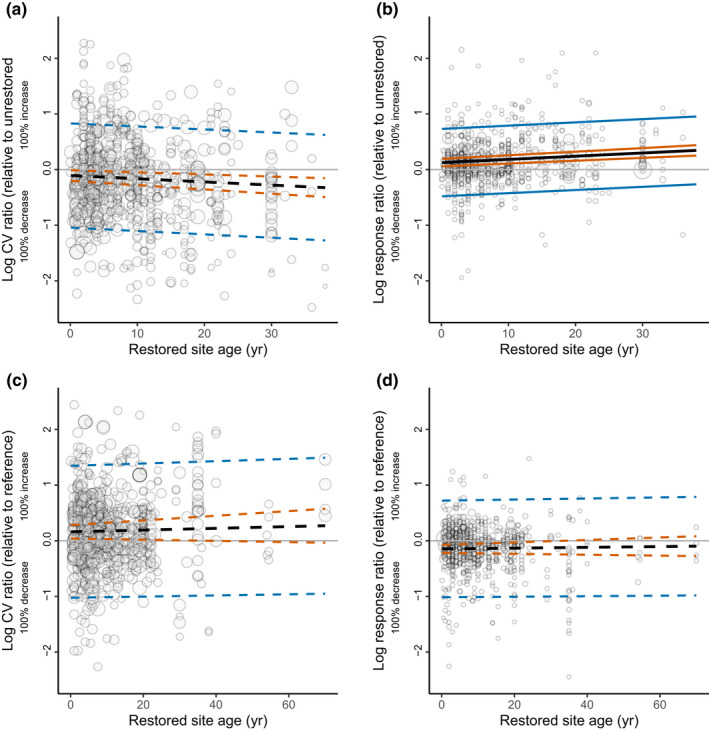
Meta‐regression model results showing the moderating effects of restoration age on (a, c) variability of biodiversity (lnCVR) and (b, d) mean (lnRR) biodiversity relative to both unrestored (a, b) and reference (c, d) sites (red dashed line represents 95% confidence intervals, and blue dashed line 95% credibility intervals). Each background point is an effect size, and its size is scaled by the precision of that estimate (1/SE). Dashed/solid lines represent statistically non‐significant/significant results

Age of restoration sites had varied effects on the relative mean and variability of biodiversity. The effect of restoration on mean biodiversity relative to unrestored ecosystems was more pronounced at older restored sites. Biodiversity at restored sites showed a mean increase of approximately 0.6% per year (LnRR = 0.006, CI = 0.003–0.009, *p* < 0.001, *N* = 728; Figure 3a), relative to unrestored sites, and no significant change in variability through time (LnCVR = −0.005, CI = −0.013–0.002, *p* = 0.15, *N* = 728; Figure 3b). Age of restoration also had no significant effect on the mean (estimate = 0.001, CI = −0.003–0.006, *p* = 0.58, *N* = 739; Figure 3a) or variability (estimate = 0.001, CI = −0.006–0.009, *p* = 0.71, *N* = 739; Figure 3b) of biodiversity relative to reference sites. When incorporating a taxon‐level interaction with age, there was no significant heterogeneity between groups except in mean biodiversity comparisons between restored and unrestored sites (Table S16). However, pairwise comparisons between taxon in this model showed no significant differences between each taxa (Table S17).

**FIGURE 4:**
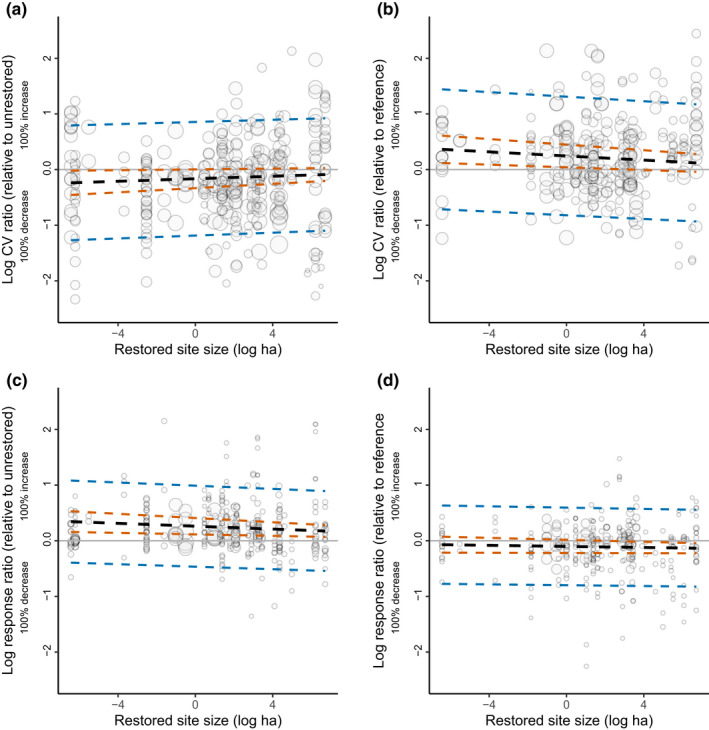
Meta‐regression model results showing the moderating effects of site size against (a, b) variability of biodiversity (lnCVR) and (c, d) mean (lnRR) biodiversity relative to both unrestored (a, c) and reference (b, d) sites (red dashed line represents 95% confidence intervals, and blue dashed line 95% credibility intervals). Each background point is an effect size, and its size is scaled by the precision of that estimate (1/SE). Dashed/solid lines represent statistically non‐significant/significant results

There was no significant effect of restoration scale on mean or variability of biodiversity for either set of models (Figure 4a–b; *N* = 347 for restored/unrestored models and *N* = 297 for restored/reference models).

**FIGURE 5:**
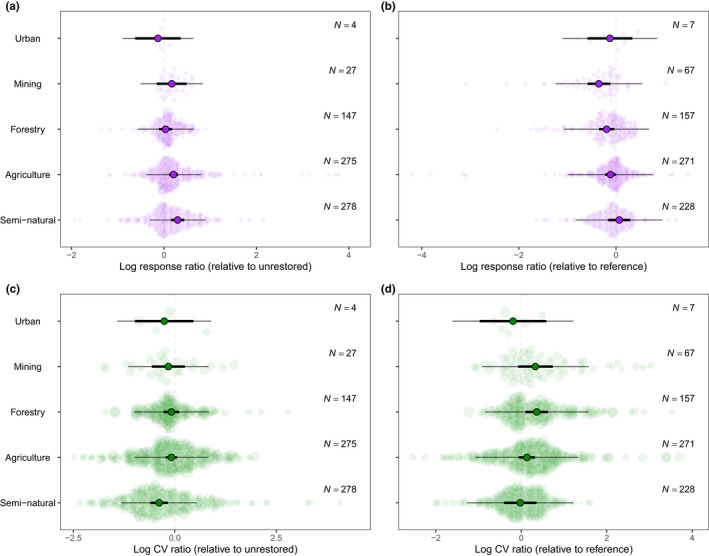
Meta‐regression model results showing the moderating effects of past land status mean (a, b; lnRR) and variability (c, d; lnCVR) of biodiversity relative to both unrestored and reference sites (points represent model estimated slopes and distributional margins represent 95% confidence intervals)

Restoration of semi‐natural (e.g. sites subjected to thinning, burning, mowing) and agricultural land produced higher mean biodiversity increases (compared with degraded unrestored systems) than other past land‐uses (Figure 5a). Restored semi‐natural sites showed the least variation in biodiversity outcomes of any past land‐use type, relative to unrestored sites (Figure 5b). Restoration of sites that had been subjected to agriculture, forestry, urban use, invasive species removal or mining did not significantly affect biodiversity variability (Figure 5a–b).

## DISCUSSION

We found strong empirical evidence that restoration generally increased mean biodiversity and reduced the variability of biodiversity compared with unrestored levels. However, mean biodiversity was lower and variability higher at restored sites than at reference sites, indicating that, on average, there are still biodiversity shortfalls and uncertainties in reported restorations. Our models suggest that restoration scale is not an important moderator of biodiversity outcomes. The effects of restoration, relative to unrestored sites, became stronger with increasing age, yet there were no significant effects of age when comparing with reference sites. Only in previously semi‐natural systems was there a significant effect of restoration on biodiversity variability, with reduced variability relative to the original state. The results of our main models and age effects were consistent across broad taxonomic groups. Together, these results advance our understanding of the effects and drivers of biodiversity and its variability to restoration actions.

We found that restorations generally occupied a middling condition between degraded and reference sites in terms of both mean and variability of biodiversity. The mean results reinforce the findings of past work that restoration usually leads to a deviation from the degraded state but rarely a full recovery to the reference state (Benayas et al., 2009; Jones et al., 2018; Meli et al., 2017). It may also be that the typical time scales of restoration studies do not allow sufficient time to enable full recovery (Ruiz‐Jaen & Aide, 2005; Tischew et al., 2010). Historical land‐use type and intensity are likely to play major roles in determining the response of the environment to restoration (Bullock et al., 2011; Prach et al., 2020) since land use can have lasting effects on soil seed banks (Bekker et al., 1997; Bossuyt & Hermy, 2001) and the status of soil nutrients and compaction (Standish et al., 2007). Our models indicate that agricultural and semi‐natural systems have the greatest capacity to shift away from a degraded state (Figure 5a), which might relate to their relatively low alteration from original or reference conditions compared with mining and forestry.

The reduction in the variability of biodiversity relative to degraded sites, where disturbances are not likely to be entirely homogeneous, implies that restoration actions are initiating the convergence of biodiversity toward a new state (although not one comparable with reference ecosystems). One explanation for this finding is that restoration actions (e.g. thinning, mowing, and burning of vegetation) may be favouring a particular suite of species that respond positively to these treatments (Kahmen & Poschlod, 2008; Spasojevic et al., 2010; Stammel et al., 2003). Another possibility is that convergence could be explained by the direct planting or translocating of similar species during restoration (Gann et al., 2019; Holl, 2020). However, we also found that biodiversity variability in restorations remained much higher than reference ecosystems, which is likely to be driven by similar divergence processes as those that act on mean biodiversity, such as successional dynamics and species invasions. This finding might also be explained, in some cases, by the selection of reference sites that fail to represent the complete diversity of environmental conditions experienced by the target ecological community or restored landscape. Similarly, a bias in favour of selecting reference sites of particularly high quality could lead to variability goals that are unattainable in practice, and in some cases it may be appropriate to select more realistic reference targets given the extent of degradation (Hobbs, 2007; Miller & Hobbs, 2007).

We showed that the mean biodiversity of restorations significantly increased over time. This suggests that restoration efforts can develop ecosystem conditions that are suitable for the accrual of additional species. However, we did not detect a moderating influence of age on biodiversity differences among restored and reference sites. This may reflect the relatively short timeframes of study for the majority of projects or may be evidence of restoration methods that produce incomplete (or divergent) ecosystem recovery (Matzek et al., 2016; Parkhurst et al., 2021; Salaria et al., 2019). Alternatively, the absence of biodiversity accrual at restoration sites relative to reference levels may be explained by restorations following nonlinear trajectories (Jones et al., 2018). Heterogeneity among the effects of restoration was high in all models (Table S3), indicating that despite significant overall effect sizes, there are large inconsistencies in the effect of restoration on both mean and variability of biodiversity. As monitored restorations age, it may be possible to better disentangle this relationship with site age and community assembly, and the development of novel restoration strategies to close lingering biodiversity deficits between restorations and reference sites. Regardless of the causes, this biodiversity gap underscores the inappropriateness of our current restoration practices to substitute conservation (Jones et al., 2018). Importantly, the effects of age were consistent across broad taxonomic categories.

Our analysis found no evidence that biodiversity variability changed with time since restoration, that is, restored sites failed to approach the low variability values typical of reference sites. Therefore, a potentially critical opportunity in current restoration projects to decrease variability in biodiversity is at the onset of restoration, perhaps by standardising initial restoration practises (Matthews & Spyreas, 2010). Doing so will be important for maximising the reliability of future restorations. Support for this notion has been found in some restoration systems in mean biodiversity response to restoration activities, for example, the initial floristics model where initial inputs predict established biodiversity at a later stage (Egler, 1954; Koch, 2007).

The most marked reduction of variability was at sites that were categorised as ‘semi‐natural’ suggesting that such methods may predictably select for certain groups of species adapted to those management interventions, resulting in less variable outcomes (Matthews, 2015; Matthews & Spyreas, 2010; Newbold et al., 2020). We used categories of land use because we expected the treatments used in some categories to be simpler and more homogenous (e.g. potentially producing less variable outcomes), as well as being associated with lower degradation or modification of natural abiotic and biotic conditions (e.g. soil conditions, seedbanks). However, we also thought that more intense land‐uses could produce more homogenous disturbance, resulting in less variable outcomes compared with less‐disturbed sites. Our results suggest the former explanation is the more generalisable. Further, these types of sites are possibly better able to support the suites of species desired in restoration because soil, seedbanks, and other ecosystem properties are less likely to be fundamentally altered (De Barros et al., 2020; Li et al., 2017; Prach et al., 2020). Although variability among restored sites was generally higher than among reference sites, such an effect was not present at semi‐natural sites. This result suggests that the restoration of semi‐natural landscapes is producing less‐variable results more consistent with reference levels. Research specifically confronting variability in more uncertain sites, such as those previously used for forestry, will result in more reliable and predictable restoration outcomes. Furthermore, while past land‐use categories enabled us to test the broader findings across a range of common restoration settings, we are not able to directly compare the effect of increased degradation. Future synthesis of the effects of degradation level will provide valuable information for restoration practitioners.

In our study, we have advanced novel generalities on the variability of biodiversity among restoration efforts. While restoration is generally successful in promoting targeted biodiversity and does not exacerbate variability in diversity, restoration efforts do not return these key diversity measures to those observed in reference communities. Our results show that age, size and broad categories of land use are not strong predictors of variability. Further resolving these drivers at finer taxonomic and geographic scales will provide an opportunity to increase restoration predictability and better manage limited conservation resources (Brudvig & Catano, 2021). Although current data are limited, we hope that future synthesis capitalising on the many efforts currently underway to develop comprehensive databases of restoration data (Ladouceur et al., 2022). More detailed restoration data will be able to extend the present work by quantifying the difference in responses of native versus exotic biodiversity, the effectiveness of various levels of restoration intervention (across and among taxa), and the effects of gradients of disturbance and the long‐term resilience of restoration activities.

Although a degree of the predictive capacity of restoration ecology inevitably operates at a site‐specific level (Reid et al., 2018), some generalities can and have been made about the variability and mean biodiversity response to restoration. Finally, restorations that are unpredictable may negatively influence the perceptions of the value of restoration by policy‐makers, volunteer groups, and other key funding and support groups (Zahawi et al., 2014). As global commitments to restoration soar (Fagan et al., 2020), garnering ongoing public support and good faith engagement from policy‐makers will be vital.

## CONFLICT OF INTEREST

The authors have no conflicts of interest to declare.

## AUTHOR CONTRIBUTION

JA led study conceptualisation with input from all authors. JA conducted data collection and analysis. All authors critically contributed to drafting and subsequent revisions of the manuscript.

### PEER REVIEW

The peer review history for this article is available at https://publons.com/publon/10.1111/ele.14025.

### OPEN RESEARCH BADGES

This article has earned Open Data and Open Materials Design badges. Data and materials design and analysis plan are available at: https://doi.org/10.17605/OSF.IO/4AUCP.

## Supporting information

Appendix S1

## Data Availability

All data and code for running the analysis and data visualisation are available from the Open Science Framework at https://doi.org/10.17605/OSF.IO/4AUCP
