# Supplementary material for: Terrestrial ecosystem restoration increases biodiversity and reduces its variability, but not to reference levels: A global meta‐analysis
Source: Ecol Lett. 2022 May 12;25(7):1725–37. doi: 10.1111/ele.14025 (PMC9320827; doi:10.1111/ele.14025)
Supplement: Supplementary file 1 — Appendix S1 [file ELE-25-1725-s001.html]

Terrestrial ecosystem restoration increases biodiversity and reduces its variability, but not to reference levels: a global meta-analysis


Code 

- Show All Code
- Hide All Code
- Download Rmd

# Terrestrial ecosystem restoration increases biodiversity and reduces its variability, but not to reference levels: a global meta-analysis

### Electronic Supplementary Material

#### Joe Atkinson, Lars Brudvig, Max Mallen-Cooper, Shinichi Nakagawa, Angela T. Moles, Stephen P. Bonser

#### 21 March 2022

## Setups

### Loading packages and custom functions

To run the following script, some packages may need to be installed
from `Github`.

```
# install.packages("devtools")
# install.packages("tidyverse")
# install.packages("metafor")
# install.packages("patchwork")
# install.packages("R.rsp")
# 
# devtools::install_github("itchyshin/orchard_plot", subdir = "orchaRd", force = TRUE, build_vignettes = TRUE)

library(metafor)
library(broom)
library(readxl)
library(tidyverse)
library(rmarkdown)
library(kableExtra)
library(orchaRd)
library(patchwork)
library(sjPlot)
library(gridExtra)
library(jtools)
library(ggtext)
library(purrr)
```

### Custom functions

```
#' @title Covariance and correlation matrix function basing on shared level ID
#' @description Function for generating simple covariance and correlation matrices 
#' @param data Dataframe object containing effect sizes, their variance, unique IDs and clustering variable
#' @param V Name of the variable (as a string – e.g, "V1") containing effect size variances variances
#' @param cluster Name of the variable (as a string – e.g, "V1") indicating which effects belong to the same cluster. Same value of 'cluster' are assumed to be nonindependent (correlated).
#' @param obs Name of the variable (as a string – e.g, "V1") containing individual IDs for each value in the V (Vector of variances). If this parameter is missing, label will be labelled with consecutive integers starting from 1.
#' @param rho Known or assumed correlation value among effect sizes sharing same 'cluster' value. Default value is 0.5.
#' @param type Optional logical parameter indicating whether a full variance-covariance matrix (default or "vcv") is needed or a correlation matrix ("cor") for the non-independent blocks of variance values.
#' @export

make_VCV_matrix <- function(data, V, cluster, obs, type=c("vcv", "cor"), rho=0.5){
  type <- match.arg(type)
  if (missing(data)) {
    stop("Must specify dataframe via 'data' argument.")
  }
  if (missing(V)) {
    stop("Must specify name of the variance variable via 'V' argument.")
  }
  if (missing(cluster)) {
    stop("Must specify name of the clustering variable via 'cluster' argument.")
  }
  if (missing(obs)) {
    obs <- 1:length(V)   
  }
  if (missing(type)) {
    type <- "vcv" 
  }
  
  new_matrix <- matrix(0,nrow = dim(data)[1],ncol = dim(data)[1]) #make empty matrix of the same size as data length
  rownames(new_matrix) <- data[ ,obs]
  colnames(new_matrix) <- data[ ,obs]
  # find start and end coordinates for the subsets
  shared_coord <- which(data[ ,cluster] %in% data[duplicated(data[ ,cluster]), cluster]==TRUE)
  # matrix of combinations of coordinates for each experiment with shared control
  combinations <- do.call("rbind", tapply(shared_coord, data[shared_coord,cluster], function(x) t(utils::combn(x,2))))
  
  if(type == "vcv"){
    # calculate covariance values between  values at the positions in shared_list and place them on the matrix
    for (i in 1:dim(combinations)[1]){
      p1 <- combinations[i,1]
      p2 <- combinations[i,2]
      p1_p2_cov <- rho * sqrt(data[p1,V]) * sqrt(data[p2,V])
      new_matrix[p1,p2] <- p1_p2_cov
      new_matrix[p2,p1] <- p1_p2_cov
    }
    diag(new_matrix) <- data[ ,V]   #add the diagonal
  }
  
  if(type == "cor"){
    # calculate covariance values between  values at the positions in shared_list and place them on the matrix
    for (i in 1:dim(combinations)[1]){
      p1 <- combinations[i,1]
      p2 <- combinations[i,2]
      p1_p2_cov <- rho
      new_matrix[p1,p2] <- p1_p2_cov
      new_matrix[p2,p1] <- p1_p2_cov
    }
    diag(new_matrix) <- 1   #add the diagonal of 1
  }
  
  return(new_matrix)
}


#' @title model_table: univariate rma.mv models
#' @description Function to get estimates, CIs (confidence intervals) from rma objects (metafor) and output into neat table - this one designed for three models at a time 
#' @param m1: first rma.mv object 
#' @param m2: second rma.mv object
#' @param m3: third rma.mv object
#' @param names: list of names for the "Effect size" column, must be length three and correspond to m1,m2,m3 effect sizes

model_table<-function(m1, m2, m3, names){
  #r2 <- r2_ml(model1, model2, model3)
  
  # creating a table
  
  tibble(`Effect size` = names,
         `Estimate` = c(m1$b, m2$b, m3$b), 
         `Lower CI [0.025]` = c(m1$ci.lb, m2$ci.lb, m3$ci.lb), 
         `Upper CI  [0.975]` = c(m1$ci.ub, m2$ci.ub, m3$ci.ub), 
         `P value` = c(m1$pval, m2$pval, m3$pval)) %>% kable("html", digits = 3) %>% 
    kable_styling(position = "left") 
  
  
}

model_table<-function(m1, m2, m3, names){
  #r2 <- r2_ml(model1, model2, model3)
  
  # creating a table
  
  tibble(`Effect size` = names,
         `Estimate` = c(m1$b, m2$b, m3$b), 
         `Lower CI [0.025]` = c(m1$ci.lb, m2$ci.lb, m3$ci.lb), 
         `Upper CI  [0.975]` = c(m1$ci.ub, m2$ci.ub, m3$ci.ub), 
         `P value` = c(m1$pval, m2$pval, m3$pval)) %>% kable("html", digits = 3) %>% 
    kable_styling(position = "left") 
  
  
}

get_pred1 <- function(model, mod = " ") {
  name <- name <- firstup(as.character(stringr::str_replace(row.names(model$beta), 
                                                            mod, "")))
  len <- length(name)
  
  if (len != 1) {
    newdata <- matrix(NA, ncol = len, nrow = len)
    for (i in 1:len) {
      pos <- which(model$X[, i] == 1)[[1]]
      newdata[, i] <- model$X[pos, ]
    }
    pred <- metafor::predict.rma(model, newmods = newdata)
  } else {
    pred <- metafor::predict.rma(model)
  }
  estimate <- pred$pred
  lowerCL <- pred$ci.lb
  upperCL <- pred$ci.ub
  lowerPR <- pred$cr.lb
  upperPR <- pred$cr.ub
  
  table <- tibble(name = factor(name, levels = name, labels = name), estimate = estimate, 
                  lowerCL = lowerCL, upperCL = upperCL, pval = model$pval, lowerPR = lowerPR, 
                  upperPR = upperPR)
}

get_pred2 <- function(model, mod = " ") {
  name <- as.factor(str_replace(row.names(model$beta), paste0("relevel", "\\(", 
                                                              mod, ", ref = name", "\\)"), ""))
  len <- length(name)
  
  if (len != 1) {
    newdata <- diag(len)
    pred <- predict.rma(model, intercept = FALSE, newmods = newdata[, -1])
  } else {
    pred <- predict.rma(model)
  }
  estimate <- pred$pred
  lowerCL <- pred$ci.lb
  upperCL <- pred$ci.ub
  lowerPR <- pred$cr.lb
  upperPR <- pred$cr.ub
  
  table <- tibble(name = factor(name, levels = name, labels = name), estimate = estimate, 
                  lowerCL = lowerCL, upperCL = upperCL, pval = model$pval, lowerPR = lowerPR, 
                  upperPR = upperPR)
}


mod_tab<-function(m){
# getting marginal R2
  
r2 <- r2_ml(m)

# creating a table
tibble(`Fixed effect` = row.names(m$beta), Estimate = c(m$b), 
       `Lower CI [0.025]` = c(m$ci.lb), `Upper CI  [0.975]` = c(m$ci.ub), 
       `P value` = c(m$pval), R2 = c(r2[1],        rep(NA, (length(m$beta)-1)))) %>% kable("html", digits = 3) %>% kable_styling("striped", position = "left") 
}

uni_mod_plot<-function(m, df, log_ratio, response, variance){
p <- predict.rma(m)
df %>% mutate(ymin = p$ci.lb, 
                                                  ymax = p$ci.ub, ymin2 = p$cr.lb, 
                                                  ymax2 = p$cr.ub, pred = p$pred) %>% 
  ggplot(aes(x = response, y = log_ratio, size = sqrt(1/variance))) + geom_point(shape = 21, alpha= 0.2,
                                                                     fill = "grey90") + 
  geom_hline(yintercept = 0, size = .5, colour = "gray70")+
  geom_smooth(aes(y = ymin2), method = "lm", se = FALSE, lty = "solid", lwd = 0.75, 
              colour = "#0072B2") + geom_smooth(aes(y = ymax2), method = "lm", se = FALSE, 
                                                lty = "solid", lwd = 0.75, colour = "#0072B2") + geom_smooth(aes(y = ymin), 
                                                                                                              method = "lm", se = FALSE, lty = "solid", lwd = 0.75, colour = "#D55E00") + 
  geom_smooth(aes(y = ymax), method = "lm", se = FALSE, lty = "solid", lwd = 0.75, 
              colour = "#D55E00") + geom_smooth(aes(y = pred), method = "lm", se = FALSE, 
                                                lty = "solid", lwd = 1, colour = "black") + 
  labs(x = "\n ln(restoration site age)", y = "ln(restored/unrestored) - mean biodiversity", size = "Precision (1/SE)") + guides(fill = "none", 
                                                                                                                  colour = "none") + # themses
  theme_classic() + theme(legend.position = c(0, 1), legend.justification = c(0, 1)) + theme(legend.direction = "horizontal") + 
  theme(legend.background = element_blank()) + theme(axis.text.y = element_text(size = 8, 
                                                                                colour = "black", hjust = 0.5, angle = 90))+
  coord_cartesian(ylim = c(-2.5, 2.5))+
  scale_y_continuous(limits = c(-2.5, 2.5),
                     breaks = c(-2, -1, 0, 1, 2),
                     labels = c("\n \n -2", "100% decrease \n \n -1", "\n \n 0.0", "100% increase \n \n 1", "\n \n2")) +
  theme(legend.position = "none") 
}


uni_mod_plot_ns<-function(m, df, log_ratio, response, variance){
p <- predict.rma(m)
df %>% mutate(ymin = p$ci.lb, 
                                                  ymax = p$ci.ub, ymin2 = p$cr.lb, 
                                                  ymax2 = p$cr.ub, pred = p$pred) %>% 
  ggplot(aes(x = response, y = log_ratio, size = sqrt(1/variance))) + geom_point(shape = 21, alpha= 0.2,
                                                                     fill = "grey90") + 
  geom_hline(yintercept = 0, size = .5, colour = "gray70")+
  geom_smooth(aes(y = ymin2), method = "lm", se = FALSE, lty = "dashed", lwd = 0.75, 
              colour = "#0072B2") + geom_smooth(aes(y = ymax2), method = "lm", se = FALSE, 
                                                lty = "dashed", lwd = 0.75, colour = "#0072B2") + geom_smooth(aes(y = ymin), 
                                                                                                              method = "lm", se = FALSE, lty = "dashed", lwd = 0.75, colour = "#D55E00") + 
  geom_smooth(aes(y = ymax), method = "lm", se = FALSE, lty = "dashed", lwd = 0.75, 
              colour = "#D55E00") + geom_smooth(aes(y = pred), method = "lm", se = FALSE, 
                                                lty = "dashed", lwd = 1, colour = "black") + 
  labs(x = "\n ln(restoration site age)", y = "ln(restored/unrestored) - mean biodiversity", size = "Precision (1/SE)") + guides(fill = "none", 
                                                                                                                  colour = "none") + # themses
  theme_classic() + theme(legend.position = c(0, 1), legend.justification = c(0, 1)) + theme(legend.direction = "horizontal") + 
  theme(legend.background = element_blank()) + theme(axis.text.y = element_text(size = 8, 
                                                                                colour = "black", hjust = 0.5, angle = 90))+
  coord_cartesian(ylim = c(-2.5, 2.5))+
  scale_y_continuous(limits = c(-2.5, 2.5),
                     breaks = c(-2, -1, 0, 1, 2),
                     labels = c("\n \n -2", "100% decrease \n \n -1", "\n \n 0.0", "100% increase \n \n 1", "\n \n2")) +
  theme(legend.position = "none") 
}


uni_egger_plot_cvr<-function(m, data){# getting marginal R2
r2 <- r2_ml(m)
# getting estimates: name does not work for slopes
est <- get_est(m, mod = "sqrt(vi)")


# creating a table
tibble(`Fixed effect` = row.names(m$beta), Estimate = c(est$estimate), 
       `Lower CI [0.025]` = c(est$lowerCL), `Upper CI  [0.975]` = c(est$upperCL), 
       `P value` = c(m$pval), R2 = c(r2[1], 
                                                              NA)) %>% kable("html", digits = 3) %>% kable_styling("striped", position = "left")


pred <- predict.rma(m)


# plotting
fit <- data %>% drop_na(vi_cvr)%>% mutate(ymin = pred$ci.lb, ymax = pred$ci.ub, ymin2 = pred$cr.lb, ymax2 = pred$cr.ub, pred = pred$pred) %>% 
  ggplot(aes(x = sqrt(vi_cvr), y = yi_cvr, size = sqrt(1/vi_cvr))) + geom_point(shape = 21, fill = "grey90", alpha = 0.3) + 
  geom_smooth(aes(y = ymin2), method = "loess", se = FALSE, lty = "dotted", lwd = 0.25, colour = "#0072B2") + 
  geom_smooth(aes(y = ymax2), method = "loess", se = FALSE, lty = "dotted", lwd = 0.25, colour = "#0072B2") + 
  geom_smooth(aes(y = ymin), method = "loess", se = FALSE, lty = "dotted", lwd = 0.25, colour = "#D55E00") + 
  geom_smooth(aes(y = ymax), method = "loess", se = FALSE, lty = "dotted", lwd = 0.25, colour = "#D55E00") +
  geom_smooth(aes(y = pred), method = "loess", se = FALSE, lty = "dashed", lwd = 0.5, colour = "black") +
  labs(x = "sqrt(sampling variance)", y = "lnRR (effect size)", size = "Precision (1/SE)") + 
  guides(fill = "none", colour = "none") + 
  theme_bw() + theme(legend.position = c(0, 1), legend.justification = c(0, 1)) + theme(legend.direction = "horizontal") + 
  theme(legend.background = element_blank()) + theme(axis.text.y = element_text(size = 10, colour = "black", hjust = 0.5, angle = 90))
fit
}

uni_egger_plot_vr<-function(m, data){# getting marginal R2
r2 <- r2_ml(m)
# getting estimates: name does not work for slopes
est <- get_est(m, mod = "sqrt(vi)")


# creating a table
tibble(`Fixed effect` = row.names(m$beta), Estimate = c(est$estimate), 
       `Lower CI [0.025]` = c(est$lowerCL), `Upper CI  [0.975]` = c(est$upperCL), 
       `P value` = c(m$pval), R2 = c(r2[1], 
                                                              NA)) %>% kable("html", digits = 3) %>% kable_styling("striped", position = "left")


pred <- predict.rma(m)


# plotting
fit <- data %>% drop_na(vi_vr)%>% mutate(ymin = pred$ci.lb, ymax = pred$ci.ub, ymin2 = pred$cr.lb, ymax2 = pred$cr.ub, pred = pred$pred) %>% 
  ggplot(aes(x = sqrt(vi_vr), y = yi_vr, size = sqrt(1/vi_vr))) + geom_point(shape = 21, fill = "grey90", alpha = 0.3) + 
  geom_smooth(aes(y = ymin2), method = "loess", se = FALSE, lty = "dotted", lwd = 0.25, colour = "#0072B2") + 
  geom_smooth(aes(y = ymax2), method = "loess", se = FALSE, lty = "dotted", lwd = 0.25, colour = "#0072B2") + 
  geom_smooth(aes(y = ymin), method = "loess", se = FALSE, lty = "dotted", lwd = 0.25, colour = "#D55E00") + 
  geom_smooth(aes(y = ymax), method = "loess", se = FALSE, lty = "dotted", lwd = 0.25, colour = "#D55E00") +
  geom_smooth(aes(y = pred), method = "loess", se = FALSE, lty = "dashed", lwd = 0.5, colour = "black") +
  labs(x = "sqrt(sampling variance)", y = "lnRR (effect size)", size = "Precision (1/SE)") + 
  guides(fill = "none", colour = "none") + 
  theme_bw() + theme(legend.position = c(0, 1), legend.justification = c(0, 1)) + theme(legend.direction = "horizontal") + 
  theme(legend.background = element_blank()) + theme(axis.text.y = element_text(size = 10, colour = "black", hjust = 0.5, angle = 90))
fit
}

uni_egger_plot_mean<-function(m, data){# getting marginal R2
r2 <- r2_ml(m)
# getting estimates: name does not work for slopes
est <- get_est(m, mod = "sqrt(vi)")

# creating a table
tibble(`Fixed effect` = row.names(m$beta), Estimate = c(est$estimate), 
       `Lower CI [0.025]` = c(est$lowerCL), `Upper CI  [0.975]` = c(est$upperCL), 
       `P value` = c(m$pval), R2 = c(r2[1], 
                                                              NA)) %>% kable("html", digits = 3) %>% kable_styling("striped", position = "left")


pred <- predict.rma(m)


# plotting
fit <- data %>% drop_na(vi_mean)%>% mutate(ymin = pred$ci.lb, ymax = pred$ci.ub, ymin2 = pred$cr.lb, ymax2 = pred$cr.ub, pred = pred$pred) %>% 
  ggplot(aes(x = sqrt(vi_mean), y = yi_mean, size = sqrt(1/vi_mean))) + geom_point(shape = 21, fill = "grey90", alpha = 0.3) + 
  geom_smooth(aes(y = ymin2), method = "loess", se = FALSE, lty = "dotted", lwd = 0.25, colour = "#0072B2") + 
  geom_smooth(aes(y = ymax2), method = "loess", se = FALSE, lty = "dotted", lwd = 0.25, colour = "#0072B2") + 
  geom_smooth(aes(y = ymin), method = "loess", se = FALSE, lty = "dotted", lwd = 0.25, colour = "#D55E00") + 
  geom_smooth(aes(y = ymax), method = "loess", se = FALSE, lty = "dotted", lwd = 0.25, colour = "#D55E00") +
  geom_smooth(aes(y = pred), method = "loess", se = FALSE, lty = "dashed", lwd = 0.5, colour = "black") +
  labs(x = "sqrt(sampling variance)", y = "lnRR (effect size)", size = "Precision (1/SE)") + 
  guides(fill = "none", colour = "none") + 
  theme_bw() + theme(legend.position = c(0, 1), legend.justification = c(0, 1)) + theme(legend.direction = "horizontal") + 
  theme(legend.background = element_blank()) + theme(axis.text.y = element_text(size = 10, colour = "black", hjust = 0.5, angle = 90))
fit
}


I2 <- function(model, method = c("Wolfgang", "Shinichi")) {
    
    ## evaluate choices
    method <- match.arg(method)
    
    # Wolfgang's method
    if (method == "Wolfgang") {
        W <- solve(model$V)
        X <- model.matrix(model)
        P <- W - W %*% X %*% solve(t(X) %*% W %*% X) %*% t(X) %*% W
        I2_total <- sum(model$sigma2)/(sum(model$sigma2) + (model$k - model$p)/sum(diag(P)))
        I2_each <- model$sigma2/(sum(model$sigma2) + (model$k - model$p)/sum(diag(P)))
        names(I2_each) = paste0("I2_", model$s.names)
        
        # putting all together
        I2s <- c(I2_total = I2_total, I2_each)
        
        # or my way
    } else {
        # sigma2_v = typical sampling error variance
        sigma2_v <- sum(1/model$vi) * (model$k - 1)/(sum(1/model$vi)^2 - sum((1/model$vi)^2))
        I2_total <- sum(model$sigma2)/(sum(model$sigma2) + sigma2_v)  #s^2_t = total variance
        I2_each <- model$sigma2/(sum(model$sigma2) + sigma2_v)
        names(I2_each) = paste0("I2_", model$s.names)
        
        # putting all together
        I2s <- c(I2_total = I2_total, I2_each)
    }
    return(I2s)
}
```

The meta-analytic dataset is available at https://osf.io/4aucp/.

## Literature search

The core details of the literature search are included in the main
body of the manuscript so are not included here. However, there are some
additional details that are important to note.

Titles of results were screened for any non-terrestrial studies by
removal of titles containing the following words: aquatic, stream,
river, marine, ocean, lake, fish, wetland, saltmarsh, pond, coral, reef,
plankton. Other useful blanket screening terms include, “prioritiz\*”,
“implications for”, which helped to identify empirical studies not of
restoration sites but of experiments with potential implications for
ecological restoration. This removed 555 records for a total of 1721
studies remaining. Titles were then individually screened in detail to
only include studies that clearly assessed restoration outcomes using
some form of biodiversity. Where study titles were ambiguous they were
not removed. Only obviously irrelevant studies were removed at this
stage. This removed a further 1137 studies.

All screening was conducted by lead author JA.

We did not record a measure of the individual quality of studies
included in the meta-analysis (e.g. blinded data collection, reporting
quality, and experimental vs observational).

See below for a PRISMA diagram showing the workflow for the
literature search.

**Figure S1.** PRISMA diagram detailing literature
screening process (formatted according to Page et al. 2020)

## Meta-analysis: the effect of restoration on variability in biodiversity

### Choosing effect size statistics: checking the mean-variance relationship

We checked the mean-variance relationship in our data. If there is
such a relationship, it is better to use the logarithm of response
ratio, lnRR rather than standardized mean difference (often known as
Cohen’s *d* or Hedges’ *g*) because the latter assumes the
homogeneity of variance.

```
full_data<-read.csv("Data/variation_data.csv", stringsAsFactors=FALSE)

# A)
dat_t<-full_data %>% drop_na(t_mean) # some have NA values here so log transform produces NAs, creating clean dataset for each plot
dat_c<-full_data %>% drop_na(c_mean) %>% filter(c_sd != 0) # t = treatment (restored), c = control (unrestored), r = reference (some SD = 0 in control dataset)
dat_r<-full_data %>% drop_na(r_mean)

cor_1 <- round(with(dat_t,cor(log(t_mean), log(t_sd))), 3)
plot_res <- ggplot(dat_t, aes(log(t_mean), log(t_sd))) + geom_point() +
  geom_smooth(method = "lm") + 
  labs(x = "ln(mean[experiment])", 
       y = "ln(SD[experiment])", 
       title = "Restoration sites mean vs variance (sd)") +
  xlim(-5, 7.5) + ylim(-9, 9) + annotate('text',x = 7.5, y = -8, label = paste("r = ", cor_1))

# B)
cor_2 <- round(with(dat_c,cor(log(c_mean), log(c_sd))), 3)
plot_con <- ggplot(dat_c, aes(log(c_mean), log(c_sd))) + geom_point() +
  geom_smooth(method = "lm") + 
  labs(x = "ln(mean[control])", 
       y = "ln(SD[control])",
       title = "Unrestored sites mean vs variance (sd)")+
  xlim(-5, 7.5) + ylim(-9, 9) + annotate('text',x = 7.5, y = -8, label = paste("r = ", cor_2))

# c)
cor_3 <- round(with(dat_r,cor.test(log(r_mean), log(r_sd)))$estimate[[1]], 3)
plot_ref <- ggplot(dat_r, aes(log(r_mean), log(r_sd))) + geom_point() +
  geom_smooth(method = "lm") + 
  labs(x = "ln(mean[experiment])", 
       y = "ln(SD[experiment])",
       title = "Reference sites mean vs variance (sd)")+
  xlim(-5, 7.5) + ylim(-9, 9) + annotate('text',x = 7.5, y = -8, label = paste("r = ", cor_3))


mean_SD <- (plot_res / plot_con / plot_ref) +
  plot_annotation(tag_levels = "A", tag_suffix = ")")

mean_SD
```

**Figure S2:** Correlations between mean and variance in
the restored sites, unrestored sites, and reference sites.

### Calculating effect sizes

We found extremely strong correlations between mean and variance
(standard deviation) on the log scale above, so instead, we report
differences in variability within-studies as the difference in lnCVR
(the log of the coefficient of variation ratio. We use the log response
ratio to compare mean differences for a range of reasons outlined in the
methods of the main body of the paper, and as is illustrated further
below, it is also less sensitive to scale bias.

Effect sizes are calculated using `escalc` function in
`metafor`. Here we calculate effect sizes for two separate
meta-analyses, comparing restored sites to control (unrestored) sites,
and comparing restored sites to reference (goal) sites. We also use the
`make_VCV_matrix` function to calculate the
variance-covariance matrix to use in the model in place of the error
term `vi`. The code essentially performs the same procedure
for caluclating effect sizes twice. We also calculate lnVR (the log
variability ratio that uses SD), despite the correlations shown above,
to present alongside the main results (though these are not presented in
the body of the paper).

```
###########################
## UNRESTORED / RESTORED ##
###########################

# un_re = unrestored / restored 

un_re<-read.csv("Data/variation_data.csv", stringsAsFactors = F)

un_re$c_quad_n = as.numeric(un_re$c_quad_n)
un_re$c_mean =  as.numeric(un_re$c_mean)
un_re$c_sd = as.numeric(un_re$c_sd)

#remove studies with only a restored sites comparison
un_re<-un_re[!is.na(un_re$c_mean),]
#un_re %>% group_by(id, c_mean, c_sd) %>% distinct(shared_ctrl) %>% filter(n()>1) # checking shared controls is accurate

#calculate the lnCVR and lnRR and lnVR effect size and un_reiance with escalc
CVR<-escalc(measure = "CVR", n1i = un_re$t_quad_n, n2i = un_re$c_quad_n, m1i = un_re$t_mean, m2i = un_re$c_mean, sd1i = un_re$t_sd, sd2i = un_re$c_sd)
lnRR<-escalc(measure = "ROM", n1i = un_re$t_quad_n, n2i = un_re$c_quad_n, m1i = un_re$t_mean, m2i = un_re$c_mean, sd1i = un_re$t_sd, sd2i = un_re$c_sd)
lnVR<-escalc(measure = "VR", n1i = un_re$t_quad_n, n2i = un_re$c_quad_n, m1i = un_re$t_mean, m2i = un_re$c_mean, sd1i = un_re$t_sd, sd2i = un_re$c_sd)

#combined effect sizes with relevant un_rea frames
un_re <-bind_cols(un_re, lnRR, lnVR, CVR)

# name the un_rea something meaningful and remove all the columns unneeded
un_re<-un_re %>% rename(yi_mean = yi...36, vi_mean = vi...37, yi_vr = yi...38, vi_vr = vi...39, yi_cvr = yi...40, vi_cvr = vi...41)

#remove studies that have vi=NA - usually where control SD = 0
un_re<-un_re[!is.na(un_re$vi_vr),]

un_re$plu<-as.factor(un_re$plu)
un_re$plu<-relevel(un_re$plu, "semi-natural")

#need another random factor for 'unit'

unit <- factor(1:length(un_re$yi_mean))
un_re$unit <- unit

vcv_cvr<-make_VCV_matrix(un_re, V ="vi_cvr", "shared_ctrl", "unit", rho=0.5)
vcv_mean<-make_VCV_matrix(un_re, V ="vi_mean", "shared_ctrl", "unit", rho=0.5)
vcv_vr<-make_VCV_matrix(un_re, V ="vi_vr", "shared_ctrl", "unit", rho=0.5)


##########################
## RESTORED / REFERENCE ##
##########################

# re_ref = restored / reference 

re_ref<-read.csv("Data/variation_data.csv", stringsAsFactors = F)

#remove studies with only a degraded site comparison
re_ref<-re_ref[!is.na(re_ref$r_mean),]

#remove studies that have vi=NA - usually where control SD = 0
re_ref<-re_ref %>% filter(r_sd != 0)
re_ref<-re_ref %>% filter(!is.na(r_sd))

# there is a few sites where the reference control is shared, but the degraded one is not, need to add a "ref_shared_ctrl" to correct this
re_ref<-re_ref %>% group_by(id, r_mean, r_sd) %>% mutate(ref_shared_ctrl = cur_group_id())
#re_ref %>% group_by(id, r_mean, r_sd) %>% distinct(ref_shared_ctrl) %>% filter(n()>1) # to check any errors in the shared_control tagging


re_ref$r_quad_n = as.numeric(re_ref$r_quad_n)
re_ref$r_mean =  as.numeric(re_ref$r_mean)
re_ref$r_sd = as.numeric(re_ref$r_sd)

re_ref<-re_ref %>% filter(r_quad_n > 1) # a few sample sizes of 1 or 0?


#calculate the lnCVR and lnRR effect size and re_refiance with escalc
CVR<-escalc(measure = "CVR", n1i = re_ref$t_quad_n, n2i = re_ref$r_quad_n, m1i = re_ref$t_mean, m2i = re_ref$r_mean, sd1i = re_ref$t_sd, sd2i = re_ref$r_sd)
lnRR<-escalc(measure = "ROM", n1i = re_ref$t_quad_n, n2i = re_ref$r_quad_n, m1i = re_ref$t_mean, m2i = re_ref$r_mean, sd1i = re_ref$t_sd, sd2i = re_ref$r_sd)
lnVR<-escalc(measure = "VR", n1i = re_ref$t_quad_n, n2i = re_ref$r_quad_n, m1i = re_ref$t_mean, m2i = re_ref$r_mean, sd1i = re_ref$t_sd, sd2i = re_ref$r_sd)


#combined effect sizes with relevant data frames
re_ref <-bind_cols(re_ref, lnRR, lnVR, CVR)
# name the data something meaningful and remove all the columns unneeded
re_ref<-re_ref %>% rename(yi_mean = yi...37, vi_mean = vi...38, yi_vr = yi...39, vi_vr = vi...40, yi_cvr = yi...41, vi_cvr = vi...42)


re_ref$plu<-as.factor(re_ref$plu)
re_ref$plu<-relevel(re_ref$plu, "semi-natural")

#need another random factor for 'unit'

unit <- factor(1:length(re_ref$yi_mean))
re_ref$unit <- unit

re_ref<-as.data.frame(re_ref) # the group_by to do the shared control check above turns this bad boy into a tibble, needs to be a dataframe for the below function

vcv_cvr_rr<-make_VCV_matrix(data = re_ref, V ="vi_cvr", cluster = "ref_shared_ctrl", obs = "unit", rho=0.5)
vcv_mean_rr<-make_VCV_matrix(re_ref, V ="vi_mean", "ref_shared_ctrl", "unit", rho=0.5)
vcv_vr_rr<-make_VCV_matrix(re_ref, V ="vi_vr", "ref_shared_ctrl", "unit", rho=0.5)
```

## Meta-analytic models: lnCVR, lnVR and lnRR

We conducted meta-analyses (i.e. ran the intercept models) using the
`rma.mv` function in `metafor`. For every model
(lnCVR, lnRR, and lnVR), we conduct the same model a second time
including the sampling scale (measured as quadrat size) to see if the
results are robust to variation in sampling scale. Note that this
reduces the sample size of the model overall in all cases as not all
biodiversity sampling methods have a comparable scale (e.g. butterfly
net sweeps, linear transects).

```
# "mean" in model name refers to lnRR, vr = lnVR, cvr = lnCVR
# ur = unrestored/restored comparison, rr = restored/reference comparison, q = quadrat size included
un_re$ln_qsize<-log(un_re$t_qsize_m2)
re_ref$ln_qsize<-log(re_ref$t_qsize_m2)

cvr_ur <- rma.mv(yi_cvr, vcv_cvr, random = list(~1 | id, ~1 | plot_id, ~1 | unit), method = "REML", data = un_re)
cvr_q_ur <- rma.mv(yi_cvr, vcv_cvr, mods= ~ln_qsize - 1, random = list(~1 | id, ~1 | plot_id, ~1 | unit), method = "REML", data = un_re)
vr_ur <- rma.mv(yi_vr, vcv_vr, random = list(~1 | id, ~1 | plot_id, ~1 | unit), method = "REML", data = un_re)
vr_q_ur <- rma.mv(yi_vr, vcv_vr, mods= ~ln_qsize - 1, random = list(~1 | id, ~1 | plot_id, ~1 | unit), method = "REML", data = un_re)
mean_ur <- rma.mv(yi_mean, vcv_mean, random = list(~1 | id, ~1 | plot_id, ~1 | unit), method = "REML", data = un_re)
mean_q_ur <- rma.mv(yi_mean, vcv_mean, mods= ~ln_qsize - 1, random = list(~1 | id, ~1 | plot_id, ~1 | unit), method = "REML", data = un_re)

cvr_rr <- rma.mv(yi_cvr, vcv_cvr_rr, random = list(~1 | id, ~1 | plot_id, ~1 | unit), method = "REML", data = re_ref)
cvr_q_rr <- rma.mv(yi_cvr, vcv_cvr_rr, mods= ~ln_qsize - 1, random = list(~1 | id, ~1 | plot_id, ~1 | unit), method = "REML", data = re_ref)
vr_rr <- rma.mv(yi_vr, vcv_vr_rr, random = list(~1 | id, ~1 | plot_id, ~1 | unit), method = "REML", data = re_ref)
vr_q_rr <- rma.mv(yi_vr, vcv_vr_rr, mods= ~ln_qsize - 1, random = list(~1 | id, ~1 | plot_id, ~1 | unit), method = "REML", data = re_ref)
mean_rr <- rma.mv(yi_mean, vcv_mean_rr, random = list(~1 | id, ~1 | plot_id, ~1 | unit), method = "REML", data = re_ref)
mean_q_rr <- rma.mv(yi_mean, vcv_mean_rr, mods= ~ln_qsize - 1, random = list(~1 | id, ~1 | plot_id, ~1 | unit), method = "REML", data = re_ref)
```

**Table S1:** Overall effects (meta-analytic means), 95%
confidence intervals (CIs) and 95% prediction intervals (95%). lnCVR =
log CV ratio (coefficient of variation), lnRR = log response ratio
(mean), lnVR = log variation ratio (SD).

```
# getting a table of CI and PI

pred_cvr_ur <- get_pred1(cvr_ur, mod = "Int")
pred_vr_ur <- get_pred1(vr_ur, mod = "Int")
pred_mean_ur <- get_pred1(mean_ur, mod = "Int")
pred_cvr_rr <- get_pred1(cvr_rr, mod = "Int")
pred_vr_rr <- get_pred1(vr_rr, mod = "Int")
pred_mean_rr <- get_pred1(mean_rr, mod = "Int")


# Drawing a table for meta-analyses
tibble(`Effect size` = c("lnCVR - unrestored", "lnVR - unrestored", "lnRR - unrestored", "lnCVR - reference", "lnVR - reference", "lnRR - referemce"), 
       `Overall mean` = c(pred_cvr_ur$estimate, pred_vr_ur$estimate, pred_mean_ur$estimate, pred_cvr_rr$estimate, pred_vr_rr$estimate, pred_mean_rr$estimate), 
       `Lower CI [0.025]` = c(pred_cvr_ur$lowerCL, pred_vr_ur$lowerCL, pred_mean_ur$lowerCL, pred_cvr_rr$lowerCL, pred_vr_rr$lowerCL, pred_mean_rr$lowerCL), 
       `Upper CI [0.975]` = c(pred_cvr_ur$upperCL, pred_vr_ur$upperCL, pred_mean_ur$upperCL, pred_cvr_rr$upperCL, pred_vr_rr$upperCL, pred_mean_rr$upperCL),
       `P value`          = c(pred_cvr_ur$pval, pred_vr_ur$pval, pred_mean_ur$pval,pred_cvr_rr$pval, pred_vr_rr$pval, pred_mean_rr$pval),
       `Lower PI [0.025]` = c(pred_cvr_ur$lowerPR, pred_vr_ur$lowerPR, pred_mean_ur$lowerPR, pred_cvr_rr$lowerPR, pred_vr_rr$lowerPR, pred_mean_rr$lowerPR), 
       `Upper PI [0.975]` = c(pred_cvr_ur$upperPR, pred_vr_ur$upperPR, pred_mean_ur$upperPR, pred_cvr_rr$upperPR, pred_vr_rr$upperPR, pred_mean_rr$upperPR)) %>% 
  kable("html", digits = 3) %>% 
  kable_styling("striped", position = "left")%>%
    scroll_box(width = "800px", height = "300px")
```

| Effect size | Overall mean | Lower CI [0.025] | Upper CI [0.975] | P value | Lower PI [0.025] | Upper PI [0.975] |
| --- | --- | --- | --- | --- | --- | --- |
| lnCVR - unrestored | -0.152 | -0.248 | -0.055 | 0.002 | -1.071 | 0.768 |
| lnVR - unrestored | 0.079 | -0.031 | 0.190 | 0.158 | -0.955 | 1.113 |
| lnRR - unrestored | 0.182 | 0.112 | 0.251 | 0.000 | -0.425 | 0.788 |
| lnCVR - reference | 0.183 | 0.051 | 0.315 | 0.007 | -1.004 | 1.370 |
| lnVR - reference | 0.048 | -0.056 | 0.152 | 0.368 | -0.981 | 1.076 |
| lnRR - referemce | -0.139 | -0.218 | -0.061 | 0.000 | -1.007 | 0.728 |

**Table S2:** Heterogeneity among effects of
restoration, measured using I^2.

```
cvrur<-I2(cvr_ur)
vrur<-I2(vr_ur)
meanur<-I2(mean_ur)
cvrrr<-I2(cvr_rr)
vrrr<-I2(vr_rr)
meanrr<-I2(mean_rr)

tbl<-rbind(cvrur, vrur, meanur, cvrrr, vrrr, meanrr)
tbl<-as.data.frame(tbl)
tbl$Model<-c("LnCVR - unrestored/restored", "LnVR - unrestored/restored","LnRR - unrestored/restored",
             "LnCVR - reference/restored", "LnVR - reference/restored", "LnRR - reference/restored")
tbl %>% kable("html", digits = 4) %>% 
  kable_styling("striped", position = "left")
```

|  | I2\_total | I2\_id | I2\_plot\_id | I2\_unit | Model |
| --- | --- | --- | --- | --- | --- |
| cvrur | 0.6717 | 0.1461 | 0.2175 | 0.3081 | LnCVR - unrestored/restored |
| vrur | 0.7712 | 0.2642 | 0.1750 | 0.3320 | LnVR - unrestored/restored |
| meanur | 0.9979 | 0.5724 | 0.0163 | 0.4093 | LnRR - unrestored/restored |
| cvrrr | 0.8014 | 0.4050 | 0.0708 | 0.3256 | LnCVR - reference/restored |
| vrrr | 0.7853 | 0.2785 | 0.0473 | 0.4595 | LnVR - reference/restored |
| meanrr | 0.9994 | 0.3454 | 0.0039 | 0.6501 | LnRR - reference/restored |

### Univariate models of age, size, and past land use

```
 ######
# SIZE #
 ######


# getting a table of CI and PI

un_re$site_size<-ifelse(un_re$site_size == 0, 0.1, un_re$site_size)
un_re_sz<-un_re %>% drop_na(site_size)

# need a new vcv matrix because above reduces the sample slightly (NA age removal)

vcv_cvr_sz<-make_VCV_matrix(un_re_sz, V ="vi_cvr", "shared_ctrl", "unit", rho=0.5)
vcv_mean_sz<-make_VCV_matrix(un_re_sz, V ="vi_mean", "shared_ctrl", "unit", rho=0.5)
vcv_vr_sz<-make_VCV_matrix(un_re_sz, V ="vi_vr", "shared_ctrl", "unit", rho=0.5)

mean_size_ur <- rma.mv(yi_mean, vcv_mean_sz, mods = ~log(site_size), random = list(~1 | id, ~1 | plot_id, ~1 | unit), method = "REML", data = un_re_sz)
vr_size_ur <- rma.mv(yi_vr, vcv_vr_sz, mods = ~log(site_size), random = list(~1 | id, ~1 | plot_id, ~1 | unit), method = "REML", data = un_re_sz)
cvr_size_ur <- rma.mv(yi_cvr, vcv_cvr_sz, mods = ~log(site_size), random = list(~1 | id, ~1 | plot_id, ~1 | unit), method = "REML", data = un_re_sz)
mean_size_ur_q <- rma.mv(yi_mean, vcv_mean_sz, mods = ~log(site_size) + log(t_qsize_m2), random = list(~1 | id, ~1 | plot_id, ~1 | unit), method = "REML", data = un_re_sz)
vr_size_ur_q <- rma.mv(yi_vr, vcv_vr_sz, mods = ~log(site_size) + log(t_qsize_m2), random = list(~1 | id, ~1 | plot_id, ~1 | unit), method = "REML", data = un_re_sz)
cvr_size_ur_q <- rma.mv(yi_cvr, vcv_cvr_sz, mods = ~log(site_size) + log(t_qsize_m2), random = list(~1 | id, ~1 | plot_id, ~1 | unit), method = "REML", data = un_re_sz)


# need a new vcv matrix because above reduces the sample slightly (NA size removal)

re_ref$site_size<-ifelse(re_ref$site_size == 0, 0.1, re_ref$site_size)
re_ref_sz<-re_ref %>% drop_na(site_size)

vcv_cvr_rr_sz<-make_VCV_matrix(re_ref_sz, V ="vi_cvr", "shared_ctrl", "unit", rho=0.5)
vcv_mean_rr_sz<-make_VCV_matrix(re_ref_sz, V ="vi_mean", "shared_ctrl", "unit", rho=0.5)
vcv_vr_rr_sz<-make_VCV_matrix(re_ref_sz, V ="vi_vr", "shared_ctrl", "unit", rho=0.5)

mean_size_rr <- rma.mv(yi_mean, vcv_mean_rr_sz, mods = ~log(site_size), random = list(~1 | id, ~1 | plot_id, ~1 | unit), method = "REML", data = re_ref_sz)
vr_size_rr <- rma.mv(yi_vr, vcv_vr_rr_sz, mods = ~log(site_size), random = list(~1 | id, ~1 | plot_id, ~1 | unit), method = "REML", data = re_ref_sz)
cvr_size_rr <- rma.mv(yi_cvr, vcv_cvr_rr_sz, mods = ~log(site_size), random = list(~1 | id, ~1 | plot_id, ~1 | unit), method = "REML", data = re_ref_sz)
mean_size_rr_q <- rma.mv(yi_mean, vcv_mean_rr_sz, mods = ~log(site_size)+ log(t_qsize_m2), random = list(~1 | id, ~1 | plot_id, ~1 | unit), method = "REML", data = re_ref_sz)
vr_size_rr_q <- rma.mv(yi_vr, vcv_vr_rr_sz, mods = ~log(site_size)+ log(t_qsize_m2), random = list(~1 | id, ~1 | plot_id, ~1 | unit), method = "REML", data = re_ref_sz)
cvr_size_rr_q <- rma.mv(yi_cvr, vcv_cvr_rr_sz, mods = ~log(site_size)+ log(t_qsize_m2), random = list(~1 | id, ~1 | plot_id, ~1 | unit), method = "REML", data = re_ref_sz)


 #####
# AGE #
 #####

un_re$age.rest.<-ifelse(un_re$age.rest. == 0, 0.1, un_re$age.rest.)
un_re_ag<-un_re %>% tidyr::drop_na(age.rest.)

# need a new vcv matrix because above reduces the sample slightly (NA age removal)

vcv_cvr_ag<-make_VCV_matrix(un_re_ag, V ="vi_cvr", "shared_ctrl", "unit", rho=0.5)
vcv_mean_ag<-make_VCV_matrix(un_re_ag, V ="vi_mean", "shared_ctrl", "unit", rho=0.5)
vcv_vr_ag<-make_VCV_matrix(un_re_ag, V ="vi_vr", "shared_ctrl", "unit", rho=0.5)

mean_age_ur <- rma.mv(yi_mean, vcv_mean_ag, mods = ~(age.rest.), random = list(~1 | id, ~1 | plot_id, ~1 | unit), method = "REML", data = un_re_ag)
vr_age_ur <- rma.mv(yi_vr, vcv_vr_ag, mods = ~(age.rest.) , random = list(~1 | id, ~1 | plot_id, ~1 | unit), method = "REML", data = un_re_ag)
cvr_age_ur <- rma.mv(yi_cvr, vcv_cvr_ag, mods = ~(age.rest.), random = list(~1 | id, ~1 | plot_id, ~1 | unit), method = "REML", data = un_re_ag)
mean_age_ur_q <- rma.mv(yi_mean, vcv_mean_ag, mods = ~(age.rest.)+ log(t_qsize_m2), random = list(~1 | id, ~1 | plot_id, ~1 | unit), method = "REML", data = un_re_ag)
vr_age_ur_q <- rma.mv(yi_vr, vcv_vr_ag, mods = ~(age.rest.) + log(t_qsize_m2), random = list(~1 | id, ~1 | plot_id, ~1 | unit), method = "REML", data = un_re_ag)
cvr_age_ur_q <- rma.mv(yi_cvr, vcv_cvr_ag, mods = ~(age.rest.)+ log(t_qsize_m2), random = list(~1 | id, ~1 | plot_id, ~1 | unit), method = "REML", data = un_re_ag)


# need a new vcv matrix because above reduces the sample slightly (NA age removal)

re_ref$age.rest.<-ifelse(re_ref$age.rest. == 0, 0.1, re_ref$age.rest.)
re_ref_ag<-re_ref %>% tidyr::drop_na(age.rest.)

vcv_cvr_rr_ag<-make_VCV_matrix(re_ref_ag, V ="vi_cvr", "shared_ctrl", "unit", rho=0.5)
vcv_mean_rr_ag<-make_VCV_matrix(re_ref_ag, V ="vi_mean", "shared_ctrl", "unit", rho=0.5)
vcv_vr_rr_ag<-make_VCV_matrix(re_ref_ag, V ="vi_vr", "shared_ctrl", "unit", rho=0.5)

mean_age_rr <- rma.mv(yi_mean, vcv_mean_rr_ag, mods = ~(age.rest.), random = list(~1 | id, ~1 | plot_id, ~1 | unit), method = "REML", data = re_ref_ag)
vr_age_rr <- rma.mv(yi_vr, vcv_vr_rr_ag, mods = ~(age.rest.) , random = list(~1 | id, ~1 | plot_id, ~1 | unit), method = "REML", data = re_ref_ag)
cvr_age_rr <- rma.mv(yi_cvr, vcv_cvr_rr_ag, mods = ~(age.rest.), random = list(~1 | id, ~1 | plot_id, ~1 | unit), method = "REML", data = re_ref_ag)
mean_age_rr_q <- rma.mv(yi_mean, vcv_mean_rr_ag, mods = ~(age.rest.)+ log(t_qsize_m2), random = list(~1 | id, ~1 | plot_id, ~1 | unit), method = "REML", data = re_ref_ag)
vr_age_rr_q <- rma.mv(yi_vr, vcv_vr_rr_ag, mods = ~(age.rest.) + log(t_qsize_m2), random = list(~1 | id, ~1 | plot_id, ~1 | unit), method = "REML", data = re_ref_ag)
cvr_age_rr_q <- rma.mv(yi_cvr, vcv_cvr_rr_ag, mods = ~(age.rest.)+ log(t_qsize_m2), random = list(~1 | id, ~1 | plot_id, ~1 | unit), method = "REML", data = re_ref_ag)


 #####
# PLU #
 #####
 

mean_plu_ur <- rma.mv(yi_mean, vcv_mean, mods = ~plu - 1 , random = list(~1 | id, ~1 | plot_id, ~1 | unit), method = "REML", data = un_re)
vr_plu_ur <- rma.mv(yi_vr, vcv_vr, mods = ~plu - 1, random = list(~1 | id, ~1 | plot_id, ~1 | unit), method = "REML", data = un_re)
cvr_plu_ur <- rma.mv(yi_cvr, vcv_cvr, mods = ~plu - 1, random = list(~1 | id, ~1 | plot_id, ~1 | unit), method = "REML", data = un_re)
mean_plu_ur_q <- rma.mv(yi_mean, vcv_mean, mods = ~plu + log(t_qsize_m2) - 1, random = list(~1 | id, ~1 | plot_id, ~1 | unit), method = "REML", data = un_re)
vr_plu_ur_q <- rma.mv(yi_vr, vcv_vr, mods = ~plu + log(t_qsize_m2)- 1, random = list(~1 | id, ~1 | plot_id, ~1 | unit), method = "REML", data = un_re)
cvr_plu_ur_q <- rma.mv(yi_cvr, vcv_cvr, mods = ~plu + log(t_qsize_m2)- 1, random = list(~1 | id, ~1 | plot_id, ~1 | unit), method = "REML", data = un_re)


mean_plu_rr <- rma.mv(yi_mean, vcv_mean_rr, mods = ~plu - 1, random = list(~1 | id, ~1 | plot_id, ~1 | unit), method = "REML", data = re_ref)
vr_plu_rr <- rma.mv(yi_vr, vcv_vr_rr, mods = ~plu - 1, random = list(~1 | id, ~1 | plot_id, ~1 | unit), method = "REML", data = re_ref)
cvr_plu_rr <- rma.mv(yi_cvr, vcv_cvr_rr, mods = ~plu - 1, random = list(~1 | id, ~1 | plot_id, ~1 | unit), method = "REML", data = re_ref)
mean_plu_rr_q <- rma.mv(yi_mean, vcv_mean_rr, mods = ~plu + log(t_qsize_m2)- 1, random = list(~1 | id, ~1 | plot_id, ~1 | unit), method = "REML", data = re_ref)
vr_plu_rr_q <- rma.mv(yi_vr, vcv_vr_rr, mods = ~plu + log(t_qsize_m2)- 1, random = list(~1 | id, ~1 | plot_id, ~1 | unit), method = "REML", data = re_ref)
cvr_plu_rr_q <- rma.mv(yi_cvr, vcv_cvr_rr, mods = ~plu + log(t_qsize_m2)- 1, random = list(~1 | id, ~1 | plot_id, ~1 | unit), method = "REML", data = re_ref)
```

## Manuscript plots

### Location of studies

Our study sites had a global distribution, however with some clear
biases towards the Global North (particularly North America and Europe),
with the continents of Africa, Asia and South America poorly
represented.

```
library(ggplot2)  # ggplot() fortify()
library(rworldmap)  # getMap()


#load spatial data
spatial_data <- read_csv("Data/studies.csv")
world <- getMap(resolution = "high")


ggplot() +
  geom_polygon(data=map_data('world'), mapping=aes(x=long, y=lat, group=group), fill="gray90", colour="gray70", size = 0.25) + 
  geom_point(data = spatial_data, aes(x = Y, y = X), shape= 19, color = "red4",  size = 1)+
  theme_bw(base_size = 15)+
  coord_equal()+
  ylim(-60, 90) +
  xlim(-179, 195) +
 theme(axis.title = element_blank(),
       axis.ticks = element_blank(),
       axis.text = element_blank(),
       panel.grid.major = element_blank(), panel.grid.minor = element_blank())
```

```
#ggsave("Figure_1.pdf", height = 4, width = 8)
```

**Figure S4.** Location of studies included in the
meta-analysis.

### Meta-analytic model

I^2 (heterogeneity index) values are calculated within the code
chunk.

```
# drawing plots

i2cvrur<-I2(cvr_ur)
 p1 <- orchard_plot(cvr_ur, mod="Int", xlab = "log(CV ratio) - unrestored/restored", alpha = 0.05, k = TRUE) +
  scale_y_discrete(labels = "Overall mean") + 
  scale_fill_manual(values="green4") +
  scale_colour_manual(values="green4") +
  coord_cartesian(xlim = c(-3, 3)) + theme(legend.position = "none")+
  geom_richtext(x = 2,
           y = 0.7,
           label = paste('<i>N<sub>effect size</sub</i> =', cvr_ur$k.all), size =3, label.size  = NA)
 
i2vrur<-I2(vr_ur)
p2 <- orchard_plot(vr_ur, mod="Int", xlab = "log(variability ratio) - unrestored/restored", alpha = 0.1, k=F) +
  scale_y_discrete(labels = "Overall mean") +
  scale_fill_manual(values="red") +
  scale_colour_manual(values="red") +
  coord_cartesian(xlim = c(-3, 3))+ theme(legend.position = "none")+
  geom_richtext(x = 2,
           y = 0.7,
           label = paste('<i>N<sub>effect size</sub</i> =', vr_ur$k.all), size =3, label.size  = NA)

i2meanur<-I2(mean_ur)
p3 <- orchard_plot(mean_ur, mod="Int", xlab = "log(Response ratio) - unrestored/restored", alpha = 0.1, k=F) +
  scale_y_discrete(labels = "Overall mean") + 
  scale_fill_manual(values="purple") +
  scale_colour_manual(values="purple") +
  coord_cartesian(xlim = c(-3, 3))+ theme(legend.position = "none")+
  geom_richtext(x = 2,
           y = 0.7,
           label = paste('<i>N<sub>effect size</sub</i> =', mean_ur$k.all), size =3, label.size  = NA)

i2cvrrr<-I2(cvr_rr)
p4 <- orchard_plot(cvr_rr, mod="Int", xlab = "log(CV ratio) - reference/restored", alpha = 0.05, k=F) +
  scale_y_discrete(labels = "Overall mean") +
  scale_fill_manual(values="green4") +
  scale_colour_manual(values="green4") +
  coord_cartesian(xlim = c(-3, 3))+ theme(legend.position = "none")+
  geom_richtext(x = 2,
           y = 0.7,
           label = paste('<i>N<sub>effect size</sub</i> =', cvr_rr$k.all), size =3, label.size  = NA)

i2vrrr<-I2(vr_rr)
p5 <- orchard_plot(vr_rr, mod="Int", xlab = "log(variability ratio) - reference/restored", alpha = 0.1, k=F) +
  scale_y_discrete(labels = "Overall mean") + 
  scale_fill_manual(values="red") +
  scale_colour_manual(values="red") +
  coord_cartesian(xlim = c(-3, 3))+ theme(legend.position = "none") +
  geom_richtext(x = 2,
           y = 0.7,
           label = paste('<i>N<sub>effect size</sub</i> =', vr_rr$k.all), size =3, label.size  = NA)

i2meanrr<-I2(mean_rr)
p6 <- orchard_plot(mean_rr, mod="Int", xlab = "log(Response ratio) - reference/restored", alpha = 0.1, k=F) +
  scale_y_discrete(labels = "Overall mean") +
  scale_fill_manual(values="purple") +
  scale_colour_manual(values="purple") +
  coord_cartesian(xlim = c(-3, 3))+ theme(legend.position = "none")+
  geom_richtext(x = 2,
           y = 0.7,
           label = paste('<i>N<sub>effect size</sub</i> =', mean_rr$k.all), size =3, label.size  = NA)
  
fig<-p1/p2/p3/p4/p5/p6
fig
```

```
Figure2<-p3/p1/p6/p4+plot_annotation(tag_prefix = "(", tag_levels = "a", tag_suffix = ")")
#ggsave("Figure_2.pdf", Figure2, height = 10, width = 7)
```

```
rr<-(((p3+theme(axis.title = element_blank(),
       panel.grid.major = element_blank(),
       panel.grid.minor = element_blank(),
       axis.text.x = element_blank(),
       axis.text.y = element_text(angle=0)
       )+scale_y_discrete(labels="Relative to \nunrestored")))
 )/
  (p6+theme(
       axis.line.x.top = element_blank(),
       axis.text.y = element_text(angle=0),
       panel.grid.major = element_blank(), panel.grid.minor = element_blank())+xlab("Log response ratio")+scale_y_discrete(labels="Relative to \nreference"))/
(((p1+theme(axis.title = element_blank(),
       panel.grid.major = element_blank(),
       panel.grid.minor = element_blank(),
       axis.text.x = element_blank(),
       axis.text.y = element_text(angle=0),
       )+scale_y_discrete(labels="Relative to \nunrestored")))
 )/
  (p4+theme(
       axis.text.y = element_text(angle=0),
       panel.grid.major = element_blank(), 
       panel.grid.minor = element_blank())+xlab("Log CV ratio")+scale_y_discrete(labels="Relative to \nreference"))

#ggsave("Figure_2.pdf", rr)
```

**Figure S4. An orchard plot showing the meta-analytic mean
(mean effect size) with its 95% confidence interval (thick line) and 95%
prediction interval (thin line), with observed effect sizes based on
various precisions (1/SE).**

```
names<-as.data.frame(c("mean - restored/unrestored", "mean - restored/reference", "cvr - restored/unrestored", "cvr - restored/reference", "vr - restored/unrestored",  "vr - restored/reference"))

names<-`colnames<-`(names, "model")

i2_all<-bind_rows(i2meanur, i2meanrr, i2cvrur, i2cvrrr, i2vrur, i2vrrr)

i2_all<-bind_cols(names, i2_all)

kable(i2_all) %>% kable_styling()%>%
    scroll_box(width = "800px", height = "300px")
```

| model | I2\_total | I2\_id | I2\_plot\_id | I2\_unit |
| --- | --- | --- | --- | --- |
| mean - restored/unrestored | 0.9979406 | 0.5724067 | 0.0162736 | 0.4092603 |
| mean - restored/reference | 0.9994044 | 0.3453757 | 0.0039141 | 0.6501146 |
| cvr - restored/unrestored | 0.6717313 | 0.1461263 | 0.2174874 | 0.3081176 |
| cvr - restored/reference | 0.8014435 | 0.4050007 | 0.0708160 | 0.3256269 |
| vr - restored/unrestored | 0.7712294 | 0.2642332 | 0.1749586 | 0.3320375 |
| vr - restored/reference | 0.7853423 | 0.2785496 | 0.0473188 | 0.4594739 |

**Table S3.** i2 values (measure of heterogeneity among
results) for all models

### Univariate (uni-predictor) analyses

We ran a univariate meta-regression models above for each of the
following moderators: 1) `site_size`, 2)
`age.rest.`, 3) `plu`, to test our research
questions. We also ran the same models with the quadrat size on a
reduced sample of the data (not all sampling methods use sampling method
measurable in area). In no cases did terms change in significance or
direction as a result of the inclusion of quadrat size.

The following code runs plots and tables of the result.

### Age of restoration site

```
a1<-uni_mod_plot_ns(cvr_age_ur, un_re_ag, log_ratio = un_re_ag$yi_cvr, response = un_re_ag$age.rest., variance = un_re_ag$vi_cvr)+ylab("Log CV ratio (relative to unrestored")+xlab("Restored site age (yr)")

a2<-uni_mod_plot_ns(vr_age_ur, un_re_ag, log_ratio = un_re_ag$yi_vr, response = un_re_ag$age.rest., variance = un_re_ag$vi_vr)+ylab("Log SD ratio  (relative to unrestored)")+xlab("Restored site age (yr)")

a3<-uni_mod_plot(mean_age_ur, un_re_ag, log_ratio = un_re_ag$yi_mean, response = un_re_ag$age.rest., variance = un_re_ag$vi_mean)+ylab("Log response ratio (relative to unrestored)")+xlab("Restored site age (yr)")

a4<-uni_mod_plot_ns(cvr_age_rr, re_ref_ag, log_ratio = re_ref_ag$yi_cvr, response = re_ref_ag$age.rest., variance = re_ref_ag$vi_cvr)+ylab("Log CV ratio (relative to reference)")+xlab("Restored site age (yr)")

a5<-uni_mod_plot_ns(vr_age_rr, re_ref_ag, log_ratio = re_ref_ag$yi_vr, response = re_ref_ag$age.rest., variance = re_ref_ag$vi_vr)+ylab("Log SD ratio (relative to referenec)")+xlab("Restored site age (yr)")

a6<-uni_mod_plot_ns(mean_age_rr, re_ref_ag, log_ratio = re_ref_ag$yi_mean, response = re_ref_ag$age.rest., variance = re_ref_ag$vi_mean)+ylab("Log response ratio (relative to reference)")+xlab("Restored site age (yr)")


(a1| a2 | a3) / (a4 | a5 | a6) +plot_annotation(tag_levels = "A")
```

```
fig3<-(a1|a3)/(a4|a6)+plot_annotation(tag_prefix = "(", tag_levels = "a", tag_suffix = ")")
#ggsave("Figure_3.pdf", fig3, height = 8, width = 8)
```

**Figure S5.** The relationship between site age and the
meta-analyitic mean, with its 95% confidence interval (red dashed line)
and 95% prediction interval (blue dashed line), with observed effect
sizes based on various precisions (1/SE).

### Size of restoration site

```
sz1<-uni_mod_plot_ns(cvr_size_ur, un_re_sz, log_ratio = un_re_sz$yi_cvr, response = log(un_re_sz$site_size), variance = un_re_sz$vi_cvr)

sz2<-uni_mod_plot_ns(vr_size_ur, un_re_sz, log_ratio = un_re_sz$yi_vr, response = log(un_re_sz$site_size), variance = un_re_sz$vi_vr)

sz3<-uni_mod_plot_ns(mean_size_ur, un_re_sz, log_ratio = un_re_sz$yi_mean, response = log(un_re_sz$site_size), variance = un_re_sz$vi_mean)

sz4<-uni_mod_plot_ns(cvr_size_rr, re_ref_sz, log_ratio = re_ref_sz$yi_cvr, response = log(re_ref_sz$site_size), variance = re_ref_sz$vi_cvr)
sz5<-uni_mod_plot_ns(vr_size_rr, re_ref_sz, log_ratio = re_ref_sz$yi_vr, response = log(re_ref_sz$site_size), variance = re_ref_sz$vi_vr)
sz6<-uni_mod_plot_ns(mean_size_rr, re_ref_sz, log_ratio = re_ref_sz$yi_mean, response = log(re_ref_sz$site_size), variance = re_ref_sz$vi_mean)

sz_list<-list(sz1,sz2,sz3,sz4,sz5,sz6)

(sz1 + xlab("ln restoration site size (ha)") + ylab("ln(restored / unrestored) [lnCVR]") | sz2 + xlab("ln restoration site size (ha)")+ ylab("ln(restored / unrestored) [lnVR]")| sz3+ xlab("ln restoration site size (ha)")+ ylab("ln(restored / unrestored) [lnRR]") ) / (sz4+ xlab("ln restoration site size (ha)") + ylab("ln(restored / reference) [lnCVR]") | sz5 + xlab("ln restoration site size (ha)") + ylab("ln(restored / unrestored) [lnVR]")| sz6+ xlab("ln restoration site size (ha)")+ ylab("ln(restored / unrestored) [lnRR]")) +plot_annotation(tag_levels = "A")
```

```
Figure_4<-(sz1 + xlab("Restored site size (log ha)")+ ylab("Log CV ratio (relative to unrestored)")|sz4+ xlab("Restored site size (log ha)")+ ylab("Log CV ratio (relative to reference)")) / (sz3+ xlab("Restored site size (log ha)")+ ylab("Log response ratio (relative to unrestored)") | sz6+ xlab("Restored site size (log ha)")+ ylab("Log response ratio (relative to reference")) +plot_annotation(tag_prefix = "(", tag_levels = "a", tag_suffix = ")")


#ggsave("Figure_4.pdf", plot = Figure_4, height = 8, width = 8)
```

**Figure S6.** The relationship between restoration site
size (ha) and the biodiversity change following restoration compared to
unrestored and reference levels.

### Past land use

```
# modify orchard_plot function to not paste K but N instead

orchard_plot<-function (object, mod = "Int", xlab, N = "none", 
    alpha = 0.1, angle = 90, cb = TRUE, k = TRUE, transfm = c("none", 
        "tanh")) 
{
    transfm <- match.arg(transfm)
    if (any(class(object) %in% c("rma.mv", "rma"))) {
        if (mod != "Int") {
            object <- mod_results(object, mod)
        }
        else {
            object <- mod_results(object, mod = "Int")
        }
    }
    mod_table <- object$mod_table
    data <- object$data
    data$moderator <- factor(data$moderator, levels = mod_table$name, 
        labels = mod_table$name)
    data$scale <- (1/sqrt(data[, "vi"]))
    legend <- "Precision (1/SE)"
    if (any(N != "none")) {
        data$scale <- N
        legend <- "Sample Size (N)"
    }
    if (transfm == "tanh") {
        cols <- sapply(mod_table, is.numeric)
        mod_table[, cols] <- Zr_to_r(mod_table[, cols])
        data$yi <- Zr_to_r(data$yi)
        label <- xlab
    }
    else {
        label <- xlab
    }
    mod_table$K <- as.vector(by(data, data[, "moderator"], 
        function(x) length(x[, "yi"])))
    group_no <- nrow(mod_table)
    cbpl <- c("#E69F00", "#009E73", "#F0E442", 
        "#0072B2", "#D55E00", "#CC79A7", "#56B4E9", 
        "#999999")
    plot <- ggplot2::ggplot(data = mod_table, aes(x = estimate, 
        y = name)) + ggbeeswarm::geom_quasirandom(data = data, 
        aes(x = yi, y = moderator, size = scale, colour = moderator), 
        groupOnX = FALSE, alpha = alpha) + ggplot2::geom_errorbarh(aes(xmin = lowerPR, 
        xmax = upperPR), height = 0, show.legend = FALSE, size = 0.5, 
        alpha = 0.6) + ggplot2::geom_errorbarh(aes(xmin = lowerCL, 
        xmax = upperCL), height = 0, show.legend = FALSE, size = 1.2) + 
        ggplot2::geom_vline(xintercept = 0, linetype = 2, colour = "black", 
            alpha = alpha) + ggplot2::geom_point(aes(fill = name), 
        size = 3, shape = 21) + ggplot2::theme_bw() + ggplot2::guides(fill = "none", 
        colour = "none") + ggplot2::theme(legend.position = c(1, 
        0), legend.justification = c(1, 0)) + ggplot2::theme(legend.title = element_text(size = 9)) + 
        ggplot2::theme(legend.direction = "horizontal") + 
        ggplot2::theme(legend.background = element_blank()) + 
        ggplot2::labs(x = label, y = "", size = legend) + 
        ggplot2::theme(axis.text.y = element_text(size = 10, 
            colour = "black", hjust = 0.5, angle = angle))
    if (cb == TRUE) {
        plot <- plot + scale_fill_manual(values = cbpl) + scale_colour_manual(values = cbpl)
    }
    if (k == TRUE) {
        plot <- plot + ggplot2::annotate("text", x = (max(data$yi) + 
            (max(data$yi) * 0.1)), y = (seq(1, group_no, 1) + 
            0.3), label = paste("italic(N)==", mod_table$K), 
            parse = TRUE, hjust = "right", size = 3.5)
    }
    return(plot)
}


pl3<-orchard_plot(mean_plu_ur, mod = "plu", alpha = 0.08, xlab = "lnRR - unrestored/restored")+ theme(legend.position = "none")
pl2<-orchard_plot(vr_plu_ur, mod = "plu", alpha = 0.08,xlab = "lnVR - unrestored/restored")+ theme(legend.position = "none")
pl1<-orchard_plot(cvr_plu_ur, mod = "plu", alpha = 0.08,xlab = "lnCVR - unrestored/restored")+ theme(legend.position = "none")

pl6<-orchard_plot(mean_plu_rr, mod = "plu", alpha = 0.08,xlab = "lnRR - reference/restored")+ theme(legend.position = "none")
pl5<-orchard_plot(vr_plu_rr, mod = "plu", alpha = 0.08,xlab = "lnVR - reference/restored")+ theme(legend.position = "none")
pl4<-orchard_plot(cvr_plu_rr, mod = "plu", alpha = 0.08,xlab = "lnCVR - reference/restored")+ theme(legend.position = "none")


(pl1 | pl2 | pl3) / (pl4 | pl5 | pl6) +plot_annotation(tag_levels = "A")
```

```
pl1<-pl1+ scale_fill_manual(values = rep("green4", 5)) + scale_colour_manual(values = rep("green4", 5))+
       theme(axis.title.y = element_blank(),
       panel.grid.major = element_blank(),
       panel.grid.minor = element_blank(),
       axis.text.y = element_text(angle=0),
       )+xlab("Log CV ratio (relative to unrestored)")

pl3<-pl3+ scale_fill_manual(values = rep("purple", 5)) + scale_colour_manual(values = rep("purple", 5)) +
       theme(axis.title.y = element_blank(),
       panel.grid.major = element_blank(),
       panel.grid.minor = element_blank(),
       axis.text.y = element_text(angle=0),
       )+xlab("Log response ratio (relative to unrestored)")

pl6<-pl6+ scale_fill_manual(values = rep("purple", 5)) + scale_colour_manual(values = rep("purple", 5)) +
       theme(axis.title.y = element_blank(),
       panel.grid.major = element_blank(),
       panel.grid.minor = element_blank(),
       axis.text.y = element_blank(),
       )+xlab("Log response ratio (relative to reference)")

pl4<-pl4+ scale_fill_manual(values = rep("green4", 5)) + scale_colour_manual(values = rep("green4", 5))+
       theme(axis.title.y = element_blank(),
       panel.grid.major = element_blank(),
       panel.grid.minor = element_blank(),
       axis.text.y = element_blank(),
       )+xlab("Log CV ratio (relative to reference)")

Figure5<-((pl3 + pl6) / (pl1 + pl4)) +plot_annotation(tag_prefix = "(", tag_levels = "a", tag_suffix = ")")
#ggsave("Figure_5.pdf", Figure5, height = 8.5, width = 11)
```

**Figure S7** Orchard plots showing the relationship
between restoration site past land use and the biodiversity change
following restoration compared to unrestored and reference levels.

**Table S4.** Effect of age of restoration site on
variability of biodiversity (lnCVR, lnVR) and mean biodiversity (lnRR)
compared to unrestored and reference levels

```
age_list<-list(cvr_age_ur, cvr_age_ur_q, vr_age_ur, vr_age_ur_q, mean_age_ur, mean_age_ur_q, cvr_age_rr, cvr_age_rr_q, vr_age_rr, vr_age_rr_q, mean_age_rr, mean_age_rr_q)
age<-map(.x = age_list, .f = tidy) 
age_res<-bind_rows(age)
names<-c("lnCVR restored/unrestored", "lnCVR restored/unrestored - with quadrat size","lnVR restored/unrestored","lnVR restored/unrestored - with quadrat size","lnRR restored/unrestored","lnRR restored/unrestored - with quadrat size", "lnCVR restored/reference", "lnCVR restored/reference - with quadrat size","lnVR restored/reference","lnVR restored/reference - with quadrat size","lnRR restored/reference","lnRR restored/reference - with quadrat size")
age_res<-age_res %>% mutate(model = rep(names, times = c(2,3,2,3,2,3,2,3,2,3,2,3))) %>% select(model, everything())
kable(age_res) %>% kable_styling()%>%
    scroll_box(width = "800px", height = "300px")
```

| model | term | type | estimate | std.error | statistic | p.value |
| --- | --- | --- | --- | --- | --- | --- |
| lnCVR restored/unrestored | intercept | summary | -0.1068276 | 0.0607740 | -1.7577842 | 0.0787842 |
| lnCVR restored/unrestored | age.rest. | summary | -0.0057333 | 0.0037186 | -1.5417827 | 0.1231264 |
| lnCVR restored/unrestored - with quadrat size | intercept | summary | -0.1588189 | 0.1027788 | -1.5452503 | 0.1222857 |
| lnCVR restored/unrestored - with quadrat size | age.rest. | summary | -0.0069050 | 0.0053638 | -1.2873307 | 0.1979790 |
| lnCVR restored/unrestored - with quadrat size | log(t\_qsize\_m2) | summary | 0.0178760 | 0.0220671 | 0.8100739 | 0.4178977 |
| lnVR restored/unrestored | intercept | summary | 0.0439135 | 0.0636302 | 0.6901364 | 0.4901084 |
| lnVR restored/unrestored | age.rest. | summary | 0.0024611 | 0.0035212 | 0.6989235 | 0.4845999 |
| lnVR restored/unrestored - with quadrat size | intercept | summary | -0.0439626 | 0.1153917 | -0.3809859 | 0.7032137 |
| lnVR restored/unrestored - with quadrat size | age.rest. | summary | 0.0049332 | 0.0048394 | 1.0193958 | 0.3080151 |
| lnVR restored/unrestored - with quadrat size | log(t\_qsize\_m2) | summary | 0.0234839 | 0.0242783 | 0.9672798 | 0.3334042 |
| lnRR restored/unrestored | intercept | summary | 0.1250887 | 0.0383468 | 3.2620400 | 0.0011061 |
| lnRR restored/unrestored | age.rest. | summary | 0.0057740 | 0.0016158 | 3.5735175 | 0.0003522 |
| lnRR restored/unrestored - with quadrat size | intercept | summary | 0.1185138 | 0.0659021 | 1.7983325 | 0.0721243 |
| lnRR restored/unrestored - with quadrat size | age.rest. | summary | 0.0070783 | 0.0022683 | 3.1205543 | 0.0018051 |
| lnRR restored/unrestored - with quadrat size | log(t\_qsize\_m2) | summary | 0.0002807 | 0.0134049 | 0.0209428 | 0.9832913 |
| lnCVR restored/reference | intercept | summary | 0.1609789 | 0.0788196 | 2.0423700 | 0.0411148 |
| lnCVR restored/reference | age.rest. | summary | 0.0015570 | 0.0037609 | 0.4139927 | 0.6788794 |
| lnCVR restored/reference - with quadrat size | intercept | summary | 0.1597338 | 0.1259884 | 1.2678454 | 0.2048532 |
| lnCVR restored/reference - with quadrat size | age.rest. | summary | 0.0038033 | 0.0049450 | 0.7691173 | 0.4418237 |
| lnCVR restored/reference - with quadrat size | log(t\_qsize\_m2) | summary | 0.0082353 | 0.0242982 | 0.3389254 | 0.7346660 |
| lnVR restored/reference | intercept | summary | 0.0067172 | 0.0633476 | 0.1060364 | 0.9155534 |
| lnVR restored/reference | age.rest. | summary | 0.0033310 | 0.0032555 | 1.0231973 | 0.3062146 |
| lnVR restored/reference - with quadrat size | intercept | summary | -0.0341376 | 0.0923360 | -0.3697103 | 0.7115984 |
| lnVR restored/reference - with quadrat size | age.rest. | summary | 0.0042421 | 0.0038553 | 1.1003215 | 0.2711921 |
| lnVR restored/reference - with quadrat size | log(t\_qsize\_m2) | summary | 0.0121269 | 0.0190593 | 0.6362697 | 0.5246007 |
| lnRR restored/reference | intercept | summary | -0.1468596 | 0.0470307 | -3.1226325 | 0.0017924 |
| lnRR restored/reference | age.rest. | summary | 0.0007055 | 0.0022126 | 0.3188355 | 0.7498512 |
| lnRR restored/reference - with quadrat size | intercept | summary | -0.1867170 | 0.0872582 | -2.1398224 | 0.0323691 |
| lnRR restored/reference - with quadrat size | age.rest. | summary | 0.0000736 | 0.0032305 | 0.0227871 | 0.9818201 |
| lnRR restored/reference - with quadrat size | log(t\_qsize\_m2) | summary | 0.0076610 | 0.0172119 | 0.4451001 | 0.6562474 |

**Table S5.** Effect of size (ha) of restoration site on
variability of biodiversity (lnCVR, lnVR) and mean biodiversity (lnRR)
compared to unrestored and reference levels

```
size_list<-list(cvr_size_ur, cvr_size_ur_q, vr_size_ur, vr_size_ur_q, mean_size_ur,mean_size_ur_q, cvr_size_rr,cvr_size_rr_q, vr_size_rr, vr_size_rr_q, mean_size_rr, mean_size_rr_q)

size<-map(.x = size_list, .f = tidy) 
size_res<-bind_rows(size)
names<-c("lnCVR restored/unrestored", "lnCVR restored/unrestored - with quadrat size","lnVR restored/unrestored","lnVR restored/unrestored - with quadrat size","lnRR restored/unrestored","lnRR restored/unrestored - with quadrat size", "lnCVR restored/reference", "lnCVR restored/reference - with quadrat size","lnVR restored/reference","lnVR restored/reference - with quadrat size","lnRR restored/reference","lnRR restored/reference - with quadrat size")
size_res<-size_res %>% mutate(model = rep(names, times = c(2,3,2,3,2,3,2,3,2,3,2,3))) %>% select(model, everything())
kable(size_res) %>% kable_styling()%>%
    scroll_box(width = "800px", height = "300px")
```

| model | term | type | estimate | std.error | statistic | p.value |
| --- | --- | --- | --- | --- | --- | --- |
| lnCVR restored/unrestored | intercept | summary | -0.1650070 | 0.0633310 | -2.6054691 | 0.0091749 |
| lnCVR restored/unrestored | log(site\_size) | summary | 0.0113341 | 0.0169283 | 0.6695349 | 0.5031544 |
| lnCVR restored/unrestored - with quadrat size | intercept | summary | -0.2882260 | 0.1189602 | -2.4228784 | 0.0153981 |
| lnCVR restored/unrestored - with quadrat size | log(site\_size) | summary | -0.0104319 | 0.0234301 | -0.4452365 | 0.6561489 |
| lnCVR restored/unrestored - with quadrat size | log(t\_qsize\_m2) | summary | 0.0400190 | 0.0341938 | 1.1703582 | 0.2418569 |
| lnVR restored/unrestored | intercept | summary | 0.1010521 | 0.0794432 | 1.2720053 | 0.2033712 |
| lnVR restored/unrestored | log(site\_size) | summary | 0.0137065 | 0.0209209 | 0.6551590 | 0.5123654 |
| lnVR restored/unrestored - with quadrat size | intercept | summary | 0.0566133 | 0.1671474 | 0.3387027 | 0.7348337 |
| lnVR restored/unrestored - with quadrat size | log(site\_size) | summary | 0.0012972 | 0.0336589 | 0.0385384 | 0.9692584 |
| lnVR restored/unrestored - with quadrat size | log(t\_qsize\_m2) | summary | 0.0210472 | 0.0442273 | 0.4758864 | 0.6341553 |
| lnRR restored/unrestored | intercept | summary | 0.2615080 | 0.0573297 | 4.5614773 | 0.0000051 |
| lnRR restored/unrestored | log(site\_size) | summary | -0.0128193 | 0.0139094 | -0.9216260 | 0.3567237 |
| lnRR restored/unrestored - with quadrat size | intercept | summary | 0.2142117 | 0.1135340 | 1.8867627 | 0.0591922 |
| lnRR restored/unrestored - with quadrat size | log(site\_size) | summary | -0.0037902 | 0.0236403 | -0.1603288 | 0.8726221 |
| lnRR restored/unrestored - with quadrat size | log(t\_qsize\_m2) | summary | 0.0209292 | 0.0272426 | 0.7682542 | 0.4423362 |
| lnCVR restored/reference | intercept | summary | 0.2444956 | 0.0847242 | 2.8857826 | 0.0039044 |
| lnCVR restored/reference | log(site\_size) | summary | -0.0185232 | 0.0227547 | -0.8140358 | 0.4156244 |
| lnCVR restored/reference - with quadrat size | intercept | summary | 0.2596436 | 0.1239166 | 2.0953098 | 0.0361435 |
| lnCVR restored/reference - with quadrat size | log(site\_size) | summary | -0.0509273 | 0.0274077 | -1.8581370 | 0.0631496 |
| lnCVR restored/reference - with quadrat size | log(t\_qsize\_m2) | summary | -0.0046997 | 0.0303315 | -0.1549444 | 0.8768651 |
| lnVR restored/reference | intercept | summary | 0.1301382 | 0.0778002 | 1.6727220 | 0.0943820 |
| lnVR restored/reference | log(site\_size) | summary | -0.0198852 | 0.0208759 | -0.9525410 | 0.3408227 |
| lnVR restored/reference - with quadrat size | intercept | summary | 0.0750873 | 0.1102572 | 0.6810190 | 0.4958595 |
| lnVR restored/reference - with quadrat size | log(site\_size) | summary | -0.0524892 | 0.0235936 | -2.2247170 | 0.0261002 |
| lnVR restored/reference - with quadrat size | log(t\_qsize\_m2) | summary | 0.0073618 | 0.0272550 | 0.2701095 | 0.7870760 |
| lnRR restored/reference | intercept | summary | -0.1017665 | 0.0490825 | -2.0733749 | 0.0381374 |
| lnRR restored/reference | log(site\_size) | summary | -0.0048247 | 0.0131968 | -0.3655968 | 0.7146660 |
| lnRR restored/reference - with quadrat size | intercept | summary | -0.1773332 | 0.0808711 | -2.1927878 | 0.0283227 |
| lnRR restored/reference - with quadrat size | log(site\_size) | summary | 0.0020029 | 0.0181781 | 0.1101847 | 0.9122629 |
| lnRR restored/reference - with quadrat size | log(t\_qsize\_m2) | summary | 0.0125650 | 0.0193315 | 0.6499744 | 0.5157088 |

**Table S6.** Effect of past land status of restoration
site on variability of biodiversity (lnCVR, lnVR) and mean biodiversity
(lnRR) compared to unrestored and reference levels

```
plu_list<-list(cvr_plu_ur, cvr_plu_ur_q, vr_plu_ur, vr_plu_ur_q, mean_plu_ur, mean_plu_ur_q, cvr_plu_rr, cvr_plu_rr_q, vr_plu_rr, vr_plu_rr_q, mean_plu_rr, mean_plu_rr_q)

plu<-map(.x = plu_list, .f = tidy) 
plu_res<-bind_rows(plu)
names<-c("lnCVR restored/unrestored", "lnCVR restored/unrestored - with quadrat size","lnVR restored/unrestored","lnVR restored/unrestored - with quadrat size","lnRR restored/unrestored","lnRR restored/unrestored - with quadrat size", "lnCVR restored/reference", "lnCVR restored/reference - with quadrat size","lnVR restored/reference","lnVR restored/reference - with quadrat size","lnRR restored/reference","lnRR restored/reference - with quadrat size")
plu_res<-plu_res %>% mutate(model = rep(names, times = c(5,5,5,5,5,5,5,6,5,6,5,6))) #%>% select(model, everything())
kable(plu_res) %>% kable_styling()%>%
    scroll_box(width = "800px", height = "300px")
```

| term | type | estimate | std.error | statistic | p.value | model |
| --- | --- | --- | --- | --- | --- | --- |
| plusemi-natural | summary | -0.3852357 | 0.1077520 | -3.5752081 | 0.0003499 | lnCVR restored/unrestored |
| pluagriculture | summary | -0.0829547 | 0.0710309 | -1.1678664 | 0.2428607 | lnCVR restored/unrestored |
| pluforestry | summary | -0.0846212 | 0.0962468 | -0.8792107 | 0.3792871 | lnCVR restored/unrestored |
| plumining | summary | -0.1584071 | 0.2076465 | -0.7628693 | 0.4455413 | lnCVR restored/unrestored |
| pluurban | summary | -0.2599954 | 0.3680248 | -0.7064615 | 0.4799012 | lnCVR restored/unrestored |
| plusemi-natural | summary | -0.4413136 | 0.1281706 | -3.4431738 | 0.0005749 | lnCVR restored/unrestored - with quadrat size |
| pluagriculture | summary | -0.0853426 | 0.1023533 | -0.8338049 | 0.4043910 | lnCVR restored/unrestored - with quadrat size |
| pluforestry | summary | -0.2638104 | 0.1492127 | -1.7680153 | 0.0770583 | lnCVR restored/unrestored - with quadrat size |
| pluurban | summary | -0.2566981 | 0.3698492 | -0.6940616 | 0.4876436 | lnCVR restored/unrestored - with quadrat size |
| log(t\_qsize\_m2) | summary | 0.0257898 | 0.0214315 | 1.2033554 | 0.2288388 | lnCVR restored/unrestored - with quadrat size |
| plusemi-natural | summary | -0.0082467 | 0.1223165 | -0.0674209 | 0.9462466 | lnVR restored/unrestored |
| pluagriculture | summary | 0.1866603 | 0.0818572 | 2.2803175 | 0.0225889 | lnVR restored/unrestored |
| pluforestry | summary | -0.0103818 | 0.1148719 | -0.0903771 | 0.9279876 | lnVR restored/unrestored |
| plumining | summary | 0.0164567 | 0.2608203 | 0.0630960 | 0.9496901 | lnVR restored/unrestored |
| pluurban | summary | -0.3516609 | 0.4317616 | -0.8144793 | 0.4153704 | lnVR restored/unrestored |
| plusemi-natural | summary | -0.1714823 | 0.1529290 | -1.1213197 | 0.2621518 | lnVR restored/unrestored - with quadrat size |
| pluagriculture | summary | 0.2179067 | 0.1234627 | 1.7649601 | 0.0775705 | lnVR restored/unrestored - with quadrat size |
| pluforestry | summary | -0.2748811 | 0.1837485 | -1.4959634 | 0.1346632 | lnVR restored/unrestored - with quadrat size |
| pluurban | summary | -0.3511681 | 0.4477038 | -0.7843760 | 0.4328195 | lnVR restored/unrestored - with quadrat size |
| log(t\_qsize\_m2) | summary | 0.0386884 | 0.0242723 | 1.5939330 | 0.1109511 | lnVR restored/unrestored - with quadrat size |
| plusemi-natural | summary | 0.2956695 | 0.0731717 | 4.0407623 | 0.0000533 | lnRR restored/unrestored |
| pluagriculture | summary | 0.2083782 | 0.0491856 | 4.2365694 | 0.0000227 | lnRR restored/unrestored |
| pluforestry | summary | 0.0347762 | 0.0721479 | 0.4820126 | 0.6297970 | lnRR restored/unrestored |
| plumining | summary | 0.1678914 | 0.1656187 | 1.0137226 | 0.3107151 | lnRR restored/unrestored |
| pluurban | summary | -0.1310971 | 0.2517307 | -0.5207831 | 0.6025179 | lnRR restored/unrestored |
| plusemi-natural | summary | 0.2476480 | 0.0950021 | 2.6067642 | 0.0091402 | lnRR restored/unrestored - with quadrat size |
| pluagriculture | summary | 0.2500708 | 0.0774378 | 3.2293127 | 0.0012409 | lnRR restored/unrestored - with quadrat size |
| pluforestry | summary | 0.0075252 | 0.1153611 | 0.0652314 | 0.9479897 | lnRR restored/unrestored - with quadrat size |
| pluurban | summary | -0.1326478 | 0.2781277 | -0.4769313 | 0.6334111 | lnRR restored/unrestored - with quadrat size |
| log(t\_qsize\_m2) | summary | 0.0043270 | 0.0131356 | 0.3294128 | 0.7418437 | lnRR restored/unrestored - with quadrat size |
| plusemi-natural | summary | -0.0228100 | 0.1941139 | -0.1175084 | 0.9064572 | lnCVR restored/reference |
| pluagriculture | summary | 0.1314184 | 0.0966701 | 1.3594519 | 0.1740034 | lnCVR restored/reference |
| pluforestry | summary | 0.3612461 | 0.1335275 | 2.7054073 | 0.0068221 | lnCVR restored/reference |
| plumining | summary | 0.3291036 | 0.2081652 | 1.5809728 | 0.1138842 | lnCVR restored/reference |
| pluurban | summary | -0.1934379 | 0.3967512 | -0.4875547 | 0.6258653 | lnCVR restored/reference |
| plusemi-natural | summary | -0.1258523 | 0.2673475 | -0.4707444 | 0.6378233 | lnCVR restored/reference - with quadrat size |
| pluagriculture | summary | 0.1967008 | 0.1357583 | 1.4489044 | 0.1473643 | lnCVR restored/reference - with quadrat size |
| pluforestry | summary | 0.5183067 | 0.1940891 | 2.6704574 | 0.0075748 | lnCVR restored/reference - with quadrat size |
| plumining | summary | 0.6026451 | 0.2945680 | 2.0458606 | 0.0407701 | lnCVR restored/reference - with quadrat size |
| pluurban | summary | -0.1859859 | 0.3922212 | -0.4741861 | 0.6353672 | lnCVR restored/reference - with quadrat size |
| log(t\_qsize\_m2) | summary | -0.0079093 | 0.0255702 | -0.3093172 | 0.7570802 | lnCVR restored/reference - with quadrat size |
| plusemi-natural | summary | 0.0094039 | 0.1580410 | 0.0595030 | 0.9525515 | lnVR restored/reference |
| pluagriculture | summary | 0.0177378 | 0.0771091 | 0.2300352 | 0.8180644 | lnVR restored/reference |
| pluforestry | summary | 0.1835670 | 0.1047408 | 1.7525826 | 0.0796737 | lnVR restored/reference |
| plumining | summary | -0.0451151 | 0.1646990 | -0.2739244 | 0.7841427 | lnVR restored/reference |
| pluurban | summary | -0.2390748 | 0.3301516 | -0.7241365 | 0.4689820 | lnVR restored/reference |
| plusemi-natural | summary | -0.0718948 | 0.2012747 | -0.3571972 | 0.7209442 | lnVR restored/reference - with quadrat size |
| pluagriculture | summary | 0.0124196 | 0.1016058 | 0.1222331 | 0.9027145 | lnVR restored/reference - with quadrat size |
| pluforestry | summary | 0.2325126 | 0.1421050 | 1.6362030 | 0.1017971 | lnVR restored/reference - with quadrat size |
| plumining | summary | -0.0041445 | 0.2082891 | -0.0198978 | 0.9841249 | lnVR restored/reference - with quadrat size |
| pluurban | summary | -0.2336051 | 0.3075150 | -0.7596541 | 0.4474614 | lnVR restored/reference - with quadrat size |
| log(t\_qsize\_m2) | summary | 0.0023398 | 0.0205270 | 0.1139867 | 0.9092483 | lnVR restored/reference - with quadrat size |
| plusemi-natural | summary | 0.0653162 | 0.1166493 | 0.5599365 | 0.5755228 | lnRR restored/reference |
| pluagriculture | summary | -0.1127956 | 0.0568177 | -1.9852179 | 0.0471202 | lnRR restored/reference |
| pluforestry | summary | -0.1936367 | 0.0788148 | -2.4568577 | 0.0140158 | lnRR restored/reference |
| plumining | summary | -0.3526318 | 0.1192666 | -2.9566677 | 0.0031098 | lnRR restored/reference |
| pluurban | summary | -0.1252452 | 0.2358817 | -0.5309661 | 0.5954423 | lnRR restored/reference |
| plusemi-natural | summary | 0.0948855 | 0.1823099 | 0.5204629 | 0.6027410 | lnRR restored/reference - with quadrat size |
| pluagriculture | summary | -0.1563267 | 0.0942632 | -1.6584061 | 0.0972355 | lnRR restored/reference - with quadrat size |
| pluforestry | summary | -0.2794606 | 0.1363858 | -2.0490451 | 0.0404577 | lnRR restored/reference - with quadrat size |
| plumining | summary | -0.5663590 | 0.2020657 | -2.8028461 | 0.0050654 | lnRR restored/reference - with quadrat size |
| pluurban | summary | -0.1392828 | 0.2674524 | -0.5207760 | 0.6025228 | lnRR restored/reference - with quadrat size |
| log(t\_qsize\_m2) | summary | 0.0100630 | 0.0176291 | 0.5708176 | 0.5681233 | lnRR restored/reference - with quadrat size |

## Publication bias

We used multiple approaches to assess the effect of publication bias
on our results. We used a multilevel version of Egger’s regression with
the square-root of the sampling variances as a moderator to test for
asymmetry in funnel plots of meta-analytic models (Nakagawa & Poulin
2012). We also used traditional funnel plots to visually assess
asymmetry. Intercepts in Egger’s regressions were different from zero in
all models, providing evidence for publication bias. We added the
publication year to meta-regressions to test for the effect of a
time-lag bias on our results (Appendix S2, Table S4-S7). The effect of
publication year was not related to effect sizes in any model (Appendix
S2, Table S4-S7).

```
############################################################
###AND majority of code lifted from from Johnson et al 2020 \####
###Silicon is a global plant defence but effectiveness###### 
###depends on herbivore feeding strategy: a meta-analysis"##


library(metafor)
library(kableExtra)
library(knitr)
library(ggplot2)

#univariate egger regressoin

egger_uni_ur_cvr <- rma.mv(yi = yi_cvr, V = vi_cvr, mods = ~sqrt(vi_cvr), test = "t", random = list(~1 | id, ~1 | plot_id, ~1 | unit), method = "REML", data = un_re)
egger_uni_ur_vr <- rma.mv(yi = yi_vr, V = vi_vr, mods = ~sqrt(vi_vr), test = "t", random = list(~1 | id, ~1 | plot_id, ~1 | unit), method = "REML", data = un_re)
egger_uni_ur_mean <- rma.mv(yi = yi_mean, V = vi_mean, mods = ~sqrt(vi_mean), test = "t", random = list(~1 | id, ~1 | plot_id, ~1 | unit), method = "REML", data = un_re)
egger_uni_rr_cvr <- rma.mv(yi = yi_cvr, V = vi_cvr, mods = ~sqrt(vi_cvr), test = "t", random = list(~1 | id, ~1 | plot_id, ~1 | unit), method = "REML", data = re_ref)
egger_uni_rr_vr <- rma.mv(yi = yi_vr, V = vi_vr, mods = ~sqrt(vi_vr), test = "t", random = list(~1 | id, ~1 | plot_id, ~1 | unit), method = "REML", data = re_ref)
egger_uni_rr_mean <- rma.mv(yi = yi_mean, V = vi_mean, mods = ~sqrt(vi_mean), test = "t", random = list(~1 | id, ~1 | plot_id, ~1 | unit), method = "REML", data = re_ref)
```

### Traditional funnel plots

```
###Publication bias analysis - unscaled models
# can't get this to print neatly, for now? something to do with metafor::funnel ? nvm this works:
par(mfrow=c(3,2)) 

funnel(egger_uni_ur_cvr, yaxis = "seinv", level = c(90, 95, 99), shade = c("white", "gray55", "gray75"), refline = 0, legend = TRUE)
funnel(egger_uni_ur_vr, yaxis = "seinv", level = c(90, 95, 99), shade = c("white", "gray55", "gray75"), refline = 0, legend = TRUE)
funnel(egger_uni_ur_mean, yaxis = "seinv", level = c(90, 95, 99), shade = c("white", "gray55", "gray75"), refline = 0, legend = TRUE)
funnel(egger_uni_rr_cvr, yaxis = "seinv", level = c(90, 95, 99), shade = c("white", "gray55", "gray75"), refline = 0, legend = TRUE)
funnel(egger_uni_rr_vr, yaxis = "seinv", level = c(90, 95, 99), shade = c("white", "gray55", "gray75"), refline = 0, legend = TRUE)
funnel(egger_uni_rr_mean, yaxis = "seinv", level = c(90, 95, 99), shade = c("white", "gray55", "gray75"), refline = 0, legend = TRUE)
```

**Figure S8** Funnel plots testing for asymmetry for all
models and effect sizes. A = restored/unrestored lnCVR, B =
restored/unrestored lnVR, C = restored/unrestored lnRR, D =
restored/reference lnCVR, E = restored/reference lnVR, F =
restored/reference lnRR

### Plot of univariate egger regressions

```
uni1<-uni_egger_plot_cvr(egger_uni_ur_cvr, data = un_re)+ylab("lnCVR (effect size")
uni2<-uni_egger_plot_vr(egger_uni_ur_vr, data = un_re)+ylab("lnVR (effect size")
uni3<-uni_egger_plot_mean(egger_uni_ur_mean, data = un_re)
uni4<-uni_egger_plot_cvr(egger_uni_rr_cvr, data = re_ref)+ylab("lnCVR (effect size")
uni5<-uni_egger_plot_vr(egger_uni_rr_vr, data = re_ref)+ylab("lnVR (effect size")
uni6<-uni_egger_plot_mean(egger_uni_rr_mean, data = re_ref)

(uni1 + uni2 + uni3) / (uni4 + uni5 + uni6) + plot_annotation(tag_levels = "A")
```

**Figure S9.** Plot of univariate egger regression for
all models and effect sizes

## Time-lag bias

**Table S7.** Relationship between publication year and
effect size (lnRR - restored/unrestored)

```
##################

time_lag_effect_uni_lnRR <- rma.mv(yi = yi_mean, V = vcv_mean, mods = ~Year, test = "t", 
                                   random = list(~1 | id, ~1 | shared_ctrl, ~1 | unit), method = "REML", data = un_re)
# getting marginal R2
r2_time_lag_effect_uni_lnRR <- r2_ml(time_lag_effect_uni_lnRR)
# getting estimates: name does not work for slopes
res_time_lag_effect_uni_lnRR <- get_est(time_lag_effect_uni_lnRR, mod = "Year")
# creating a table
tibble(`Fixed effect` = row.names(time_lag_effect_uni_lnRR$beta), Estimate = c(res_time_lag_effect_uni_lnRR$estimate), 
       `Lower CI [0.025]` = c(res_time_lag_effect_uni_lnRR$lowerCL), `Upper CI  [0.975]` = c(res_time_lag_effect_uni_lnRR$upperCL), 
       `P value` = time_lag_effect_uni_lnRR$pval, R2 = c(r2_time_lag_effect_uni_lnRR[1], 
                                                         NA)) %>% kable("html", digits = 3) %>% kable_styling("striped", position = "left")
```

| Fixed effect | Estimate | Lower CI [0.025] | Upper CI [0.975] | P value | R2 |
| --- | --- | --- | --- | --- | --- |
| intrcpt | -7.803 | -45.842 | 30.236 | 0.687 | 0.002 |
| Year | 0.004 | -0.015 | 0.023 | 0.680 | NA |

**Table S8.** Relationship between publication year and
effect size (lnCVR - restored/unrestored)

```
##################

time_lag_effect_uni_lnCVR <- rma.mv(yi = yi_cvr, V = vcv_cvr, mods = ~Year, test = "t", 
                                   random = list(~1 | id, ~1 | shared_ctrl, ~1 | unit), method = "REML", data = un_re)
# getting marginal R2
r2_time_lag_effect_uni_lnCVR <- r2_ml(time_lag_effect_uni_lnCVR)
# getting estimates: name does not work for slopes
res_time_lag_effect_uni_lnCVR <- get_est(time_lag_effect_uni_lnCVR, mod = "Year")
# creating a table
tibble(`Fixed effect` = row.names(time_lag_effect_uni_lnCVR$beta), Estimate = c(res_time_lag_effect_uni_lnCVR$estimate), 
       `Lower CI [0.025]` = c(res_time_lag_effect_uni_lnCVR$lowerCL), `Upper CI  [0.975]` = c(res_time_lag_effect_uni_lnCVR$upperCL), 
       `P value` = time_lag_effect_uni_lnCVR$pval, R2 = c(r2_time_lag_effect_uni_lnCVR[1], 
                                                         NA)) %>% kable("html", digits = 3) %>% kable_styling("striped", position = "left")
```

| Fixed effect | Estimate | Lower CI [0.025] | Upper CI [0.975] | P value | R2 |
| --- | --- | --- | --- | --- | --- |
| intrcpt | 9.285 | -40.984 | 59.555 | 0.717 | 0.001 |
| Year | -0.005 | -0.030 | 0.020 | 0.713 | NA |

**Table S9.** Relationship between publication year and
effect size (lnRR - restored/reference)

```
##################

time_lag_effect_uni_lnRRreref <- rma.mv(yi = yi_mean, V = vcv_mean_rr, mods = ~Year, test = "t", 
                                   random = list(~1 | id, ~1 | shared_ctrl, ~1 | unit), method = "REML", data = re_ref)
# getting marginal R2
r2_time_lag_effect_uni_lnRRreref <- r2_ml(time_lag_effect_uni_lnRRreref)
# getting estimates: name does not work for slopes
res_time_lag_effect_uni_lnRRreref <- get_est(time_lag_effect_uni_lnRRreref, mod = "Year")
# creating a table
tibble(`Fixed effect` = row.names(time_lag_effect_uni_lnRRreref$beta), Estimate = c(res_time_lag_effect_uni_lnRRreref$estimate), 
       `Lower CI [0.025]` = c(res_time_lag_effect_uni_lnRRreref$lowerCL), `Upper CI  [0.975]` = c(res_time_lag_effect_uni_lnRRreref$upperCL), 
       `P value` = time_lag_effect_uni_lnRRreref$pval, R2 = c(r2_time_lag_effect_uni_lnRRreref[1], 
                                                         NA)) %>% kable("html", digits = 3) %>% kable_styling("striped", position = "left")
```

| Fixed effect | Estimate | Lower CI [0.025] | Upper CI [0.975] | P value | R2 |
| --- | --- | --- | --- | --- | --- |
| intrcpt | 32.946 | -5.642 | 71.535 | 0.094 | 0.015 |
| Year | -0.016 | -0.036 | 0.003 | 0.093 | NA |

**Table S10.** Relationship between publication year and
effect size (lnRR - restored/reference)

```
##################

time_lag_effect_uni_lnCVRreref <- rma.mv(yi = yi_cvr, V = vcv_cvr_rr, mods = ~Year, test = "t", 
                                   random = list(~1 | id, ~1 | shared_ctrl, ~1 | unit), method = "REML", data = re_ref)
# getting marginal R2
r2_time_lag_effect_uni_lnRRreref <- r2_ml(time_lag_effect_uni_lnRRreref)
# getting estimates: name does not work for slopes
res_time_lag_effect_uni_lnRRreref <- get_est(time_lag_effect_uni_lnRRreref, mod = "Year")
# creating a table
tibble(`Fixed effect` = row.names(time_lag_effect_uni_lnRRreref$beta), Estimate = c(res_time_lag_effect_uni_lnRRreref$estimate), 
       `Lower CI [0.025]` = c(res_time_lag_effect_uni_lnRRreref$lowerCL), `Upper CI  [0.975]` = c(res_time_lag_effect_uni_lnRRreref$upperCL), 
       `P value` = time_lag_effect_uni_lnRRreref$pval, R2 = c(r2_time_lag_effect_uni_lnRRreref[1], 
                                                         NA)) %>% kable("html", digits = 3) %>% kable_styling("striped", position = "left")
```

| Fixed effect | Estimate | Lower CI [0.025] | Upper CI [0.975] | P value | R2 |
| --- | --- | --- | --- | --- | --- |
| intrcpt | 32.946 | -5.642 | 71.535 | 0.094 | 0.015 |
| Year | -0.016 | -0.036 | 0.003 | 0.093 | NA |

## Scale dependency

We follow recommendations by Spake et al. (2020) where possible to
reduce the scale bias in our meta-analyses. Firstly, we use the log
response ratio which has been shown to be more robust in scenarios of
cross-study syntheses at varying spatial grain, compared with Hedges’ g
(Spake et al. 2020) . Our data does not allow the use of asymptotic
measures of richness (e.g. rarefied richness) since only a very small
minority of studies report biodiversity in this manner. However, we run
all models including the quadrat size as a term in all our models and
compare to the results without this term. The significance or
non-significance of all results reported were not changed when models
were run on the reduced dataset models including quadrat sizes where
available. Though, the nature of many biodiversity sampling methods
(e.g. pitfall traps, butterfly nets, transects) are not comparable in m2
meaning that these diagnostic models exclude 20-30% of the data points.
Therefore, the model results presented in the main text are excluding
quadrat size.

The plots below show that our effect sizes would have been sensitive
to size had we used Hedges *g*, however they show little response
to scale when using the log response ratio or log CV ratio.

```
dug <- read.csv("Data/variation_data.csv")#put data file here

duglr <- metafor::escalc(measure="ROM",m1i=dug$t_mean, m2i=dug$c_mean, sd1i=dug$t_sd, sd2i=c_sd, n1i=dug$t_quad_n, n2i=dug$c_quad_n, append=T, data=dug) #non-equal variances 

#lnCVR
dugcvr <- metafor::escalc(measure="CVR",m1i=dug$t_mean, m2i=dug$c_mean, sd1i=dug$t_sd, sd2i=c_sd, n1i=dug$t_quad_n, n2i=dug$c_quad_n, append=T, data=dug) 

#Hedges' g
dughg <- metafor::escalc(measure="SMD",m1i=dug$t_mean, m2i=dug$c_mean, sd1i=dug$t_sd, sd2i=c_sd, n1i=dug$t_quad_n, n2i=dug$c_quad_n, append=T, data=dug) 

#alternative variance estimate for g
n1=as.numeric(dug$t_quad_n); n2=as.numeric(dug$c_quad_n)
n_tilde=n2*n1/(n2+n1)
var_d_n.DENS=((1-3/(4*(n2+n1-2)-1))^2)*(n2+n1-2)/(n_tilde*(n2+n1-4)) #Hedges variance that does not contain d https://esajournals.onlinelibrary.wiley.com/doi/full/10.1002/ecs2.2419

dughg$vi2<- var_d_n.DENS

unit <- factor(1:length(duglr$yi))
duglr$unit <- unit

unit <- factor(1:length(dugcvr$yi))
dugcvr$unit <- unit

unit <- factor(1:length(dughg$yi))
dughg$unit <- unit

duglr<-duglr %>% drop_na(c(id, plot_id, unit))
dugcvr<-duglr %>% drop_na(c(id, plot_id, unit))
dughg<-dughg %>% drop_na(c(id, plot_id, unit))

duglr<-duglr %>% drop_na(t_qsize_m2)
dugcvr<-duglr %>% drop_na(t_qsize_m2)
dughg<-dughg %>% drop_na(t_qsize_m2)

#random-effects, conventional weighted meta-analysis
lr.ma.ran <- rma.mv(yi=yi, V=vi, data=duglr, method="REML", random = list(~1 | id, ~1 | plot_id, ~1 | unit))
#unweighted 
lr.ma.un <- rma.mv(yi=yi, V=vi, data=duglr, method="REML", random= list(~1 | id, ~1 | plot_id, ~1 | unit), W=1)
#fixed-effects, conventional weighted meta-analysis
lr.ma.fix <- rma.mv(yi=yi, V=vi, data=duglr, method="REML", random= list(~1 | id, ~1 | plot_id, ~1 | unit), W = 1/vi)

#random-effects, conventional weighted meta-analysis
cvr.ma.ran <- rma.mv(yi=yi, V=vi, data=dugcvr, method="REML", random = list(~1 | id, ~1 | plot_id, ~1 | unit))
#unweighted 
cvr.ma.un <- rma.mv(yi=yi, V=vi, data=dugcvr, method="REML", random= list(~1 | id, ~1 | plot_id, ~1 | unit), W=1)
#fixed-effects, conventional weighted meta-analysis
cvr.ma.fix <- rma.mv(yi=yi, V=vi, data=dugcvr, method="REML", random= list(~1 | id, ~1 | plot_id, ~1 | unit), W = 1/vi)

#random-effects, conventional weighted meta-analysis
hg.ma.ran <- rma.mv(yi=yi, V=vi, data=dughg, method="REML", random= list(~1 | id, ~1 | plot_id, ~1 | unit))
#unweighted 
hg.ma.un <- rma.mv(yi=yi, V=vi, data=dughg, method="REML", random= list(~1 | id, ~1 | plot_id, ~1 | unit), W=1)
#fixed-effects, conventional weighted meta-analysis
hg.ma.fix <- rma.mv(yi=yi, V=vi, data=dughg, method="REML", random= list(~1 | id, ~1 | plot_id, ~1 | unit), W = 1/vi)

hg.ma.ran.d_alt <- rma.mv(yi=yi, V=vi2, data=dughg, method="REML", random= list(~1 | id, ~1 | plot_id, ~1 | unit))

hg.ma.fix.dalt <- rma.mv(yi=yi, V=vi2, data=dughg, method="REML", random= list(~1 | id, ~1 | plot_id, ~1 | unit), W = 1/vi2)
```

### Exploratory bubble plots

```
duglr<-duglr %>% mutate(id = as.factor(id)) # currently thinks id is numeric - is that an issue?
dugcvr<-dugcvr %>% mutate(id = as.factor(id)) # currently thinks id is numeric - is that an issue?
dughg<-dughg %>% mutate(id = as.factor(id)) # currently thinks id is numeric - is that an issue?

NvsA.LR <- ggplot(duglr) + geom_point(aes(x=log(t_qsize_m2/10000), y=t_quad_n, size=1/vi,col=id,alpha=0.3))+ theme_bw() + theme(panel.grid.major = element_blank(),
panel.grid.minor = element_blank(), axis.line = element_line(colour = "black"))+ ylab(expression(italic(N)))+xlab("Log(Plot size (ha))")+geom_hline(yintercept = 0, linetype = "dashed") + scale_size_continuous(name = expression(paste("1/",italic(V))),labels=NULL)+scale_color_discrete("", guide=F)+scale_alpha("",guide=F)+
    theme(plot.title = element_text(size = 8))+theme(axis.text.x=element_text(size=rel(0.7)))

NvsA.CVR <- ggplot(dugcvr) + geom_point(aes(x=log(t_qsize_m2/10000), y=t_quad_n, size=1/vi,col=id,alpha=0.3))+ theme_bw() + theme(panel.grid.major = element_blank(),
panel.grid.minor = element_blank(), axis.line = element_line(colour = "black"))+ ylab(expression(italic(N)))+xlab("Log(Plot size (ha))")+geom_hline(yintercept = 0, linetype = "dashed") + scale_size_continuous(name = expression(paste("1/",italic(V))),labels=NULL)+scale_color_discrete("", guide=F)+scale_alpha("",guide=F)+
    theme(plot.title = element_text(size = 8))+theme(axis.text.x=element_text(size=rel(0.7)))

NvsA.g <- ggplot(dughg) + geom_point(aes(x=log(t_qsize_m2/10000), y=t_quad_n, size=1/vi, color=id,alpha=0.3))+ theme_bw() + theme(panel.grid.major = element_blank(),
panel.grid.minor = element_blank(), axis.line = element_line(colour = "black"))+ ylab(expression(italic(N)))+xlab("Log(Plot size (ha))")+geom_hline(yintercept = 0, linetype = "dashed") + scale_size_continuous(name = expression(paste("1/",italic(V))),labels=NULL)+scale_color_discrete("", guide=F)+scale_alpha("",guide=F)+ 
    theme(plot.title = element_text(size = 8))+theme(axis.text.x=element_text(size=rel(0.7)))


LRvsA <- ggplot(duglr) + geom_point(aes(x=log(t_qsize_m2/10000), y=yi, size=1/vi, color=id,alpha=0.3))+ theme_bw() + theme(panel.grid.major = element_blank(),
panel.grid.minor = element_blank(), axis.line = element_line(colour = "black"))+ ylab(expression(italic("LR")))+xlab("Log(Plot size (ha))")+geom_hline(yintercept = 0, linetype = "dashed") + scale_size_continuous(name = expression(paste("1/",italic(V))),labels=NULL)+scale_color_discrete("study")+scale_color_discrete("", guide=F)+scale_alpha("",guide=F)+ 
    theme(plot.title = element_text(size = 8))+theme(axis.text.x=element_text(size=rel(0.7)))

CVRvsA <- ggplot(dugcvr) + geom_point(aes(x=log(t_qsize_m2/10000), y=yi, size=1/vi, color=id,alpha=0.3))+ theme_bw() + theme(panel.grid.major = element_blank(),
panel.grid.minor = element_blank(), axis.line = element_line(colour = "black"))+ ylab(expression(italic("CVR")))+xlab("Log(Plot size (ha))")+geom_hline(yintercept = 0, linetype = "dashed") + scale_size_continuous(name = expression(paste("1/",italic(V))),labels=NULL)+scale_color_discrete("study")+scale_color_discrete("", guide=F)+scale_alpha("",guide=F)+ 
    theme(plot.title = element_text(size = 8))+theme(axis.text.x=element_text(size=rel(0.7)))


gvsA <-  ggplot(dughg) + geom_point(aes(x=log(t_qsize_m2/10000), y=yi, size=1/vi, color=id,alpha=0.3))+ theme_bw() + theme(panel.grid.major = element_blank(),
panel.grid.minor = element_blank(), axis.line = element_line(colour = "black"))+ ylab(expression(italic(g)))+xlab("Log(Plot size (ha))")+geom_hline(yintercept = 0, linetype = "dashed") +scale_size_continuous(name = expression(paste("1/",italic(V))),labels=NULL)+scale_color_discrete("", guide=F)+scale_alpha("",guide=F)+ 
    theme(plot.title = element_text(size = 8))+theme(axis.text.x=element_text(size=rel(0.7)))


LR.VarvsA <- ggplot(duglr) + geom_point(aes(x=log(t_qsize_m2/10000), y=vi, size=t_quad_n, color=id,alpha=0.3))+ theme_bw() + theme(panel.grid.major = element_blank(),
panel.grid.minor = element_blank(), axis.line = element_line(colour = "black"))+ ylab(expression(paste("Variance of ",italic(LR))))+xlab("Log(Plot size (ha))")+geom_hline(yintercept = 0, linetype = "dashed") +scale_size_continuous(name = expression(italic(N)),labels=NULL)+scale_color_discrete("", guide=F)+scale_alpha("",guide=F)+ 
    theme(plot.title = element_text(size = 8))+theme(axis.text.x=element_text(size=rel(0.7)))

CVR.VarvsA <- ggplot(dugcvr) + geom_point(aes(x=log(t_qsize_m2/10000), y=vi, size=t_quad_n, color=id,alpha=0.3))+ theme_bw() + theme(panel.grid.major = element_blank(),
panel.grid.minor = element_blank(), axis.line = element_line(colour = "black"))+ ylab(expression(paste("Variance of ",italic(CVR))))+xlab("Log(Plot size (ha))")+geom_hline(yintercept = 0, linetype = "dashed") +scale_size_continuous(name = expression(italic(N)),labels=NULL)+scale_color_discrete("", guide=F)+scale_alpha("",guide=F)+ 
    theme(plot.title = element_text(size = 8))+theme(axis.text.x=element_text(size=rel(0.7)))

g.VarvsA <- ggplot(dughg) + geom_point(aes(x=log(t_qsize_m2/10000), y=vi, size=t_quad_n, color=id,alpha=0.3))+ theme_bw() + theme(panel.grid.major = element_blank(),
panel.grid.minor = element_blank(), axis.line = element_line(colour = "black"))+ ylab(expression(paste("Variance of ",italic(g))))+xlab("Log(Plot size (ha))")+geom_hline(yintercept = 0, linetype = "dashed") +scale_size_continuous(name = expression(italic(N)),labels=NULL)+scale_color_discrete("", guide=F)+scale_alpha("",guide=F)+ 
    theme(plot.title = element_text(size = 8))+theme(axis.text=element_text(size=rel(0.7)))

library(gridExtra)

gridExtra::grid.arrange(NvsA.g, gvsA, g.VarvsA,NvsA.LR,  LRvsA,LR.VarvsA, NvsA.CVR,  CVRvsA,CVR.VarvsA, ncol=3)
```

**Figure S10 Bubble plot of plot size against various effect
sizes (labelled)**

```
hdeff <- data.frame(estimate=c(hg.ma.ran$b,hg.ma.un$b,hg.ma.fix$b,hg.ma.ran.d_alt$b,hg.ma.fix.dalt$b),ci.up=c(hg.ma.ran$ci.ub,hg.ma.un$ci.ub,hg.ma.fix$ci.ub, hg.ma.ran.d_alt$ci.ub,hg.ma.fix.dalt$ci.ub), ci.lo=c(hg.ma.ran$ci.lb,hg.ma.un$ci.lb,hg.ma.fix$ci.lb,hg.ma.ran.d_alt$ci.lb,hg.ma.fix.dalt$ci.lb), weighting=c("R", "U", "F", "R", "F"), vtype=c("d","d","d","d_alt","d_alt"))

hdeff <- hdeff %>%
arrange(weighting) %>%    # First sort by val. This sort the dataframe but NOT the factor levels
  mutate(weighting=factor(weighting, levels=
                            c("R", "F", "U")))    # This trick update the factor levels

hdeff.p.a <-ggplot(hdeff) + geom_point(aes(y = estimate, x = weighting, colour=vtype), position=position_dodge(width = 0.5)) + geom_errorbar(aes(x=weighting,ymin=ci.lo, ymax=ci.up, width = 0.1, colour=vtype),, position=position_dodge(width = 0.5))+
   geom_abline(intercept = 0, slope=0, colour = "darkgray", linetype="dashed",) +
  xlab(NULL) +
  ylab(expression(italic(g)))+
  theme_bw() + 
  theme(plot.title = element_text(size=10),axis.title = element_text(size=10),axis.text = element_text(size=9) )
hdeff.p.a <-hdeff.p.a +theme(axis.text.x = element_text(angle=0,face = c(rep('plain',16), 'bold', 'bold'))) +scale_color_manual(values=c("black", "darkgray"))

lreff <- data.frame(estimate=c(lr.ma.ran$b,lr.ma.un$b,lr.ma.fix$b),ci.up=c(lr.ma.ran$ci.ub,lr.ma.un$ci.ub,lr.ma.fix$ci.ub), ci.lo=c(lr.ma.ran$ci.lb,lr.ma.un$ci.lb,lr.ma.fix$ci.lb), weighting=c("R", "U", "F"))
lreff <- lreff %>%
arrange(weighting) %>%    # First sort by val. This sort the dataframe but NOT the factor levels
  mutate(weighting=factor(weighting, levels=
                            c("R", "F", "U")))    # This trick update the factor levels


lreff.p.a <-ggplot(lreff) + geom_point(aes(y = estimate, x = weighting)) + geom_errorbar(aes(x=weighting,ymin=ci.lo, ymax=ci.up, width = 0.1))+
  geom_abline(intercept = 0, slope=0, colour = "darkgray", linetype="dashed") +
  xlab(NULL) +
 ylab(expression(italic(LR)))+
  theme_bw() + 
  theme(plot.title = element_text(size=10),axis.title = element_text(size=10),axis.text = element_text(size=9) )
lreff.p.a <-lreff.p.a +theme(axis.text.x = element_text(angle=0,face = c(rep('plain',16), 'bold', 'bold')))

cvreff <- data.frame(estimate=c(cvr.ma.ran$b,cvr.ma.un$b,cvr.ma.fix$b),ci.up=c(cvr.ma.ran$ci.ub,cvr.ma.un$ci.ub,cvr.ma.fix$ci.ub), ci.lo=c(cvr.ma.ran$ci.lb,cvr.ma.un$ci.lb,cvr.ma.fix$ci.lb), weighting=c("R", "U", "F"))
cvreff <- cvreff %>%
arrange(weighting) %>%    # First sort by val. This sort the dataframe but NOT the factor levels
  mutate(weighting=factor(weighting, levels=
                            c("R", "F", "U")))    # This trick update the factor levels


cvreff.p.a <-ggplot(cvreff) + geom_point(aes(y = estimate, x = weighting)) + geom_errorbar(aes(x=weighting,ymin=ci.lo, ymax=ci.up, width = 0.1))+
  geom_abline(intercept = 0, slope=0, colour = "darkgray", linetype="dashed") +
  xlab(NULL) +
 ylab(expression(italic(cvr)))+
  theme_bw() + 
  theme(plot.title = element_text(size=10),axis.title = element_text(size=10),axis.text = element_text(size=9) )
cvreff.p.a <-cvreff.p.a +theme(axis.text.x = element_text(angle=0,face = c(rep('plain',16), 'bold', 'bold')))


gridExtra::grid.arrange(hdeff.p.a, lreff.p.a, cvreff.p.a, ncol=3, widths = c(3, 2.3, 2.3))
```

```
#LR
#random, conventionally weighted
lr.ma.ran <- rma.mv(yi=scale(yi), V=vi, mods=log(t_qsize_m2), data=duglr, method="REML", random= list(~1 | id, ~1 | plot_id, ~1 | unit))
# unweighted 
lr.ma.un <- rma.mv(yi=scale(yi), V=vi, mods=log(t_qsize_m2), data=duglr, method="REML", random= list(~1 | id, ~1 | plot_id, ~1 | unit), W=1)
#fixed effect 
lr.ma.fix <- rma.mv(yi=scale(yi), V=vi, mods=log(t_qsize_m2), data=duglr, method="REML", random= list(~1 | id, ~1 | plot_id, ~1 | unit), W = 1/vi)


#cvr
#random, conventionally weighted
cvr.ma.ran <- rma.mv(yi=scale(yi), V=vi, mods=log(t_qsize_m2), data=dugcvr, method="REML", random= list(~1 | id, ~1 | plot_id, ~1 | unit))
# unweighted 
cvr.ma.un <- rma.mv(yi=scale(yi), V=vi, mods=log(t_qsize_m2), data=dugcvr, method="REML", random= list(~1 | id, ~1 | plot_id, ~1 | unit), W=1)
#fixed effect 
cvr.ma.fix <- rma.mv(yi=scale(yi), V=vi, mods=log(t_qsize_m2), data=dugcvr, method="REML", random= list(~1 | id, ~1 | plot_id, ~1 | unit), W = 1/vi)

#HG
#random, conventionally weighted
hg.ma.ran <- rma.mv(yi=scale(yi), V=vi, mods=log(t_qsize_m2), data=dughg, method="REML", random= list(~1 | id, ~1 | plot_id, ~1 | unit))
# unweighted 
hg.ma.un <- rma.mv(yi=scale(yi), V=vi, mods=log(t_qsize_m2), data=dughg, method="REML", random= list(~1 | id, ~1 | plot_id, ~1 | unit), W=1)
#fixed effects 
hg.ma.fix <- rma.mv(yi=scale(yi), V=vi, mods=log(t_qsize_m2), data=dughg, method="REML", random= list(~1 | id, ~1 | plot_id, ~1 | unit), W = 1/vi)

hg.ma.ran.d_alt <- rma.mv(yi=scale(yi), V=vi2, mods=log(t_qsize_m2), data=dughg, method="REML", random= list(~1 | id, ~1 | plot_id, ~1 | unit))

hg.ma.fix.dalt <- rma.mv(yi=scale(yi), V=vi2, mods=log(t_qsize_m2), data=dughg, method="REML", random= list(~1 | id, ~1 | plot_id, ~1 | unit), W = 1/vi2)
```

```
hdeff <- data.frame(estimate=c(hg.ma.ran$b[2],hg.ma.un$b[2],hg.ma.fix$b[2],hg.ma.ran.d_alt$b[2],hg.ma.fix.dalt$b[2]),ci.up=c(hg.ma.ran$ci.ub[2],hg.ma.un$ci.ub[2],hg.ma.fix$ci.ub[2], hg.ma.ran.d_alt$ci.ub[2],hg.ma.fix.dalt$ci.ub[2]), ci.lo=c(hg.ma.ran$ci.lb[2],hg.ma.un$ci.lb[2],hg.ma.fix$ci.lb[2],hg.ma.ran.d_alt$ci.lb[2],hg.ma.fix.dalt$ci.lb[2]), weighting=c("R", "U", "F", "R", "F"), vtype=c("d","d","d","d_alt","d_alt"))

hdeff <- hdeff %>%
arrange(weighting) %>%    # First sort by val. This sort the dataframe but NOT the factor levels
  mutate(weighting=factor(weighting, levels=
                            c("R", "F", "U")))    # This trick update the factor levels

hdeff.p.a <-ggplot(hdeff) + geom_point(aes(y = estimate, x = weighting, colour=vtype), position=position_dodge(width = 0.5)) + geom_errorbar(aes(x=weighting,ymin=ci.lo, ymax=ci.up, width = 0.1, colour=vtype),, position=position_dodge(width = 0.5))+
   geom_abline(intercept = 0, slope=0, colour = "darkgray", linetype="dashed",) +
  xlab(NULL) +
  ylab("Effect of plot size (regression coefficient)")+
  theme_bw() + 
  theme(plot.title = element_text(size=10),axis.title = element_text(size=10),axis.text = element_text(size=9) )
hdeff.p.a <-hdeff.p.a +theme(axis.text.x = element_text(angle=0,face = c(rep('plain',16), 'bold', 'bold')))+ylim(-1,1.2) +scale_color_manual(values=c("black", "darkgray"), guide=F)

lreff <- data.frame(estimate=c(lr.ma.ran$b[2],lr.ma.un$b[2],lr.ma.fix$b[2]),ci.up=c(lr.ma.ran$ci.ub[2],lr.ma.un$ci.ub[2],lr.ma.fix$ci.ub[2]), ci.lo=c(lr.ma.ran$ci.lb[2],lr.ma.un$ci.lb[2],lr.ma.fix$ci.lb[2]), weighting=c("R", "U", "F"))
lreff <- lreff %>%
arrange(weighting) %>%    # First sort by val. This sort the dataframe but NOT the factor levels
  mutate(weighting=factor(weighting, levels=
                            c("R", "F", "U")))    # This trick update the factor levels


lreff.p.a <-ggplot(lreff) + geom_point(aes(y = estimate, x = weighting)) + geom_errorbar(aes(x=weighting,ymin=ci.lo, ymax=ci.up, width = 0.1))+
  geom_abline(intercept = 0, slope=0, colour = "darkgray", linetype="dashed") +
  xlab(NULL) +
  ylab("Effect of plot size (regression coefficient)")+
  theme_bw() + 
  theme(plot.title = element_text(size=10),axis.title = element_text(size=10),axis.text = element_text(size=9) )
lreff.p.a <-lreff.p.a +theme(axis.text.x = element_text(angle=0,face = c(rep('plain',16), 'bold', 'bold')))+ylim(-1.2,1.5)


cvreff <- data.frame(estimate=c(cvr.ma.ran$b[2],cvr.ma.un$b[2],cvr.ma.fix$b[2]),ci.up=c(cvr.ma.ran$ci.ub[2],cvr.ma.un$ci.ub[2],cvr.ma.fix$ci.ub[2]), ci.lo=c(cvr.ma.ran$ci.lb[2],cvr.ma.un$ci.lb[2],cvr.ma.fix$ci.lb[2]), weighting=c("R", "U", "F"))
cvreff <- cvreff %>%
arrange(weighting) %>%    # First sort by val. This sort the dataframe but NOT the factor levels
  mutate(weighting=factor(weighting, levels=
                            c("R", "F", "U")))    # This trick update the factor levels


cvreff.p.a <-ggplot(cvreff) + geom_point(aes(y = estimate, x = weighting)) + geom_errorbar(aes(x=weighting,ymin=ci.lo, ymax=ci.up, width = 0.1))+
  geom_abline(intercept = 0, slope=0, colour = "darkgray", linetype="dashed") +
  xlab(NULL) +
  ylab("Effect of plot size (regression coefficient)")+
  theme_bw() + 
  theme(plot.title = element_text(size=10),axis.title = element_text(size=10),axis.text = element_text(size=9) )
cvreff.p.a <-cvreff.p.a +theme(axis.text.x = element_text(angle=0,face = c(rep('plain',16), 'bold', 'bold')))+ylim(-1.2,1.5)

gridExtra::grid.arrange(hdeff.p.a, lreff.p.a, cvreff.p.a, ncol=3, widths=c(3,2.3, 2.3))
```

Re-run meta-analyses, but on unscaled response variables so can make
predictions of unscaled effect sizes.

```
hg.ma.ran <- rma.mv(yi=yi, V=vi, mods=log(t_qsize_m2), data=dughg, method="REML", random= list(~1 | id, ~1 | plot_id, ~1 | unit))
#random unweighted (same meta-est if fixed unweighted, but diff se)
hg.ma.un <- rma.mv(yi=yi, V=vi, mods=log(t_qsize_m2), data=dughg, method="REML", random= list(~1 | id, ~1 | plot_id, ~1 | unit), W=1)
#random effect but control the weights, do 1/v. same est as a fixed effect
hg.ma.fix <- rma.mv(yi=yi, V=vi, mods=log(t_qsize_m2), data=dughg, method="REML", random= list(~1 | id, ~1 | plot_id, ~1 | unit), W = 1/vi)

hg.ma.ran.d_alt <- rma.mv(yi=yi, V=vi2, mods=log(t_qsize_m2), data=dughg, method="REML", random= list(~1 | id, ~1 | plot_id, ~1 | unit))

hg.ma.fix.dalt <- rma.mv(yi=yi, V=vi2, mods=log(t_qsize_m2), data=dughg, method="REML", random= list(~1 | id, ~1 | plot_id, ~1 | unit), W = 1/vi2)


lr.ma.ran <- rma.mv(yi=yi, V=vi, mods=log(t_qsize_m2), data=duglr, method="REML", random= list(~1 | id, ~1 | plot_id, ~1 | unit))
#random unweighted (same meta-est if fixed unweighted, but diff se)
lr.ma.un <- rma.mv(yi=yi, V=vi, mods=log(t_qsize_m2), data=duglr, method="REML", random= list(~1 | id, ~1 | plot_id, ~1 | unit), W=1)
#random effect but control the weights, do 1/v. same est as a fixed effect
lr.ma.fix <- rma.mv(yi=yi, V=vi, mods=log(t_qsize_m2), data=duglr, method="REML", random= list(~1 | id, ~1 | plot_id, ~1 | unit), W = 1/vi)


cvr.ma.ran <- rma.mv(yi=yi, V=vi, mods=log(t_qsize_m2), data=dugcvr, method="REML", random= list(~1 | id, ~1 | plot_id, ~1 | unit))
#random unweighted (same meta-est if fixed unweighted, but diff se)
cvr.ma.un <- rma.mv(yi=yi, V=vi, mods=log(t_qsize_m2), data=dugcvr, method="REML", random= list(~1 | id, ~1 | plot_id, ~1 | unit), W=1)
#random effect but control the weights, do 1/v. same est as a fixed effect
cvr.ma.fix <- rma.mv(yi=yi, V=vi, mods=log(t_qsize_m2), data=dugcvr, method="REML", random= list(~1 | id, ~1 | plot_id, ~1 | unit), W = 1/vi)
```

Now predict the effect sizes across all interpolated values of A,
plot the meta-regression slopes:

```
newmods=data.frame(intercept=hg.ma.ran$b[1], t_qsize_m2=seq(min(dughg$t_qsize_m2), max(dughg$t_qsize_m2), 0.1) )
newmods=as.matrix(newmods)
#head(newmods)
hg.ma.ran.preds=data.frame(predict(hg.ma.ran,  addx=TRUE))
hg.ma.fix.preds=data.frame(predict(hg.ma.fix,  addx=TRUE))
hg.ma.un.preds=data.frame(predict(hg.ma.un,  addx=TRUE))
#weights(hg.ma.ran)
hg.ma.ran.p=ggplot()+geom_point(data=dughg,aes(x=log(t_qsize_m2), y=yi,colour=id), 
                                               #size=weights(hg.ma.ran)), 
                                alpha=0.3) +geom_line(aes(x=hg.ma.ran.preds$X.mods, y=hg.ma.ran.preds$pred))+geom_line(aes(x=hg.ma.ran.preds$X.mods, y=hg.ma.ran.preds$pred))+geom_ribbon(aes(x=hg.ma.ran.preds$X.mods,ymin=hg.ma.ran.preds$ci.lb, ymax=hg.ma.ran.preds$ci.ub ),alpha=0.2)+scale_size_continuous(guide=FALSE)+scale_color_discrete(guide=F)+theme_bw()+ylab(expression(italic(g)))+xlab(NULL)+geom_hline(yintercept = 0, linetype = "dashed") +ggtitle(expression(paste("Random-effects meta-analysis, wt = 1/(", italic("V"),"+",tau^2,")")))+theme(plot.title = element_text(size = 8))+ylim(-12,7.2)


hg.ma.fix.p=ggplot()+geom_point(data=dughg,aes(x=log(t_qsize_m2), y=yi,colour=id), #size=weights(hg.ma.fix)),
                                               alpha=0.3) +geom_line(aes(x=hg.ma.fix.preds$X.mods, y=hg.ma.fix.preds$pred))+geom_line(aes(x=hg.ma.fix.preds$X.mods, y=hg.ma.fix.preds$pred))+geom_ribbon(aes(x=hg.ma.fix.preds$X.mods,ymin=hg.ma.fix.preds$ci.lb, ymax=hg.ma.fix.preds$ci.ub ),alpha=0.2)+scale_size_continuous(guide=FALSE)+scale_color_discrete(guide=F)+theme_bw()+ylab(expression(italic(g)))+xlab(NULL)+geom_hline(yintercept = 0, linetype = "dashed")+ggtitle(expression(paste("Fixed-effects meta-analysis, wt = 1/(", italic("V"),")")))+theme(plot.title = element_text(size = 8))+ylim(-12,7.2)

hg.ma.un.p=ggplot()+geom_point(data=dughg,aes(x=log(t_qsize_m2), y=yi,colour=id, size=1), alpha=0.3) +geom_line(aes(x=hg.ma.fix.preds$X.mods, y=hg.ma.un.preds$pred))+geom_line(aes(x=hg.ma.un.preds$X.mods, y=hg.ma.un.preds$pred))+geom_ribbon(aes(x=hg.ma.un.preds$X.mods,ymin=hg.ma.un.preds$ci.lb, ymax=hg.ma.un.preds$ci.ub ),alpha=0.2)+scale_color_discrete(guide=F)+theme_bw()+ylab(expression(italic(g)))+xlab("Log(Plot size (ha))")+geom_hline(yintercept = 0, linetype = "dashed")+scale_size_continuous(guide=FALSE)+ggtitle("Unweighted meta-analysis, wt = 1")+theme(plot.title = element_text(size = 8))+ylim(-12,7.2)


newmods=data.frame(intercept=lr.ma.ran$b[1], t_qsize_m2=seq(min(duglr$t_qsize_m2), max(duglr$t_qsize_m2), 0.1) )
newmods=as.matrix(newmods)
#head(newmods)
lr.ma.ran.preds=data.frame(predict(lr.ma.ran,  addx=TRUE))
lr.ma.fix.preds=data.frame(predict(lr.ma.fix,  addx=TRUE))
lr.ma.un.preds=data.frame(predict(lr.ma.un,  addx=TRUE))

lr.ma.ran.p=ggplot()+geom_point(data=duglr,aes(x=log(t_qsize_m2), y=yi,colour=id),# size=weights(lr.ma.ran)),
                                alpha=0.3) +geom_line(aes(x=lr.ma.ran.preds$X.mods, y=lr.ma.ran.preds$pred))+geom_line(aes(x=lr.ma.ran.preds$X.mods, y=lr.ma.ran.preds$pred))+geom_ribbon(aes(x=lr.ma.ran.preds$X.mods,ymin=lr.ma.ran.preds$ci.lb, ymax=lr.ma.ran.preds$ci.ub ),alpha=0.2)+scale_size_continuous(guide=FALSE)+scale_color_discrete(guide=F)+theme_bw()+ylab(expression(italic(LR)))+xlab(NULL)+geom_hline(yintercept = 0, linetype = "dashed") +ggtitle("")+theme(plot.title = element_text(size = 8))+ylim(-1.6,1.2)


lr.ma.fix.p=ggplot()+geom_point(data=duglr,aes(x=log(t_qsize_m2), y=yi,colour=id),# size=weights(lr.ma.fix)),
                                alpha=0.3) +geom_line(aes(x=lr.ma.fix.preds$X.mods, y=lr.ma.fix.preds$pred))+geom_line(aes(x=lr.ma.fix.preds$X.mods, y=lr.ma.fix.preds$pred))+geom_ribbon(aes(x=lr.ma.fix.preds$X.mods,ymin=lr.ma.fix.preds$ci.lb, ymax=lr.ma.fix.preds$ci.ub ),alpha=0.2)+scale_size_continuous(guide=FALSE)+scale_color_discrete(guide=F)+theme_bw()+ylab(expression(italic(LR)))+xlab(NULL)+geom_hline(yintercept = 0, linetype = "dashed")+ggtitle("")+theme(plot.title = element_text(size = 8))+coord_cartesian(ylim = c(-1.6, 1.2)) 


lr.ma.un.p=ggplot()+geom_point(data=duglr,aes(x=log(t_qsize_m2), y=yi,colour=id, size=1), alpha=0.3) +geom_line(aes(x=lr.ma.fix.preds$X.mods, y=lr.ma.un.preds$pred))+geom_line(aes(x=lr.ma.un.preds$X.mods, y=lr.ma.un.preds$pred))+geom_ribbon(aes(x=lr.ma.un.preds$X.mods,ymin=lr.ma.un.preds$ci.lb, ymax=lr.ma.un.preds$ci.ub ),alpha=0.2)+scale_color_discrete(guide=F)+theme_bw()+ylab(expression(italic(LR)))+xlab("Log(Plot size (ha))")+geom_hline(yintercept = 0, linetype = "dashed")+scale_size_continuous(guide=FALSE)+ggtitle("")+theme(plot.title = element_text(size = 8))+ylim(-1.6, 1.2)

newmods=data.frame(intercept=cvr.ma.ran$b[1], t_qsize_m2=seq(min(dugcvr$t_qsize_m2), max(dugcvr$t_qsize_m2), 0.1) )
newmods=as.matrix(newmods)
#head(newmods)
cvr.ma.ran.preds=data.frame(predict(cvr.ma.ran,  addx=TRUE))
cvr.ma.fix.preds=data.frame(predict(cvr.ma.fix,  addx=TRUE))
cvr.ma.un.preds=data.frame(predict(cvr.ma.un,  addx=TRUE))

cvr.ma.ran.p=ggplot()+geom_point(data=dugcvr,aes(x=log(t_qsize_m2), y=yi,colour=id),# size=weights(cvr.ma.ran)),
                                alpha=0.3) +geom_line(aes(x=cvr.ma.ran.preds$X.mods, y=cvr.ma.ran.preds$pred))+geom_line(aes(x=cvr.ma.ran.preds$X.mods, y=cvr.ma.ran.preds$pred))+geom_ribbon(aes(x=cvr.ma.ran.preds$X.mods,ymin=cvr.ma.ran.preds$ci.lb, ymax=cvr.ma.ran.preds$ci.ub ),alpha=0.2)+scale_size_continuous(guide=FALSE)+scale_color_discrete(guide=F)+theme_bw()+ylab(expression(italic(cvr)))+xlab(NULL)+geom_hline(yintercept = 0, linetype = "dashed") +ggtitle("")+theme(plot.title = element_text(size = 8))+ylim(-1.6,1.2)


cvr.ma.fix.p=ggplot()+geom_point(data=dugcvr,aes(x=log(t_qsize_m2), y=yi,colour=id),# size=weights(cvr.ma.fix)),
                                alpha=0.3) +geom_line(aes(x=cvr.ma.fix.preds$X.mods, y=cvr.ma.fix.preds$pred))+geom_line(aes(x=cvr.ma.fix.preds$X.mods, y=cvr.ma.fix.preds$pred))+geom_ribbon(aes(x=cvr.ma.fix.preds$X.mods,ymin=cvr.ma.fix.preds$ci.lb, ymax=cvr.ma.fix.preds$ci.ub ),alpha=0.2)+scale_size_continuous(guide=FALSE)+scale_color_discrete(guide=F)+theme_bw()+ylab(expression(italic(cvr)))+xlab(NULL)+geom_hline(yintercept = 0, linetype = "dashed")+ggtitle("")+theme(plot.title = element_text(size = 8))+coord_cartesian(ylim = c(-1.6, 1.2)) 


cvr.ma.un.p=ggplot()+geom_point(data=dugcvr,aes(x=log(t_qsize_m2), y=yi,colour=id, size=1), alpha=0.3) +geom_line(aes(x=cvr.ma.fix.preds$X.mods, y=cvr.ma.un.preds$pred))+geom_line(aes(x=cvr.ma.un.preds$X.mods, y=cvr.ma.un.preds$pred))+geom_ribbon(aes(x=cvr.ma.un.preds$X.mods,ymin=cvr.ma.un.preds$ci.lb, ymax=cvr.ma.un.preds$ci.ub ),alpha=0.2)+scale_color_discrete(guide=F)+theme_bw()+ylab(expression(italic(cvr)))+xlab("Log(Plot size (ha))")+geom_hline(yintercept = 0, linetype = "dashed")+scale_size_continuous(guide=FALSE)+ggtitle("")+theme(plot.title = element_text(size = 8))+ylim(-1.6, 1.2)


gridExtra::grid.arrange(hg.ma.ran.p, lr.ma.ran.p,cvr.ma.ran.p,
                        hg.ma.fix.p, lr.ma.fix.p,cvr.ma.fix.p,
                        hg.ma.un.p, lr.ma.un.p,cvr.ma.fix.p,
                        ncol=3, heights=c(2,2,2.2))#+ylim(-3.2, 0.7)
```

**Figure S11. Meta-regression slopes of models using hedges g,
lnRR, and lnCVR against log(plot size). Each meta-regression is
conducted as a random-effect, fixed-effects, and unweighted
meta-analysis**

This size-bias testing has only been conducted for the
restored/unrestored comparison. The chunk below will re-run the same
tests on the restored/reference comparison, however these plots will not
be presented as they do not deviate significantly from the results just
presented

```
dug <- read.csv("Data/variation_data.csv")#put data file here
dug<-dug %>% filter(r_quad_n >= 1)
duglr <- metafor::escalc(measure="ROM",m1i=dug$t_mean, m2i=dug$r_mean, sd1i=dug$t_sd, sd2i=r_sd, n1i=dug$t_quad_n, n2i=dug$r_quad_n, append=T, data=dug) #non-equal variances 

#lnCVR
dugcvr <- metafor::escalc(measure="CVR",m1i=dug$t_mean, m2i=dug$r_mean, sd1i=dug$t_sd, sd2i=r_sd, n1i=dug$t_quad_n, n2i=dug$r_quad_n, append=T, data=dug) 

#Hedges' g
dughg <- metafor::escalc(measure="SMD",m1i=dug$t_mean, m2i=dug$r_mean, sd1i=dug$t_sd, sd2i=r_sd, n1i=dug$t_quad_n, n2i=dug$r_quad_n, append=T, data=dug) 

#alternative variance estimate for g
n1=as.numeric(dug$t_quad_n); n2=as.numeric(dug$c_quad_n)
n_tilde=n2*n1/(n2+n1)
var_d_n.DENS=((1-3/(4*(n2+n1-2)-1))^2)*(n2+n1-2)/(n_tilde*(n2+n1-4)) #Hedges variance that does not contain d https://esajournals.onlinelibrary.wiley.com/doi/full/10.1002/ecs2.2419

dughg$vi2<- var_d_n.DENS
dughg<-dughg %>% filter(vi2 > 0 ) # some SD = 0 mucking up the calcs below?

unit <- factor(1:length(duglr$yi))
duglr$unit <- unit

unit <- factor(1:length(dugcvr$yi))
dugcvr$unit <- unit

unit <- factor(1:length(dughg$yi))
dughg$unit <- unit

duglr<-duglr %>% drop_na(c(id, plot_id, unit))
dugcvr<-duglr %>% drop_na(c(id, plot_id, unit))
dughg<-dughg %>% drop_na(c(id, plot_id, unit))

duglr<-duglr %>% drop_na(t_qsize_m2)
dugcvr<-duglr %>% drop_na(t_qsize_m2)
dughg<-dughg %>% drop_na(t_qsize_m2)

#random-effects, conventional weighted meta-analysis
lr.ma.ran <- rma.mv(yi=yi, V=vi, data=duglr, method="REML", random = list(~1 | id, ~1 | plot_id, ~1 | unit))
#unweighted 
lr.ma.un <- rma.mv(yi=yi, V=vi, data=duglr, method="REML", random= list(~1 | id, ~1 | plot_id, ~1 | unit), W=1)
#fixed-effects, conventional weighted meta-analysis
lr.ma.fix <- rma.mv(yi=yi, V=vi, data=duglr, method="REML", random= list(~1 | id, ~1 | plot_id, ~1 | unit), W = 1/vi)

#random-effects, conventional weighted meta-analysis
cvr.ma.ran <- rma.mv(yi=yi, V=vi, data=dugcvr, method="REML", random = list(~1 | id, ~1 | plot_id, ~1 | unit))
#unweighted 
cvr.ma.un <- rma.mv(yi=yi, V=vi, data=dugcvr, method="REML", random= list(~1 | id, ~1 | plot_id, ~1 | unit), W=1)
#fixed-effects, conventional weighted meta-analysis
cvr.ma.fix <- rma.mv(yi=yi, V=vi, data=dugcvr, method="REML", random= list(~1 | id, ~1 | plot_id, ~1 | unit), W = 1/vi)

#random-effects, conventional weighted meta-analysis
hg.ma.ran <- rma.mv(yi=yi, V=vi, data=dughg, method="REML", random= list(~1 | id, ~1 | plot_id, ~1 | unit))
#unweighted 
hg.ma.un <- rma.mv(yi=yi, V=vi, data=dughg, method="REML", random= list(~1 | id, ~1 | plot_id, ~1 | unit), W=1)
#fixed-effects, conventional weighted meta-analysis
hg.ma.fix <- rma.mv(yi=yi, V=vi, data=dughg, method="REML", random= list(~1 | id, ~1 | plot_id, ~1 | unit), W = 1/vi)

hg.ma.ran.d_alt <- rma.mv(yi=yi, V=vi2, data=dughg, method="REML", random= list(~1 | id, ~1 | plot_id, ~1 | unit))

hg.ma.fix.dalt <- rma.mv(yi=yi, V=vi2, data=dughg, method="REML", random= list(~1 | id, ~1 | plot_id, ~1 | unit), W = 1/vi2)


duglr<-duglr %>% mutate(id = as.factor(id)) # currently thinks id is numeric - is that an issue?
dugcvr<-dugcvr %>% mutate(id = as.factor(id)) # currently thinks id is numeric - is that an issue?
dughg<-dughg %>% mutate(id = as.factor(id)) # currently thinks id is numeric - is that an issue?

NvsA.LR <- ggplot(duglr) + geom_point(aes(x=log(t_qsize_m2/10000), y=t_quad_n, size=1/vi,col=id,alpha=0.3))+ theme_bw() + theme(panel.grid.major = element_blank(),
panel.grid.minor = element_blank(), axis.line = element_line(colour = "black"))+ ylab(expression(italic(N)))+xlab("Log(Plot size (ha))")+geom_hline(yintercept = 0, linetype = "dashed") + scale_size_continuous(name = expression(paste("1/",italic(V))),labels=NULL)+scale_color_discrete("", guide=F)+scale_alpha("",guide=F)+
    theme(plot.title = element_text(size = 8))+theme(axis.text.x=element_text(size=rel(0.7)))

NvsA.CVR <- ggplot(dugcvr) + geom_point(aes(x=log(t_qsize_m2/10000), y=t_quad_n, size=1/vi,col=id,alpha=0.3))+ theme_bw() + theme(panel.grid.major = element_blank(),
panel.grid.minor = element_blank(), axis.line = element_line(colour = "black"))+ ylab(expression(italic(N)))+xlab("Log(Plot size (ha))")+geom_hline(yintercept = 0, linetype = "dashed") + scale_size_continuous(name = expression(paste("1/",italic(V))),labels=NULL)+scale_color_discrete("", guide=F)+scale_alpha("",guide=F)+
    theme(plot.title = element_text(size = 8))+theme(axis.text.x=element_text(size=rel(0.7)))

NvsA.g <- ggplot(dughg) + geom_point(aes(x=log(t_qsize_m2/10000), y=t_quad_n, size=1/vi, color=id,alpha=0.3))+ theme_bw() + theme(panel.grid.major = element_blank(),
panel.grid.minor = element_blank(), axis.line = element_line(colour = "black"))+ ylab(expression(italic(N)))+xlab("Log(Plot size (ha))")+geom_hline(yintercept = 0, linetype = "dashed") + scale_size_continuous(name = expression(paste("1/",italic(V))),labels=NULL)+scale_color_discrete("", guide=F)+scale_alpha("",guide=F)+ 
    theme(plot.title = element_text(size = 8))+theme(axis.text.x=element_text(size=rel(0.7)))


LRvsA <- ggplot(duglr) + geom_point(aes(x=log(t_qsize_m2/10000), y=yi, size=1/vi, color=id,alpha=0.3))+ theme_bw() + theme(panel.grid.major = element_blank(),
panel.grid.minor = element_blank(), axis.line = element_line(colour = "black"))+ ylab(expression(italic("LR")))+xlab("Log(Plot size (ha))")+geom_hline(yintercept = 0, linetype = "dashed") + scale_size_continuous(name = expression(paste("1/",italic(V))),labels=NULL)+scale_color_discrete("study")+scale_color_discrete("", guide=F)+scale_alpha("",guide=F)+ 
    theme(plot.title = element_text(size = 8))+theme(axis.text.x=element_text(size=rel(0.7)))

CVRvsA <- ggplot(dugcvr) + geom_point(aes(x=log(t_qsize_m2/10000), y=yi, size=1/vi, color=id,alpha=0.3))+ theme_bw() + theme(panel.grid.major = element_blank(),
panel.grid.minor = element_blank(), axis.line = element_line(colour = "black"))+ ylab(expression(italic("CVR")))+xlab("Log(Plot size (ha))")+geom_hline(yintercept = 0, linetype = "dashed") + scale_size_continuous(name = expression(paste("1/",italic(V))),labels=NULL)+scale_color_discrete("study")+scale_color_discrete("", guide=F)+scale_alpha("",guide=F)+ 
    theme(plot.title = element_text(size = 8))+theme(axis.text.x=element_text(size=rel(0.7)))


gvsA <-  ggplot(dughg) + geom_point(aes(x=log(t_qsize_m2/10000), y=yi, size=1/vi, color=id,alpha=0.3))+ theme_bw() + theme(panel.grid.major = element_blank(),
panel.grid.minor = element_blank(), axis.line = element_line(colour = "black"))+ ylab(expression(italic(g)))+xlab("Log(Plot size (ha))")+geom_hline(yintercept = 0, linetype = "dashed") +scale_size_continuous(name = expression(paste("1/",italic(V))),labels=NULL)+scale_color_discrete("", guide=F)+scale_alpha("",guide=F)+ 
    theme(plot.title = element_text(size = 8))+theme(axis.text.x=element_text(size=rel(0.7)))


LR.VarvsA <- ggplot(duglr) + geom_point(aes(x=log(t_qsize_m2/10000), y=vi, size=t_quad_n, color=id,alpha=0.3))+ theme_bw() + theme(panel.grid.major = element_blank(),
panel.grid.minor = element_blank(), axis.line = element_line(colour = "black"))+ ylab(expression(paste("Variance of ",italic(LR))))+xlab("Log(Plot size (ha))")+geom_hline(yintercept = 0, linetype = "dashed") +scale_size_continuous(name = expression(italic(N)),labels=NULL)+scale_color_discrete("", guide=F)+scale_alpha("",guide=F)+ 
    theme(plot.title = element_text(size = 8))+theme(axis.text.x=element_text(size=rel(0.7)))

CVR.VarvsA <- ggplot(dugcvr) + geom_point(aes(x=log(t_qsize_m2/10000), y=vi, size=t_quad_n, color=id,alpha=0.3))+ theme_bw() + theme(panel.grid.major = element_blank(),
panel.grid.minor = element_blank(), axis.line = element_line(colour = "black"))+ ylab(expression(paste("Variance of ",italic(CVR))))+xlab("Log(Plot size (ha))")+geom_hline(yintercept = 0, linetype = "dashed") +scale_size_continuous(name = expression(italic(N)),labels=NULL)+scale_color_discrete("", guide=F)+scale_alpha("",guide=F)+ 
    theme(plot.title = element_text(size = 8))+theme(axis.text.x=element_text(size=rel(0.7)))

g.VarvsA <- ggplot(dughg) + geom_point(aes(x=log(t_qsize_m2/10000), y=vi, size=t_quad_n, color=id,alpha=0.3))+ theme_bw() + theme(panel.grid.major = element_blank(),
panel.grid.minor = element_blank(), axis.line = element_line(colour = "black"))+ ylab(expression(paste("Variance of ",italic(g))))+xlab("Log(Plot size (ha))")+geom_hline(yintercept = 0, linetype = "dashed") +scale_size_continuous(name = expression(italic(N)),labels=NULL)+scale_color_discrete("", guide=F)+scale_alpha("",guide=F)+ 
    theme(plot.title = element_text(size = 8))+theme(axis.text=element_text(size=rel(0.7)))


gridExtra::grid.arrange(NvsA.g, gvsA, g.VarvsA,NvsA.LR,  LRvsA,LR.VarvsA, NvsA.CVR,  CVRvsA,CVR.VarvsA, ncol=3)


hdeff <- data.frame(estimate=c(hg.ma.ran$b,hg.ma.un$b,hg.ma.fix$b,hg.ma.ran.d_alt$b,hg.ma.fix.dalt$b),ci.up=c(hg.ma.ran$ci.ub,hg.ma.un$ci.ub,hg.ma.fix$ci.ub, hg.ma.ran.d_alt$ci.ub,hg.ma.fix.dalt$ci.ub), ci.lo=c(hg.ma.ran$ci.lb,hg.ma.un$ci.lb,hg.ma.fix$ci.lb,hg.ma.ran.d_alt$ci.lb,hg.ma.fix.dalt$ci.lb), weighting=c("R", "U", "F", "R", "F"), vtype=c("d","d","d","d_alt","d_alt"))

hdeff <- hdeff %>%
arrange(weighting) %>%    # First sort by val. This sort the dataframe but NOT the factor levels
  mutate(weighting=factor(weighting, levels=
                            c("R", "F", "U")))    # This trick update the factor levels

hdeff.p.a <-ggplot(hdeff) + geom_point(aes(y = estimate, x = weighting, colour=vtype), position=position_dodge(width = 0.5)) + geom_errorbar(aes(x=weighting,ymin=ci.lo, ymax=ci.up, width = 0.1, colour=vtype),, position=position_dodge(width = 0.5))+
   geom_abline(intercept = 0, slope=0, colour = "darkgray", linetype="dashed",) +
  xlab(NULL) +
  ylab(expression(italic(g)))+
  theme_bw() + 
  theme(plot.title = element_text(size=10),axis.title = element_text(size=10),axis.text = element_text(size=9) )
hdeff.p.a <-hdeff.p.a +theme(axis.text.x = element_text(angle=0,face = c(rep('plain',16), 'bold', 'bold'))) +scale_color_manual(values=c("black", "darkgray"))

lreff <- data.frame(estimate=c(lr.ma.ran$b,lr.ma.un$b,lr.ma.fix$b),ci.up=c(lr.ma.ran$ci.ub,lr.ma.un$ci.ub,lr.ma.fix$ci.ub), ci.lo=c(lr.ma.ran$ci.lb,lr.ma.un$ci.lb,lr.ma.fix$ci.lb), weighting=c("R", "U", "F"))
lreff <- lreff %>%
arrange(weighting) %>%    # First sort by val. This sort the dataframe but NOT the factor levels
  mutate(weighting=factor(weighting, levels=
                            c("R", "F", "U")))    # This trick update the factor levels


lreff.p.a <-ggplot(lreff) + geom_point(aes(y = estimate, x = weighting)) + geom_errorbar(aes(x=weighting,ymin=ci.lo, ymax=ci.up, width = 0.1))+
  geom_abline(intercept = 0, slope=0, colour = "darkgray", linetype="dashed") +
  xlab(NULL) +
 ylab(expression(italic(LR)))+
  theme_bw() + 
  theme(plot.title = element_text(size=10),axis.title = element_text(size=10),axis.text = element_text(size=9) )
lreff.p.a <-lreff.p.a +theme(axis.text.x = element_text(angle=0,face = c(rep('plain',16), 'bold', 'bold')))

cvreff <- data.frame(estimate=c(cvr.ma.ran$b,cvr.ma.un$b,cvr.ma.fix$b),ci.up=c(cvr.ma.ran$ci.ub,cvr.ma.un$ci.ub,cvr.ma.fix$ci.ub), ci.lo=c(cvr.ma.ran$ci.lb,cvr.ma.un$ci.lb,cvr.ma.fix$ci.lb), weighting=c("R", "U", "F"))
cvreff <- cvreff %>%
arrange(weighting) %>%    # First sort by val. This sort the dataframe but NOT the factor levels
  mutate(weighting=factor(weighting, levels=
                            c("R", "F", "U")))    # This trick update the factor levels


cvreff.p.a <-ggplot(cvreff) + geom_point(aes(y = estimate, x = weighting)) + geom_errorbar(aes(x=weighting,ymin=ci.lo, ymax=ci.up, width = 0.1))+
  geom_abline(intercept = 0, slope=0, colour = "darkgray", linetype="dashed") +
  xlab(NULL) +
 ylab(expression(italic(cvr)))+
  theme_bw() + 
  theme(plot.title = element_text(size=10),axis.title = element_text(size=10),axis.text = element_text(size=9) )
cvreff.p.a <-cvreff.p.a +theme(axis.text.x = element_text(angle=0,face = c(rep('plain',16), 'bold', 'bold')))


gridExtra::grid.arrange(hdeff.p.a, lreff.p.a, cvreff.p.a, ncol=3, widths = c(3, 2.3, 2.3))


#LR
#random, conventionally weighted
lr.ma.ran <- rma.mv(yi=scale(yi), V=vi, mods=log(t_qsize_m2), data=duglr, method="REML", random= list(~1 | id, ~1 | plot_id, ~1 | unit))
# unweighted 
lr.ma.un <- rma.mv(yi=scale(yi), V=vi, mods=log(t_qsize_m2), data=duglr, method="REML", random= list(~1 | id, ~1 | plot_id, ~1 | unit), W=1)
#fixed effect 
lr.ma.fix <- rma.mv(yi=scale(yi), V=vi, mods=log(t_qsize_m2), data=duglr, method="REML", random= list(~1 | id, ~1 | plot_id, ~1 | unit), W = 1/vi)


#cvr
#random, conventionally weighted
cvr.ma.ran <- rma.mv(yi=scale(yi), V=vi, mods=log(t_qsize_m2), data=dugcvr, method="REML", random= list(~1 | id, ~1 | plot_id, ~1 | unit))
# unweighted 
cvr.ma.un <- rma.mv(yi=scale(yi), V=vi, mods=log(t_qsize_m2), data=dugcvr, method="REML", random= list(~1 | id, ~1 | plot_id, ~1 | unit), W=1)
#fixed effect 
cvr.ma.fix <- rma.mv(yi=scale(yi), V=vi, mods=log(t_qsize_m2), data=dugcvr, method="REML", random= list(~1 | id, ~1 | plot_id, ~1 | unit), W = 1/vi)

#HG
#random, conventionally weighted
hg.ma.ran <- rma.mv(yi=scale(yi), V=vi, mods=log(t_qsize_m2), data=dughg, method="REML", random= list(~1 | id, ~1 | plot_id, ~1 | unit))
# unweighted 
hg.ma.un <- rma.mv(yi=scale(yi), V=vi, mods=log(t_qsize_m2), data=dughg, method="REML", random= list(~1 | id, ~1 | plot_id, ~1 | unit), W=1)
#fixed effects 
hg.ma.fix <- rma.mv(yi=scale(yi), V=vi, mods=log(t_qsize_m2), data=dughg, method="REML", random= list(~1 | id, ~1 | plot_id, ~1 | unit), W = 1/vi)

hg.ma.ran.d_alt <- rma.mv(yi=scale(yi), V=vi2, mods=log(t_qsize_m2), data=dughg, method="REML", random= list(~1 | id, ~1 | plot_id, ~1 | unit))

hg.ma.fix.dalt <- rma.mv(yi=scale(yi), V=vi2, mods=log(t_qsize_m2), data=dughg, method="REML", random= list(~1 | id, ~1 | plot_id, ~1 | unit), W = 1/vi2)

hdeff <- data.frame(estimate=c(hg.ma.ran$b[2],hg.ma.un$b[2],hg.ma.fix$b[2],hg.ma.ran.d_alt$b[2],hg.ma.fix.dalt$b[2]),ci.up=c(hg.ma.ran$ci.ub[2],hg.ma.un$ci.ub[2],hg.ma.fix$ci.ub[2], hg.ma.ran.d_alt$ci.ub[2],hg.ma.fix.dalt$ci.ub[2]), ci.lo=c(hg.ma.ran$ci.lb[2],hg.ma.un$ci.lb[2],hg.ma.fix$ci.lb[2],hg.ma.ran.d_alt$ci.lb[2],hg.ma.fix.dalt$ci.lb[2]), weighting=c("R", "U", "F", "R", "F"), vtype=c("d","d","d","d_alt","d_alt"))

hdeff <- hdeff %>%
arrange(weighting) %>%    # First sort by val. This sort the dataframe but NOT the factor levels
  mutate(weighting=factor(weighting, levels=
                            c("R", "F", "U")))    # This trick update the factor levels

hdeff.p.a <-ggplot(hdeff) + geom_point(aes(y = estimate, x = weighting, colour=vtype), position=position_dodge(width = 0.5)) + geom_errorbar(aes(x=weighting,ymin=ci.lo, ymax=ci.up, width = 0.1, colour=vtype),, position=position_dodge(width = 0.5))+
   geom_abline(intercept = 0, slope=0, colour = "darkgray", linetype="dashed",) +
  xlab(NULL) +
  ylab("Effect of plot size (regression coefficient)")+
  theme_bw() + 
  theme(plot.title = element_text(size=10),axis.title = element_text(size=10),axis.text = element_text(size=9) )
hdeff.p.a <-hdeff.p.a +theme(axis.text.x = element_text(angle=0,face = c(rep('plain',16), 'bold', 'bold')))+ylim(-1,1.2) +scale_color_manual(values=c("black", "darkgray"), guide=F)

lreff <- data.frame(estimate=c(lr.ma.ran$b[2],lr.ma.un$b[2],lr.ma.fix$b[2]),ci.up=c(lr.ma.ran$ci.ub[2],lr.ma.un$ci.ub[2],lr.ma.fix$ci.ub[2]), ci.lo=c(lr.ma.ran$ci.lb[2],lr.ma.un$ci.lb[2],lr.ma.fix$ci.lb[2]), weighting=c("R", "U", "F"))
lreff <- lreff %>%
arrange(weighting) %>%    # First sort by val. This sort the dataframe but NOT the factor levels
  mutate(weighting=factor(weighting, levels=
                            c("R", "F", "U")))    # This trick update the factor levels


lreff.p.a <-ggplot(lreff) + geom_point(aes(y = estimate, x = weighting)) + geom_errorbar(aes(x=weighting,ymin=ci.lo, ymax=ci.up, width = 0.1))+
  geom_abline(intercept = 0, slope=0, colour = "darkgray", linetype="dashed") +
  xlab(NULL) +
  ylab("Effect of plot size (regression coefficient)")+
  theme_bw() + 
  theme(plot.title = element_text(size=10),axis.title = element_text(size=10),axis.text = element_text(size=9) )
lreff.p.a <-lreff.p.a +theme(axis.text.x = element_text(angle=0,face = c(rep('plain',16), 'bold', 'bold')))+ylim(-1.2,1.5)


cvreff <- data.frame(estimate=c(cvr.ma.ran$b[2],cvr.ma.un$b[2],cvr.ma.fix$b[2]),ci.up=c(cvr.ma.ran$ci.ub[2],cvr.ma.un$ci.ub[2],cvr.ma.fix$ci.ub[2]), ci.lo=c(cvr.ma.ran$ci.lb[2],cvr.ma.un$ci.lb[2],cvr.ma.fix$ci.lb[2]), weighting=c("R", "U", "F"))
cvreff <- cvreff %>%
arrange(weighting) %>%    # First sort by val. This sort the dataframe but NOT the factor levels
  mutate(weighting=factor(weighting, levels=
                            c("R", "F", "U")))    # This trick update the factor levels


cvreff.p.a <-ggplot(cvreff) + geom_point(aes(y = estimate, x = weighting)) + geom_errorbar(aes(x=weighting,ymin=ci.lo, ymax=ci.up, width = 0.1))+
  geom_abline(intercept = 0, slope=0, colour = "darkgray", linetype="dashed") +
  xlab(NULL) +
  ylab("Effect of plot size (regression coefficient)")+
  theme_bw() + 
  theme(plot.title = element_text(size=10),axis.title = element_text(size=10),axis.text = element_text(size=9) )
cvreff.p.a <-cvreff.p.a +theme(axis.text.x = element_text(angle=0,face = c(rep('plain',16), 'bold', 'bold')))+ylim(-1.2,1.5)

gridExtra::grid.arrange(hdeff.p.a, lreff.p.a, cvreff.p.a, ncol=3, widths=c(3,2.3, 2.3))

hg.ma.ran <- rma.mv(yi=yi, V=vi, mods=log(t_qsize_m2), data=dughg, method="REML", random= list(~1 | id, ~1 | plot_id, ~1 | unit))
#random unweighted (same meta-est if fixed unweighted, but diff se)
hg.ma.un <- rma.mv(yi=yi, V=vi, mods=log(t_qsize_m2), data=dughg, method="REML", random= list(~1 | id, ~1 | plot_id, ~1 | unit), W=1)
#random effect but control the weights, do 1/v. same est as a fixed effect
hg.ma.fix <- rma.mv(yi=yi, V=vi, mods=log(t_qsize_m2), data=dughg, method="REML", random= list(~1 | id, ~1 | plot_id, ~1 | unit), W = 1/vi)

hg.ma.ran.d_alt <- rma.mv(yi=yi, V=vi2, mods=log(t_qsize_m2), data=dughg, method="REML", random= list(~1 | id, ~1 | plot_id, ~1 | unit))

hg.ma.fix.dalt <- rma.mv(yi=yi, V=vi2, mods=log(t_qsize_m2), data=dughg, method="REML", random= list(~1 | id, ~1 | plot_id, ~1 | unit), W = 1/vi2)


lr.ma.ran <- rma.mv(yi=yi, V=vi, mods=log(t_qsize_m2), data=duglr, method="REML", random= list(~1 | id, ~1 | plot_id, ~1 | unit))
#random unweighted (same meta-est if fixed unweighted, but diff se)
lr.ma.un <- rma.mv(yi=yi, V=vi, mods=log(t_qsize_m2), data=duglr, method="REML", random= list(~1 | id, ~1 | plot_id, ~1 | unit), W=1)
#random effect but control the weights, do 1/v. same est as a fixed effect
lr.ma.fix <- rma.mv(yi=yi, V=vi, mods=log(t_qsize_m2), data=duglr, method="REML", random= list(~1 | id, ~1 | plot_id, ~1 | unit), W = 1/vi)


cvr.ma.ran <- rma.mv(yi=yi, V=vi, mods=log(t_qsize_m2), data=dugcvr, method="REML", random= list(~1 | id, ~1 | plot_id, ~1 | unit))
#random unweighted (same meta-est if fixed unweighted, but diff se)
cvr.ma.un <- rma.mv(yi=yi, V=vi, mods=log(t_qsize_m2), data=dugcvr, method="REML", random= list(~1 | id, ~1 | plot_id, ~1 | unit), W=1)
#random effect but control the weights, do 1/v. same est as a fixed effect
cvr.ma.fix <- rma.mv(yi=yi, V=vi, mods=log(t_qsize_m2), data=dugcvr, method="REML", random= list(~1 | id, ~1 | plot_id, ~1 | unit), W = 1/vi)


newmods=data.frame(intercept=hg.ma.ran$b[1], t_qsize_m2=seq(min(dughg$t_qsize_m2), max(dughg$t_qsize_m2), 0.1) )
newmods=as.matrix(newmods)

hg.ma.ran.preds=data.frame(predict(hg.ma.ran,  addx=TRUE))
hg.ma.fix.preds=data.frame(predict(hg.ma.fix,  addx=TRUE))
hg.ma.un.preds=data.frame(predict(hg.ma.un,  addx=TRUE))

hg.ma.ran.p=ggplot()+geom_point(data=dughg,aes(x=log(t_qsize_m2), y=yi,colour=id), 
                                               #size=weights(hg.ma.ran)), 
                                alpha=0.3) +geom_line(aes(x=hg.ma.ran.preds$X.mods, y=hg.ma.ran.preds$pred))+geom_line(aes(x=hg.ma.ran.preds$X.mods, y=hg.ma.ran.preds$pred))+geom_ribbon(aes(x=hg.ma.ran.preds$X.mods,ymin=hg.ma.ran.preds$ci.lb, ymax=hg.ma.ran.preds$ci.ub ),alpha=0.2)+scale_size_continuous(guide=FALSE)+scale_color_discrete(guide=F)+theme_bw()+ylab(expression(italic(g)))+xlab(NULL)+geom_hline(yintercept = 0, linetype = "dashed") +ggtitle(expression(paste("Random-effects meta-analysis, wt = 1/(", italic("V"),"+",tau^2,")")))+theme(plot.title = element_text(size = 8))+ylim(-12,7.2)


hg.ma.fix.p=ggplot()+geom_point(data=dughg,aes(x=log(t_qsize_m2), y=yi,colour=id), #size=weights(hg.ma.fix)),
                                               alpha=0.3) +geom_line(aes(x=hg.ma.fix.preds$X.mods, y=hg.ma.fix.preds$pred))+geom_line(aes(x=hg.ma.fix.preds$X.mods, y=hg.ma.fix.preds$pred))+geom_ribbon(aes(x=hg.ma.fix.preds$X.mods,ymin=hg.ma.fix.preds$ci.lb, ymax=hg.ma.fix.preds$ci.ub ),alpha=0.2)+scale_size_continuous(guide=FALSE)+scale_color_discrete(guide=F)+theme_bw()+ylab(expression(italic(g)))+xlab(NULL)+geom_hline(yintercept = 0, linetype = "dashed")+ggtitle(expression(paste("Fixed-effects meta-analysis, wt = 1/(", italic("V"),")")))+theme(plot.title = element_text(size = 8))+ylim(-12,7.2)

hg.ma.un.p=ggplot()+geom_point(data=dughg,aes(x=log(t_qsize_m2), y=yi,colour=id, size=1), alpha=0.3) +geom_line(aes(x=hg.ma.fix.preds$X.mods, y=hg.ma.un.preds$pred))+geom_line(aes(x=hg.ma.un.preds$X.mods, y=hg.ma.un.preds$pred))+geom_ribbon(aes(x=hg.ma.un.preds$X.mods,ymin=hg.ma.un.preds$ci.lb, ymax=hg.ma.un.preds$ci.ub ),alpha=0.2)+scale_color_discrete(guide=F)+theme_bw()+ylab(expression(italic(g)))+xlab("Log(Plot size (ha))")+geom_hline(yintercept = 0, linetype = "dashed")+scale_size_continuous(guide=FALSE)+ggtitle("Unweighted meta-analysis, wt = 1")+theme(plot.title = element_text(size = 8))+ylim(-12,7.2)


newmods=data.frame(intercept=lr.ma.ran$b[1], t_qsize_m2=seq(min(duglr$t_qsize_m2), max(duglr$t_qsize_m2), 0.1) )
newmods=as.matrix(newmods)
head(newmods)
lr.ma.ran.preds=data.frame(predict(lr.ma.ran,  addx=TRUE))
lr.ma.fix.preds=data.frame(predict(lr.ma.fix,  addx=TRUE))
lr.ma.un.preds=data.frame(predict(lr.ma.un,  addx=TRUE))

lr.ma.ran.p=ggplot()+geom_point(data=duglr,aes(x=log(t_qsize_m2), y=yi,colour=id),# size=weights(lr.ma.ran)),
                                alpha=0.3) +geom_line(aes(x=lr.ma.ran.preds$X.mods, y=lr.ma.ran.preds$pred))+geom_line(aes(x=lr.ma.ran.preds$X.mods, y=lr.ma.ran.preds$pred))+geom_ribbon(aes(x=lr.ma.ran.preds$X.mods,ymin=lr.ma.ran.preds$ci.lb, ymax=lr.ma.ran.preds$ci.ub ),alpha=0.2)+scale_size_continuous(guide=FALSE)+scale_color_discrete(guide=F)+theme_bw()+ylab(expression(italic(LR)))+xlab(NULL)+geom_hline(yintercept = 0, linetype = "dashed") +ggtitle("")+theme(plot.title = element_text(size = 8))+ylim(-1.6,1.2)


lr.ma.fix.p=ggplot()+geom_point(data=duglr,aes(x=log(t_qsize_m2), y=yi,colour=id),# size=weights(lr.ma.fix)),
                                alpha=0.3) +geom_line(aes(x=lr.ma.fix.preds$X.mods, y=lr.ma.fix.preds$pred))+geom_line(aes(x=lr.ma.fix.preds$X.mods, y=lr.ma.fix.preds$pred))+geom_ribbon(aes(x=lr.ma.fix.preds$X.mods,ymin=lr.ma.fix.preds$ci.lb, ymax=lr.ma.fix.preds$ci.ub ),alpha=0.2)+scale_size_continuous(guide=FALSE)+scale_color_discrete(guide=F)+theme_bw()+ylab(expression(italic(LR)))+xlab(NULL)+geom_hline(yintercept = 0, linetype = "dashed")+ggtitle("")+theme(plot.title = element_text(size = 8))+coord_cartesian(ylim = c(-1.6, 1.2)) 


lr.ma.un.p=ggplot()+geom_point(data=duglr,aes(x=log(t_qsize_m2), y=yi,colour=id, size=1), alpha=0.3) +geom_line(aes(x=lr.ma.fix.preds$X.mods, y=lr.ma.un.preds$pred))+geom_line(aes(x=lr.ma.un.preds$X.mods, y=lr.ma.un.preds$pred))+geom_ribbon(aes(x=lr.ma.un.preds$X.mods,ymin=lr.ma.un.preds$ci.lb, ymax=lr.ma.un.preds$ci.ub ),alpha=0.2)+scale_color_discrete(guide=F)+theme_bw()+ylab(expression(italic(LR)))+xlab("Log(Plot size (ha))")+geom_hline(yintercept = 0, linetype = "dashed")+scale_size_continuous(guide=FALSE)+ggtitle("")+theme(plot.title = element_text(size = 8))+ylim(-1.6, 1.2)

newmods=data.frame(intercept=cvr.ma.ran$b[1], t_qsize_m2=seq(min(dugcvr$t_qsize_m2), max(dugcvr$t_qsize_m2), 0.1) )
newmods=as.matrix(newmods)

cvr.ma.ran.preds=data.frame(predict(cvr.ma.ran,  addx=TRUE))
cvr.ma.fix.preds=data.frame(predict(cvr.ma.fix,  addx=TRUE))
cvr.ma.un.preds=data.frame(predict(cvr.ma.un,  addx=TRUE))

cvr.ma.ran.p=ggplot()+geom_point(data=dugcvr,aes(x=log(t_qsize_m2), y=yi,colour=id),# size=weights(cvr.ma.ran)),
                                alpha=0.3) +geom_line(aes(x=cvr.ma.ran.preds$X.mods, y=cvr.ma.ran.preds$pred))+geom_line(aes(x=cvr.ma.ran.preds$X.mods, y=cvr.ma.ran.preds$pred))+geom_ribbon(aes(x=cvr.ma.ran.preds$X.mods,ymin=cvr.ma.ran.preds$ci.lb, ymax=cvr.ma.ran.preds$ci.ub ),alpha=0.2)+scale_size_continuous(guide=FALSE)+scale_color_discrete(guide=F)+theme_bw()+ylab(expression(italic(cvr)))+xlab(NULL)+geom_hline(yintercept = 0, linetype = "dashed") +ggtitle("")+theme(plot.title = element_text(size = 8))+ylim(-1.6,1.2)


cvr.ma.fix.p=ggplot()+geom_point(data=dugcvr,aes(x=log(t_qsize_m2), y=yi,colour=id),# size=weights(cvr.ma.fix)),
                                alpha=0.3) +geom_line(aes(x=cvr.ma.fix.preds$X.mods, y=cvr.ma.fix.preds$pred))+geom_line(aes(x=cvr.ma.fix.preds$X.mods, y=cvr.ma.fix.preds$pred))+geom_ribbon(aes(x=cvr.ma.fix.preds$X.mods,ymin=cvr.ma.fix.preds$ci.lb, ymax=cvr.ma.fix.preds$ci.ub ),alpha=0.2)+scale_size_continuous(guide=FALSE)+scale_color_discrete(guide=F)+theme_bw()+ylab(expression(italic(cvr)))+xlab(NULL)+geom_hline(yintercept = 0, linetype = "dashed")+ggtitle("")+theme(plot.title = element_text(size = 8))+coord_cartesian(ylim = c(-1.6, 1.2)) 


cvr.ma.un.p=ggplot()+geom_point(data=dugcvr,aes(x=log(t_qsize_m2), y=yi,colour=id, size=1), alpha=0.3) +geom_line(aes(x=cvr.ma.fix.preds$X.mods, y=cvr.ma.un.preds$pred))+geom_line(aes(x=cvr.ma.un.preds$X.mods, y=cvr.ma.un.preds$pred))+geom_ribbon(aes(x=cvr.ma.un.preds$X.mods,ymin=cvr.ma.un.preds$ci.lb, ymax=cvr.ma.un.preds$ci.ub ),alpha=0.2)+scale_color_discrete(guide=F)+theme_bw()+ylab(expression(italic(cvr)))+xlab("Log(Plot size (ha))")+geom_hline(yintercept = 0, linetype = "dashed")+scale_size_continuous(guide=FALSE)+ggtitle("")+theme(plot.title = element_text(size = 8))+ylim(-1.6, 1.2)


gridExtra::grid.arrange(hg.ma.ran.p, lr.ma.ran.p,cvr.ma.ran.p,
                        hg.ma.fix.p, lr.ma.fix.p,cvr.ma.fix.p,
                        hg.ma.un.p, lr.ma.un.p,cvr.ma.fix.p,
                        ncol=3, heights=c(2,2,2.2))#+ylim(-3.2, 0.7)
```

```
# studies with all three comparisons
re_ref %>% drop_na(c_mean, r_mean) %>% dplyr::select(id) %>% distinct()

# taxon breakdown
full_data %>% group_by(.$taxon) %>% summarise(n())

# metric breakdown
full_data %>% group_by(.$measure_type) %>% summarise(n())

# metric breakdown
full_data %>% group_by(.$plu) %>% summarise(n())

# size breakdown
full_data %>% drop_na(site_size, r_mean) %>% dplyr::summarise(n())

#final triple check of model values against in-text values
summary(mean_ur)
exp(.1815)
summary(cvr_ur)
exp(-0.1519)
summary(mean_rr)
exp(-0.1395)
summary(cvr_rr)
exp(0.183)
summary(mean_age_ur)
exp(0.006)

summary(cvr_age_ur)
summary(mean_age_rr)
summary(cvr_age_rr)
summary(mean_size_ur)
summary(cvr_size_ur)
summary(mean_size_rr)
summary(cvr_size_rr)

# resotration method breakdown
full_data %>% select(id, restoration_method) %>% distinct() %>%  group_by(restoration_method) %>% summarise(n())

# number of studies for each separate MA
full_data %>% drop_na(c_mean) %>% dplyr::select(id) %>%  distinct() %>%  summarise(n())
full_data %>% drop_na(r_mean) %>% dplyr::select(id) %>%  distinct() %>%  summarise(n())
full_data %>% drop_na(r_mean, c_mean) %>% dplyr::select(id) %>%  distinct() %>%  summarise(n())
full_data %>% select(id) %>%  distinct() %>%  summarise(n())
```

### Loading extra packages for taxon analysis

```
library(purrr)
library(multcomp)
```

### Custom functions

```
get_pred1 <- function(model, mod = " ") {
  name <- name <- firstup(as.character(stringr::str_replace(row.names(model$beta), 
                                                            mod, "")))
  len <- length(name)
  
  if (len != 1) {
    newdata <- matrix(NA, ncol = len, nrow = len)
    for (i in 1:len) {
      pos <- which(model$X[, i] == 1)[[1]]
      newdata[, i] <- model$X[pos, ]
    }
    pred <- metafor::predict.rma(model, newmods = newdata)
  } else {
    pred <- metafor::predict.rma(model)
  }
  estimate <- pred$pred
  lowerCL <- pred$ci.lb
  upperCL <- pred$ci.ub
  lowerPR <- pred$cr.lb
  upperPR <- pred$cr.ub
  
  table <- tibble(name = factor(name, levels = name, labels = name), estimate = estimate, 
                  lowerCL = lowerCL, upperCL = upperCL, pval = model$pval, lowerPR = lowerPR, 
                  upperPR = upperPR)
}

get_pred2 <- function(model, mod = " ") {
  name <- as.factor(str_replace(row.names(model$beta), paste0("relevel", "\\(", 
                                                              mod, ", ref = name", "\\)"), ""))
  len <- length(name)
  
  if (len != 1) {
    newdata <- diag(len)
    pred <- predict.rma(model, intercept = FALSE, newmods = newdata[, -1])
  } else {
    pred <- predict.rma(model)
  }
  estimate <- pred$pred
  lowerCL <- pred$ci.lb
  upperCL <- pred$ci.ub
  lowerPR <- pred$cr.lb
  upperPR <- pred$cr.ub
  
  table <- tibble(name = factor(name, levels = name, labels = name), estimate = estimate, 
                  lowerCL = lowerCL, upperCL = upperCL, pval = model$pval, lowerPR = lowerPR, 
                  upperPR = upperPR)
}


uni_mod_plot<-function(m, df, log_ratio, response, variance){
p <- predict.rma(m)
df %>% mutate(ymin = p$ci.lb, 
                                                  ymax = p$ci.ub, ymin2 = p$cr.lb, 
                                                  ymax2 = p$cr.ub, pred = p$pred) %>% 
  ggplot(aes(x = response, y = log_ratio, size = sqrt(1/variance))) + geom_point(shape = 21, alpha= 0.2,
                                                                     fill = "grey90") + 
  geom_hline(yintercept = 0, size = .5, colour = "gray70")+
  geom_smooth(aes(y = ymin2), method = "lm", se = FALSE, lty = "solid", lwd = 0.75, 
              colour = "#0072B2") + geom_smooth(aes(y = ymax2), method = "lm", se = FALSE, 
                                                lty = "solid", lwd = 0.75, colour = "#0072B2") + geom_smooth(aes(y = ymin), 
                                                                                                              method = "lm", se = FALSE, lty = "dashed", lwd = 0.75, colour = "#D55E00") + 
  geom_smooth(aes(y = ymax), method = "lm", se = FALSE, lty = "solid", lwd = 0.75, 
              colour = "#D55E00") + geom_smooth(aes(y = pred), method = "lm", se = FALSE, 
                                                lty = "solid", lwd = 1, colour = "black") + 
  labs(x = "\n ln(restoration site age)", y = "ln(restored/unrestored) - mean biodiversity", size = "Precision (1/SE)") + guides(fill = "none", 
                                                                                                                  colour = "none") + # themses
  theme_classic() + theme(legend.position = c(0, 1), legend.justification = c(0, 1)) + theme(legend.direction = "horizontal") + 
  theme(legend.background = element_blank()) + theme(axis.text.y = element_text(size = 8, 
                                                                                colour = "black", hjust = 0.5, angle = 90))+
  coord_cartesian(ylim = c(-2.5, 2.5))+
  scale_y_continuous(limits = c(-2.5, 2.5),
                     breaks = c(-2, -1, 0, 1, 2),
                     labels = c("\n \n -2", "100% decrease \n \n -1", "\n \n 0.0", "100% increase \n \n 1", "\n \n2")) +
  theme(legend.position = "none") 
}


uni_mod_plot_ns<-function(m, df, log_ratio, response, variance){
p <- predict.rma(m)
df %>% mutate(ymin = p$ci.lb, 
                                                  ymax = p$ci.ub, ymin2 = p$cr.lb, 
                                                  ymax2 = p$cr.ub, pred = p$pred) %>% 
  ggplot(aes(x = response, y = log_ratio, size = sqrt(1/variance))) + geom_point(shape = 21, alpha= 0.2,
                                                                     fill = "grey90") + 
  geom_hline(yintercept = 0, size = .5, colour = "gray70")+
  geom_smooth(aes(y = ymin2), method = "lm", se = FALSE, lty = "dashed", lwd = 0.75, 
              colour = "#0072B2") + geom_smooth(aes(y = ymax2), method = "lm", se = FALSE, 
                                                lty = "dashed", lwd = 0.75, colour = "#0072B2") + geom_smooth(aes(y = ymin), 
                                                                                                              method = "lm", se = FALSE, lty = "dashed", lwd = 0.75, colour = "#D55E00") + 
  geom_smooth(aes(y = ymax), method = "lm", se = FALSE, lty = "dashed", lwd = 0.75, 
              colour = "#D55E00") + geom_smooth(aes(y = pred), method = "lm", se = FALSE, 
                                                lty = "dashed", lwd = 1, colour = "black") + 
  labs(x = "\n ln(restoration site age)", y = "ln(restored/unrestored) - mean biodiversity", size = "Precision (1/SE)") + guides(fill = "none", 
                                                                                                                  colour = "none") + # themses
  theme_classic() + theme(legend.position = c(0, 1), legend.justification = c(0, 1)) + theme(legend.direction = "horizontal") + 
  theme(legend.background = element_blank()) + theme(axis.text.y = element_text(size = 8, 
                                                                                colour = "black", hjust = 0.5, angle = 90))+
  coord_cartesian(ylim = c(-2.5, 2.5))+
  scale_y_continuous(limits = c(-2.5, 2.5),
                     breaks = c(-2, -1, 0, 1, 2),
                     labels = c("\n \n -2", "100% decrease \n \n -1", "\n \n 0.0", "100% increase \n \n 1", "\n \n2")) +
  theme(legend.position = "none") 
}
```

```
dat<-read.csv("Data/variation_data.csv")
```

## Vegetation types

Below are three tables that summarise various subgroups within the
data including: the breakdown of broad vegetation types, categorised
into woody/non-woody. These are very coarse categories and “woody”
encompasses a large range of vegetation types from woodland to shrubland
to rainforest. Non-woody is a catch-all for herbaceous vegetation
communities, e.g. prairie, forb- or herb-dominated ecosystems.

**Table S11** Number of effect sizes included in the
meta-analysis by dominant vegetation

```
df<-dat
df %>% group_by(woody_nonwoody) %>% summarise(n()) %>% rename(`Dominant vegetation type` = woody_nonwoody, `Number of effect sizes` = `n()`) %>% kable("html")  %>% 
  kable_styling("striped", position = "left")
```

| Dominant vegetation type | Number of effect sizes |
| --- | --- |
| nonwoody | 406 |
| woody | 583 |

hile broad taxonomic groups were used for subgroup analyses in the
main-text, we include here the more detailed notes on taxon collected
during the literature search which may be of additional interest.

**Table S12** Number of effect sizes included in the
meta-analysis by taxon

```
df %>% group_by(taxon, taxon_detail) %>% summarise(n()) %>% rename(Taxon = taxon, Taxon_detail = taxon_detail, `Number of effect sizes` = `n()`) %>% kable("html")  %>% 
  kable_styling("striped", position = "left") %>%
    scroll_box(width = "800px", height = "300px")
```

| Taxon | Taxon\_detail | Number of effect sizes |
| --- | --- | --- |
| amoebae | amoebae | 12 |
| fungi | fungi | 41 |
| invertebrates | ants | 3 |
| invertebrates | arthropods | 55 |
| invertebrates | bees | 12 |
| invertebrates | beetles | 58 |
| invertebrates | birds | 1 |
| invertebrates | butterflies | 14 |
| invertebrates | diptera | 9 |
| invertebrates | dragonflies | 4 |
| invertebrates | epigeic beetles | 2 |
| invertebrates | floricolous beetles | 2 |
| invertebrates | invertebrates | 14 |
| invertebrates | leafhoppers | 1 |
| invertebrates | Lepidoptera | 2 |
| invertebrates | mites | 8 |
| invertebrates | moths | 2 |
| invertebrates | nematodes | 21 |
| invertebrates | odonata | 2 |
| invertebrates | orthoptera | 35 |
| invertebrates | pollinators | 4 |
| invertebrates | reptiles | 2 |
| invertebrates | saproxylic beetles | 2 |
| invertebrates | spiders | 13 |
| invertebrates | termites | 9 |
| invertebrates | vascular plants | 2 |
| invertebrates | vegetation | 3 |
| plants | bryophytes | 2 |
| plants | Eurasian steppe | 6 |
| plants | ferns | 8 |
| plants | grassland | 35 |
| plants | herbs | 10 |
| plants | lichens | 8 |
| plants | pollinators | 1 |
| plants | seedbank | 9 |
| plants | seeds | 2 |
| plants | shrubs | 2 |
| plants | steppe | 28 |
| plants | trees | 2 |
| plants | understorey plants | 9 |
| plants | vascular plants | 3 |
| plants | vegetation | 478 |
| plants | woody plants | 5 |
| soil microbes | soil microbes | 6 |
| vertebrates | amphibians | 3 |
| vertebrates | bats | 3 |
| vertebrates | birds | 12 |
| vertebrates | reptiles | 3 |
| vertebrates | small-medium mammals | 15 |
| vertebrates | small mammals | 6 |

While we conducted all analyses through the lens of mean
“biodiversity” and variability of “biodiversity”, biodiversity can be
measured in many different ways. Below we detail the variety of measures
of biodiversity included in the meta-analysis.

**Table S13** Number of effect sizes included in the
meta-analysis by measure of biodiversity

```
df %>% group_by(measure_type, measure) %>% summarise(n()) %>% rename(Measure = measure, Measure_detail = measure_type, `Number of effect sizes` = `n()`) %>% kable("html")  %>% 
  kable_styling("striped", position = "left")%>%
    scroll_box(width = "800px", height = "300px")
```

| Measure\_detail | Measure | Number of effect sizes |
| --- | --- | --- |
| functional | func\_disp | 11 |
| functional | func\_div | 66 |
| functional | func\_even | 55 |
| functional | func\_rich | 66 |
| functional | RaoQ | 45 |
| index | even | 58 |
| index | pielou | 12 |
| index | shan\_div | 16 |
| index | Shan\_div | 127 |
| index | Simp\_div | 34 |
| index | struc\_div | 1 |
| phylogenetic | phylo\_div | 9 |
| taxonomic | Chao1 | 9 |
| taxonomic | Chao2 | 4 |
| taxonomic | Jack1 | 3 |
| taxonomic | rare\_sp\_rich | 20 |
| taxonomic | sp\_div | 30 |
| taxonomic | sp\_rich | 422 |
| taxonomic | taxo\_distinct | 1 |

**Table S14** Number of effect sizes included in the
meta-analysis by measure of biodiversity

```
df %>% group_by(taxon, measure) %>% summarise(n()) %>% rename(Measure = measure, Taxon = taxon, `Number of effect sizes` = `n()`) %>% kable("html")  %>% 
  kable_styling("striped", position = "left")%>%
    scroll_box(width = "800px", height = "300px")
```

| Taxon | Measure | Number of effect sizes |
| --- | --- | --- |
| amoebae | Shan\_div | 6 |
| amoebae | sp\_rich | 6 |
| fungi | Chao1 | 8 |
| fungi | rare\_sp\_rich | 3 |
| fungi | Shan\_div | 11 |
| fungi | Simp\_div | 8 |
| fungi | sp\_rich | 11 |
| invertebrates | Chao2 | 3 |
| invertebrates | even | 29 |
| invertebrates | func\_disp | 3 |
| invertebrates | func\_even | 3 |
| invertebrates | func\_rich | 9 |
| invertebrates | Jack1 | 3 |
| invertebrates | pielou | 3 |
| invertebrates | rare\_sp\_rich | 17 |
| invertebrates | shan\_div | 4 |
| invertebrates | Shan\_div | 35 |
| invertebrates | Simp\_div | 9 |
| invertebrates | sp\_div | 8 |
| invertebrates | sp\_rich | 153 |
| invertebrates | taxo\_distinct | 1 |
| plants | Chao1 | 1 |
| plants | even | 29 |
| plants | func\_disp | 5 |
| plants | func\_div | 62 |
| plants | func\_even | 48 |
| plants | func\_rich | 53 |
| plants | phylo\_div | 9 |
| plants | pielou | 9 |
| plants | RaoQ | 45 |
| plants | shan\_div | 12 |
| plants | Shan\_div | 72 |
| plants | Simp\_div | 17 |
| plants | sp\_div | 7 |
| plants | sp\_rich | 238 |
| plants | struc\_div | 1 |
| soil microbes | sp\_div | 6 |
| vertebrates | Chao2 | 1 |
| vertebrates | func\_disp | 3 |
| vertebrates | func\_div | 4 |
| vertebrates | func\_even | 4 |
| vertebrates | func\_rich | 4 |
| vertebrates | Shan\_div | 3 |
| vertebrates | sp\_div | 9 |
| vertebrates | sp\_rich | 14 |

```
###########################
## UNRESTORED / RESTORED ##
###########################

# un_re = unrestored / restored 

un_re<-read.csv("Data/variation_data.csv", stringsAsFactors = F)

un_re$c_quad_n = as.numeric(un_re$c_quad_n)
un_re$c_mean =  as.numeric(un_re$c_mean)
un_re$c_sd = as.numeric(un_re$c_sd)

#remove studies with only a restored sites comparison
un_re<-un_re[!is.na(un_re$c_mean),]
#un_re %>% group_by(id, c_mean, c_sd) %>% distinct(shared_ctrl) %>% filter(n()>1) # checking shared controls is accurate

#calculate the lnCVR and lnRR and lnVR effect size and un_reiance with escalc
CVR<-escalc(measure = "CVR", n1i = un_re$t_quad_n, n2i = un_re$c_quad_n, m1i = un_re$t_mean, m2i = un_re$c_mean, sd1i = un_re$t_sd, sd2i = un_re$c_sd)
lnRR<-escalc(measure = "ROM", n1i = un_re$t_quad_n, n2i = un_re$c_quad_n, m1i = un_re$t_mean, m2i = un_re$c_mean, sd1i = un_re$t_sd, sd2i = un_re$c_sd)
lnVR<-escalc(measure = "VR", n1i = un_re$t_quad_n, n2i = un_re$c_quad_n, m1i = un_re$t_mean, m2i = un_re$c_mean, sd1i = un_re$t_sd, sd2i = un_re$c_sd)

#combined effect sizes with relevant un_rea frames
un_re <-bind_cols(un_re, lnRR, lnVR, CVR)

# name the un_rea something meaningful and remove all the columns unneeded
un_re<-un_re %>% rename(yi_mean = yi...36, vi_mean = vi...37, yi_vr = yi...38, vi_vr = vi...39, yi_cvr = yi...40, vi_cvr = vi...41)

#remove studies that have vi=NA - usually where control SD = 0
un_re<-un_re[!is.na(un_re$vi_vr),]

un_re$plu<-as.factor(un_re$plu)
un_re$plu<-relevel(un_re$plu, "semi-natural")

#need another random factor for 'unit'

unit <- factor(1:length(un_re$yi_mean))
un_re$unit <- unit

vcv_cvr<-make_VCV_matrix(un_re, V ="vi_cvr", "shared_ctrl", "unit", rho=0.5)
vcv_mean<-make_VCV_matrix(un_re, V ="vi_mean", "shared_ctrl", "unit", rho=0.5)
vcv_vr<-make_VCV_matrix(un_re, V ="vi_vr", "shared_ctrl", "unit", rho=0.5)


##########################
## RESTORED / REFERENCE ##
##########################

# re_ref = restored / reference 

re_ref<-read.csv("Data/variation_data.csv", stringsAsFactors = F)

#remove studies with only a degraded site comparison
re_ref<-re_ref[!is.na(re_ref$r_mean),]

#remove studies that have vi=NA - usually where control SD = 0
re_ref<-re_ref %>% filter(r_sd != 0)
re_ref<-re_ref %>% filter(!is.na(r_sd))

# there is a few sites where the reference control is shared, but the degraded one is not, need to add a "ref_shared_ctrl" to correct this
re_ref<-re_ref %>% group_by(id, r_mean, r_sd) %>% mutate(ref_shared_ctrl = cur_group_id())
#re_ref %>% group_by(id, r_mean, r_sd) %>% distinct(ref_shared_ctrl) %>% filter(n()>1) # to check any errors in the shared_control tagging

re_ref$r_quad_n = as.numeric(re_ref$r_quad_n)
re_ref$r_mean =  as.numeric(re_ref$r_mean)
re_ref$r_sd = as.numeric(re_ref$r_sd)

re_ref<-re_ref %>% filter(r_quad_n > 1) # a few sample sizes of 1 or 0?


#calculate the lnCVR and lnRR effect size and re_refiance with escalc
CVR<-escalc(measure = "CVR", n1i = re_ref$t_quad_n, n2i = re_ref$r_quad_n, m1i = re_ref$t_mean, m2i = re_ref$r_mean, sd1i = re_ref$t_sd, sd2i = re_ref$r_sd)
lnRR<-escalc(measure = "ROM", n1i = re_ref$t_quad_n, n2i = re_ref$r_quad_n, m1i = re_ref$t_mean, m2i = re_ref$r_mean, sd1i = re_ref$t_sd, sd2i = re_ref$r_sd)
lnVR<-escalc(measure = "VR", n1i = re_ref$t_quad_n, n2i = re_ref$r_quad_n, m1i = re_ref$t_mean, m2i = re_ref$r_mean, sd1i = re_ref$t_sd, sd2i = re_ref$r_sd)


#combined effect sizes with relevant data frames
re_ref <-bind_cols(re_ref, lnRR, lnVR, CVR)
# name the data something meaningful and remove all the columns unneeded
re_ref<-re_ref %>% rename(yi_mean = yi...37, vi_mean = vi...38, yi_vr = yi...39, vi_vr = vi...40, yi_cvr = yi...41, vi_cvr = vi...42)

re_ref$plu<-as.factor(re_ref$plu)
re_ref$plu<-relevel(re_ref$plu, "semi-natural")

#need another random factor for 'unit'

unit <- factor(1:length(re_ref$yi_mean))
re_ref$unit <- unit

re_ref<-as.data.frame(re_ref) # the group_by to do the shared control check above turns this bad boy into a tibble, needs to be a dataframe for the below function

vcv_cvr_rr<-make_VCV_matrix(data = re_ref, V ="vi_cvr", cluster = "ref_shared_ctrl", obs = "unit", rho=0.5)
vcv_mean_rr<-make_VCV_matrix(re_ref, V ="vi_mean", "ref_shared_ctrl", "unit", rho=0.5)
vcv_vr_rr<-make_VCV_matrix(re_ref, V ="vi_vr", "ref_shared_ctrl", "unit", rho=0.5)
```

## Meta-analytic models re-run including taxon as a moderator

Additionally, we test for any differences of both main effects and
age effects for each broad taxon category (‘plants’, ‘invertebrates’,
‘vertebrates’, ‘soil microbes’, ‘amoeba’, and ‘fungi’). First we run
meta-analytic models of LnCVR and LnRR for both restored/unrestored and
restored/reference comparisons. In Table S4, we print the Qm statistic
for these or the so-called “omnibus test” which to paraphrase Wolfgang
Viechtbauer (see http://www.metafor-project.org/doku.php/tips:testing\_factors\_lincoms
) for extended discussion) is to test if at least part of the
heterogeneity in the true effects is related to some of the variables in
the model (in this case, taxon).

```
cvr_ur <- rma.mv(yi_cvr, vcv_cvr, mods=~taxon, random = list(~1 | id, ~1 | plot_id, ~1 | unit), method = "REML", data = un_re) # can't remove intercept, or else we are testing whether the average true outcome is equal to 0 for all levels, not if there are between-group differences. grande differenza!!

mean_ur <- rma.mv(yi_mean, vcv_mean,mods=~taxon, random = list(~1 | id, ~1 | plot_id, ~1 | unit), method = "REML", data = un_re)
cvr_rr <- rma.mv(yi_cvr, vcv_cvr_rr, mods=~taxon,random = list(~1 | id, ~1 | plot_id, ~1 | unit), method = "REML", data = re_ref)
mean_rr <- rma.mv(yi_mean, vcv_mean_rr, mods=~taxon,random = list(~1 | id, ~1 | plot_id, ~1 | unit), method = "REML", data = re_ref)
```

**Table S15:** QM statistics, degrees of freedom, and
p-values for the test of moderators for each meta-analytic model.

```
options(scipen = 999)

x<-cbind(c("Log CV - restored/unrestored", "Log response ratio - restored/unrestored", "Log CV - restored/reference", "Log response ratio - restored/reference"), c(cvr_ur$QM, mean_ur$QM, cvr_rr$QM, mean_rr$QM),
c(cvr_ur$QMdf[1], mean_ur$QMdf[1], cvr_rr$QMdf[1], mean_rr$QMdf[1]),
c(cvr_ur$QMp, mean_ur$QMp, cvr_rr$QMp, mean_rr$QMp))

x<-`colnames<-`(x, c("Model", "QM", "QM df", "QM p value"))

x<-as.data.frame(x)

x<-x %>% mutate(QM =  as.numeric(QM),
             `QM df` =  as.numeric(`QM df`),
             `QM p value` =  as.numeric(`QM p value`))


x %>% kable("html", digits = 3) %>% 
  kable_styling("striped", position = "left")
```

| Model | QM | QM df | QM p value |
| --- | --- | --- | --- |
| Log CV - restored/unrestored | 9.798 | 5 | 0.081 |
| Log response ratio - restored/unrestored | 5.972 | 5 | 0.309 |
| Log CV - restored/reference | 1.184 | 4 | 0.881 |
| Log response ratio - restored/reference | 5.242 | 4 | 0.263 |

We see no evidence for this in any of the models, therefore do not
proceed to posthoc tests to adjust for the multiple comparisons and get
pairwise differences between groups.

```
# this was to make plots, which are current not printing because unecessary 

# cvrorgur<-orchard_plot(cvr_ur, mod="taxon", xlab = "log CV ratio - unrestored/restored", alpha = 0.1, k=T)+theme_classic()
# meanorgur<-orchard_plot(mean_ur, mod="taxon", xlab = "log response ratio - unrestored/restored", alpha = 0.1, k=T)+theme_classic()
# cvrorgrr<-orchard_plot(cvr_rr, mod="taxon", xlab = "log CV ratio - reference/restored", alpha = 0.1, k=T)+theme_classic()
# meanorgrr<-orchard_plot(mean_rr, mod="taxon", xlab = "log response ratio - reference/restored", alpha = 0.1, k=T)+theme_classic()
# 
# 
# (cvrorgur/meanorgur)+plot_annotation(tag_levels = "a", tag_suffix = ")")
```

```
# as above

# (cvrorgrr/meanorgrr)+plot_annotation(tag_levels = "a", tag_suffix = ")")
```

## Age effects by taxon

Next, we do the same thing to test if there is significant variation
in the interaction between `taxon` and `age`.

```
cvr_ur_age <- rma.mv(yi_cvr, vcv_cvr, mods = ~age.rest.:taxon, random = list(~1 | id, ~1 | plot_id, ~1 | unit), method = "REML", data = un_re)
mean_ur_age <- rma.mv(yi_mean, vcv_mean, mods = ~age.rest.:taxon, random = list(~1 | id, ~1 | plot_id, ~1 | unit), method = "REML", data = un_re)

cvr_rr_age <- rma.mv(yi_cvr, vcv_cvr_rr, mods = ~age.rest.:taxon, random = list(~1 | id, ~1 | plot_id, ~1 | unit), method = "REML", data = re_ref)
mean_rr_age <- rma.mv(yi_mean, vcv_mean_rr, mods = ~age.rest.:taxon, random = list(~1 | id, ~1 | plot_id, ~1 | unit), method = "REML", data = re_ref)


# cvrurage<-orchard_plot(cvr_ur_age, mod="taxon", xlab = "log(variability ratio) - unrestored/restored", alpha = 0.1, k=T)+theme_classic()
# meanurage<-orchard_plot(mean_ur_age, mod="taxon", xlab = "log(variability ratio) - unrestored/restored", alpha = 0.1, k=T)+theme_classic()
# cvrrrage<-orchard_plot(cvr_rr_age, mod="taxon", xlab = "log(variability ratio) - unrestored/restored", alpha = 0.1, k=T)+theme_classic()
# meanrrage<-orchard_plot(mean_rr_age, mod="taxon", xlab = "log(variability ratio) - unrestored/restored", alpha = 0.1, k=T)+theme_classic()
```

**Table S16:** QM statistics, degrees of freedom, and
p-values for the test of moderators for each meta-analytic model.

```
x<-cbind(c("Log CV - restored/unrestored (age:taxon interaction)", "Log response ratio - restored/unrestored (age:taxon interaction)", "Log CV - restored/reference (age:taxon interaction)", "Log response ratio - restored/reference (age:taxon interaction)"), c(cvr_ur_age$QM, mean_ur_age$QM, cvr_rr_age$QM, mean_rr_age$QM),
c(cvr_ur_age$QMdf[1], mean_ur_age$QMdf[1], cvr_rr_age$QMdf[1], mean_rr_age$QMdf[1]),
c(cvr_ur_age$QMp, mean_ur_age$QMp, cvr_rr_age$QMp, mean_rr_age$QMp))

x<-`colnames<-`(x, c("Model", "QM", "QM df", "QM p value"))

x<-as.data.frame(x)

x<-x %>% mutate(QM =  as.numeric(QM),
             `QM df` =  as.numeric(`QM df`),
             `QM p value` =  as.numeric(`QM p value`))


x %>% kable("html", digits = 3) %>% 
  kable_styling("striped", position = "left")
```

| Model | QM | QM df | QM p value |
| --- | --- | --- | --- |
| Log CV - restored/unrestored (age:taxon interaction) | 5.465 | 6 | 0.486 |
| Log response ratio - restored/unrestored (age:taxon interaction) | 19.643 | 6 | 0.003 |
| Log CV - restored/reference (age:taxon interaction) | 7.722 | 5 | 0.172 |
| Log response ratio - restored/reference (age:taxon interaction) | 2.359 | 5 | 0.798 |

Here, we can see that there is evidence that some of the
heterogeneity in the “true effect” is due to the included moderators

## Subgroup analyses between taxon and interacting with age

Last, since we had a significant QM statistic in the LnRR model
comparing restored/unrestored model, we conduct a posthoc test below to
test for differences between each group (each group being the
interaction between taxon and age of restored site). We have to run a
new model without the intercept, because otherwise we are including that
in the combinations of pairs (see discussion here: https://stats.stackexchange.com/questions/324885/easy-post-hoc-tests-when-meta-analyzing-with-the-metafor-package-in-r
and Wolfgang Viechtbauer’s discussion of this here http://www.metafor-project.org/doku.php/tips:testing\_factors\_lincoms).

In the code, but not printed in the output, we run a second posthoc
test adjusted for multiplicity just to be thorough, also finding no
differences.

**Table S17.** Pairwise comparison between subgroups
(taxon:age interaction) for log response ratio between restored and
unrestored sites.

```
mean_ur_age <- rma.mv(yi_mean, vcv_mean, mods = ~age.rest.:taxon-1, random = list(~1 | id, ~1 | plot_id, ~1 | unit), method = "REML", data = un_re)

rrmean_age<-summary(glht(mean_ur_age, linfct=cbind(contrMat(rep(1,6), type="Tukey"))), test=adjusted("none"))
#rrmean_age<-summary(glht(mean_ur_age, linfct=cbind(contrMat(rep(1,6), type="Tukey"))), test=adjusted("Westfall")) # check using a correction for multiplicity, no different so leaving along to keep consistent with the above models

# plus, on reading Westfall/Shaffer, it seems that corrections reduces the Type I error rate, and then not correcting reduces the Type II error - which since thre is n.s. results either way this is not that important here? 


rrmeanage<-as.data.frame(cbind(rrmean_age$test$coefficients, rrmean_age$test$sigma, rrmean_age$test$tstat, rrmean_age$test$pvalues)) %>%
  rename(coefficient = V1,
         sigma = V2,
         tstat = V3,
         p = V4)

a<-bind_cols(c(1:6), c("amoebae", "fungi", "invertebrates", "plants", "soil microbes", "vertebrates")) %>% rename(num = `...1`, tax = `...2`)

rmeanage<-as.data.frame(rownames(rrmeanage)) %>% rename(comp = `rownames(rrmeanage)`) %>% mutate(ref = as.numeric(word(comp, 2, sep = "-")), comparison = as.numeric(word(comp, 1, sep = "-"))) %>% left_join(., a, by = c("ref" = "num")) %>% rename(reference_taxa = tax) %>%
  left_join(., a, by = c("comparison" = "num")) %>% rename(comparison_taxa = tax) %>% bind_cols(., rrmeanage)

rownames(rmeanage) <- NULL

rmeanage %>% dplyr::select(-c(1:3)) %>% kable(digits = 3) %>% kable_styling()%>%
    scroll_box(width = "800px", height = "350px")
```

| reference\_taxa | comparison\_taxa | coefficient | sigma | tstat | p |
| --- | --- | --- | --- | --- | --- |
| amoebae | fungi | 0.050 | 0.030 | 1.663 | 0.096 |
| amoebae | invertebrates | 0.023 | 0.021 | 1.096 | 0.273 |
| amoebae | plants | 0.028 | 0.021 | 1.315 | 0.189 |
| amoebae | soil microbes | 0.018 | 0.022 | 0.789 | 0.430 |
| amoebae | vertebrates | 0.032 | 0.022 | 1.440 | 0.150 |
| fungi | invertebrates | -0.027 | 0.022 | -1.243 | 0.214 |
| fungi | plants | -0.023 | 0.022 | -1.040 | 0.299 |
| fungi | soil microbes | -0.033 | 0.023 | -1.413 | 0.158 |
| fungi | vertebrates | -0.019 | 0.023 | -0.819 | 0.413 |
| invertebrates | plants | 0.004 | 0.003 | 1.684 | 0.092 |
| invertebrates | soil microbes | -0.005 | 0.008 | -0.685 | 0.493 |
| invertebrates | vertebrates | 0.009 | 0.007 | 1.220 | 0.222 |
| plants | soil microbes | -0.010 | 0.008 | -1.248 | 0.212 |
| plants | vertebrates | 0.004 | 0.007 | 0.603 | 0.547 |
| soil microbes | vertebrates | 0.014 | 0.010 | 1.372 | 0.170 |

## R Session Information

```
library(pander)
sessionInfo() %>% pander()
```

**R version 4.1.2 (2021-11-01)**

**Platform:** x86\_64-w64-mingw32/x64 (64-bit)

**locale:** *LC\_COLLATE=English\_Australia.1252*,
*LC\_CTYPE=English\_Australia.1252*,
*LC\_MONETARY=English\_Australia.1252*, *LC\_NUMERIC=C* and
*LC\_TIME=English\_Australia.1252*

**attached base packages:** *stats*,
*graphics*, *grDevices*, *utils*,
*datasets*, *methods* and *base*

**other attached packages:** *pander(v.0.6.4)*,
*multcomp(v.1.4-18)*, *TH.data(v.1.1-0)*,
*MASS(v.7.3-54)*, *survival(v.3.2-13)*,
*mvtnorm(v.1.1-3)*, *knitr(v.1.37)*,
*rworldmap(v.1.3-6)*, *sp(v.1.4-6)*,
*ggtext(v.0.1.1)*, *jtools(v.2.1.4)*,
*gridExtra(v.2.3)*, *sjPlot(v.2.8.10)*,
*patchwork(v.1.1.1)*, *orchaRd(v.0.0.0.9000)*,
*kableExtra(v.1.3.4)*, *rmarkdown(v.2.12)*,
*forcats(v.0.5.1)*, *stringr(v.1.4.0)*,
*dplyr(v.1.0.8)*, *purrr(v.0.3.4)*,
*readr(v.2.1.2)*, *tidyr(v.1.2.0)*,
*tibble(v.3.1.6)*, *ggplot2(v.3.3.5)*,
*tidyverse(v.1.3.1)*, *readxl(v.1.3.1)*,
*broom(v.0.7.12)*, *metafor(v.3.0-2)* and
*Matrix(v.1.3-4)*

**loaded via a namespace (and not attached):**
*ggbeeswarm(v.0.6.0)*, *minqa(v.1.2.4)*,
*colorspace(v.2.0-3)*, *ellipsis(v.0.3.2)*,
*sjlabelled(v.1.1.8)*, *estimability(v.1.3)*,
*markdown(v.1.1)*, *parameters(v.0.16.0)*,
*fs(v.1.5.2)*, *gridtext(v.0.1.4)*,
*rstudioapi(v.0.13)*, *farver(v.2.1.0)*,
*bit64(v.4.0.5)*, *fansi(v.1.0.2)*,
*lubridate(v.1.8.0)*, *mathjaxr(v.1.6-0)*,
*xml2(v.1.3.3)*, *codetools(v.0.2-18)*,
*splines(v.4.1.2)*, *sjmisc(v.2.8.9)*,
*spam(v.2.8-0)*, *jsonlite(v.1.8.0)*,
*nloptr(v.2.0.0)*, *ggeffects(v.1.1.1)*,
*dbplyr(v.2.1.1)*, *effectsize(v.0.6.0.1)*,
*compiler(v.4.1.2)*, *httr(v.1.4.2)*,
*sjstats(v.0.18.1)*, *emmeans(v.1.7.2)*,
*backports(v.1.4.1)*, *assertthat(v.0.2.1)*,
*fastmap(v.1.1.0)*, *cli(v.3.2.0)*,
*htmltools(v.0.5.2)*, *tools(v.4.1.2)*,
*dotCall64(v.1.0-1)*, *gtable(v.0.3.0)*,
*glue(v.1.6.2)*, *maps(v.3.4.0)*, *Rcpp(v.1.0.8)*,
*cellranger(v.1.1.0)*, *jquerylib(v.0.1.4)*,
*vctrs(v.0.3.8)*, *svglite(v.2.1.0)*,
*nlme(v.3.1-153)*, *insight(v.0.16.0)*,
*xfun(v.0.29)*, *lme4(v.1.1-28)*, *rvest(v.1.0.2)*,
*lifecycle(v.1.0.1)*, *zoo(v.1.8-9)*,
*scales(v.1.1.1)*, *vroom(v.1.5.7)*,
*hms(v.1.1.1)*, *parallel(v.4.1.2)*,
*sandwich(v.3.0-1)*, *fields(v.13.3)*,
*yaml(v.2.3.5)*, *sass(v.0.4.0)*,
*stringi(v.1.7.6)*, *highr(v.0.9)*,
*bayestestR(v.0.11.5)*, *maptools(v.1.1-2)*,
*boot(v.1.3-28)*, *rlang(v.1.0.1)*,
*pkgconfig(v.2.0.3)*, *systemfonts(v.1.0.4)*,
*evaluate(v.0.15)*, *lattice(v.0.20-45)*,
*labeling(v.0.4.2)*, *bit(v.4.0.4)*,
*tidyselect(v.1.1.2)*, *magrittr(v.2.0.2)*,
*R6(v.2.5.1)*, *generics(v.0.1.2)*, *DBI(v.1.1.2)*,
*foreign(v.0.8-81)*, *pillar(v.1.7.0)*,
*haven(v.2.4.3)*, *withr(v.2.5.0)*,
*mgcv(v.1.8-38)*, *datawizard(v.0.2.3)*,
*performance(v.0.8.0)*, *modelr(v.0.1.8)*,
*crayon(v.1.5.0)*, *utf8(v.1.2.2)*,
*tzdb(v.0.2.0)*, *viridis(v.0.6.2)*,
*grid(v.4.1.2)*, *reprex(v.2.0.1)*,
*digest(v.0.6.29)*, *webshot(v.0.5.2)*,
*xtable(v.1.8-4)*, *munsell(v.0.5.0)*,
*beeswarm(v.0.4.0)*, *viridisLite(v.0.4.0)*,
*vipor(v.0.4.5)* and *bslib(v.0.3.1)*

LS0tDQp0aXRsZTogIlRlcnJlc3RyaWFsIGVjb3N5c3RlbSByZXN0b3JhdGlvbiBpbmNyZWFzZXMgYmlvZGl2ZXJzaXR5IGFuZCByZWR1Y2VzIGl0cyB2YXJpYWJpbGl0eSwgYnV0IG5vdCB0byByZWZlcmVuY2UgbGV2ZWxzOiBhIGdsb2JhbCBtZXRhLWFuYWx5c2lzIg0KYXV0aG9yOiAiSm9lIEF0a2luc29uLCBMYXJzIEJydWR2aWcsIE1heCBNYWxsZW4tQ29vcGVyLCBTaGluaWNoaSBOYWthZ2F3YSwgQW5nZWxhIFQuIE1vbGVzLCBTdGVwaGVuIFAuIEJvbnNlciINCmRhdGU6ICJgciBmb3JtYXQoU3lzLnRpbWUoKSwgJyVkICVCICVZJylgIg0Kb3V0cHV0Og0KICBodG1sX2RvY3VtZW50Og0KICAgIGNvZGVfZm9sZGluZzogaGlkZQ0KICAgIGNvZGVfZG93bmxvYWQ6IHRydWUNCiAgICBkZXB0aDogNA0KICAgIG51bWJlcl9zZWN0aW9uczogbm8NCiAgICB0aGVtZTogIGpvdXJuYWwgIyDigJxkZWZhdWx04oCdLCDigJxjZXJ1bGVhbuKAnSwg4oCcY29zbW/igJ0sIOKAnGZsYXRseeKAnSwg4oCcZGFya2x54oCdLCDigJxyZWFkYWJsZeKAnSwg4oCcc3BhY2VsYWLigJ0sIOKAnHVuaXRlZOKAnSwg4oCcY29zbW/igJ0sIOKAnGx1bWVu4oCdLCDigJxwYXBlcuKAnSwg4oCcc2FuZHN0b25l4oCdLCDigJxzaW1wbGV44oCdLCDigJx5ZXRp4oCdDQogICAgdG9jOiB5ZXMNCiAgICB0b2NfZmxvYXQ6IHllcw0KICAgIHRvY19kZXB0aDogNA0KICBwZGZfZG9jdW1lbnQ6DQogICAgdG9jOiB5ZXMNCnN1YnRpdGxlOiBFbGVjdHJvbmljIFN1cHBsZW1lbnRhcnkgTWF0ZXJpYWwNCi0tLQ0KDQpgYGB7ciBzZXR1cCwgaW5jbHVkZSA9IEZBTFNFfQ0KIyBrbml0ZXIgc2V0dGluZw0Ka25pdHI6Om9wdHNfY2h1bmskc2V0KA0KICBtZXNzYWdlID0gRkFMU0UsDQogIHdhcm5pbmcgPSBGQUxTRSwgIyBubyB3YXJuaW5ncw0KICBjYWNoZSA9IFRSVUUsDQogIGVjaG8gPSBUUlVFDQopDQoNCg0KYGBgDQoNCiMjIFNldHVwcw0KDQojIyMgTG9hZGluZyBwYWNrYWdlcyBhbmQgY3VzdG9tIGZ1bmN0aW9ucw0KDQpUbyBydW4gdGhlIGZvbGxvd2luZyBzY3JpcHQsIHNvbWUgcGFja2FnZXMgbWF5IG5lZWQgdG8gYmUgaW5zdGFsbGVkIGZyb20gYEdpdGh1YmAuDQoNCmBgYHtyfQ0KIyBpbnN0YWxsLnBhY2thZ2VzKCJkZXZ0b29scyIpDQojIGluc3RhbGwucGFja2FnZXMoInRpZHl2ZXJzZSIpDQojIGluc3RhbGwucGFja2FnZXMoIm1ldGFmb3IiKQ0KIyBpbnN0YWxsLnBhY2thZ2VzKCJwYXRjaHdvcmsiKQ0KIyBpbnN0YWxsLnBhY2thZ2VzKCJSLnJzcCIpDQojIA0KIyBkZXZ0b29sczo6aW5zdGFsbF9naXRodWIoIml0Y2h5c2hpbi9vcmNoYXJkX3Bsb3QiLCBzdWJkaXIgPSAib3JjaGFSZCIsIGZvcmNlID0gVFJVRSwgYnVpbGRfdmlnbmV0dGVzID0gVFJVRSkNCg0KbGlicmFyeShtZXRhZm9yKQ0KbGlicmFyeShicm9vbSkNCmxpYnJhcnkocmVhZHhsKQ0KbGlicmFyeSh0aWR5dmVyc2UpDQpsaWJyYXJ5KHJtYXJrZG93bikNCmxpYnJhcnkoa2FibGVFeHRyYSkNCmxpYnJhcnkob3JjaGFSZCkNCmxpYnJhcnkocGF0Y2h3b3JrKQ0KbGlicmFyeShzalBsb3QpDQpsaWJyYXJ5KGdyaWRFeHRyYSkNCmxpYnJhcnkoanRvb2xzKQ0KbGlicmFyeShnZ3RleHQpDQpsaWJyYXJ5KHB1cnJyKQ0KYGBgDQoNCiMjIyBDdXN0b20gZnVuY3Rpb25zDQoNCg0KYGBge3J9DQoNCiMnIEB0aXRsZSBDb3ZhcmlhbmNlIGFuZCBjb3JyZWxhdGlvbiBtYXRyaXggZnVuY3Rpb24gYmFzaW5nIG9uIHNoYXJlZCBsZXZlbCBJRA0KIycgQGRlc2NyaXB0aW9uIEZ1bmN0aW9uIGZvciBnZW5lcmF0aW5nIHNpbXBsZSBjb3ZhcmlhbmNlIGFuZCBjb3JyZWxhdGlvbiBtYXRyaWNlcyANCiMnIEBwYXJhbSBkYXRhIERhdGFmcmFtZSBvYmplY3QgY29udGFpbmluZyBlZmZlY3Qgc2l6ZXMsIHRoZWlyIHZhcmlhbmNlLCB1bmlxdWUgSURzIGFuZCBjbHVzdGVyaW5nIHZhcmlhYmxlDQojJyBAcGFyYW0gViBOYW1lIG9mIHRoZSB2YXJpYWJsZSAoYXMgYSBzdHJpbmcg4oCTIGUuZywgIlYxIikgY29udGFpbmluZyBlZmZlY3Qgc2l6ZSB2YXJpYW5jZXMgdmFyaWFuY2VzDQojJyBAcGFyYW0gY2x1c3RlciBOYW1lIG9mIHRoZSB2YXJpYWJsZSAoYXMgYSBzdHJpbmcg4oCTIGUuZywgIlYxIikgaW5kaWNhdGluZyB3aGljaCBlZmZlY3RzIGJlbG9uZyB0byB0aGUgc2FtZSBjbHVzdGVyLiBTYW1lIHZhbHVlIG9mICdjbHVzdGVyJyBhcmUgYXNzdW1lZCB0byBiZSBub25pbmRlcGVuZGVudCAoY29ycmVsYXRlZCkuDQojJyBAcGFyYW0gb2JzIE5hbWUgb2YgdGhlIHZhcmlhYmxlIChhcyBhIHN0cmluZyDigJMgZS5nLCAiVjEiKSBjb250YWluaW5nIGluZGl2aWR1YWwgSURzIGZvciBlYWNoIHZhbHVlIGluIHRoZSBWIChWZWN0b3Igb2YgdmFyaWFuY2VzKS4gSWYgdGhpcyBwYXJhbWV0ZXIgaXMgbWlzc2luZywgbGFiZWwgd2lsbCBiZSBsYWJlbGxlZCB3aXRoIGNvbnNlY3V0aXZlIGludGVnZXJzIHN0YXJ0aW5nIGZyb20gMS4NCiMnIEBwYXJhbSByaG8gS25vd24gb3IgYXNzdW1lZCBjb3JyZWxhdGlvbiB2YWx1ZSBhbW9uZyBlZmZlY3Qgc2l6ZXMgc2hhcmluZyBzYW1lICdjbHVzdGVyJyB2YWx1ZS4gRGVmYXVsdCB2YWx1ZSBpcyAwLjUuDQojJyBAcGFyYW0gdHlwZSBPcHRpb25hbCBsb2dpY2FsIHBhcmFtZXRlciBpbmRpY2F0aW5nIHdoZXRoZXIgYSBmdWxsIHZhcmlhbmNlLWNvdmFyaWFuY2UgbWF0cml4IChkZWZhdWx0IG9yICJ2Y3YiKSBpcyBuZWVkZWQgb3IgYSBjb3JyZWxhdGlvbiBtYXRyaXggKCJjb3IiKSBmb3IgdGhlIG5vbi1pbmRlcGVuZGVudCBibG9ja3Mgb2YgdmFyaWFuY2UgdmFsdWVzLg0KIycgQGV4cG9ydA0KDQptYWtlX1ZDVl9tYXRyaXggPC0gZnVuY3Rpb24oZGF0YSwgViwgY2x1c3Rlciwgb2JzLCB0eXBlPWMoInZjdiIsICJjb3IiKSwgcmhvPTAuNSl7DQogIHR5cGUgPC0gbWF0Y2guYXJnKHR5cGUpDQogIGlmIChtaXNzaW5nKGRhdGEpKSB7DQogICAgc3RvcCgiTXVzdCBzcGVjaWZ5IGRhdGFmcmFtZSB2aWEgJ2RhdGEnIGFyZ3VtZW50LiIpDQogIH0NCiAgaWYgKG1pc3NpbmcoVikpIHsNCiAgICBzdG9wKCJNdXN0IHNwZWNpZnkgbmFtZSBvZiB0aGUgdmFyaWFuY2UgdmFyaWFibGUgdmlhICdWJyBhcmd1bWVudC4iKQ0KICB9DQogIGlmIChtaXNzaW5nKGNsdXN0ZXIpKSB7DQogICAgc3RvcCgiTXVzdCBzcGVjaWZ5IG5hbWUgb2YgdGhlIGNsdXN0ZXJpbmcgdmFyaWFibGUgdmlhICdjbHVzdGVyJyBhcmd1bWVudC4iKQ0KICB9DQogIGlmIChtaXNzaW5nKG9icykpIHsNCiAgICBvYnMgPC0gMTpsZW5ndGgoVikgICANCiAgfQ0KICBpZiAobWlzc2luZyh0eXBlKSkgew0KICAgIHR5cGUgPC0gInZjdiIgDQogIH0NCiAgDQogIG5ld19tYXRyaXggPC0gbWF0cml4KDAsbnJvdyA9IGRpbShkYXRhKVsxXSxuY29sID0gZGltKGRhdGEpWzFdKSAjbWFrZSBlbXB0eSBtYXRyaXggb2YgdGhlIHNhbWUgc2l6ZSBhcyBkYXRhIGxlbmd0aA0KICByb3duYW1lcyhuZXdfbWF0cml4KSA8LSBkYXRhWyAsb2JzXQ0KICBjb2xuYW1lcyhuZXdfbWF0cml4KSA8LSBkYXRhWyAsb2JzXQ0KICAjIGZpbmQgc3RhcnQgYW5kIGVuZCBjb29yZGluYXRlcyBmb3IgdGhlIHN1YnNldHMNCiAgc2hhcmVkX2Nvb3JkIDwtIHdoaWNoKGRhdGFbICxjbHVzdGVyXSAlaW4lIGRhdGFbZHVwbGljYXRlZChkYXRhWyAsY2x1c3Rlcl0pLCBjbHVzdGVyXT09VFJVRSkNCiAgIyBtYXRyaXggb2YgY29tYmluYXRpb25zIG9mIGNvb3JkaW5hdGVzIGZvciBlYWNoIGV4cGVyaW1lbnQgd2l0aCBzaGFyZWQgY29udHJvbA0KICBjb21iaW5hdGlvbnMgPC0gZG8uY2FsbCgicmJpbmQiLCB0YXBwbHkoc2hhcmVkX2Nvb3JkLCBkYXRhW3NoYXJlZF9jb29yZCxjbHVzdGVyXSwgZnVuY3Rpb24oeCkgdCh1dGlsczo6Y29tYm4oeCwyKSkpKQ0KICANCiAgaWYodHlwZSA9PSAidmN2Iil7DQogICAgIyBjYWxjdWxhdGUgY292YXJpYW5jZSB2YWx1ZXMgYmV0d2VlbiAgdmFsdWVzIGF0IHRoZSBwb3NpdGlvbnMgaW4gc2hhcmVkX2xpc3QgYW5kIHBsYWNlIHRoZW0gb24gdGhlIG1hdHJpeA0KICAgIGZvciAoaSBpbiAxOmRpbShjb21iaW5hdGlvbnMpWzFdKXsNCiAgICAgIHAxIDwtIGNvbWJpbmF0aW9uc1tpLDFdDQogICAgICBwMiA8LSBjb21iaW5hdGlvbnNbaSwyXQ0KICAgICAgcDFfcDJfY292IDwtIHJobyAqIHNxcnQoZGF0YVtwMSxWXSkgKiBzcXJ0KGRhdGFbcDIsVl0pDQogICAgICBuZXdfbWF0cml4W3AxLHAyXSA8LSBwMV9wMl9jb3YNCiAgICAgIG5ld19tYXRyaXhbcDIscDFdIDwtIHAxX3AyX2Nvdg0KICAgIH0NCiAgICBkaWFnKG5ld19tYXRyaXgpIDwtIGRhdGFbICxWXSAgICNhZGQgdGhlIGRpYWdvbmFsDQogIH0NCiAgDQogIGlmKHR5cGUgPT0gImNvciIpew0KICAgICMgY2FsY3VsYXRlIGNvdmFyaWFuY2UgdmFsdWVzIGJldHdlZW4gIHZhbHVlcyBhdCB0aGUgcG9zaXRpb25zIGluIHNoYXJlZF9saXN0IGFuZCBwbGFjZSB0aGVtIG9uIHRoZSBtYXRyaXgNCiAgICBmb3IgKGkgaW4gMTpkaW0oY29tYmluYXRpb25zKVsxXSl7DQogICAgICBwMSA8LSBjb21iaW5hdGlvbnNbaSwxXQ0KICAgICAgcDIgPC0gY29tYmluYXRpb25zW2ksMl0NCiAgICAgIHAxX3AyX2NvdiA8LSByaG8NCiAgICAgIG5ld19tYXRyaXhbcDEscDJdIDwtIHAxX3AyX2Nvdg0KICAgICAgbmV3X21hdHJpeFtwMixwMV0gPC0gcDFfcDJfY292DQogICAgfQ0KICAgIGRpYWcobmV3X21hdHJpeCkgPC0gMSAgICNhZGQgdGhlIGRpYWdvbmFsIG9mIDENCiAgfQ0KICANCiAgcmV0dXJuKG5ld19tYXRyaXgpDQp9DQoNCg0KIycgQHRpdGxlIG1vZGVsX3RhYmxlOiB1bml2YXJpYXRlIHJtYS5tdiBtb2RlbHMNCiMnIEBkZXNjcmlwdGlvbiBGdW5jdGlvbiB0byBnZXQgZXN0aW1hdGVzLCBDSXMgKGNvbmZpZGVuY2UgaW50ZXJ2YWxzKSBmcm9tIHJtYSBvYmplY3RzIChtZXRhZm9yKSBhbmQgb3V0cHV0IGludG8gbmVhdCB0YWJsZSAtIHRoaXMgb25lIGRlc2lnbmVkIGZvciB0aHJlZSBtb2RlbHMgYXQgYSB0aW1lIA0KIycgQHBhcmFtIG0xOiBmaXJzdCBybWEubXYgb2JqZWN0IA0KIycgQHBhcmFtIG0yOiBzZWNvbmQgcm1hLm12IG9iamVjdA0KIycgQHBhcmFtIG0zOiB0aGlyZCBybWEubXYgb2JqZWN0DQojJyBAcGFyYW0gbmFtZXM6IGxpc3Qgb2YgbmFtZXMgZm9yIHRoZSAiRWZmZWN0IHNpemUiIGNvbHVtbiwgbXVzdCBiZSBsZW5ndGggdGhyZWUgYW5kIGNvcnJlc3BvbmQgdG8gbTEsbTIsbTMgZWZmZWN0IHNpemVzDQoNCm1vZGVsX3RhYmxlPC1mdW5jdGlvbihtMSwgbTIsIG0zLCBuYW1lcyl7DQogICNyMiA8LSByMl9tbChtb2RlbDEsIG1vZGVsMiwgbW9kZWwzKQ0KICANCiAgIyBjcmVhdGluZyBhIHRhYmxlDQogIA0KICB0aWJibGUoYEVmZmVjdCBzaXplYCA9IG5hbWVzLA0KICAgICAgICAgYEVzdGltYXRlYCA9IGMobTEkYiwgbTIkYiwgbTMkYiksIA0KICAgICAgICAgYExvd2VyIENJIFswLjAyNV1gID0gYyhtMSRjaS5sYiwgbTIkY2kubGIsIG0zJGNpLmxiKSwgDQogICAgICAgICBgVXBwZXIgQ0kgIFswLjk3NV1gID0gYyhtMSRjaS51YiwgbTIkY2kudWIsIG0zJGNpLnViKSwgDQogICAgICAgICBgUCB2YWx1ZWAgPSBjKG0xJHB2YWwsIG0yJHB2YWwsIG0zJHB2YWwpKSAlPiUga2FibGUoImh0bWwiLCBkaWdpdHMgPSAzKSAlPiUgDQogICAga2FibGVfc3R5bGluZyhwb3NpdGlvbiA9ICJsZWZ0IikgDQogIA0KICANCn0NCg0KbW9kZWxfdGFibGU8LWZ1bmN0aW9uKG0xLCBtMiwgbTMsIG5hbWVzKXsNCiAgI3IyIDwtIHIyX21sKG1vZGVsMSwgbW9kZWwyLCBtb2RlbDMpDQogIA0KICAjIGNyZWF0aW5nIGEgdGFibGUNCiAgDQogIHRpYmJsZShgRWZmZWN0IHNpemVgID0gbmFtZXMsDQogICAgICAgICBgRXN0aW1hdGVgID0gYyhtMSRiLCBtMiRiLCBtMyRiKSwgDQogICAgICAgICBgTG93ZXIgQ0kgWzAuMDI1XWAgPSBjKG0xJGNpLmxiLCBtMiRjaS5sYiwgbTMkY2kubGIpLCANCiAgICAgICAgIGBVcHBlciBDSSAgWzAuOTc1XWAgPSBjKG0xJGNpLnViLCBtMiRjaS51YiwgbTMkY2kudWIpLCANCiAgICAgICAgIGBQIHZhbHVlYCA9IGMobTEkcHZhbCwgbTIkcHZhbCwgbTMkcHZhbCkpICU+JSBrYWJsZSgiaHRtbCIsIGRpZ2l0cyA9IDMpICU+JSANCiAgICBrYWJsZV9zdHlsaW5nKHBvc2l0aW9uID0gImxlZnQiKSANCiAgDQogIA0KfQ0KDQpnZXRfcHJlZDEgPC0gZnVuY3Rpb24obW9kZWwsIG1vZCA9ICIgIikgew0KICBuYW1lIDwtIG5hbWUgPC0gZmlyc3R1cChhcy5jaGFyYWN0ZXIoc3RyaW5ncjo6c3RyX3JlcGxhY2Uocm93Lm5hbWVzKG1vZGVsJGJldGEpLCANCiAgICAgICAgICAgICAgICAgICAgICAgICAgICAgICAgICAgICAgICAgICAgICAgICAgICAgICAgICAgIG1vZCwgIiIpKSkNCiAgbGVuIDwtIGxlbmd0aChuYW1lKQ0KICANCiAgaWYgKGxlbiAhPSAxKSB7DQogICAgbmV3ZGF0YSA8LSBtYXRyaXgoTkEsIG5jb2wgPSBsZW4sIG5yb3cgPSBsZW4pDQogICAgZm9yIChpIGluIDE6bGVuKSB7DQogICAgICBwb3MgPC0gd2hpY2gobW9kZWwkWFssIGldID09IDEpW1sxXV0NCiAgICAgIG5ld2RhdGFbLCBpXSA8LSBtb2RlbCRYW3BvcywgXQ0KICAgIH0NCiAgICBwcmVkIDwtIG1ldGFmb3I6OnByZWRpY3Qucm1hKG1vZGVsLCBuZXdtb2RzID0gbmV3ZGF0YSkNCiAgfSBlbHNlIHsNCiAgICBwcmVkIDwtIG1ldGFmb3I6OnByZWRpY3Qucm1hKG1vZGVsKQ0KICB9DQogIGVzdGltYXRlIDwtIHByZWQkcHJlZA0KICBsb3dlckNMIDwtIHByZWQkY2kubGINCiAgdXBwZXJDTCA8LSBwcmVkJGNpLnViDQogIGxvd2VyUFIgPC0gcHJlZCRjci5sYg0KICB1cHBlclBSIDwtIHByZWQkY3IudWINCiAgDQogIHRhYmxlIDwtIHRpYmJsZShuYW1lID0gZmFjdG9yKG5hbWUsIGxldmVscyA9IG5hbWUsIGxhYmVscyA9IG5hbWUpLCBlc3RpbWF0ZSA9IGVzdGltYXRlLCANCiAgICAgICAgICAgICAgICAgIGxvd2VyQ0wgPSBsb3dlckNMLCB1cHBlckNMID0gdXBwZXJDTCwgcHZhbCA9IG1vZGVsJHB2YWwsIGxvd2VyUFIgPSBsb3dlclBSLCANCiAgICAgICAgICAgICAgICAgIHVwcGVyUFIgPSB1cHBlclBSKQ0KfQ0KDQpnZXRfcHJlZDIgPC0gZnVuY3Rpb24obW9kZWwsIG1vZCA9ICIgIikgew0KICBuYW1lIDwtIGFzLmZhY3RvcihzdHJfcmVwbGFjZShyb3cubmFtZXMobW9kZWwkYmV0YSksIHBhc3RlMCgicmVsZXZlbCIsICJcXCgiLCANCiAgICAgICAgICAgICAgICAgICAgICAgICAgICAgICAgICAgICAgICAgICAgICAgICAgICAgICAgICAgICAgbW9kLCAiLCByZWYgPSBuYW1lIiwgIlxcKSIpLCAiIikpDQogIGxlbiA8LSBsZW5ndGgobmFtZSkNCiAgDQogIGlmIChsZW4gIT0gMSkgew0KICAgIG5ld2RhdGEgPC0gZGlhZyhsZW4pDQogICAgcHJlZCA8LSBwcmVkaWN0LnJtYShtb2RlbCwgaW50ZXJjZXB0ID0gRkFMU0UsIG5ld21vZHMgPSBuZXdkYXRhWywgLTFdKQ0KICB9IGVsc2Ugew0KICAgIHByZWQgPC0gcHJlZGljdC5ybWEobW9kZWwpDQogIH0NCiAgZXN0aW1hdGUgPC0gcHJlZCRwcmVkDQogIGxvd2VyQ0wgPC0gcHJlZCRjaS5sYg0KICB1cHBlckNMIDwtIHByZWQkY2kudWINCiAgbG93ZXJQUiA8LSBwcmVkJGNyLmxiDQogIHVwcGVyUFIgPC0gcHJlZCRjci51Yg0KICANCiAgdGFibGUgPC0gdGliYmxlKG5hbWUgPSBmYWN0b3IobmFtZSwgbGV2ZWxzID0gbmFtZSwgbGFiZWxzID0gbmFtZSksIGVzdGltYXRlID0gZXN0aW1hdGUsIA0KICAgICAgICAgICAgICAgICAgbG93ZXJDTCA9IGxvd2VyQ0wsIHVwcGVyQ0wgPSB1cHBlckNMLCBwdmFsID0gbW9kZWwkcHZhbCwgbG93ZXJQUiA9IGxvd2VyUFIsIA0KICAgICAgICAgICAgICAgICAgdXBwZXJQUiA9IHVwcGVyUFIpDQp9DQoNCg0KbW9kX3RhYjwtZnVuY3Rpb24obSl7DQojIGdldHRpbmcgbWFyZ2luYWwgUjINCiAgDQpyMiA8LSByMl9tbChtKQ0KDQojIGNyZWF0aW5nIGEgdGFibGUNCnRpYmJsZShgRml4ZWQgZWZmZWN0YCA9IHJvdy5uYW1lcyhtJGJldGEpLCBFc3RpbWF0ZSA9IGMobSRiKSwgDQogICAgICAgYExvd2VyIENJIFswLjAyNV1gID0gYyhtJGNpLmxiKSwgYFVwcGVyIENJICBbMC45NzVdYCA9IGMobSRjaS51YiksIA0KICAgICAgIGBQIHZhbHVlYCA9IGMobSRwdmFsKSwgUjIgPSBjKHIyWzFdLCAgICAgICAgcmVwKE5BLCAobGVuZ3RoKG0kYmV0YSktMSkpKSkgJT4lIGthYmxlKCJodG1sIiwgZGlnaXRzID0gMykgJT4lIGthYmxlX3N0eWxpbmcoInN0cmlwZWQiLCBwb3NpdGlvbiA9ICJsZWZ0IikgDQp9DQoNCnVuaV9tb2RfcGxvdDwtZnVuY3Rpb24obSwgZGYsIGxvZ19yYXRpbywgcmVzcG9uc2UsIHZhcmlhbmNlKXsNCnAgPC0gcHJlZGljdC5ybWEobSkNCmRmICU+JSBtdXRhdGUoeW1pbiA9IHAkY2kubGIsIA0KICAgICAgICAgICAgICAgICAgICAgICAgICAgICAgICAgICAgICAgICAgICAgICAgICB5bWF4ID0gcCRjaS51YiwgeW1pbjIgPSBwJGNyLmxiLCANCiAgICAgICAgICAgICAgICAgICAgICAgICAgICAgICAgICAgICAgICAgICAgICAgICAgeW1heDIgPSBwJGNyLnViLCBwcmVkID0gcCRwcmVkKSAlPiUgDQogIGdncGxvdChhZXMoeCA9IHJlc3BvbnNlLCB5ID0gbG9nX3JhdGlvLCBzaXplID0gc3FydCgxL3ZhcmlhbmNlKSkpICsgZ2VvbV9wb2ludChzaGFwZSA9IDIxLCBhbHBoYT0gMC4yLA0KICAgICAgICAgICAgICAgICAgICAgICAgICAgICAgICAgICAgICAgICAgICAgICAgICAgICAgICAgICAgICAgICAgICAgZmlsbCA9ICJncmV5OTAiKSArIA0KICBnZW9tX2hsaW5lKHlpbnRlcmNlcHQgPSAwLCBzaXplID0gLjUsIGNvbG91ciA9ICJncmF5NzAiKSsNCiAgZ2VvbV9zbW9vdGgoYWVzKHkgPSB5bWluMiksIG1ldGhvZCA9ICJsbSIsIHNlID0gRkFMU0UsIGx0eSA9ICJzb2xpZCIsIGx3ZCA9IDAuNzUsIA0KICAgICAgICAgICAgICBjb2xvdXIgPSAiIzAwNzJCMiIpICsgZ2VvbV9zbW9vdGgoYWVzKHkgPSB5bWF4MiksIG1ldGhvZCA9ICJsbSIsIHNlID0gRkFMU0UsIA0KICAgICAgICAgICAgICAgICAgICAgICAgICAgICAgICAgICAgICAgICAgICAgICAgbHR5ID0gInNvbGlkIiwgbHdkID0gMC43NSwgY29sb3VyID0gIiMwMDcyQjIiKSArIGdlb21fc21vb3RoKGFlcyh5ID0geW1pbiksIA0KICAgICAgICAgICAgICAgICAgICAgICAgICAgICAgICAgICAgICAgICAgICAgICAgICAgICAgICAgICAgICAgICAgICAgICAgICAgICAgICAgICAgICAgICAgICAgICAgICAgICAgICAgICAgICBtZXRob2QgPSAibG0iLCBzZSA9IEZBTFNFLCBsdHkgPSAic29saWQiLCBsd2QgPSAwLjc1LCBjb2xvdXIgPSAiI0Q1NUUwMCIpICsgDQogIGdlb21fc21vb3RoKGFlcyh5ID0geW1heCksIG1ldGhvZCA9ICJsbSIsIHNlID0gRkFMU0UsIGx0eSA9ICJzb2xpZCIsIGx3ZCA9IDAuNzUsIA0KICAgICAgICAgICAgICBjb2xvdXIgPSAiI0Q1NUUwMCIpICsgZ2VvbV9zbW9vdGgoYWVzKHkgPSBwcmVkKSwgbWV0aG9kID0gImxtIiwgc2UgPSBGQUxTRSwgDQogICAgICAgICAgICAgICAgICAgICAgICAgICAgICAgICAgICAgICAgICAgICAgICBsdHkgPSAic29saWQiLCBsd2QgPSAxLCBjb2xvdXIgPSAiYmxhY2siKSArIA0KICBsYWJzKHggPSAiXG4gbG4ocmVzdG9yYXRpb24gc2l0ZSBhZ2UpIiwgeSA9ICJsbihyZXN0b3JlZC91bnJlc3RvcmVkKSAtIG1lYW4gYmlvZGl2ZXJzaXR5Iiwgc2l6ZSA9ICJQcmVjaXNpb24gKDEvU0UpIikgKyBndWlkZXMoZmlsbCA9ICJub25lIiwgDQogICAgICAgICAgICAgICAgICAgICAgICAgICAgICAgICAgICAgICAgICAgICAgICAgICAgICAgICAgICAgICAgICAgICAgICAgICAgICAgICAgICAgICAgICAgICAgICAgICAgICAgICAgICAgICAgICBjb2xvdXIgPSAibm9uZSIpICsgIyB0aGVtc2VzDQogIHRoZW1lX2NsYXNzaWMoKSArIHRoZW1lKGxlZ2VuZC5wb3NpdGlvbiA9IGMoMCwgMSksIGxlZ2VuZC5qdXN0aWZpY2F0aW9uID0gYygwLCAxKSkgKyB0aGVtZShsZWdlbmQuZGlyZWN0aW9uID0gImhvcml6b250YWwiKSArIA0KICB0aGVtZShsZWdlbmQuYmFja2dyb3VuZCA9IGVsZW1lbnRfYmxhbmsoKSkgKyB0aGVtZShheGlzLnRleHQueSA9IGVsZW1lbnRfdGV4dChzaXplID0gOCwgDQogICAgICAgICAgICAgICAgICAgICAgICAgICAgICAgICAgICAgICAgICAgICAgICAgICAgICAgICAgICAgICAgICAgICAgICAgICAgICAgIGNvbG91ciA9ICJibGFjayIsIGhqdXN0ID0gMC41LCBhbmdsZSA9IDkwKSkrDQogIGNvb3JkX2NhcnRlc2lhbih5bGltID0gYygtMi41LCAyLjUpKSsNCiAgc2NhbGVfeV9jb250aW51b3VzKGxpbWl0cyA9IGMoLTIuNSwgMi41KSwNCiAgICAgICAgICAgICAgICAgICAgIGJyZWFrcyA9IGMoLTIsIC0xLCAwLCAxLCAyKSwNCiAgICAgICAgICAgICAgICAgICAgIGxhYmVscyA9IGMoIlxuIFxuIC0yIiwgIjEwMCUgZGVjcmVhc2UgXG4gXG4gLTEiLCAiXG4gXG4gMC4wIiwgIjEwMCUgaW5jcmVhc2UgXG4gXG4gMSIsICJcbiBcbjIiKSkgKw0KICB0aGVtZShsZWdlbmQucG9zaXRpb24gPSAibm9uZSIpIA0KfQ0KDQoNCnVuaV9tb2RfcGxvdF9uczwtZnVuY3Rpb24obSwgZGYsIGxvZ19yYXRpbywgcmVzcG9uc2UsIHZhcmlhbmNlKXsNCnAgPC0gcHJlZGljdC5ybWEobSkNCmRmICU+JSBtdXRhdGUoeW1pbiA9IHAkY2kubGIsIA0KICAgICAgICAgICAgICAgICAgICAgICAgICAgICAgICAgICAgICAgICAgICAgICAgICB5bWF4ID0gcCRjaS51YiwgeW1pbjIgPSBwJGNyLmxiLCANCiAgICAgICAgICAgICAgICAgICAgICAgICAgICAgICAgICAgICAgICAgICAgICAgICAgeW1heDIgPSBwJGNyLnViLCBwcmVkID0gcCRwcmVkKSAlPiUgDQogIGdncGxvdChhZXMoeCA9IHJlc3BvbnNlLCB5ID0gbG9nX3JhdGlvLCBzaXplID0gc3FydCgxL3ZhcmlhbmNlKSkpICsgZ2VvbV9wb2ludChzaGFwZSA9IDIxLCBhbHBoYT0gMC4yLA0KICAgICAgICAgICAgICAgICAgICAgICAgICAgICAgICAgICAgICAgICAgICAgICAgICAgICAgICAgICAgICAgICAgICAgZmlsbCA9ICJncmV5OTAiKSArIA0KICBnZW9tX2hsaW5lKHlpbnRlcmNlcHQgPSAwLCBzaXplID0gLjUsIGNvbG91ciA9ICJncmF5NzAiKSsNCiAgZ2VvbV9zbW9vdGgoYWVzKHkgPSB5bWluMiksIG1ldGhvZCA9ICJsbSIsIHNlID0gRkFMU0UsIGx0eSA9ICJkYXNoZWQiLCBsd2QgPSAwLjc1LCANCiAgICAgICAgICAgICAgY29sb3VyID0gIiMwMDcyQjIiKSArIGdlb21fc21vb3RoKGFlcyh5ID0geW1heDIpLCBtZXRob2QgPSAibG0iLCBzZSA9IEZBTFNFLCANCiAgICAgICAgICAgICAgICAgICAgICAgICAgICAgICAgICAgICAgICAgICAgICAgIGx0eSA9ICJkYXNoZWQiLCBsd2QgPSAwLjc1LCBjb2xvdXIgPSAiIzAwNzJCMiIpICsgZ2VvbV9zbW9vdGgoYWVzKHkgPSB5bWluKSwgDQogICAgICAgICAgICAgICAgICAgICAgICAgICAgICAgICAgICAgICAgICAgICAgICAgICAgICAgICAgICAgICAgICAgICAgICAgICAgICAgICAgICAgICAgICAgICAgICAgICAgICAgICAgICAgIG1ldGhvZCA9ICJsbSIsIHNlID0gRkFMU0UsIGx0eSA9ICJkYXNoZWQiLCBsd2QgPSAwLjc1LCBjb2xvdXIgPSAiI0Q1NUUwMCIpICsgDQogIGdlb21fc21vb3RoKGFlcyh5ID0geW1heCksIG1ldGhvZCA9ICJsbSIsIHNlID0gRkFMU0UsIGx0eSA9ICJkYXNoZWQiLCBsd2QgPSAwLjc1LCANCiAgICAgICAgICAgICAgY29sb3VyID0gIiNENTVFMDAiKSArIGdlb21fc21vb3RoKGFlcyh5ID0gcHJlZCksIG1ldGhvZCA9ICJsbSIsIHNlID0gRkFMU0UsIA0KICAgICAgICAgICAgICAgICAgICAgICAgICAgICAgICAgICAgICAgICAgICAgICAgbHR5ID0gImRhc2hlZCIsIGx3ZCA9IDEsIGNvbG91ciA9ICJibGFjayIpICsgDQogIGxhYnMoeCA9ICJcbiBsbihyZXN0b3JhdGlvbiBzaXRlIGFnZSkiLCB5ID0gImxuKHJlc3RvcmVkL3VucmVzdG9yZWQpIC0gbWVhbiBiaW9kaXZlcnNpdHkiLCBzaXplID0gIlByZWNpc2lvbiAoMS9TRSkiKSArIGd1aWRlcyhmaWxsID0gIm5vbmUiLCANCiAgICAgICAgICAgICAgICAgICAgICAgICAgICAgICAgICAgICAgICAgICAgICAgICAgICAgICAgICAgICAgICAgICAgICAgICAgICAgICAgICAgICAgICAgICAgICAgICAgICAgICAgICAgICAgICAgIGNvbG91ciA9ICJub25lIikgKyAjIHRoZW1zZXMNCiAgdGhlbWVfY2xhc3NpYygpICsgdGhlbWUobGVnZW5kLnBvc2l0aW9uID0gYygwLCAxKSwgbGVnZW5kLmp1c3RpZmljYXRpb24gPSBjKDAsIDEpKSArIHRoZW1lKGxlZ2VuZC5kaXJlY3Rpb24gPSAiaG9yaXpvbnRhbCIpICsgDQogIHRoZW1lKGxlZ2VuZC5iYWNrZ3JvdW5kID0gZWxlbWVudF9ibGFuaygpKSArIHRoZW1lKGF4aXMudGV4dC55ID0gZWxlbWVudF90ZXh0KHNpemUgPSA4LCANCiAgICAgICAgICAgICAgICAgICAgICAgICAgICAgICAgICAgICAgICAgICAgICAgICAgICAgICAgICAgICAgICAgICAgICAgICAgICAgICAgY29sb3VyID0gImJsYWNrIiwgaGp1c3QgPSAwLjUsIGFuZ2xlID0gOTApKSsNCiAgY29vcmRfY2FydGVzaWFuKHlsaW0gPSBjKC0yLjUsIDIuNSkpKw0KICBzY2FsZV95X2NvbnRpbnVvdXMobGltaXRzID0gYygtMi41LCAyLjUpLA0KICAgICAgICAgICAgICAgICAgICAgYnJlYWtzID0gYygtMiwgLTEsIDAsIDEsIDIpLA0KICAgICAgICAgICAgICAgICAgICAgbGFiZWxzID0gYygiXG4gXG4gLTIiLCAiMTAwJSBkZWNyZWFzZSBcbiBcbiAtMSIsICJcbiBcbiAwLjAiLCAiMTAwJSBpbmNyZWFzZSBcbiBcbiAxIiwgIlxuIFxuMiIpKSArDQogIHRoZW1lKGxlZ2VuZC5wb3NpdGlvbiA9ICJub25lIikgDQp9DQoNCg0KdW5pX2VnZ2VyX3Bsb3RfY3ZyPC1mdW5jdGlvbihtLCBkYXRhKXsjIGdldHRpbmcgbWFyZ2luYWwgUjINCnIyIDwtIHIyX21sKG0pDQojIGdldHRpbmcgZXN0aW1hdGVzOiBuYW1lIGRvZXMgbm90IHdvcmsgZm9yIHNsb3Blcw0KZXN0IDwtIGdldF9lc3QobSwgbW9kID0gInNxcnQodmkpIikNCg0KDQojIGNyZWF0aW5nIGEgdGFibGUNCnRpYmJsZShgRml4ZWQgZWZmZWN0YCA9IHJvdy5uYW1lcyhtJGJldGEpLCBFc3RpbWF0ZSA9IGMoZXN0JGVzdGltYXRlKSwgDQogICAgICAgYExvd2VyIENJIFswLjAyNV1gID0gYyhlc3QkbG93ZXJDTCksIGBVcHBlciBDSSAgWzAuOTc1XWAgPSBjKGVzdCR1cHBlckNMKSwgDQogICAgICAgYFAgdmFsdWVgID0gYyhtJHB2YWwpLCBSMiA9IGMocjJbMV0sIA0KICAgICAgICAgICAgICAgICAgICAgICAgICAgICAgICAgICAgICAgICAgICAgICAgICAgICAgICAgICAgICBOQSkpICU+JSBrYWJsZSgiaHRtbCIsIGRpZ2l0cyA9IDMpICU+JSBrYWJsZV9zdHlsaW5nKCJzdHJpcGVkIiwgcG9zaXRpb24gPSAibGVmdCIpDQoNCg0KcHJlZCA8LSBwcmVkaWN0LnJtYShtKQ0KDQoNCg0KIyBwbG90dGluZw0KZml0IDwtIGRhdGEgJT4lIGRyb3BfbmEodmlfY3ZyKSU+JSBtdXRhdGUoeW1pbiA9IHByZWQkY2kubGIsIHltYXggPSBwcmVkJGNpLnViLCB5bWluMiA9IHByZWQkY3IubGIsIHltYXgyID0gcHJlZCRjci51YiwgcHJlZCA9IHByZWQkcHJlZCkgJT4lIA0KICBnZ3Bsb3QoYWVzKHggPSBzcXJ0KHZpX2N2ciksIHkgPSB5aV9jdnIsIHNpemUgPSBzcXJ0KDEvdmlfY3ZyKSkpICsgZ2VvbV9wb2ludChzaGFwZSA9IDIxLCBmaWxsID0gImdyZXk5MCIsIGFscGhhID0gMC4zKSArIA0KICBnZW9tX3Ntb290aChhZXMoeSA9IHltaW4yKSwgbWV0aG9kID0gImxvZXNzIiwgc2UgPSBGQUxTRSwgbHR5ID0gImRvdHRlZCIsIGx3ZCA9IDAuMjUsIGNvbG91ciA9ICIjMDA3MkIyIikgKyANCiAgZ2VvbV9zbW9vdGgoYWVzKHkgPSB5bWF4MiksIG1ldGhvZCA9ICJsb2VzcyIsIHNlID0gRkFMU0UsIGx0eSA9ICJkb3R0ZWQiLCBsd2QgPSAwLjI1LCBjb2xvdXIgPSAiIzAwNzJCMiIpICsgDQogIGdlb21fc21vb3RoKGFlcyh5ID0geW1pbiksIG1ldGhvZCA9ICJsb2VzcyIsIHNlID0gRkFMU0UsIGx0eSA9ICJkb3R0ZWQiLCBsd2QgPSAwLjI1LCBjb2xvdXIgPSAiI0Q1NUUwMCIpICsgDQogIGdlb21fc21vb3RoKGFlcyh5ID0geW1heCksIG1ldGhvZCA9ICJsb2VzcyIsIHNlID0gRkFMU0UsIGx0eSA9ICJkb3R0ZWQiLCBsd2QgPSAwLjI1LCBjb2xvdXIgPSAiI0Q1NUUwMCIpICsNCiAgZ2VvbV9zbW9vdGgoYWVzKHkgPSBwcmVkKSwgbWV0aG9kID0gImxvZXNzIiwgc2UgPSBGQUxTRSwgbHR5ID0gImRhc2hlZCIsIGx3ZCA9IDAuNSwgY29sb3VyID0gImJsYWNrIikgKw0KICBsYWJzKHggPSAic3FydChzYW1wbGluZyB2YXJpYW5jZSkiLCB5ID0gImxuUlIgKGVmZmVjdCBzaXplKSIsIHNpemUgPSAiUHJlY2lzaW9uICgxL1NFKSIpICsgDQogIGd1aWRlcyhmaWxsID0gIm5vbmUiLCBjb2xvdXIgPSAibm9uZSIpICsgDQogIHRoZW1lX2J3KCkgKyB0aGVtZShsZWdlbmQucG9zaXRpb24gPSBjKDAsIDEpLCBsZWdlbmQuanVzdGlmaWNhdGlvbiA9IGMoMCwgMSkpICsgdGhlbWUobGVnZW5kLmRpcmVjdGlvbiA9ICJob3Jpem9udGFsIikgKyANCiAgdGhlbWUobGVnZW5kLmJhY2tncm91bmQgPSBlbGVtZW50X2JsYW5rKCkpICsgdGhlbWUoYXhpcy50ZXh0LnkgPSBlbGVtZW50X3RleHQoc2l6ZSA9IDEwLCBjb2xvdXIgPSAiYmxhY2siLCBoanVzdCA9IDAuNSwgYW5nbGUgPSA5MCkpDQpmaXQNCn0NCg0KdW5pX2VnZ2VyX3Bsb3RfdnI8LWZ1bmN0aW9uKG0sIGRhdGEpeyMgZ2V0dGluZyBtYXJnaW5hbCBSMg0KcjIgPC0gcjJfbWwobSkNCiMgZ2V0dGluZyBlc3RpbWF0ZXM6IG5hbWUgZG9lcyBub3Qgd29yayBmb3Igc2xvcGVzDQplc3QgPC0gZ2V0X2VzdChtLCBtb2QgPSAic3FydCh2aSkiKQ0KDQoNCiMgY3JlYXRpbmcgYSB0YWJsZQ0KdGliYmxlKGBGaXhlZCBlZmZlY3RgID0gcm93Lm5hbWVzKG0kYmV0YSksIEVzdGltYXRlID0gYyhlc3QkZXN0aW1hdGUpLCANCiAgICAgICBgTG93ZXIgQ0kgWzAuMDI1XWAgPSBjKGVzdCRsb3dlckNMKSwgYFVwcGVyIENJICBbMC45NzVdYCA9IGMoZXN0JHVwcGVyQ0wpLCANCiAgICAgICBgUCB2YWx1ZWAgPSBjKG0kcHZhbCksIFIyID0gYyhyMlsxXSwgDQogICAgICAgICAgICAgICAgICAgICAgICAgICAgICAgICAgICAgICAgICAgICAgICAgICAgICAgICAgICAgIE5BKSkgJT4lIGthYmxlKCJodG1sIiwgZGlnaXRzID0gMykgJT4lIGthYmxlX3N0eWxpbmcoInN0cmlwZWQiLCBwb3NpdGlvbiA9ICJsZWZ0IikNCg0KDQpwcmVkIDwtIHByZWRpY3Qucm1hKG0pDQoNCg0KDQojIHBsb3R0aW5nDQpmaXQgPC0gZGF0YSAlPiUgZHJvcF9uYSh2aV92ciklPiUgbXV0YXRlKHltaW4gPSBwcmVkJGNpLmxiLCB5bWF4ID0gcHJlZCRjaS51YiwgeW1pbjIgPSBwcmVkJGNyLmxiLCB5bWF4MiA9IHByZWQkY3IudWIsIHByZWQgPSBwcmVkJHByZWQpICU+JSANCiAgZ2dwbG90KGFlcyh4ID0gc3FydCh2aV92ciksIHkgPSB5aV92ciwgc2l6ZSA9IHNxcnQoMS92aV92cikpKSArIGdlb21fcG9pbnQoc2hhcGUgPSAyMSwgZmlsbCA9ICJncmV5OTAiLCBhbHBoYSA9IDAuMykgKyANCiAgZ2VvbV9zbW9vdGgoYWVzKHkgPSB5bWluMiksIG1ldGhvZCA9ICJsb2VzcyIsIHNlID0gRkFMU0UsIGx0eSA9ICJkb3R0ZWQiLCBsd2QgPSAwLjI1LCBjb2xvdXIgPSAiIzAwNzJCMiIpICsgDQogIGdlb21fc21vb3RoKGFlcyh5ID0geW1heDIpLCBtZXRob2QgPSAibG9lc3MiLCBzZSA9IEZBTFNFLCBsdHkgPSAiZG90dGVkIiwgbHdkID0gMC4yNSwgY29sb3VyID0gIiMwMDcyQjIiKSArIA0KICBnZW9tX3Ntb290aChhZXMoeSA9IHltaW4pLCBtZXRob2QgPSAibG9lc3MiLCBzZSA9IEZBTFNFLCBsdHkgPSAiZG90dGVkIiwgbHdkID0gMC4yNSwgY29sb3VyID0gIiNENTVFMDAiKSArIA0KICBnZW9tX3Ntb290aChhZXMoeSA9IHltYXgpLCBtZXRob2QgPSAibG9lc3MiLCBzZSA9IEZBTFNFLCBsdHkgPSAiZG90dGVkIiwgbHdkID0gMC4yNSwgY29sb3VyID0gIiNENTVFMDAiKSArDQogIGdlb21fc21vb3RoKGFlcyh5ID0gcHJlZCksIG1ldGhvZCA9ICJsb2VzcyIsIHNlID0gRkFMU0UsIGx0eSA9ICJkYXNoZWQiLCBsd2QgPSAwLjUsIGNvbG91ciA9ICJibGFjayIpICsNCiAgbGFicyh4ID0gInNxcnQoc2FtcGxpbmcgdmFyaWFuY2UpIiwgeSA9ICJsblJSIChlZmZlY3Qgc2l6ZSkiLCBzaXplID0gIlByZWNpc2lvbiAoMS9TRSkiKSArIA0KICBndWlkZXMoZmlsbCA9ICJub25lIiwgY29sb3VyID0gIm5vbmUiKSArIA0KICB0aGVtZV9idygpICsgdGhlbWUobGVnZW5kLnBvc2l0aW9uID0gYygwLCAxKSwgbGVnZW5kLmp1c3RpZmljYXRpb24gPSBjKDAsIDEpKSArIHRoZW1lKGxlZ2VuZC5kaXJlY3Rpb24gPSAiaG9yaXpvbnRhbCIpICsgDQogIHRoZW1lKGxlZ2VuZC5iYWNrZ3JvdW5kID0gZWxlbWVudF9ibGFuaygpKSArIHRoZW1lKGF4aXMudGV4dC55ID0gZWxlbWVudF90ZXh0KHNpemUgPSAxMCwgY29sb3VyID0gImJsYWNrIiwgaGp1c3QgPSAwLjUsIGFuZ2xlID0gOTApKQ0KZml0DQp9DQoNCnVuaV9lZ2dlcl9wbG90X21lYW48LWZ1bmN0aW9uKG0sIGRhdGEpeyMgZ2V0dGluZyBtYXJnaW5hbCBSMg0KcjIgPC0gcjJfbWwobSkNCiMgZ2V0dGluZyBlc3RpbWF0ZXM6IG5hbWUgZG9lcyBub3Qgd29yayBmb3Igc2xvcGVzDQplc3QgPC0gZ2V0X2VzdChtLCBtb2QgPSAic3FydCh2aSkiKQ0KDQojIGNyZWF0aW5nIGEgdGFibGUNCnRpYmJsZShgRml4ZWQgZWZmZWN0YCA9IHJvdy5uYW1lcyhtJGJldGEpLCBFc3RpbWF0ZSA9IGMoZXN0JGVzdGltYXRlKSwgDQogICAgICAgYExvd2VyIENJIFswLjAyNV1gID0gYyhlc3QkbG93ZXJDTCksIGBVcHBlciBDSSAgWzAuOTc1XWAgPSBjKGVzdCR1cHBlckNMKSwgDQogICAgICAgYFAgdmFsdWVgID0gYyhtJHB2YWwpLCBSMiA9IGMocjJbMV0sIA0KICAgICAgICAgICAgICAgICAgICAgICAgICAgICAgICAgICAgICAgICAgICAgICAgICAgICAgICAgICAgICBOQSkpICU+JSBrYWJsZSgiaHRtbCIsIGRpZ2l0cyA9IDMpICU+JSBrYWJsZV9zdHlsaW5nKCJzdHJpcGVkIiwgcG9zaXRpb24gPSAibGVmdCIpDQoNCg0KcHJlZCA8LSBwcmVkaWN0LnJtYShtKQ0KDQoNCg0KIyBwbG90dGluZw0KZml0IDwtIGRhdGEgJT4lIGRyb3BfbmEodmlfbWVhbiklPiUgbXV0YXRlKHltaW4gPSBwcmVkJGNpLmxiLCB5bWF4ID0gcHJlZCRjaS51YiwgeW1pbjIgPSBwcmVkJGNyLmxiLCB5bWF4MiA9IHByZWQkY3IudWIsIHByZWQgPSBwcmVkJHByZWQpICU+JSANCiAgZ2dwbG90KGFlcyh4ID0gc3FydCh2aV9tZWFuKSwgeSA9IHlpX21lYW4sIHNpemUgPSBzcXJ0KDEvdmlfbWVhbikpKSArIGdlb21fcG9pbnQoc2hhcGUgPSAyMSwgZmlsbCA9ICJncmV5OTAiLCBhbHBoYSA9IDAuMykgKyANCiAgZ2VvbV9zbW9vdGgoYWVzKHkgPSB5bWluMiksIG1ldGhvZCA9ICJsb2VzcyIsIHNlID0gRkFMU0UsIGx0eSA9ICJkb3R0ZWQiLCBsd2QgPSAwLjI1LCBjb2xvdXIgPSAiIzAwNzJCMiIpICsgDQogIGdlb21fc21vb3RoKGFlcyh5ID0geW1heDIpLCBtZXRob2QgPSAibG9lc3MiLCBzZSA9IEZBTFNFLCBsdHkgPSAiZG90dGVkIiwgbHdkID0gMC4yNSwgY29sb3VyID0gIiMwMDcyQjIiKSArIA0KICBnZW9tX3Ntb290aChhZXMoeSA9IHltaW4pLCBtZXRob2QgPSAibG9lc3MiLCBzZSA9IEZBTFNFLCBsdHkgPSAiZG90dGVkIiwgbHdkID0gMC4yNSwgY29sb3VyID0gIiNENTVFMDAiKSArIA0KICBnZW9tX3Ntb290aChhZXMoeSA9IHltYXgpLCBtZXRob2QgPSAibG9lc3MiLCBzZSA9IEZBTFNFLCBsdHkgPSAiZG90dGVkIiwgbHdkID0gMC4yNSwgY29sb3VyID0gIiNENTVFMDAiKSArDQogIGdlb21fc21vb3RoKGFlcyh5ID0gcHJlZCksIG1ldGhvZCA9ICJsb2VzcyIsIHNlID0gRkFMU0UsIGx0eSA9ICJkYXNoZWQiLCBsd2QgPSAwLjUsIGNvbG91ciA9ICJibGFjayIpICsNCiAgbGFicyh4ID0gInNxcnQoc2FtcGxpbmcgdmFyaWFuY2UpIiwgeSA9ICJsblJSIChlZmZlY3Qgc2l6ZSkiLCBzaXplID0gIlByZWNpc2lvbiAoMS9TRSkiKSArIA0KICBndWlkZXMoZmlsbCA9ICJub25lIiwgY29sb3VyID0gIm5vbmUiKSArIA0KICB0aGVtZV9idygpICsgdGhlbWUobGVnZW5kLnBvc2l0aW9uID0gYygwLCAxKSwgbGVnZW5kLmp1c3RpZmljYXRpb24gPSBjKDAsIDEpKSArIHRoZW1lKGxlZ2VuZC5kaXJlY3Rpb24gPSAiaG9yaXpvbnRhbCIpICsgDQogIHRoZW1lKGxlZ2VuZC5iYWNrZ3JvdW5kID0gZWxlbWVudF9ibGFuaygpKSArIHRoZW1lKGF4aXMudGV4dC55ID0gZWxlbWVudF90ZXh0KHNpemUgPSAxMCwgY29sb3VyID0gImJsYWNrIiwgaGp1c3QgPSAwLjUsIGFuZ2xlID0gOTApKQ0KZml0DQp9DQoNCg0KDQpJMiA8LSBmdW5jdGlvbihtb2RlbCwgbWV0aG9kID0gYygiV29sZmdhbmciLCAiU2hpbmljaGkiKSkgew0KICAgIA0KICAgICMjIGV2YWx1YXRlIGNob2ljZXMNCiAgICBtZXRob2QgPC0gbWF0Y2guYXJnKG1ldGhvZCkNCiAgICANCiAgICAjIFdvbGZnYW5nJ3MgbWV0aG9kDQogICAgaWYgKG1ldGhvZCA9PSAiV29sZmdhbmciKSB7DQogICAgICAgIFcgPC0gc29sdmUobW9kZWwkVikNCiAgICAgICAgWCA8LSBtb2RlbC5tYXRyaXgobW9kZWwpDQogICAgICAgIFAgPC0gVyAtIFcgJSolIFggJSolIHNvbHZlKHQoWCkgJSolIFcgJSolIFgpICUqJSB0KFgpICUqJSBXDQogICAgICAgIEkyX3RvdGFsIDwtIHN1bShtb2RlbCRzaWdtYTIpLyhzdW0obW9kZWwkc2lnbWEyKSArIChtb2RlbCRrIC0gbW9kZWwkcCkvc3VtKGRpYWcoUCkpKQ0KICAgICAgICBJMl9lYWNoIDwtIG1vZGVsJHNpZ21hMi8oc3VtKG1vZGVsJHNpZ21hMikgKyAobW9kZWwkayAtIG1vZGVsJHApL3N1bShkaWFnKFApKSkNCiAgICAgICAgbmFtZXMoSTJfZWFjaCkgPSBwYXN0ZTAoIkkyXyIsIG1vZGVsJHMubmFtZXMpDQogICAgICAgIA0KICAgICAgICAjIHB1dHRpbmcgYWxsIHRvZ2V0aGVyDQogICAgICAgIEkycyA8LSBjKEkyX3RvdGFsID0gSTJfdG90YWwsIEkyX2VhY2gpDQogICAgICAgIA0KICAgICAgICAjIG9yIG15IHdheQ0KICAgIH0gZWxzZSB7DQogICAgICAgICMgc2lnbWEyX3YgPSB0eXBpY2FsIHNhbXBsaW5nIGVycm9yIHZhcmlhbmNlDQogICAgICAgIHNpZ21hMl92IDwtIHN1bSgxL21vZGVsJHZpKSAqIChtb2RlbCRrIC0gMSkvKHN1bSgxL21vZGVsJHZpKV4yIC0gc3VtKCgxL21vZGVsJHZpKV4yKSkNCiAgICAgICAgSTJfdG90YWwgPC0gc3VtKG1vZGVsJHNpZ21hMikvKHN1bShtb2RlbCRzaWdtYTIpICsgc2lnbWEyX3YpICAjc14yX3QgPSB0b3RhbCB2YXJpYW5jZQ0KICAgICAgICBJMl9lYWNoIDwtIG1vZGVsJHNpZ21hMi8oc3VtKG1vZGVsJHNpZ21hMikgKyBzaWdtYTJfdikNCiAgICAgICAgbmFtZXMoSTJfZWFjaCkgPSBwYXN0ZTAoIkkyXyIsIG1vZGVsJHMubmFtZXMpDQogICAgICAgIA0KICAgICAgICAjIHB1dHRpbmcgYWxsIHRvZ2V0aGVyDQogICAgICAgIEkycyA8LSBjKEkyX3RvdGFsID0gSTJfdG90YWwsIEkyX2VhY2gpDQogICAgfQ0KICAgIHJldHVybihJMnMpDQp9DQoNCg0KYGBgDQoNClRoZSBtZXRhLWFuYWx5dGljIGRhdGFzZXQgaXMgYXZhaWxhYmxlIGF0IDxodHRwczovL29zZi5pby80YXVjcC8+Lg0KDQojIyBMaXRlcmF0dXJlIHNlYXJjaA0KDQpUaGUgY29yZSBkZXRhaWxzIG9mIHRoZSBsaXRlcmF0dXJlIHNlYXJjaCBhcmUgaW5jbHVkZWQgaW4gdGhlIG1haW4gYm9keSBvZiB0aGUgbWFudXNjcmlwdCBzbyBhcmUgbm90IGluY2x1ZGVkIGhlcmUuIEhvd2V2ZXIsIHRoZXJlIGFyZSBzb21lIGFkZGl0aW9uYWwgZGV0YWlscyB0aGF0IGFyZSBpbXBvcnRhbnQgdG8gbm90ZS4NCg0KVGl0bGVzIG9mIHJlc3VsdHMgd2VyZSBzY3JlZW5lZCBmb3IgYW55IG5vbi10ZXJyZXN0cmlhbCBzdHVkaWVzIGJ5IHJlbW92YWwgb2YgdGl0bGVzIGNvbnRhaW5pbmcgdGhlIGZvbGxvd2luZyB3b3JkczogYXF1YXRpYywgc3RyZWFtLCByaXZlciwgbWFyaW5lLCBvY2VhbiwgbGFrZSwgZmlzaCwgd2V0bGFuZCwgc2FsdG1hcnNoLCBwb25kLCBjb3JhbCwgcmVlZiwgcGxhbmt0b24uIE90aGVyIHVzZWZ1bCBibGFua2V0IHNjcmVlbmluZyB0ZXJtcyBpbmNsdWRlLCAicHJpb3JpdGl6XCoiLCAiaW1wbGljYXRpb25zIGZvciIsIHdoaWNoIGhlbHBlZCB0byBpZGVudGlmeSBlbXBpcmljYWwgc3R1ZGllcyBub3Qgb2YgcmVzdG9yYXRpb24gc2l0ZXMgYnV0IG9mIGV4cGVyaW1lbnRzIHdpdGggcG90ZW50aWFsIGltcGxpY2F0aW9ucyBmb3IgZWNvbG9naWNhbCByZXN0b3JhdGlvbi4gVGhpcyByZW1vdmVkIDU1NSByZWNvcmRzIGZvciBhIHRvdGFsIG9mIDE3MjEgc3R1ZGllcyByZW1haW5pbmcuIFRpdGxlcyB3ZXJlIHRoZW4gaW5kaXZpZHVhbGx5IHNjcmVlbmVkIGluIGRldGFpbCB0byBvbmx5IGluY2x1ZGUgc3R1ZGllcyB0aGF0IGNsZWFybHkgYXNzZXNzZWQgcmVzdG9yYXRpb24gb3V0Y29tZXMgdXNpbmcgc29tZSBmb3JtIG9mIGJpb2RpdmVyc2l0eS4gV2hlcmUgc3R1ZHkgdGl0bGVzIHdlcmUgYW1iaWd1b3VzIHRoZXkgd2VyZSBub3QgcmVtb3ZlZC4gT25seSBvYnZpb3VzbHkgaXJyZWxldmFudCBzdHVkaWVzIHdlcmUgcmVtb3ZlZCBhdCB0aGlzIHN0YWdlLiBUaGlzIHJlbW92ZWQgYSBmdXJ0aGVyIDExMzcgc3R1ZGllcy4NCg0KQWxsIHNjcmVlbmluZyB3YXMgY29uZHVjdGVkIGJ5IGxlYWQgYXV0aG9yIEpBLg0KDQpXZSBkaWQgbm90IHJlY29yZCBhIG1lYXN1cmUgb2YgdGhlIGluZGl2aWR1YWwgcXVhbGl0eSBvZiBzdHVkaWVzIGluY2x1ZGVkIGluIHRoZSBtZXRhLWFuYWx5c2lzIChlLmcuIGJsaW5kZWQgZGF0YSBjb2xsZWN0aW9uLCByZXBvcnRpbmcgcXVhbGl0eSwgYW5kIGV4cGVyaW1lbnRhbCB2cyBvYnNlcnZhdGlvbmFsKS4NCg0KU2VlIGJlbG93IGZvciBhIFBSSVNNQSBkaWFncmFtIHNob3dpbmcgdGhlIHdvcmtmbG93IGZvciB0aGUgbGl0ZXJhdHVyZSBzZWFyY2guDQoNCiFbXShDOi9Vc2Vycy9Kb2UvT25lRHJpdmUgLSBVTlNXL0Rlc2t0b3AvUGhEL01ldGEtYW5hbHlzaXMvRGF0YS9wcmlzbWEuanBnKQ0KDQoNCioqRmlndXJlIFMxLioqIFBSSVNNQSBkaWFncmFtIGRldGFpbGluZyBsaXRlcmF0dXJlIHNjcmVlbmluZyBwcm9jZXNzIChmb3JtYXR0ZWQgYWNjb3JkaW5nIHRvIFBhZ2UgZXQgYWwuIDIwMjApDQoNCiMjIE1ldGEtYW5hbHlzaXM6IHRoZSBlZmZlY3Qgb2YgcmVzdG9yYXRpb24gb24gdmFyaWFiaWxpdHkgaW4gYmlvZGl2ZXJzaXR5DQoNCiMjIyBDaG9vc2luZyBlZmZlY3Qgc2l6ZSBzdGF0aXN0aWNzOiBjaGVja2luZyB0aGUgbWVhbi12YXJpYW5jZSByZWxhdGlvbnNoaXANCg0KV2UgY2hlY2tlZCB0aGUgbWVhbi12YXJpYW5jZSByZWxhdGlvbnNoaXAgaW4gb3VyIGRhdGEuIElmIHRoZXJlIGlzIHN1Y2ggYSByZWxhdGlvbnNoaXAsIGl0IGlzIGJldHRlciB0byB1c2UgdGhlIGxvZ2FyaXRobSBvZiByZXNwb25zZSByYXRpbywgbG5SUiByYXRoZXIgdGhhbiBzdGFuZGFyZGl6ZWQgbWVhbiBkaWZmZXJlbmNlIChvZnRlbiBrbm93biBhcyBDb2hlbidzICpkKiBvciBIZWRnZXMnICpnKikgYmVjYXVzZSB0aGUgbGF0dGVyIGFzc3VtZXMgdGhlIGhvbW9nZW5laXR5IG9mIHZhcmlhbmNlLg0KDQpgYGB7ciBmaWcud2lkdGg9NywgZmlnLmhlaWdodD0xMX0NCg0KZnVsbF9kYXRhPC1yZWFkLmNzdigiRGF0YS92YXJpYXRpb25fZGF0YS5jc3YiLCBzdHJpbmdzQXNGYWN0b3JzPUZBTFNFKQ0KDQojIEEpDQpkYXRfdDwtZnVsbF9kYXRhICU+JSBkcm9wX25hKHRfbWVhbikgIyBzb21lIGhhdmUgTkEgdmFsdWVzIGhlcmUgc28gbG9nIHRyYW5zZm9ybSBwcm9kdWNlcyBOQXMsIGNyZWF0aW5nIGNsZWFuIGRhdGFzZXQgZm9yIGVhY2ggcGxvdA0KZGF0X2M8LWZ1bGxfZGF0YSAlPiUgZHJvcF9uYShjX21lYW4pICU+JSBmaWx0ZXIoY19zZCAhPSAwKSAjIHQgPSB0cmVhdG1lbnQgKHJlc3RvcmVkKSwgYyA9IGNvbnRyb2wgKHVucmVzdG9yZWQpLCByID0gcmVmZXJlbmNlIChzb21lIFNEID0gMCBpbiBjb250cm9sIGRhdGFzZXQpDQpkYXRfcjwtZnVsbF9kYXRhICU+JSBkcm9wX25hKHJfbWVhbikNCg0KY29yXzEgPC0gcm91bmQod2l0aChkYXRfdCxjb3IobG9nKHRfbWVhbiksIGxvZyh0X3NkKSkpLCAzKQ0KcGxvdF9yZXMgPC0gZ2dwbG90KGRhdF90LCBhZXMobG9nKHRfbWVhbiksIGxvZyh0X3NkKSkpICsgZ2VvbV9wb2ludCgpICsNCiAgZ2VvbV9zbW9vdGgobWV0aG9kID0gImxtIikgKyANCiAgbGFicyh4ID0gImxuKG1lYW5bZXhwZXJpbWVudF0pIiwgDQogICAgICAgeSA9ICJsbihTRFtleHBlcmltZW50XSkiLCANCiAgICAgICB0aXRsZSA9ICJSZXN0b3JhdGlvbiBzaXRlcyBtZWFuIHZzIHZhcmlhbmNlIChzZCkiKSArDQogIHhsaW0oLTUsIDcuNSkgKyB5bGltKC05LCA5KSArIGFubm90YXRlKCd0ZXh0Jyx4ID0gNy41LCB5ID0gLTgsIGxhYmVsID0gcGFzdGUoInIgPSAiLCBjb3JfMSkpDQoNCiMgQikNCmNvcl8yIDwtIHJvdW5kKHdpdGgoZGF0X2MsY29yKGxvZyhjX21lYW4pLCBsb2coY19zZCkpKSwgMykNCnBsb3RfY29uIDwtIGdncGxvdChkYXRfYywgYWVzKGxvZyhjX21lYW4pLCBsb2coY19zZCkpKSArIGdlb21fcG9pbnQoKSArDQogIGdlb21fc21vb3RoKG1ldGhvZCA9ICJsbSIpICsgDQogIGxhYnMoeCA9ICJsbihtZWFuW2NvbnRyb2xdKSIsIA0KICAgICAgIHkgPSAibG4oU0RbY29udHJvbF0pIiwNCiAgICAgICB0aXRsZSA9ICJVbnJlc3RvcmVkIHNpdGVzIG1lYW4gdnMgdmFyaWFuY2UgKHNkKSIpKw0KICB4bGltKC01LCA3LjUpICsgeWxpbSgtOSwgOSkgKyBhbm5vdGF0ZSgndGV4dCcseCA9IDcuNSwgeSA9IC04LCBsYWJlbCA9IHBhc3RlKCJyID0gIiwgY29yXzIpKQ0KDQojIGMpDQpjb3JfMyA8LSByb3VuZCh3aXRoKGRhdF9yLGNvci50ZXN0KGxvZyhyX21lYW4pLCBsb2cocl9zZCkpKSRlc3RpbWF0ZVtbMV1dLCAzKQ0KcGxvdF9yZWYgPC0gZ2dwbG90KGRhdF9yLCBhZXMobG9nKHJfbWVhbiksIGxvZyhyX3NkKSkpICsgZ2VvbV9wb2ludCgpICsNCiAgZ2VvbV9zbW9vdGgobWV0aG9kID0gImxtIikgKyANCiAgbGFicyh4ID0gImxuKG1lYW5bZXhwZXJpbWVudF0pIiwgDQogICAgICAgeSA9ICJsbihTRFtleHBlcmltZW50XSkiLA0KICAgICAgIHRpdGxlID0gIlJlZmVyZW5jZSBzaXRlcyBtZWFuIHZzIHZhcmlhbmNlIChzZCkiKSsNCiAgeGxpbSgtNSwgNy41KSArIHlsaW0oLTksIDkpICsgYW5ub3RhdGUoJ3RleHQnLHggPSA3LjUsIHkgPSAtOCwgbGFiZWwgPSBwYXN0ZSgiciA9ICIsIGNvcl8zKSkNCg0KDQptZWFuX1NEIDwtIChwbG90X3JlcyAvIHBsb3RfY29uIC8gcGxvdF9yZWYpICsNCiAgcGxvdF9hbm5vdGF0aW9uKHRhZ19sZXZlbHMgPSAiQSIsIHRhZ19zdWZmaXggPSAiKSIpDQoNCm1lYW5fU0QNCg0KDQpgYGANCg0KDQoNCioqRmlndXJlIFMyOioqIENvcnJlbGF0aW9ucyBiZXR3ZWVuIG1lYW4gYW5kIHZhcmlhbmNlIGluIHRoZSByZXN0b3JlZCBzaXRlcywgdW5yZXN0b3JlZCBzaXRlcywgYW5kIHJlZmVyZW5jZSBzaXRlcy4NCg0KIyMjIENhbGN1bGF0aW5nIGVmZmVjdCBzaXplcw0KDQpXZSBmb3VuZCBleHRyZW1lbHkgc3Ryb25nIGNvcnJlbGF0aW9ucyBiZXR3ZWVuIG1lYW4gYW5kIHZhcmlhbmNlIChzdGFuZGFyZCBkZXZpYXRpb24pIG9uIHRoZSBsb2cgc2NhbGUgYWJvdmUsIHNvIGluc3RlYWQsIHdlIHJlcG9ydCBkaWZmZXJlbmNlcyBpbiB2YXJpYWJpbGl0eSB3aXRoaW4tc3R1ZGllcyBhcyB0aGUgZGlmZmVyZW5jZSBpbiBsbkNWUiAodGhlIGxvZyBvZiB0aGUgY29lZmZpY2llbnQgb2YgdmFyaWF0aW9uIHJhdGlvLiBXZSB1c2UgdGhlIGxvZyByZXNwb25zZSByYXRpbyB0byBjb21wYXJlIG1lYW4gZGlmZmVyZW5jZXMgZm9yIGEgcmFuZ2Ugb2YgcmVhc29ucyBvdXRsaW5lZCBpbiB0aGUgbWV0aG9kcyBvZiB0aGUgbWFpbiBib2R5IG9mIHRoZSBwYXBlciwgYW5kIGFzIGlzIGlsbHVzdHJhdGVkIGZ1cnRoZXIgYmVsb3csIGl0IGlzIGFsc28gbGVzcyBzZW5zaXRpdmUgdG8gc2NhbGUgYmlhcy4NCg0KRWZmZWN0IHNpemVzIGFyZSBjYWxjdWxhdGVkIHVzaW5nIGBlc2NhbGNgIGZ1bmN0aW9uIGluIGBtZXRhZm9yYC4gSGVyZSB3ZSBjYWxjdWxhdGUgZWZmZWN0IHNpemVzIGZvciB0d28gc2VwYXJhdGUgbWV0YS1hbmFseXNlcywgY29tcGFyaW5nIHJlc3RvcmVkIHNpdGVzIHRvIGNvbnRyb2wgKHVucmVzdG9yZWQpIHNpdGVzLCBhbmQgY29tcGFyaW5nIHJlc3RvcmVkIHNpdGVzIHRvIHJlZmVyZW5jZSAoZ29hbCkgc2l0ZXMuIFdlIGFsc28gdXNlIHRoZSBgbWFrZV9WQ1ZfbWF0cml4YCBmdW5jdGlvbiB0byBjYWxjdWxhdGUgdGhlIHZhcmlhbmNlLWNvdmFyaWFuY2UgbWF0cml4IHRvIHVzZSBpbiB0aGUgbW9kZWwgaW4gcGxhY2Ugb2YgdGhlIGVycm9yIHRlcm0gYHZpYC4gVGhlIGNvZGUgZXNzZW50aWFsbHkgcGVyZm9ybXMgdGhlIHNhbWUgcHJvY2VkdXJlIGZvciBjYWx1Y2xhdGluZyBlZmZlY3Qgc2l6ZXMgdHdpY2UuIFdlIGFsc28gY2FsY3VsYXRlIGxuVlIgKHRoZSBsb2cgdmFyaWFiaWxpdHkgcmF0aW8gdGhhdCB1c2VzIFNEKSwgZGVzcGl0ZSB0aGUgY29ycmVsYXRpb25zIHNob3duIGFib3ZlLCB0byBwcmVzZW50IGFsb25nc2lkZSB0aGUgbWFpbiByZXN1bHRzICh0aG91Z2ggdGhlc2UgYXJlIG5vdCBwcmVzZW50ZWQgaW4gdGhlIGJvZHkgb2YgdGhlIHBhcGVyKS4NCg0KYGBge3J9DQoNCiMjIyMjIyMjIyMjIyMjIyMjIyMjIyMjIyMjIw0KIyMgVU5SRVNUT1JFRCAvIFJFU1RPUkVEICMjDQojIyMjIyMjIyMjIyMjIyMjIyMjIyMjIyMjIyMNCg0KIyB1bl9yZSA9IHVucmVzdG9yZWQgLyByZXN0b3JlZCANCg0KdW5fcmU8LXJlYWQuY3N2KCJEYXRhL3ZhcmlhdGlvbl9kYXRhLmNzdiIsIHN0cmluZ3NBc0ZhY3RvcnMgPSBGKQ0KDQp1bl9yZSRjX3F1YWRfbiA9IGFzLm51bWVyaWModW5fcmUkY19xdWFkX24pDQp1bl9yZSRjX21lYW4gPSAgYXMubnVtZXJpYyh1bl9yZSRjX21lYW4pDQp1bl9yZSRjX3NkID0gYXMubnVtZXJpYyh1bl9yZSRjX3NkKQ0KDQojcmVtb3ZlIHN0dWRpZXMgd2l0aCBvbmx5IGEgcmVzdG9yZWQgc2l0ZXMgY29tcGFyaXNvbg0KdW5fcmU8LXVuX3JlWyFpcy5uYSh1bl9yZSRjX21lYW4pLF0NCiN1bl9yZSAlPiUgZ3JvdXBfYnkoaWQsIGNfbWVhbiwgY19zZCkgJT4lIGRpc3RpbmN0KHNoYXJlZF9jdHJsKSAlPiUgZmlsdGVyKG4oKT4xKSAjIGNoZWNraW5nIHNoYXJlZCBjb250cm9scyBpcyBhY2N1cmF0ZQ0KDQojY2FsY3VsYXRlIHRoZSBsbkNWUiBhbmQgbG5SUiBhbmQgbG5WUiBlZmZlY3Qgc2l6ZSBhbmQgdW5fcmVpYW5jZSB3aXRoIGVzY2FsYw0KQ1ZSPC1lc2NhbGMobWVhc3VyZSA9ICJDVlIiLCBuMWkgPSB1bl9yZSR0X3F1YWRfbiwgbjJpID0gdW5fcmUkY19xdWFkX24sIG0xaSA9IHVuX3JlJHRfbWVhbiwgbTJpID0gdW5fcmUkY19tZWFuLCBzZDFpID0gdW5fcmUkdF9zZCwgc2QyaSA9IHVuX3JlJGNfc2QpDQpsblJSPC1lc2NhbGMobWVhc3VyZSA9ICJST00iLCBuMWkgPSB1bl9yZSR0X3F1YWRfbiwgbjJpID0gdW5fcmUkY19xdWFkX24sIG0xaSA9IHVuX3JlJHRfbWVhbiwgbTJpID0gdW5fcmUkY19tZWFuLCBzZDFpID0gdW5fcmUkdF9zZCwgc2QyaSA9IHVuX3JlJGNfc2QpDQpsblZSPC1lc2NhbGMobWVhc3VyZSA9ICJWUiIsIG4xaSA9IHVuX3JlJHRfcXVhZF9uLCBuMmkgPSB1bl9yZSRjX3F1YWRfbiwgbTFpID0gdW5fcmUkdF9tZWFuLCBtMmkgPSB1bl9yZSRjX21lYW4sIHNkMWkgPSB1bl9yZSR0X3NkLCBzZDJpID0gdW5fcmUkY19zZCkNCg0KI2NvbWJpbmVkIGVmZmVjdCBzaXplcyB3aXRoIHJlbGV2YW50IHVuX3JlYSBmcmFtZXMNCnVuX3JlIDwtYmluZF9jb2xzKHVuX3JlLCBsblJSLCBsblZSLCBDVlIpDQoNCiMgbmFtZSB0aGUgdW5fcmVhIHNvbWV0aGluZyBtZWFuaW5nZnVsIGFuZCByZW1vdmUgYWxsIHRoZSBjb2x1bW5zIHVubmVlZGVkDQp1bl9yZTwtdW5fcmUgJT4lIHJlbmFtZSh5aV9tZWFuID0geWkuLi4zNiwgdmlfbWVhbiA9IHZpLi4uMzcsIHlpX3ZyID0geWkuLi4zOCwgdmlfdnIgPSB2aS4uLjM5LCB5aV9jdnIgPSB5aS4uLjQwLCB2aV9jdnIgPSB2aS4uLjQxKQ0KDQojcmVtb3ZlIHN0dWRpZXMgdGhhdCBoYXZlIHZpPU5BIC0gdXN1YWxseSB3aGVyZSBjb250cm9sIFNEID0gMA0KdW5fcmU8LXVuX3JlWyFpcy5uYSh1bl9yZSR2aV92ciksXQ0KDQp1bl9yZSRwbHU8LWFzLmZhY3Rvcih1bl9yZSRwbHUpDQp1bl9yZSRwbHU8LXJlbGV2ZWwodW5fcmUkcGx1LCAic2VtaS1uYXR1cmFsIikNCg0KI25lZWQgYW5vdGhlciByYW5kb20gZmFjdG9yIGZvciAndW5pdCcNCg0KdW5pdCA8LSBmYWN0b3IoMTpsZW5ndGgodW5fcmUkeWlfbWVhbikpDQp1bl9yZSR1bml0IDwtIHVuaXQNCg0KdmN2X2N2cjwtbWFrZV9WQ1ZfbWF0cml4KHVuX3JlLCBWID0idmlfY3ZyIiwgInNoYXJlZF9jdHJsIiwgInVuaXQiLCByaG89MC41KQ0KdmN2X21lYW48LW1ha2VfVkNWX21hdHJpeCh1bl9yZSwgViA9InZpX21lYW4iLCAic2hhcmVkX2N0cmwiLCAidW5pdCIsIHJobz0wLjUpDQp2Y3ZfdnI8LW1ha2VfVkNWX21hdHJpeCh1bl9yZSwgViA9InZpX3ZyIiwgInNoYXJlZF9jdHJsIiwgInVuaXQiLCByaG89MC41KQ0KDQoNCg0KIyMjIyMjIyMjIyMjIyMjIyMjIyMjIyMjIyMNCiMjIFJFU1RPUkVEIC8gUkVGRVJFTkNFICMjDQojIyMjIyMjIyMjIyMjIyMjIyMjIyMjIyMjIw0KDQojIHJlX3JlZiA9IHJlc3RvcmVkIC8gcmVmZXJlbmNlIA0KDQpyZV9yZWY8LXJlYWQuY3N2KCJEYXRhL3ZhcmlhdGlvbl9kYXRhLmNzdiIsIHN0cmluZ3NBc0ZhY3RvcnMgPSBGKQ0KDQojcmVtb3ZlIHN0dWRpZXMgd2l0aCBvbmx5IGEgZGVncmFkZWQgc2l0ZSBjb21wYXJpc29uDQpyZV9yZWY8LXJlX3JlZlshaXMubmEocmVfcmVmJHJfbWVhbiksXQ0KDQojcmVtb3ZlIHN0dWRpZXMgdGhhdCBoYXZlIHZpPU5BIC0gdXN1YWxseSB3aGVyZSBjb250cm9sIFNEID0gMA0KcmVfcmVmPC1yZV9yZWYgJT4lIGZpbHRlcihyX3NkICE9IDApDQpyZV9yZWY8LXJlX3JlZiAlPiUgZmlsdGVyKCFpcy5uYShyX3NkKSkNCg0KIyB0aGVyZSBpcyBhIGZldyBzaXRlcyB3aGVyZSB0aGUgcmVmZXJlbmNlIGNvbnRyb2wgaXMgc2hhcmVkLCBidXQgdGhlIGRlZ3JhZGVkIG9uZSBpcyBub3QsIG5lZWQgdG8gYWRkIGEgInJlZl9zaGFyZWRfY3RybCIgdG8gY29ycmVjdCB0aGlzDQpyZV9yZWY8LXJlX3JlZiAlPiUgZ3JvdXBfYnkoaWQsIHJfbWVhbiwgcl9zZCkgJT4lIG11dGF0ZShyZWZfc2hhcmVkX2N0cmwgPSBjdXJfZ3JvdXBfaWQoKSkNCiNyZV9yZWYgJT4lIGdyb3VwX2J5KGlkLCByX21lYW4sIHJfc2QpICU+JSBkaXN0aW5jdChyZWZfc2hhcmVkX2N0cmwpICU+JSBmaWx0ZXIobigpPjEpICMgdG8gY2hlY2sgYW55IGVycm9ycyBpbiB0aGUgc2hhcmVkX2NvbnRyb2wgdGFnZ2luZw0KDQoNCg0KcmVfcmVmJHJfcXVhZF9uID0gYXMubnVtZXJpYyhyZV9yZWYkcl9xdWFkX24pDQpyZV9yZWYkcl9tZWFuID0gIGFzLm51bWVyaWMocmVfcmVmJHJfbWVhbikNCnJlX3JlZiRyX3NkID0gYXMubnVtZXJpYyhyZV9yZWYkcl9zZCkNCg0KcmVfcmVmPC1yZV9yZWYgJT4lIGZpbHRlcihyX3F1YWRfbiA+IDEpICMgYSBmZXcgc2FtcGxlIHNpemVzIG9mIDEgb3IgMD8NCg0KDQojY2FsY3VsYXRlIHRoZSBsbkNWUiBhbmQgbG5SUiBlZmZlY3Qgc2l6ZSBhbmQgcmVfcmVmaWFuY2Ugd2l0aCBlc2NhbGMNCkNWUjwtZXNjYWxjKG1lYXN1cmUgPSAiQ1ZSIiwgbjFpID0gcmVfcmVmJHRfcXVhZF9uLCBuMmkgPSByZV9yZWYkcl9xdWFkX24sIG0xaSA9IHJlX3JlZiR0X21lYW4sIG0yaSA9IHJlX3JlZiRyX21lYW4sIHNkMWkgPSByZV9yZWYkdF9zZCwgc2QyaSA9IHJlX3JlZiRyX3NkKQ0KbG5SUjwtZXNjYWxjKG1lYXN1cmUgPSAiUk9NIiwgbjFpID0gcmVfcmVmJHRfcXVhZF9uLCBuMmkgPSByZV9yZWYkcl9xdWFkX24sIG0xaSA9IHJlX3JlZiR0X21lYW4sIG0yaSA9IHJlX3JlZiRyX21lYW4sIHNkMWkgPSByZV9yZWYkdF9zZCwgc2QyaSA9IHJlX3JlZiRyX3NkKQ0KbG5WUjwtZXNjYWxjKG1lYXN1cmUgPSAiVlIiLCBuMWkgPSByZV9yZWYkdF9xdWFkX24sIG4yaSA9IHJlX3JlZiRyX3F1YWRfbiwgbTFpID0gcmVfcmVmJHRfbWVhbiwgbTJpID0gcmVfcmVmJHJfbWVhbiwgc2QxaSA9IHJlX3JlZiR0X3NkLCBzZDJpID0gcmVfcmVmJHJfc2QpDQoNCg0KI2NvbWJpbmVkIGVmZmVjdCBzaXplcyB3aXRoIHJlbGV2YW50IGRhdGEgZnJhbWVzDQpyZV9yZWYgPC1iaW5kX2NvbHMocmVfcmVmLCBsblJSLCBsblZSLCBDVlIpDQojIG5hbWUgdGhlIGRhdGEgc29tZXRoaW5nIG1lYW5pbmdmdWwgYW5kIHJlbW92ZSBhbGwgdGhlIGNvbHVtbnMgdW5uZWVkZWQNCnJlX3JlZjwtcmVfcmVmICU+JSByZW5hbWUoeWlfbWVhbiA9IHlpLi4uMzcsIHZpX21lYW4gPSB2aS4uLjM4LCB5aV92ciA9IHlpLi4uMzksIHZpX3ZyID0gdmkuLi40MCwgeWlfY3ZyID0geWkuLi40MSwgdmlfY3ZyID0gdmkuLi40MikNCg0KDQpyZV9yZWYkcGx1PC1hcy5mYWN0b3IocmVfcmVmJHBsdSkNCnJlX3JlZiRwbHU8LXJlbGV2ZWwocmVfcmVmJHBsdSwgInNlbWktbmF0dXJhbCIpDQoNCiNuZWVkIGFub3RoZXIgcmFuZG9tIGZhY3RvciBmb3IgJ3VuaXQnDQoNCnVuaXQgPC0gZmFjdG9yKDE6bGVuZ3RoKHJlX3JlZiR5aV9tZWFuKSkNCnJlX3JlZiR1bml0IDwtIHVuaXQNCg0KcmVfcmVmPC1hcy5kYXRhLmZyYW1lKHJlX3JlZikgIyB0aGUgZ3JvdXBfYnkgdG8gZG8gdGhlIHNoYXJlZCBjb250cm9sIGNoZWNrIGFib3ZlIHR1cm5zIHRoaXMgYmFkIGJveSBpbnRvIGEgdGliYmxlLCBuZWVkcyB0byBiZSBhIGRhdGFmcmFtZSBmb3IgdGhlIGJlbG93IGZ1bmN0aW9uDQoNCnZjdl9jdnJfcnI8LW1ha2VfVkNWX21hdHJpeChkYXRhID0gcmVfcmVmLCBWID0idmlfY3ZyIiwgY2x1c3RlciA9ICJyZWZfc2hhcmVkX2N0cmwiLCBvYnMgPSAidW5pdCIsIHJobz0wLjUpDQp2Y3ZfbWVhbl9ycjwtbWFrZV9WQ1ZfbWF0cml4KHJlX3JlZiwgViA9InZpX21lYW4iLCAicmVmX3NoYXJlZF9jdHJsIiwgInVuaXQiLCByaG89MC41KQ0KdmN2X3ZyX3JyPC1tYWtlX1ZDVl9tYXRyaXgocmVfcmVmLCBWID0idmlfdnIiLCAicmVmX3NoYXJlZF9jdHJsIiwgInVuaXQiLCByaG89MC41KQ0KDQoNCmBgYA0KDQojIyBNZXRhLWFuYWx5dGljIG1vZGVsczogbG5DVlIsIGxuVlIgYW5kIGxuUlINCg0KV2UgY29uZHVjdGVkIG1ldGEtYW5hbHlzZXMgKGkuZS4gcmFuIHRoZSBpbnRlcmNlcHQgbW9kZWxzKSB1c2luZyB0aGUgYHJtYS5tdmAgZnVuY3Rpb24gaW4gYG1ldGFmb3JgLiBGb3IgZXZlcnkgbW9kZWwgKGxuQ1ZSLCBsblJSLCBhbmQgbG5WUiksIHdlIGNvbmR1Y3QgdGhlIHNhbWUgbW9kZWwgYSBzZWNvbmQgdGltZSBpbmNsdWRpbmcgdGhlIHNhbXBsaW5nIHNjYWxlIChtZWFzdXJlZCBhcyBxdWFkcmF0IHNpemUpIHRvIHNlZSBpZiB0aGUgcmVzdWx0cyBhcmUgcm9idXN0IHRvIHZhcmlhdGlvbiBpbiBzYW1wbGluZyBzY2FsZS4gTm90ZSB0aGF0IHRoaXMgcmVkdWNlcyB0aGUgc2FtcGxlIHNpemUgb2YgdGhlIG1vZGVsIG92ZXJhbGwgaW4gYWxsIGNhc2VzIGFzIG5vdCBhbGwgYmlvZGl2ZXJzaXR5IHNhbXBsaW5nIG1ldGhvZHMgaGF2ZSBhIGNvbXBhcmFibGUgc2NhbGUgKGUuZy4gYnV0dGVyZmx5IG5ldCBzd2VlcHMsIGxpbmVhciB0cmFuc2VjdHMpLiANCg0KYGBge3J9DQoNCiMgIm1lYW4iIGluIG1vZGVsIG5hbWUgcmVmZXJzIHRvIGxuUlIsIHZyID0gbG5WUiwgY3ZyID0gbG5DVlINCiMgdXIgPSB1bnJlc3RvcmVkL3Jlc3RvcmVkIGNvbXBhcmlzb24sIHJyID0gcmVzdG9yZWQvcmVmZXJlbmNlIGNvbXBhcmlzb24sIHEgPSBxdWFkcmF0IHNpemUgaW5jbHVkZWQNCnVuX3JlJGxuX3FzaXplPC1sb2codW5fcmUkdF9xc2l6ZV9tMikNCnJlX3JlZiRsbl9xc2l6ZTwtbG9nKHJlX3JlZiR0X3FzaXplX20yKQ0KDQpjdnJfdXIgPC0gcm1hLm12KHlpX2N2ciwgdmN2X2N2ciwgcmFuZG9tID0gbGlzdCh+MSB8IGlkLCB+MSB8IHBsb3RfaWQsIH4xIHwgdW5pdCksIG1ldGhvZCA9ICJSRU1MIiwgZGF0YSA9IHVuX3JlKQ0KY3ZyX3FfdXIgPC0gcm1hLm12KHlpX2N2ciwgdmN2X2N2ciwgbW9kcz0gfmxuX3FzaXplIC0gMSwgcmFuZG9tID0gbGlzdCh+MSB8IGlkLCB+MSB8IHBsb3RfaWQsIH4xIHwgdW5pdCksIG1ldGhvZCA9ICJSRU1MIiwgZGF0YSA9IHVuX3JlKQ0KdnJfdXIgPC0gcm1hLm12KHlpX3ZyLCB2Y3ZfdnIsIHJhbmRvbSA9IGxpc3QofjEgfCBpZCwgfjEgfCBwbG90X2lkLCB+MSB8IHVuaXQpLCBtZXRob2QgPSAiUkVNTCIsIGRhdGEgPSB1bl9yZSkNCnZyX3FfdXIgPC0gcm1hLm12KHlpX3ZyLCB2Y3ZfdnIsIG1vZHM9IH5sbl9xc2l6ZSAtIDEsIHJhbmRvbSA9IGxpc3QofjEgfCBpZCwgfjEgfCBwbG90X2lkLCB+MSB8IHVuaXQpLCBtZXRob2QgPSAiUkVNTCIsIGRhdGEgPSB1bl9yZSkNCm1lYW5fdXIgPC0gcm1hLm12KHlpX21lYW4sIHZjdl9tZWFuLCByYW5kb20gPSBsaXN0KH4xIHwgaWQsIH4xIHwgcGxvdF9pZCwgfjEgfCB1bml0KSwgbWV0aG9kID0gIlJFTUwiLCBkYXRhID0gdW5fcmUpDQptZWFuX3FfdXIgPC0gcm1hLm12KHlpX21lYW4sIHZjdl9tZWFuLCBtb2RzPSB+bG5fcXNpemUgLSAxLCByYW5kb20gPSBsaXN0KH4xIHwgaWQsIH4xIHwgcGxvdF9pZCwgfjEgfCB1bml0KSwgbWV0aG9kID0gIlJFTUwiLCBkYXRhID0gdW5fcmUpDQoNCmN2cl9yciA8LSBybWEubXYoeWlfY3ZyLCB2Y3ZfY3ZyX3JyLCByYW5kb20gPSBsaXN0KH4xIHwgaWQsIH4xIHwgcGxvdF9pZCwgfjEgfCB1bml0KSwgbWV0aG9kID0gIlJFTUwiLCBkYXRhID0gcmVfcmVmKQ0KY3ZyX3FfcnIgPC0gcm1hLm12KHlpX2N2ciwgdmN2X2N2cl9yciwgbW9kcz0gfmxuX3FzaXplIC0gMSwgcmFuZG9tID0gbGlzdCh+MSB8IGlkLCB+MSB8IHBsb3RfaWQsIH4xIHwgdW5pdCksIG1ldGhvZCA9ICJSRU1MIiwgZGF0YSA9IHJlX3JlZikNCnZyX3JyIDwtIHJtYS5tdih5aV92ciwgdmN2X3ZyX3JyLCByYW5kb20gPSBsaXN0KH4xIHwgaWQsIH4xIHwgcGxvdF9pZCwgfjEgfCB1bml0KSwgbWV0aG9kID0gIlJFTUwiLCBkYXRhID0gcmVfcmVmKQ0KdnJfcV9yciA8LSBybWEubXYoeWlfdnIsIHZjdl92cl9yciwgbW9kcz0gfmxuX3FzaXplIC0gMSwgcmFuZG9tID0gbGlzdCh+MSB8IGlkLCB+MSB8IHBsb3RfaWQsIH4xIHwgdW5pdCksIG1ldGhvZCA9ICJSRU1MIiwgZGF0YSA9IHJlX3JlZikNCm1lYW5fcnIgPC0gcm1hLm12KHlpX21lYW4sIHZjdl9tZWFuX3JyLCByYW5kb20gPSBsaXN0KH4xIHwgaWQsIH4xIHwgcGxvdF9pZCwgfjEgfCB1bml0KSwgbWV0aG9kID0gIlJFTUwiLCBkYXRhID0gcmVfcmVmKQ0KbWVhbl9xX3JyIDwtIHJtYS5tdih5aV9tZWFuLCB2Y3ZfbWVhbl9yciwgbW9kcz0gfmxuX3FzaXplIC0gMSwgcmFuZG9tID0gbGlzdCh+MSB8IGlkLCB+MSB8IHBsb3RfaWQsIH4xIHwgdW5pdCksIG1ldGhvZCA9ICJSRU1MIiwgZGF0YSA9IHJlX3JlZikNCg0KYGBgDQoNCioqVGFibGUgUzE6KiogT3ZlcmFsbCBlZmZlY3RzIChtZXRhLWFuYWx5dGljIG1lYW5zKSwgOTUlIGNvbmZpZGVuY2UgaW50ZXJ2YWxzIChDSXMpIGFuZCA5NSUgcHJlZGljdGlvbiBpbnRlcnZhbHMgKDk1JSkuIGxuQ1ZSID0gbG9nIENWIHJhdGlvIChjb2VmZmljaWVudCBvZiB2YXJpYXRpb24pLCBsblJSID0gbG9nIHJlc3BvbnNlIHJhdGlvIChtZWFuKSwgbG5WUiA9IGxvZyB2YXJpYXRpb24gcmF0aW8gKFNEKS4NCg0KYGBge3J9DQojIGdldHRpbmcgYSB0YWJsZSBvZiBDSSBhbmQgUEkNCg0KcHJlZF9jdnJfdXIgPC0gZ2V0X3ByZWQxKGN2cl91ciwgbW9kID0gIkludCIpDQpwcmVkX3ZyX3VyIDwtIGdldF9wcmVkMSh2cl91ciwgbW9kID0gIkludCIpDQpwcmVkX21lYW5fdXIgPC0gZ2V0X3ByZWQxKG1lYW5fdXIsIG1vZCA9ICJJbnQiKQ0KcHJlZF9jdnJfcnIgPC0gZ2V0X3ByZWQxKGN2cl9yciwgbW9kID0gIkludCIpDQpwcmVkX3ZyX3JyIDwtIGdldF9wcmVkMSh2cl9yciwgbW9kID0gIkludCIpDQpwcmVkX21lYW5fcnIgPC0gZ2V0X3ByZWQxKG1lYW5fcnIsIG1vZCA9ICJJbnQiKQ0KDQoNCiMgRHJhd2luZyBhIHRhYmxlIGZvciBtZXRhLWFuYWx5c2VzDQp0aWJibGUoYEVmZmVjdCBzaXplYCA9IGMoImxuQ1ZSIC0gdW5yZXN0b3JlZCIsICJsblZSIC0gdW5yZXN0b3JlZCIsICJsblJSIC0gdW5yZXN0b3JlZCIsICJsbkNWUiAtIHJlZmVyZW5jZSIsICJsblZSIC0gcmVmZXJlbmNlIiwgImxuUlIgLSByZWZlcmVtY2UiKSwgDQogICAgICAgYE92ZXJhbGwgbWVhbmAgPSBjKHByZWRfY3ZyX3VyJGVzdGltYXRlLCBwcmVkX3ZyX3VyJGVzdGltYXRlLCBwcmVkX21lYW5fdXIkZXN0aW1hdGUsIHByZWRfY3ZyX3JyJGVzdGltYXRlLCBwcmVkX3ZyX3JyJGVzdGltYXRlLCBwcmVkX21lYW5fcnIkZXN0aW1hdGUpLCANCiAgICAgICBgTG93ZXIgQ0kgWzAuMDI1XWAgPSBjKHByZWRfY3ZyX3VyJGxvd2VyQ0wsIHByZWRfdnJfdXIkbG93ZXJDTCwgcHJlZF9tZWFuX3VyJGxvd2VyQ0wsIHByZWRfY3ZyX3JyJGxvd2VyQ0wsIHByZWRfdnJfcnIkbG93ZXJDTCwgcHJlZF9tZWFuX3JyJGxvd2VyQ0wpLCANCiAgICAgICBgVXBwZXIgQ0kgWzAuOTc1XWAgPSBjKHByZWRfY3ZyX3VyJHVwcGVyQ0wsIHByZWRfdnJfdXIkdXBwZXJDTCwgcHJlZF9tZWFuX3VyJHVwcGVyQ0wsIHByZWRfY3ZyX3JyJHVwcGVyQ0wsIHByZWRfdnJfcnIkdXBwZXJDTCwgcHJlZF9tZWFuX3JyJHVwcGVyQ0wpLA0KICAgICAgIGBQIHZhbHVlYCAgICAgICAgICA9IGMocHJlZF9jdnJfdXIkcHZhbCwgcHJlZF92cl91ciRwdmFsLCBwcmVkX21lYW5fdXIkcHZhbCxwcmVkX2N2cl9yciRwdmFsLCBwcmVkX3ZyX3JyJHB2YWwsIHByZWRfbWVhbl9yciRwdmFsKSwNCiAgICAgICBgTG93ZXIgUEkgWzAuMDI1XWAgPSBjKHByZWRfY3ZyX3VyJGxvd2VyUFIsIHByZWRfdnJfdXIkbG93ZXJQUiwgcHJlZF9tZWFuX3VyJGxvd2VyUFIsIHByZWRfY3ZyX3JyJGxvd2VyUFIsIHByZWRfdnJfcnIkbG93ZXJQUiwgcHJlZF9tZWFuX3JyJGxvd2VyUFIpLCANCiAgICAgICBgVXBwZXIgUEkgWzAuOTc1XWAgPSBjKHByZWRfY3ZyX3VyJHVwcGVyUFIsIHByZWRfdnJfdXIkdXBwZXJQUiwgcHJlZF9tZWFuX3VyJHVwcGVyUFIsIHByZWRfY3ZyX3JyJHVwcGVyUFIsIHByZWRfdnJfcnIkdXBwZXJQUiwgcHJlZF9tZWFuX3JyJHVwcGVyUFIpKSAlPiUgDQogIGthYmxlKCJodG1sIiwgZGlnaXRzID0gMykgJT4lIA0KICBrYWJsZV9zdHlsaW5nKCJzdHJpcGVkIiwgcG9zaXRpb24gPSAibGVmdCIpJT4lDQogICAgc2Nyb2xsX2JveCh3aWR0aCA9ICI4MDBweCIsIGhlaWdodCA9ICIzMDBweCIpDQpgYGANCg0KDQoqKlRhYmxlIFMyOioqIEhldGVyb2dlbmVpdHkgYW1vbmcgZWZmZWN0cyBvZiByZXN0b3JhdGlvbiwgbWVhc3VyZWQgdXNpbmcgSVxeMi4NCg0KDQoNCmBgYHtyfQ0KY3ZydXI8LUkyKGN2cl91cikNCnZydXI8LUkyKHZyX3VyKQ0KbWVhbnVyPC1JMihtZWFuX3VyKQ0KY3ZycnI8LUkyKGN2cl9ycikNCnZycnI8LUkyKHZyX3JyKQ0KbWVhbnJyPC1JMihtZWFuX3JyKQ0KDQp0Ymw8LXJiaW5kKGN2cnVyLCB2cnVyLCBtZWFudXIsIGN2cnJyLCB2cnJyLCBtZWFucnIpDQp0Ymw8LWFzLmRhdGEuZnJhbWUodGJsKQ0KdGJsJE1vZGVsPC1jKCJMbkNWUiAtIHVucmVzdG9yZWQvcmVzdG9yZWQiLCAiTG5WUiAtIHVucmVzdG9yZWQvcmVzdG9yZWQiLCJMblJSIC0gdW5yZXN0b3JlZC9yZXN0b3JlZCIsDQogICAgICAgICAgICAgIkxuQ1ZSIC0gcmVmZXJlbmNlL3Jlc3RvcmVkIiwgIkxuVlIgLSByZWZlcmVuY2UvcmVzdG9yZWQiLCAiTG5SUiAtIHJlZmVyZW5jZS9yZXN0b3JlZCIpDQp0YmwgJT4lIGthYmxlKCJodG1sIiwgZGlnaXRzID0gNCkgJT4lIA0KICBrYWJsZV9zdHlsaW5nKCJzdHJpcGVkIiwgcG9zaXRpb24gPSAibGVmdCIpDQpgYGANCg0KIyMjIFVuaXZhcmlhdGUgbW9kZWxzIG9mIGFnZSwgc2l6ZSwgYW5kIHBhc3QgbGFuZCB1c2UNCg0KYGBge3IsIGZpZy53aWR0aD03LCBmaWcuaGVpZ2h0PTN9DQoNCiAjIyMjIyMNCiMgU0laRSAjDQogIyMjIyMjDQoNCg0KIyBnZXR0aW5nIGEgdGFibGUgb2YgQ0kgYW5kIFBJDQoNCnVuX3JlJHNpdGVfc2l6ZTwtaWZlbHNlKHVuX3JlJHNpdGVfc2l6ZSA9PSAwLCAwLjEsIHVuX3JlJHNpdGVfc2l6ZSkNCnVuX3JlX3N6PC11bl9yZSAlPiUgZHJvcF9uYShzaXRlX3NpemUpDQoNCiMgbmVlZCBhIG5ldyB2Y3YgbWF0cml4IGJlY2F1c2UgYWJvdmUgcmVkdWNlcyB0aGUgc2FtcGxlIHNsaWdodGx5IChOQSBhZ2UgcmVtb3ZhbCkNCg0KdmN2X2N2cl9zejwtbWFrZV9WQ1ZfbWF0cml4KHVuX3JlX3N6LCBWID0idmlfY3ZyIiwgInNoYXJlZF9jdHJsIiwgInVuaXQiLCByaG89MC41KQ0KdmN2X21lYW5fc3o8LW1ha2VfVkNWX21hdHJpeCh1bl9yZV9zeiwgViA9InZpX21lYW4iLCAic2hhcmVkX2N0cmwiLCAidW5pdCIsIHJobz0wLjUpDQp2Y3ZfdnJfc3o8LW1ha2VfVkNWX21hdHJpeCh1bl9yZV9zeiwgViA9InZpX3ZyIiwgInNoYXJlZF9jdHJsIiwgInVuaXQiLCByaG89MC41KQ0KDQptZWFuX3NpemVfdXIgPC0gcm1hLm12KHlpX21lYW4sIHZjdl9tZWFuX3N6LCBtb2RzID0gfmxvZyhzaXRlX3NpemUpLCByYW5kb20gPSBsaXN0KH4xIHwgaWQsIH4xIHwgcGxvdF9pZCwgfjEgfCB1bml0KSwgbWV0aG9kID0gIlJFTUwiLCBkYXRhID0gdW5fcmVfc3opDQp2cl9zaXplX3VyIDwtIHJtYS5tdih5aV92ciwgdmN2X3ZyX3N6LCBtb2RzID0gfmxvZyhzaXRlX3NpemUpLCByYW5kb20gPSBsaXN0KH4xIHwgaWQsIH4xIHwgcGxvdF9pZCwgfjEgfCB1bml0KSwgbWV0aG9kID0gIlJFTUwiLCBkYXRhID0gdW5fcmVfc3opDQpjdnJfc2l6ZV91ciA8LSBybWEubXYoeWlfY3ZyLCB2Y3ZfY3ZyX3N6LCBtb2RzID0gfmxvZyhzaXRlX3NpemUpLCByYW5kb20gPSBsaXN0KH4xIHwgaWQsIH4xIHwgcGxvdF9pZCwgfjEgfCB1bml0KSwgbWV0aG9kID0gIlJFTUwiLCBkYXRhID0gdW5fcmVfc3opDQptZWFuX3NpemVfdXJfcSA8LSBybWEubXYoeWlfbWVhbiwgdmN2X21lYW5fc3osIG1vZHMgPSB+bG9nKHNpdGVfc2l6ZSkgKyBsb2codF9xc2l6ZV9tMiksIHJhbmRvbSA9IGxpc3QofjEgfCBpZCwgfjEgfCBwbG90X2lkLCB+MSB8IHVuaXQpLCBtZXRob2QgPSAiUkVNTCIsIGRhdGEgPSB1bl9yZV9zeikNCnZyX3NpemVfdXJfcSA8LSBybWEubXYoeWlfdnIsIHZjdl92cl9zeiwgbW9kcyA9IH5sb2coc2l0ZV9zaXplKSArIGxvZyh0X3FzaXplX20yKSwgcmFuZG9tID0gbGlzdCh+MSB8IGlkLCB+MSB8IHBsb3RfaWQsIH4xIHwgdW5pdCksIG1ldGhvZCA9ICJSRU1MIiwgZGF0YSA9IHVuX3JlX3N6KQ0KY3ZyX3NpemVfdXJfcSA8LSBybWEubXYoeWlfY3ZyLCB2Y3ZfY3ZyX3N6LCBtb2RzID0gfmxvZyhzaXRlX3NpemUpICsgbG9nKHRfcXNpemVfbTIpLCByYW5kb20gPSBsaXN0KH4xIHwgaWQsIH4xIHwgcGxvdF9pZCwgfjEgfCB1bml0KSwgbWV0aG9kID0gIlJFTUwiLCBkYXRhID0gdW5fcmVfc3opDQoNCg0KIyBuZWVkIGEgbmV3IHZjdiBtYXRyaXggYmVjYXVzZSBhYm92ZSByZWR1Y2VzIHRoZSBzYW1wbGUgc2xpZ2h0bHkgKE5BIHNpemUgcmVtb3ZhbCkNCg0KcmVfcmVmJHNpdGVfc2l6ZTwtaWZlbHNlKHJlX3JlZiRzaXRlX3NpemUgPT0gMCwgMC4xLCByZV9yZWYkc2l0ZV9zaXplKQ0KcmVfcmVmX3N6PC1yZV9yZWYgJT4lIGRyb3BfbmEoc2l0ZV9zaXplKQ0KDQp2Y3ZfY3ZyX3JyX3N6PC1tYWtlX1ZDVl9tYXRyaXgocmVfcmVmX3N6LCBWID0idmlfY3ZyIiwgInNoYXJlZF9jdHJsIiwgInVuaXQiLCByaG89MC41KQ0KdmN2X21lYW5fcnJfc3o8LW1ha2VfVkNWX21hdHJpeChyZV9yZWZfc3osIFYgPSJ2aV9tZWFuIiwgInNoYXJlZF9jdHJsIiwgInVuaXQiLCByaG89MC41KQ0KdmN2X3ZyX3JyX3N6PC1tYWtlX1ZDVl9tYXRyaXgocmVfcmVmX3N6LCBWID0idmlfdnIiLCAic2hhcmVkX2N0cmwiLCAidW5pdCIsIHJobz0wLjUpDQoNCm1lYW5fc2l6ZV9yciA8LSBybWEubXYoeWlfbWVhbiwgdmN2X21lYW5fcnJfc3osIG1vZHMgPSB+bG9nKHNpdGVfc2l6ZSksIHJhbmRvbSA9IGxpc3QofjEgfCBpZCwgfjEgfCBwbG90X2lkLCB+MSB8IHVuaXQpLCBtZXRob2QgPSAiUkVNTCIsIGRhdGEgPSByZV9yZWZfc3opDQp2cl9zaXplX3JyIDwtIHJtYS5tdih5aV92ciwgdmN2X3ZyX3JyX3N6LCBtb2RzID0gfmxvZyhzaXRlX3NpemUpLCByYW5kb20gPSBsaXN0KH4xIHwgaWQsIH4xIHwgcGxvdF9pZCwgfjEgfCB1bml0KSwgbWV0aG9kID0gIlJFTUwiLCBkYXRhID0gcmVfcmVmX3N6KQ0KY3ZyX3NpemVfcnIgPC0gcm1hLm12KHlpX2N2ciwgdmN2X2N2cl9ycl9zeiwgbW9kcyA9IH5sb2coc2l0ZV9zaXplKSwgcmFuZG9tID0gbGlzdCh+MSB8IGlkLCB+MSB8IHBsb3RfaWQsIH4xIHwgdW5pdCksIG1ldGhvZCA9ICJSRU1MIiwgZGF0YSA9IHJlX3JlZl9zeikNCm1lYW5fc2l6ZV9ycl9xIDwtIHJtYS5tdih5aV9tZWFuLCB2Y3ZfbWVhbl9ycl9zeiwgbW9kcyA9IH5sb2coc2l0ZV9zaXplKSsgbG9nKHRfcXNpemVfbTIpLCByYW5kb20gPSBsaXN0KH4xIHwgaWQsIH4xIHwgcGxvdF9pZCwgfjEgfCB1bml0KSwgbWV0aG9kID0gIlJFTUwiLCBkYXRhID0gcmVfcmVmX3N6KQ0KdnJfc2l6ZV9ycl9xIDwtIHJtYS5tdih5aV92ciwgdmN2X3ZyX3JyX3N6LCBtb2RzID0gfmxvZyhzaXRlX3NpemUpKyBsb2codF9xc2l6ZV9tMiksIHJhbmRvbSA9IGxpc3QofjEgfCBpZCwgfjEgfCBwbG90X2lkLCB+MSB8IHVuaXQpLCBtZXRob2QgPSAiUkVNTCIsIGRhdGEgPSByZV9yZWZfc3opDQpjdnJfc2l6ZV9ycl9xIDwtIHJtYS5tdih5aV9jdnIsIHZjdl9jdnJfcnJfc3osIG1vZHMgPSB+bG9nKHNpdGVfc2l6ZSkrIGxvZyh0X3FzaXplX20yKSwgcmFuZG9tID0gbGlzdCh+MSB8IGlkLCB+MSB8IHBsb3RfaWQsIH4xIHwgdW5pdCksIG1ldGhvZCA9ICJSRU1MIiwgZGF0YSA9IHJlX3JlZl9zeikNCg0KDQogIyMjIyMNCiMgQUdFICMNCiAjIyMjIw0KDQp1bl9yZSRhZ2UucmVzdC48LWlmZWxzZSh1bl9yZSRhZ2UucmVzdC4gPT0gMCwgMC4xLCB1bl9yZSRhZ2UucmVzdC4pDQp1bl9yZV9hZzwtdW5fcmUgJT4lIHRpZHlyOjpkcm9wX25hKGFnZS5yZXN0LikNCg0KIyBuZWVkIGEgbmV3IHZjdiBtYXRyaXggYmVjYXVzZSBhYm92ZSByZWR1Y2VzIHRoZSBzYW1wbGUgc2xpZ2h0bHkgKE5BIGFnZSByZW1vdmFsKQ0KDQp2Y3ZfY3ZyX2FnPC1tYWtlX1ZDVl9tYXRyaXgodW5fcmVfYWcsIFYgPSJ2aV9jdnIiLCAic2hhcmVkX2N0cmwiLCAidW5pdCIsIHJobz0wLjUpDQp2Y3ZfbWVhbl9hZzwtbWFrZV9WQ1ZfbWF0cml4KHVuX3JlX2FnLCBWID0idmlfbWVhbiIsICJzaGFyZWRfY3RybCIsICJ1bml0IiwgcmhvPTAuNSkNCnZjdl92cl9hZzwtbWFrZV9WQ1ZfbWF0cml4KHVuX3JlX2FnLCBWID0idmlfdnIiLCAic2hhcmVkX2N0cmwiLCAidW5pdCIsIHJobz0wLjUpDQoNCm1lYW5fYWdlX3VyIDwtIHJtYS5tdih5aV9tZWFuLCB2Y3ZfbWVhbl9hZywgbW9kcyA9IH4oYWdlLnJlc3QuKSwgcmFuZG9tID0gbGlzdCh+MSB8IGlkLCB+MSB8IHBsb3RfaWQsIH4xIHwgdW5pdCksIG1ldGhvZCA9ICJSRU1MIiwgZGF0YSA9IHVuX3JlX2FnKQ0KdnJfYWdlX3VyIDwtIHJtYS5tdih5aV92ciwgdmN2X3ZyX2FnLCBtb2RzID0gfihhZ2UucmVzdC4pICwgcmFuZG9tID0gbGlzdCh+MSB8IGlkLCB+MSB8IHBsb3RfaWQsIH4xIHwgdW5pdCksIG1ldGhvZCA9ICJSRU1MIiwgZGF0YSA9IHVuX3JlX2FnKQ0KY3ZyX2FnZV91ciA8LSBybWEubXYoeWlfY3ZyLCB2Y3ZfY3ZyX2FnLCBtb2RzID0gfihhZ2UucmVzdC4pLCByYW5kb20gPSBsaXN0KH4xIHwgaWQsIH4xIHwgcGxvdF9pZCwgfjEgfCB1bml0KSwgbWV0aG9kID0gIlJFTUwiLCBkYXRhID0gdW5fcmVfYWcpDQptZWFuX2FnZV91cl9xIDwtIHJtYS5tdih5aV9tZWFuLCB2Y3ZfbWVhbl9hZywgbW9kcyA9IH4oYWdlLnJlc3QuKSsgbG9nKHRfcXNpemVfbTIpLCByYW5kb20gPSBsaXN0KH4xIHwgaWQsIH4xIHwgcGxvdF9pZCwgfjEgfCB1bml0KSwgbWV0aG9kID0gIlJFTUwiLCBkYXRhID0gdW5fcmVfYWcpDQp2cl9hZ2VfdXJfcSA8LSBybWEubXYoeWlfdnIsIHZjdl92cl9hZywgbW9kcyA9IH4oYWdlLnJlc3QuKSArIGxvZyh0X3FzaXplX20yKSwgcmFuZG9tID0gbGlzdCh+MSB8IGlkLCB+MSB8IHBsb3RfaWQsIH4xIHwgdW5pdCksIG1ldGhvZCA9ICJSRU1MIiwgZGF0YSA9IHVuX3JlX2FnKQ0KY3ZyX2FnZV91cl9xIDwtIHJtYS5tdih5aV9jdnIsIHZjdl9jdnJfYWcsIG1vZHMgPSB+KGFnZS5yZXN0LikrIGxvZyh0X3FzaXplX20yKSwgcmFuZG9tID0gbGlzdCh+MSB8IGlkLCB+MSB8IHBsb3RfaWQsIH4xIHwgdW5pdCksIG1ldGhvZCA9ICJSRU1MIiwgZGF0YSA9IHVuX3JlX2FnKQ0KDQoNCg0KDQojIG5lZWQgYSBuZXcgdmN2IG1hdHJpeCBiZWNhdXNlIGFib3ZlIHJlZHVjZXMgdGhlIHNhbXBsZSBzbGlnaHRseSAoTkEgYWdlIHJlbW92YWwpDQoNCnJlX3JlZiRhZ2UucmVzdC48LWlmZWxzZShyZV9yZWYkYWdlLnJlc3QuID09IDAsIDAuMSwgcmVfcmVmJGFnZS5yZXN0LikNCnJlX3JlZl9hZzwtcmVfcmVmICU+JSB0aWR5cjo6ZHJvcF9uYShhZ2UucmVzdC4pDQoNCnZjdl9jdnJfcnJfYWc8LW1ha2VfVkNWX21hdHJpeChyZV9yZWZfYWcsIFYgPSJ2aV9jdnIiLCAic2hhcmVkX2N0cmwiLCAidW5pdCIsIHJobz0wLjUpDQp2Y3ZfbWVhbl9ycl9hZzwtbWFrZV9WQ1ZfbWF0cml4KHJlX3JlZl9hZywgViA9InZpX21lYW4iLCAic2hhcmVkX2N0cmwiLCAidW5pdCIsIHJobz0wLjUpDQp2Y3ZfdnJfcnJfYWc8LW1ha2VfVkNWX21hdHJpeChyZV9yZWZfYWcsIFYgPSJ2aV92ciIsICJzaGFyZWRfY3RybCIsICJ1bml0IiwgcmhvPTAuNSkNCg0KbWVhbl9hZ2VfcnIgPC0gcm1hLm12KHlpX21lYW4sIHZjdl9tZWFuX3JyX2FnLCBtb2RzID0gfihhZ2UucmVzdC4pLCByYW5kb20gPSBsaXN0KH4xIHwgaWQsIH4xIHwgcGxvdF9pZCwgfjEgfCB1bml0KSwgbWV0aG9kID0gIlJFTUwiLCBkYXRhID0gcmVfcmVmX2FnKQ0KdnJfYWdlX3JyIDwtIHJtYS5tdih5aV92ciwgdmN2X3ZyX3JyX2FnLCBtb2RzID0gfihhZ2UucmVzdC4pICwgcmFuZG9tID0gbGlzdCh+MSB8IGlkLCB+MSB8IHBsb3RfaWQsIH4xIHwgdW5pdCksIG1ldGhvZCA9ICJSRU1MIiwgZGF0YSA9IHJlX3JlZl9hZykNCmN2cl9hZ2VfcnIgPC0gcm1hLm12KHlpX2N2ciwgdmN2X2N2cl9ycl9hZywgbW9kcyA9IH4oYWdlLnJlc3QuKSwgcmFuZG9tID0gbGlzdCh+MSB8IGlkLCB+MSB8IHBsb3RfaWQsIH4xIHwgdW5pdCksIG1ldGhvZCA9ICJSRU1MIiwgZGF0YSA9IHJlX3JlZl9hZykNCm1lYW5fYWdlX3JyX3EgPC0gcm1hLm12KHlpX21lYW4sIHZjdl9tZWFuX3JyX2FnLCBtb2RzID0gfihhZ2UucmVzdC4pKyBsb2codF9xc2l6ZV9tMiksIHJhbmRvbSA9IGxpc3QofjEgfCBpZCwgfjEgfCBwbG90X2lkLCB+MSB8IHVuaXQpLCBtZXRob2QgPSAiUkVNTCIsIGRhdGEgPSByZV9yZWZfYWcpDQp2cl9hZ2VfcnJfcSA8LSBybWEubXYoeWlfdnIsIHZjdl92cl9ycl9hZywgbW9kcyA9IH4oYWdlLnJlc3QuKSArIGxvZyh0X3FzaXplX20yKSwgcmFuZG9tID0gbGlzdCh+MSB8IGlkLCB+MSB8IHBsb3RfaWQsIH4xIHwgdW5pdCksIG1ldGhvZCA9ICJSRU1MIiwgZGF0YSA9IHJlX3JlZl9hZykNCmN2cl9hZ2VfcnJfcSA8LSBybWEubXYoeWlfY3ZyLCB2Y3ZfY3ZyX3JyX2FnLCBtb2RzID0gfihhZ2UucmVzdC4pKyBsb2codF9xc2l6ZV9tMiksIHJhbmRvbSA9IGxpc3QofjEgfCBpZCwgfjEgfCBwbG90X2lkLCB+MSB8IHVuaXQpLCBtZXRob2QgPSAiUkVNTCIsIGRhdGEgPSByZV9yZWZfYWcpDQoNCg0KICMjIyMjDQojIFBMVSAjDQogIyMjIyMNCiANCg0KbWVhbl9wbHVfdXIgPC0gcm1hLm12KHlpX21lYW4sIHZjdl9tZWFuLCBtb2RzID0gfnBsdSAtIDEgLCByYW5kb20gPSBsaXN0KH4xIHwgaWQsIH4xIHwgcGxvdF9pZCwgfjEgfCB1bml0KSwgbWV0aG9kID0gIlJFTUwiLCBkYXRhID0gdW5fcmUpDQp2cl9wbHVfdXIgPC0gcm1hLm12KHlpX3ZyLCB2Y3ZfdnIsIG1vZHMgPSB+cGx1IC0gMSwgcmFuZG9tID0gbGlzdCh+MSB8IGlkLCB+MSB8IHBsb3RfaWQsIH4xIHwgdW5pdCksIG1ldGhvZCA9ICJSRU1MIiwgZGF0YSA9IHVuX3JlKQ0KY3ZyX3BsdV91ciA8LSBybWEubXYoeWlfY3ZyLCB2Y3ZfY3ZyLCBtb2RzID0gfnBsdSAtIDEsIHJhbmRvbSA9IGxpc3QofjEgfCBpZCwgfjEgfCBwbG90X2lkLCB+MSB8IHVuaXQpLCBtZXRob2QgPSAiUkVNTCIsIGRhdGEgPSB1bl9yZSkNCm1lYW5fcGx1X3VyX3EgPC0gcm1hLm12KHlpX21lYW4sIHZjdl9tZWFuLCBtb2RzID0gfnBsdSArIGxvZyh0X3FzaXplX20yKSAtIDEsIHJhbmRvbSA9IGxpc3QofjEgfCBpZCwgfjEgfCBwbG90X2lkLCB+MSB8IHVuaXQpLCBtZXRob2QgPSAiUkVNTCIsIGRhdGEgPSB1bl9yZSkNCnZyX3BsdV91cl9xIDwtIHJtYS5tdih5aV92ciwgdmN2X3ZyLCBtb2RzID0gfnBsdSArIGxvZyh0X3FzaXplX20yKS0gMSwgcmFuZG9tID0gbGlzdCh+MSB8IGlkLCB+MSB8IHBsb3RfaWQsIH4xIHwgdW5pdCksIG1ldGhvZCA9ICJSRU1MIiwgZGF0YSA9IHVuX3JlKQ0KY3ZyX3BsdV91cl9xIDwtIHJtYS5tdih5aV9jdnIsIHZjdl9jdnIsIG1vZHMgPSB+cGx1ICsgbG9nKHRfcXNpemVfbTIpLSAxLCByYW5kb20gPSBsaXN0KH4xIHwgaWQsIH4xIHwgcGxvdF9pZCwgfjEgfCB1bml0KSwgbWV0aG9kID0gIlJFTUwiLCBkYXRhID0gdW5fcmUpDQoNCg0KbWVhbl9wbHVfcnIgPC0gcm1hLm12KHlpX21lYW4sIHZjdl9tZWFuX3JyLCBtb2RzID0gfnBsdSAtIDEsIHJhbmRvbSA9IGxpc3QofjEgfCBpZCwgfjEgfCBwbG90X2lkLCB+MSB8IHVuaXQpLCBtZXRob2QgPSAiUkVNTCIsIGRhdGEgPSByZV9yZWYpDQp2cl9wbHVfcnIgPC0gcm1hLm12KHlpX3ZyLCB2Y3ZfdnJfcnIsIG1vZHMgPSB+cGx1IC0gMSwgcmFuZG9tID0gbGlzdCh+MSB8IGlkLCB+MSB8IHBsb3RfaWQsIH4xIHwgdW5pdCksIG1ldGhvZCA9ICJSRU1MIiwgZGF0YSA9IHJlX3JlZikNCmN2cl9wbHVfcnIgPC0gcm1hLm12KHlpX2N2ciwgdmN2X2N2cl9yciwgbW9kcyA9IH5wbHUgLSAxLCByYW5kb20gPSBsaXN0KH4xIHwgaWQsIH4xIHwgcGxvdF9pZCwgfjEgfCB1bml0KSwgbWV0aG9kID0gIlJFTUwiLCBkYXRhID0gcmVfcmVmKQ0KbWVhbl9wbHVfcnJfcSA8LSBybWEubXYoeWlfbWVhbiwgdmN2X21lYW5fcnIsIG1vZHMgPSB+cGx1ICsgbG9nKHRfcXNpemVfbTIpLSAxLCByYW5kb20gPSBsaXN0KH4xIHwgaWQsIH4xIHwgcGxvdF9pZCwgfjEgfCB1bml0KSwgbWV0aG9kID0gIlJFTUwiLCBkYXRhID0gcmVfcmVmKQ0KdnJfcGx1X3JyX3EgPC0gcm1hLm12KHlpX3ZyLCB2Y3ZfdnJfcnIsIG1vZHMgPSB+cGx1ICsgbG9nKHRfcXNpemVfbTIpLSAxLCByYW5kb20gPSBsaXN0KH4xIHwgaWQsIH4xIHwgcGxvdF9pZCwgfjEgfCB1bml0KSwgbWV0aG9kID0gIlJFTUwiLCBkYXRhID0gcmVfcmVmKQ0KY3ZyX3BsdV9ycl9xIDwtIHJtYS5tdih5aV9jdnIsIHZjdl9jdnJfcnIsIG1vZHMgPSB+cGx1ICsgbG9nKHRfcXNpemVfbTIpLSAxLCByYW5kb20gPSBsaXN0KH4xIHwgaWQsIH4xIHwgcGxvdF9pZCwgfjEgfCB1bml0KSwgbWV0aG9kID0gIlJFTUwiLCBkYXRhID0gcmVfcmVmKQ0KDQoNCmBgYA0KDQojIyBNYW51c2NyaXB0IHBsb3RzDQoNCiMjIyBMb2NhdGlvbiBvZiBzdHVkaWVzDQoNCk91ciBzdHVkeSBzaXRlcyBoYWQgYSBnbG9iYWwgZGlzdHJpYnV0aW9uLCBob3dldmVyIHdpdGggc29tZSBjbGVhciBiaWFzZXMgdG93YXJkcyB0aGUgR2xvYmFsIE5vcnRoIChwYXJ0aWN1bGFybHkgTm9ydGggQW1lcmljYSBhbmQgRXVyb3BlKSwgd2l0aCB0aGUgY29udGluZW50cyBvZiBBZnJpY2EsIEFzaWEgYW5kIFNvdXRoIEFtZXJpY2EgcG9vcmx5IHJlcHJlc2VudGVkLg0KDQoNCmBgYHtyLCBmaWcuaGVpZ2h0ID0gNCwgZmlnLndpZHRoPTl9DQpsaWJyYXJ5KGdncGxvdDIpICAjIGdncGxvdCgpIGZvcnRpZnkoKQ0KbGlicmFyeShyd29ybGRtYXApICAjIGdldE1hcCgpDQoNCg0KI2xvYWQgc3BhdGlhbCBkYXRhDQpzcGF0aWFsX2RhdGEgPC0gcmVhZF9jc3YoIkRhdGEvc3R1ZGllcy5jc3YiKQ0Kd29ybGQgPC0gZ2V0TWFwKHJlc29sdXRpb24gPSAiaGlnaCIpDQoNCg0KZ2dwbG90KCkgKw0KICBnZW9tX3BvbHlnb24oZGF0YT1tYXBfZGF0YSgnd29ybGQnKSwgbWFwcGluZz1hZXMoeD1sb25nLCB5PWxhdCwgZ3JvdXA9Z3JvdXApLCBmaWxsPSJncmF5OTAiLCBjb2xvdXI9ImdyYXk3MCIsIHNpemUgPSAwLjI1KSArIA0KICBnZW9tX3BvaW50KGRhdGEgPSBzcGF0aWFsX2RhdGEsIGFlcyh4ID0gWSwgeSA9IFgpLCBzaGFwZT0gMTksIGNvbG9yID0gInJlZDQiLCAgc2l6ZSA9IDEpKw0KICB0aGVtZV9idyhiYXNlX3NpemUgPSAxNSkrDQogIGNvb3JkX2VxdWFsKCkrDQogIHlsaW0oLTYwLCA5MCkgKw0KICB4bGltKC0xNzksIDE5NSkgKw0KIHRoZW1lKGF4aXMudGl0bGUgPSBlbGVtZW50X2JsYW5rKCksDQogICAgICAgYXhpcy50aWNrcyA9IGVsZW1lbnRfYmxhbmsoKSwNCiAgICAgICBheGlzLnRleHQgPSBlbGVtZW50X2JsYW5rKCksDQogICAgICAgcGFuZWwuZ3JpZC5tYWpvciA9IGVsZW1lbnRfYmxhbmsoKSwgcGFuZWwuZ3JpZC5taW5vciA9IGVsZW1lbnRfYmxhbmsoKSkNCg0KDQojZ2dzYXZlKCJGaWd1cmVfMS5wZGYiLCBoZWlnaHQgPSA0LCB3aWR0aCA9IDgpDQpgYGANCg0KDQoqKkZpZ3VyZSBTNC4qKiBMb2NhdGlvbiBvZiBzdHVkaWVzIGluY2x1ZGVkIGluIHRoZSBtZXRhLWFuYWx5c2lzLg0KDQoNCiMjIyBNZXRhLWFuYWx5dGljIG1vZGVsDQoNCklcXjIgKGhldGVyb2dlbmVpdHkgaW5kZXgpIHZhbHVlcyBhcmUgY2FsY3VsYXRlZCB3aXRoaW4gdGhlIGNvZGUgY2h1bmsuDQoNCmBgYHtyIGZpZy5oZWlnaHQ9MTQsIGZpZy53aWR0aD03LCBjYWNoZT1UUlVFfQ0KIyBkcmF3aW5nIHBsb3RzDQoNCmkyY3ZydXI8LUkyKGN2cl91cikNCiBwMSA8LSBvcmNoYXJkX3Bsb3QoY3ZyX3VyLCBtb2Q9IkludCIsIHhsYWIgPSAibG9nKENWIHJhdGlvKSAtIHVucmVzdG9yZWQvcmVzdG9yZWQiLCBhbHBoYSA9IDAuMDUsIGsgPSBUUlVFKSArDQogIHNjYWxlX3lfZGlzY3JldGUobGFiZWxzID0gIk92ZXJhbGwgbWVhbiIpICsgDQogIHNjYWxlX2ZpbGxfbWFudWFsKHZhbHVlcz0iZ3JlZW40IikgKw0KICBzY2FsZV9jb2xvdXJfbWFudWFsKHZhbHVlcz0iZ3JlZW40IikgKw0KICBjb29yZF9jYXJ0ZXNpYW4oeGxpbSA9IGMoLTMsIDMpKSArIHRoZW1lKGxlZ2VuZC5wb3NpdGlvbiA9ICJub25lIikrDQogIGdlb21fcmljaHRleHQoeCA9IDIsDQogICAgICAgICAgIHkgPSAwLjcsDQogICAgICAgICAgIGxhYmVsID0gcGFzdGUoJzxpPk48c3ViPmVmZmVjdCBzaXplPC9zdWI8L2k+ID0nLCBjdnJfdXIkay5hbGwpLCBzaXplID0zLCBsYWJlbC5zaXplICA9IE5BKQ0KIA0KaTJ2cnVyPC1JMih2cl91cikNCnAyIDwtIG9yY2hhcmRfcGxvdCh2cl91ciwgbW9kPSJJbnQiLCB4bGFiID0gImxvZyh2YXJpYWJpbGl0eSByYXRpbykgLSB1bnJlc3RvcmVkL3Jlc3RvcmVkIiwgYWxwaGEgPSAwLjEsIGs9RikgKw0KICBzY2FsZV95X2Rpc2NyZXRlKGxhYmVscyA9ICJPdmVyYWxsIG1lYW4iKSArDQogIHNjYWxlX2ZpbGxfbWFudWFsKHZhbHVlcz0icmVkIikgKw0KICBzY2FsZV9jb2xvdXJfbWFudWFsKHZhbHVlcz0icmVkIikgKw0KICBjb29yZF9jYXJ0ZXNpYW4oeGxpbSA9IGMoLTMsIDMpKSsgdGhlbWUobGVnZW5kLnBvc2l0aW9uID0gIm5vbmUiKSsNCiAgZ2VvbV9yaWNodGV4dCh4ID0gMiwNCiAgICAgICAgICAgeSA9IDAuNywNCiAgICAgICAgICAgbGFiZWwgPSBwYXN0ZSgnPGk+TjxzdWI+ZWZmZWN0IHNpemU8L3N1YjwvaT4gPScsIHZyX3VyJGsuYWxsKSwgc2l6ZSA9MywgbGFiZWwuc2l6ZSAgPSBOQSkNCg0KaTJtZWFudXI8LUkyKG1lYW5fdXIpDQpwMyA8LSBvcmNoYXJkX3Bsb3QobWVhbl91ciwgbW9kPSJJbnQiLCB4bGFiID0gImxvZyhSZXNwb25zZSByYXRpbykgLSB1bnJlc3RvcmVkL3Jlc3RvcmVkIiwgYWxwaGEgPSAwLjEsIGs9RikgKw0KICBzY2FsZV95X2Rpc2NyZXRlKGxhYmVscyA9ICJPdmVyYWxsIG1lYW4iKSArIA0KICBzY2FsZV9maWxsX21hbnVhbCh2YWx1ZXM9InB1cnBsZSIpICsNCiAgc2NhbGVfY29sb3VyX21hbnVhbCh2YWx1ZXM9InB1cnBsZSIpICsNCiAgY29vcmRfY2FydGVzaWFuKHhsaW0gPSBjKC0zLCAzKSkrIHRoZW1lKGxlZ2VuZC5wb3NpdGlvbiA9ICJub25lIikrDQogIGdlb21fcmljaHRleHQoeCA9IDIsDQogICAgICAgICAgIHkgPSAwLjcsDQogICAgICAgICAgIGxhYmVsID0gcGFzdGUoJzxpPk48c3ViPmVmZmVjdCBzaXplPC9zdWI8L2k+ID0nLCBtZWFuX3VyJGsuYWxsKSwgc2l6ZSA9MywgbGFiZWwuc2l6ZSAgPSBOQSkNCg0KaTJjdnJycjwtSTIoY3ZyX3JyKQ0KcDQgPC0gb3JjaGFyZF9wbG90KGN2cl9yciwgbW9kPSJJbnQiLCB4bGFiID0gImxvZyhDViByYXRpbykgLSByZWZlcmVuY2UvcmVzdG9yZWQiLCBhbHBoYSA9IDAuMDUsIGs9RikgKw0KICBzY2FsZV95X2Rpc2NyZXRlKGxhYmVscyA9ICJPdmVyYWxsIG1lYW4iKSArDQogIHNjYWxlX2ZpbGxfbWFudWFsKHZhbHVlcz0iZ3JlZW40IikgKw0KICBzY2FsZV9jb2xvdXJfbWFudWFsKHZhbHVlcz0iZ3JlZW40IikgKw0KICBjb29yZF9jYXJ0ZXNpYW4oeGxpbSA9IGMoLTMsIDMpKSsgdGhlbWUobGVnZW5kLnBvc2l0aW9uID0gIm5vbmUiKSsNCiAgZ2VvbV9yaWNodGV4dCh4ID0gMiwNCiAgICAgICAgICAgeSA9IDAuNywNCiAgICAgICAgICAgbGFiZWwgPSBwYXN0ZSgnPGk+TjxzdWI+ZWZmZWN0IHNpemU8L3N1YjwvaT4gPScsIGN2cl9yciRrLmFsbCksIHNpemUgPTMsIGxhYmVsLnNpemUgID0gTkEpDQoNCmkydnJycjwtSTIodnJfcnIpDQpwNSA8LSBvcmNoYXJkX3Bsb3QodnJfcnIsIG1vZD0iSW50IiwgeGxhYiA9ICJsb2codmFyaWFiaWxpdHkgcmF0aW8pIC0gcmVmZXJlbmNlL3Jlc3RvcmVkIiwgYWxwaGEgPSAwLjEsIGs9RikgKw0KICBzY2FsZV95X2Rpc2NyZXRlKGxhYmVscyA9ICJPdmVyYWxsIG1lYW4iKSArIA0KICBzY2FsZV9maWxsX21hbnVhbCh2YWx1ZXM9InJlZCIpICsNCiAgc2NhbGVfY29sb3VyX21hbnVhbCh2YWx1ZXM9InJlZCIpICsNCiAgY29vcmRfY2FydGVzaWFuKHhsaW0gPSBjKC0zLCAzKSkrIHRoZW1lKGxlZ2VuZC5wb3NpdGlvbiA9ICJub25lIikgKw0KICBnZW9tX3JpY2h0ZXh0KHggPSAyLA0KICAgICAgICAgICB5ID0gMC43LA0KICAgICAgICAgICBsYWJlbCA9IHBhc3RlKCc8aT5OPHN1Yj5lZmZlY3Qgc2l6ZTwvc3ViPC9pPiA9JywgdnJfcnIkay5hbGwpLCBzaXplID0zLCBsYWJlbC5zaXplICA9IE5BKQ0KDQppMm1lYW5ycjwtSTIobWVhbl9ycikNCnA2IDwtIG9yY2hhcmRfcGxvdChtZWFuX3JyLCBtb2Q9IkludCIsIHhsYWIgPSAibG9nKFJlc3BvbnNlIHJhdGlvKSAtIHJlZmVyZW5jZS9yZXN0b3JlZCIsIGFscGhhID0gMC4xLCBrPUYpICsNCiAgc2NhbGVfeV9kaXNjcmV0ZShsYWJlbHMgPSAiT3ZlcmFsbCBtZWFuIikgKw0KICBzY2FsZV9maWxsX21hbnVhbCh2YWx1ZXM9InB1cnBsZSIpICsNCiAgc2NhbGVfY29sb3VyX21hbnVhbCh2YWx1ZXM9InB1cnBsZSIpICsNCiAgY29vcmRfY2FydGVzaWFuKHhsaW0gPSBjKC0zLCAzKSkrIHRoZW1lKGxlZ2VuZC5wb3NpdGlvbiA9ICJub25lIikrDQogIGdlb21fcmljaHRleHQoeCA9IDIsDQogICAgICAgICAgIHkgPSAwLjcsDQogICAgICAgICAgIGxhYmVsID0gcGFzdGUoJzxpPk48c3ViPmVmZmVjdCBzaXplPC9zdWI8L2k+ID0nLCBtZWFuX3JyJGsuYWxsKSwgc2l6ZSA9MywgbGFiZWwuc2l6ZSAgPSBOQSkNCiAgDQpmaWc8LXAxL3AyL3AzL3A0L3A1L3A2DQpmaWcNCg0KDQoNCg0KRmlndXJlMjwtcDMvcDEvcDYvcDQrcGxvdF9hbm5vdGF0aW9uKHRhZ19wcmVmaXggPSAiKCIsIHRhZ19sZXZlbHMgPSAiYSIsIHRhZ19zdWZmaXggPSAiKSIpDQojZ2dzYXZlKCJGaWd1cmVfMi5wZGYiLCBGaWd1cmUyLCBoZWlnaHQgPSAxMCwgd2lkdGggPSA3KQ0KDQpgYGANCg0KDQpgYGB7ciwgZmlnLmhlaWdodD02LCBmaWcud2lkdGg9Nn0NCnJyPC0oKChwMyt0aGVtZShheGlzLnRpdGxlID0gZWxlbWVudF9ibGFuaygpLA0KICAgICAgIHBhbmVsLmdyaWQubWFqb3IgPSBlbGVtZW50X2JsYW5rKCksDQogICAgICAgcGFuZWwuZ3JpZC5taW5vciA9IGVsZW1lbnRfYmxhbmsoKSwNCiAgICAgICBheGlzLnRleHQueCA9IGVsZW1lbnRfYmxhbmsoKSwNCiAgICAgICBheGlzLnRleHQueSA9IGVsZW1lbnRfdGV4dChhbmdsZT0wKQ0KICAgICAgICkrc2NhbGVfeV9kaXNjcmV0ZShsYWJlbHM9IlJlbGF0aXZlIHRvIFxudW5yZXN0b3JlZCIpKSkNCiApLw0KICAocDYrdGhlbWUoDQogICAgICAgYXhpcy5saW5lLngudG9wID0gZWxlbWVudF9ibGFuaygpLA0KICAgICAgIGF4aXMudGV4dC55ID0gZWxlbWVudF90ZXh0KGFuZ2xlPTApLA0KICAgICAgIHBhbmVsLmdyaWQubWFqb3IgPSBlbGVtZW50X2JsYW5rKCksIHBhbmVsLmdyaWQubWlub3IgPSBlbGVtZW50X2JsYW5rKCkpK3hsYWIoIkxvZyByZXNwb25zZSByYXRpbyIpK3NjYWxlX3lfZGlzY3JldGUobGFiZWxzPSJSZWxhdGl2ZSB0byBcbnJlZmVyZW5jZSIpKS8NCigoKHAxK3RoZW1lKGF4aXMudGl0bGUgPSBlbGVtZW50X2JsYW5rKCksDQogICAgICAgcGFuZWwuZ3JpZC5tYWpvciA9IGVsZW1lbnRfYmxhbmsoKSwNCiAgICAgICBwYW5lbC5ncmlkLm1pbm9yID0gZWxlbWVudF9ibGFuaygpLA0KICAgICAgIGF4aXMudGV4dC54ID0gZWxlbWVudF9ibGFuaygpLA0KICAgICAgIGF4aXMudGV4dC55ID0gZWxlbWVudF90ZXh0KGFuZ2xlPTApLA0KICAgICAgICkrc2NhbGVfeV9kaXNjcmV0ZShsYWJlbHM9IlJlbGF0aXZlIHRvIFxudW5yZXN0b3JlZCIpKSkNCiApLw0KICAocDQrdGhlbWUoDQogICAgICAgYXhpcy50ZXh0LnkgPSBlbGVtZW50X3RleHQoYW5nbGU9MCksDQogICAgICAgcGFuZWwuZ3JpZC5tYWpvciA9IGVsZW1lbnRfYmxhbmsoKSwgDQogICAgICAgcGFuZWwuZ3JpZC5taW5vciA9IGVsZW1lbnRfYmxhbmsoKSkreGxhYigiTG9nIENWIHJhdGlvIikrc2NhbGVfeV9kaXNjcmV0ZShsYWJlbHM9IlJlbGF0aXZlIHRvIFxucmVmZXJlbmNlIikpDQoNCiNnZ3NhdmUoIkZpZ3VyZV8yLnBkZiIsIHJyKQ0KYGBgDQoNCioqRmlndXJlIFM0LiBBbiBvcmNoYXJkIHBsb3Qgc2hvd2luZyB0aGUgbWV0YS1hbmFseXRpYyBtZWFuIChtZWFuIGVmZmVjdCBzaXplKSB3aXRoIGl0cyA5NSUgY29uZmlkZW5jZSBpbnRlcnZhbCAodGhpY2sgbGluZSkgYW5kIDk1JSBwcmVkaWN0aW9uIGludGVydmFsICh0aGluIGxpbmUpLCB3aXRoIG9ic2VydmVkIGVmZmVjdCBzaXplcyBiYXNlZCBvbiB2YXJpb3VzIHByZWNpc2lvbnMgKDEvU0UpLioqDQoNCmBgYHtyfQ0KbmFtZXM8LWFzLmRhdGEuZnJhbWUoYygibWVhbiAtIHJlc3RvcmVkL3VucmVzdG9yZWQiLCAibWVhbiAtIHJlc3RvcmVkL3JlZmVyZW5jZSIsICJjdnIgLSByZXN0b3JlZC91bnJlc3RvcmVkIiwgImN2ciAtIHJlc3RvcmVkL3JlZmVyZW5jZSIsICJ2ciAtIHJlc3RvcmVkL3VucmVzdG9yZWQiLCAgInZyIC0gcmVzdG9yZWQvcmVmZXJlbmNlIikpDQoNCm5hbWVzPC1gY29sbmFtZXM8LWAobmFtZXMsICJtb2RlbCIpDQoNCmkyX2FsbDwtYmluZF9yb3dzKGkybWVhbnVyLCBpMm1lYW5yciwgaTJjdnJ1ciwgaTJjdnJyciwgaTJ2cnVyLCBpMnZycnIpDQoNCmkyX2FsbDwtYmluZF9jb2xzKG5hbWVzLCBpMl9hbGwpDQoNCmthYmxlKGkyX2FsbCkgJT4lIGthYmxlX3N0eWxpbmcoKSU+JQ0KICAgIHNjcm9sbF9ib3god2lkdGggPSAiODAwcHgiLCBoZWlnaHQgPSAiMzAwcHgiKQ0KDQoNCmBgYA0KDQoqKlRhYmxlIFMzLioqIGkyIHZhbHVlcyAobWVhc3VyZSBvZiBoZXRlcm9nZW5laXR5IGFtb25nIHJlc3VsdHMpIGZvciBhbGwgbW9kZWxzDQoNCiMjIyBVbml2YXJpYXRlICh1bmktcHJlZGljdG9yKSBhbmFseXNlcw0KDQpXZSByYW4gYSB1bml2YXJpYXRlIG1ldGEtcmVncmVzc2lvbiBtb2RlbHMgYWJvdmUgZm9yIGVhY2ggb2YgdGhlIGZvbGxvd2luZyBtb2RlcmF0b3JzOiAxKSBgc2l0ZV9zaXplYCwgMikgYGFnZS5yZXN0LmAsIDMpIGBwbHVgLCB0byB0ZXN0IG91ciByZXNlYXJjaCBxdWVzdGlvbnMuIFdlIGFsc28gcmFuIHRoZSBzYW1lIG1vZGVscyB3aXRoIHRoZSBxdWFkcmF0IHNpemUgb24gYSByZWR1Y2VkIHNhbXBsZSBvZiB0aGUgZGF0YSAobm90IGFsbCBzYW1wbGluZyBtZXRob2RzIHVzZSBzYW1wbGluZyBtZXRob2QgbWVhc3VyYWJsZSBpbiBhcmVhKS4gSW4gbm8gY2FzZXMgZGlkIHRlcm1zIGNoYW5nZSBpbiBzaWduaWZpY2FuY2Ugb3IgZGlyZWN0aW9uIGFzIGEgcmVzdWx0IG9mIHRoZSBpbmNsdXNpb24gb2YgcXVhZHJhdCBzaXplLg0KDQpUaGUgZm9sbG93aW5nIGNvZGUgcnVucyBwbG90cyBhbmQgdGFibGVzIG9mIHRoZSByZXN1bHQuDQoNCiMjIyBBZ2Ugb2YgcmVzdG9yYXRpb24gc2l0ZQ0KDQpgYGB7ciwgZmlnLndpZHRoPTgsIGZpZy5oZWlnaHQ9MTJ9DQoNCg0KYTE8LXVuaV9tb2RfcGxvdF9ucyhjdnJfYWdlX3VyLCB1bl9yZV9hZywgbG9nX3JhdGlvID0gdW5fcmVfYWckeWlfY3ZyLCByZXNwb25zZSA9IHVuX3JlX2FnJGFnZS5yZXN0LiwgdmFyaWFuY2UgPSB1bl9yZV9hZyR2aV9jdnIpK3lsYWIoIkxvZyBDViByYXRpbyAocmVsYXRpdmUgdG8gdW5yZXN0b3JlZCIpK3hsYWIoIlJlc3RvcmVkIHNpdGUgYWdlICh5cikiKQ0KDQphMjwtdW5pX21vZF9wbG90X25zKHZyX2FnZV91ciwgdW5fcmVfYWcsIGxvZ19yYXRpbyA9IHVuX3JlX2FnJHlpX3ZyLCByZXNwb25zZSA9IHVuX3JlX2FnJGFnZS5yZXN0LiwgdmFyaWFuY2UgPSB1bl9yZV9hZyR2aV92cikreWxhYigiTG9nIFNEIHJhdGlvICAocmVsYXRpdmUgdG8gdW5yZXN0b3JlZCkiKSt4bGFiKCJSZXN0b3JlZCBzaXRlIGFnZSAoeXIpIikNCg0KYTM8LXVuaV9tb2RfcGxvdChtZWFuX2FnZV91ciwgdW5fcmVfYWcsIGxvZ19yYXRpbyA9IHVuX3JlX2FnJHlpX21lYW4sIHJlc3BvbnNlID0gdW5fcmVfYWckYWdlLnJlc3QuLCB2YXJpYW5jZSA9IHVuX3JlX2FnJHZpX21lYW4pK3lsYWIoIkxvZyByZXNwb25zZSByYXRpbyAocmVsYXRpdmUgdG8gdW5yZXN0b3JlZCkiKSt4bGFiKCJSZXN0b3JlZCBzaXRlIGFnZSAoeXIpIikNCg0KYTQ8LXVuaV9tb2RfcGxvdF9ucyhjdnJfYWdlX3JyLCByZV9yZWZfYWcsIGxvZ19yYXRpbyA9IHJlX3JlZl9hZyR5aV9jdnIsIHJlc3BvbnNlID0gcmVfcmVmX2FnJGFnZS5yZXN0LiwgdmFyaWFuY2UgPSByZV9yZWZfYWckdmlfY3ZyKSt5bGFiKCJMb2cgQ1YgcmF0aW8gKHJlbGF0aXZlIHRvIHJlZmVyZW5jZSkiKSt4bGFiKCJSZXN0b3JlZCBzaXRlIGFnZSAoeXIpIikNCg0KYTU8LXVuaV9tb2RfcGxvdF9ucyh2cl9hZ2VfcnIsIHJlX3JlZl9hZywgbG9nX3JhdGlvID0gcmVfcmVmX2FnJHlpX3ZyLCByZXNwb25zZSA9IHJlX3JlZl9hZyRhZ2UucmVzdC4sIHZhcmlhbmNlID0gcmVfcmVmX2FnJHZpX3ZyKSt5bGFiKCJMb2cgU0QgcmF0aW8gKHJlbGF0aXZlIHRvIHJlZmVyZW5lYykiKSt4bGFiKCJSZXN0b3JlZCBzaXRlIGFnZSAoeXIpIikNCg0KYTY8LXVuaV9tb2RfcGxvdF9ucyhtZWFuX2FnZV9yciwgcmVfcmVmX2FnLCBsb2dfcmF0aW8gPSByZV9yZWZfYWckeWlfbWVhbiwgcmVzcG9uc2UgPSByZV9yZWZfYWckYWdlLnJlc3QuLCB2YXJpYW5jZSA9IHJlX3JlZl9hZyR2aV9tZWFuKSt5bGFiKCJMb2cgcmVzcG9uc2UgcmF0aW8gKHJlbGF0aXZlIHRvIHJlZmVyZW5jZSkiKSt4bGFiKCJSZXN0b3JlZCBzaXRlIGFnZSAoeXIpIikNCg0KDQooYTF8IGEyIHwgYTMpIC8gKGE0IHwgYTUgfCBhNikgK3Bsb3RfYW5ub3RhdGlvbih0YWdfbGV2ZWxzID0gIkEiKQ0KDQpmaWczPC0oYTF8YTMpLyhhNHxhNikrcGxvdF9hbm5vdGF0aW9uKHRhZ19wcmVmaXggPSAiKCIsIHRhZ19sZXZlbHMgPSAiYSIsIHRhZ19zdWZmaXggPSAiKSIpDQojZ2dzYXZlKCJGaWd1cmVfMy5wZGYiLCBmaWczLCBoZWlnaHQgPSA4LCB3aWR0aCA9IDgpDQoNCmBgYA0KDQoqKkZpZ3VyZSBTNS4qKiBUaGUgcmVsYXRpb25zaGlwIGJldHdlZW4gc2l0ZSBhZ2UgYW5kIHRoZSBtZXRhLWFuYWx5aXRpYyBtZWFuLCB3aXRoIGl0cyA5NSUgY29uZmlkZW5jZSBpbnRlcnZhbCAocmVkIGRhc2hlZCBsaW5lKSBhbmQgOTUlIHByZWRpY3Rpb24gaW50ZXJ2YWwgKGJsdWUgZGFzaGVkIGxpbmUpLCB3aXRoIG9ic2VydmVkIGVmZmVjdCBzaXplcyBiYXNlZCBvbiB2YXJpb3VzIHByZWNpc2lvbnMgKDEvU0UpLg0KDQoNCiMjIyBTaXplIG9mIHJlc3RvcmF0aW9uIHNpdGUNCg0KYGBge3IsICBmaWcud2lkdGg9OSwgZmlnLmhlaWdodD05fQ0KDQpzejE8LXVuaV9tb2RfcGxvdF9ucyhjdnJfc2l6ZV91ciwgdW5fcmVfc3osIGxvZ19yYXRpbyA9IHVuX3JlX3N6JHlpX2N2ciwgcmVzcG9uc2UgPSBsb2codW5fcmVfc3okc2l0ZV9zaXplKSwgdmFyaWFuY2UgPSB1bl9yZV9zeiR2aV9jdnIpDQoNCnN6MjwtdW5pX21vZF9wbG90X25zKHZyX3NpemVfdXIsIHVuX3JlX3N6LCBsb2dfcmF0aW8gPSB1bl9yZV9zeiR5aV92ciwgcmVzcG9uc2UgPSBsb2codW5fcmVfc3okc2l0ZV9zaXplKSwgdmFyaWFuY2UgPSB1bl9yZV9zeiR2aV92cikNCg0Kc3ozPC11bmlfbW9kX3Bsb3RfbnMobWVhbl9zaXplX3VyLCB1bl9yZV9zeiwgbG9nX3JhdGlvID0gdW5fcmVfc3okeWlfbWVhbiwgcmVzcG9uc2UgPSBsb2codW5fcmVfc3okc2l0ZV9zaXplKSwgdmFyaWFuY2UgPSB1bl9yZV9zeiR2aV9tZWFuKQ0KDQpzejQ8LXVuaV9tb2RfcGxvdF9ucyhjdnJfc2l6ZV9yciwgcmVfcmVmX3N6LCBsb2dfcmF0aW8gPSByZV9yZWZfc3okeWlfY3ZyLCByZXNwb25zZSA9IGxvZyhyZV9yZWZfc3okc2l0ZV9zaXplKSwgdmFyaWFuY2UgPSByZV9yZWZfc3okdmlfY3ZyKQ0Kc3o1PC11bmlfbW9kX3Bsb3RfbnModnJfc2l6ZV9yciwgcmVfcmVmX3N6LCBsb2dfcmF0aW8gPSByZV9yZWZfc3okeWlfdnIsIHJlc3BvbnNlID0gbG9nKHJlX3JlZl9zeiRzaXRlX3NpemUpLCB2YXJpYW5jZSA9IHJlX3JlZl9zeiR2aV92cikNCnN6NjwtdW5pX21vZF9wbG90X25zKG1lYW5fc2l6ZV9yciwgcmVfcmVmX3N6LCBsb2dfcmF0aW8gPSByZV9yZWZfc3okeWlfbWVhbiwgcmVzcG9uc2UgPSBsb2cocmVfcmVmX3N6JHNpdGVfc2l6ZSksIHZhcmlhbmNlID0gcmVfcmVmX3N6JHZpX21lYW4pDQoNCnN6X2xpc3Q8LWxpc3Qoc3oxLHN6MixzejMsc3o0LHN6NSxzejYpDQoNCihzejEgKyB4bGFiKCJsbiByZXN0b3JhdGlvbiBzaXRlIHNpemUgKGhhKSIpICsgeWxhYigibG4ocmVzdG9yZWQgLyB1bnJlc3RvcmVkKSBbbG5DVlJdIikgfCBzejIgKyB4bGFiKCJsbiByZXN0b3JhdGlvbiBzaXRlIHNpemUgKGhhKSIpKyB5bGFiKCJsbihyZXN0b3JlZCAvIHVucmVzdG9yZWQpIFtsblZSXSIpfCBzejMrIHhsYWIoImxuIHJlc3RvcmF0aW9uIHNpdGUgc2l6ZSAoaGEpIikrIHlsYWIoImxuKHJlc3RvcmVkIC8gdW5yZXN0b3JlZCkgW2xuUlJdIikgKSAvIChzejQrIHhsYWIoImxuIHJlc3RvcmF0aW9uIHNpdGUgc2l6ZSAoaGEpIikgKyB5bGFiKCJsbihyZXN0b3JlZCAvIHJlZmVyZW5jZSkgW2xuQ1ZSXSIpIHwgc3o1ICsgeGxhYigibG4gcmVzdG9yYXRpb24gc2l0ZSBzaXplIChoYSkiKSArIHlsYWIoImxuKHJlc3RvcmVkIC8gdW5yZXN0b3JlZCkgW2xuVlJdIil8IHN6NisgeGxhYigibG4gcmVzdG9yYXRpb24gc2l0ZSBzaXplIChoYSkiKSsgeWxhYigibG4ocmVzdG9yZWQgLyB1bnJlc3RvcmVkKSBbbG5SUl0iKSkgK3Bsb3RfYW5ub3RhdGlvbih0YWdfbGV2ZWxzID0gIkEiKQ0KDQpGaWd1cmVfNDwtKHN6MSArIHhsYWIoIlJlc3RvcmVkIHNpdGUgc2l6ZSAobG9nIGhhKSIpKyB5bGFiKCJMb2cgQ1YgcmF0aW8gKHJlbGF0aXZlIHRvIHVucmVzdG9yZWQpIil8c3o0KyB4bGFiKCJSZXN0b3JlZCBzaXRlIHNpemUgKGxvZyBoYSkiKSsgeWxhYigiTG9nIENWIHJhdGlvIChyZWxhdGl2ZSB0byByZWZlcmVuY2UpIikpIC8gKHN6MysgeGxhYigiUmVzdG9yZWQgc2l0ZSBzaXplIChsb2cgaGEpIikrIHlsYWIoIkxvZyByZXNwb25zZSByYXRpbyAocmVsYXRpdmUgdG8gdW5yZXN0b3JlZCkiKSB8IHN6NisgeGxhYigiUmVzdG9yZWQgc2l0ZSBzaXplIChsb2cgaGEpIikrIHlsYWIoIkxvZyByZXNwb25zZSByYXRpbyAocmVsYXRpdmUgdG8gcmVmZXJlbmNlIikpICtwbG90X2Fubm90YXRpb24odGFnX3ByZWZpeCA9ICIoIiwgdGFnX2xldmVscyA9ICJhIiwgdGFnX3N1ZmZpeCA9ICIpIikNCg0KDQojZ2dzYXZlKCJGaWd1cmVfNC5wZGYiLCBwbG90ID0gRmlndXJlXzQsIGhlaWdodCA9IDgsIHdpZHRoID0gOCkNCg0KYGBgDQoNCioqRmlndXJlIFM2LioqIFRoZSByZWxhdGlvbnNoaXAgYmV0d2VlbiByZXN0b3JhdGlvbiBzaXRlIHNpemUgKGhhKSBhbmQgdGhlIGJpb2RpdmVyc2l0eSBjaGFuZ2UgZm9sbG93aW5nIHJlc3RvcmF0aW9uIGNvbXBhcmVkIHRvIHVucmVzdG9yZWQgYW5kIHJlZmVyZW5jZSBsZXZlbHMuDQoNCiMjIyBQYXN0IGxhbmQgdXNlDQoNCmBgYHtyLCAgZmlnLndpZHRoPTE1LCBmaWcuaGVpZ2h0PTIwfQ0KDQojIG1vZGlmeSBvcmNoYXJkX3Bsb3QgZnVuY3Rpb24gdG8gbm90IHBhc3RlIEsgYnV0IE4gaW5zdGVhZA0KDQpvcmNoYXJkX3Bsb3Q8LWZ1bmN0aW9uIChvYmplY3QsIG1vZCA9ICJJbnQiLCB4bGFiLCBOID0gIm5vbmUiLCANCiAgICBhbHBoYSA9IDAuMSwgYW5nbGUgPSA5MCwgY2IgPSBUUlVFLCBrID0gVFJVRSwgdHJhbnNmbSA9IGMoIm5vbmUiLCANCiAgICAgICAgInRhbmgiKSkgDQp7DQogICAgdHJhbnNmbSA8LSBtYXRjaC5hcmcodHJhbnNmbSkNCiAgICBpZiAoYW55KGNsYXNzKG9iamVjdCkgJWluJSBjKCJybWEubXYiLCAicm1hIikpKSB7DQogICAgICAgIGlmIChtb2QgIT0gIkludCIpIHsNCiAgICAgICAgICAgIG9iamVjdCA8LSBtb2RfcmVzdWx0cyhvYmplY3QsIG1vZCkNCiAgICAgICAgfQ0KICAgICAgICBlbHNlIHsNCiAgICAgICAgICAgIG9iamVjdCA8LSBtb2RfcmVzdWx0cyhvYmplY3QsIG1vZCA9ICJJbnQiKQ0KICAgICAgICB9DQogICAgfQ0KICAgIG1vZF90YWJsZSA8LSBvYmplY3QkbW9kX3RhYmxlDQogICAgZGF0YSA8LSBvYmplY3QkZGF0YQ0KICAgIGRhdGEkbW9kZXJhdG9yIDwtIGZhY3RvcihkYXRhJG1vZGVyYXRvciwgbGV2ZWxzID0gbW9kX3RhYmxlJG5hbWUsIA0KICAgICAgICBsYWJlbHMgPSBtb2RfdGFibGUkbmFtZSkNCiAgICBkYXRhJHNjYWxlIDwtICgxL3NxcnQoZGF0YVssICJ2aSJdKSkNCiAgICBsZWdlbmQgPC0gIlByZWNpc2lvbiAoMS9TRSkiDQogICAgaWYgKGFueShOICE9ICJub25lIikpIHsNCiAgICAgICAgZGF0YSRzY2FsZSA8LSBODQogICAgICAgIGxlZ2VuZCA8LSAiU2FtcGxlIFNpemUgKE4pIg0KICAgIH0NCiAgICBpZiAodHJhbnNmbSA9PSAidGFuaCIpIHsNCiAgICAgICAgY29scyA8LSBzYXBwbHkobW9kX3RhYmxlLCBpcy5udW1lcmljKQ0KICAgICAgICBtb2RfdGFibGVbLCBjb2xzXSA8LSBacl90b19yKG1vZF90YWJsZVssIGNvbHNdKQ0KICAgICAgICBkYXRhJHlpIDwtIFpyX3RvX3IoZGF0YSR5aSkNCiAgICAgICAgbGFiZWwgPC0geGxhYg0KICAgIH0NCiAgICBlbHNlIHsNCiAgICAgICAgbGFiZWwgPC0geGxhYg0KICAgIH0NCiAgICBtb2RfdGFibGUkSyA8LSBhcy52ZWN0b3IoYnkoZGF0YSwgZGF0YVssICJtb2RlcmF0b3IiXSwgDQogICAgICAgIGZ1bmN0aW9uKHgpIGxlbmd0aCh4WywgInlpIl0pKSkNCiAgICBncm91cF9ubyA8LSBucm93KG1vZF90YWJsZSkNCiAgICBjYnBsIDwtIGMoIiNFNjlGMDAiLCAiIzAwOUU3MyIsICIjRjBFNDQyIiwgDQogICAgICAgICIjMDA3MkIyIiwgIiNENTVFMDAiLCAiI0NDNzlBNyIsICIjNTZCNEU5IiwgDQogICAgICAgICIjOTk5OTk5IikNCiAgICBwbG90IDwtIGdncGxvdDI6OmdncGxvdChkYXRhID0gbW9kX3RhYmxlLCBhZXMoeCA9IGVzdGltYXRlLCANCiAgICAgICAgeSA9IG5hbWUpKSArIGdnYmVlc3dhcm06Omdlb21fcXVhc2lyYW5kb20oZGF0YSA9IGRhdGEsIA0KICAgICAgICBhZXMoeCA9IHlpLCB5ID0gbW9kZXJhdG9yLCBzaXplID0gc2NhbGUsIGNvbG91ciA9IG1vZGVyYXRvciksIA0KICAgICAgICBncm91cE9uWCA9IEZBTFNFLCBhbHBoYSA9IGFscGhhKSArIGdncGxvdDI6Omdlb21fZXJyb3JiYXJoKGFlcyh4bWluID0gbG93ZXJQUiwgDQogICAgICAgIHhtYXggPSB1cHBlclBSKSwgaGVpZ2h0ID0gMCwgc2hvdy5sZWdlbmQgPSBGQUxTRSwgc2l6ZSA9IDAuNSwgDQogICAgICAgIGFscGhhID0gMC42KSArIGdncGxvdDI6Omdlb21fZXJyb3JiYXJoKGFlcyh4bWluID0gbG93ZXJDTCwgDQogICAgICAgIHhtYXggPSB1cHBlckNMKSwgaGVpZ2h0ID0gMCwgc2hvdy5sZWdlbmQgPSBGQUxTRSwgc2l6ZSA9IDEuMikgKyANCiAgICAgICAgZ2dwbG90Mjo6Z2VvbV92bGluZSh4aW50ZXJjZXB0ID0gMCwgbGluZXR5cGUgPSAyLCBjb2xvdXIgPSAiYmxhY2siLCANCiAgICAgICAgICAgIGFscGhhID0gYWxwaGEpICsgZ2dwbG90Mjo6Z2VvbV9wb2ludChhZXMoZmlsbCA9IG5hbWUpLCANCiAgICAgICAgc2l6ZSA9IDMsIHNoYXBlID0gMjEpICsgZ2dwbG90Mjo6dGhlbWVfYncoKSArIGdncGxvdDI6Omd1aWRlcyhmaWxsID0gIm5vbmUiLCANCiAgICAgICAgY29sb3VyID0gIm5vbmUiKSArIGdncGxvdDI6OnRoZW1lKGxlZ2VuZC5wb3NpdGlvbiA9IGMoMSwgDQogICAgICAgIDApLCBsZWdlbmQuanVzdGlmaWNhdGlvbiA9IGMoMSwgMCkpICsgZ2dwbG90Mjo6dGhlbWUobGVnZW5kLnRpdGxlID0gZWxlbWVudF90ZXh0KHNpemUgPSA5KSkgKyANCiAgICAgICAgZ2dwbG90Mjo6dGhlbWUobGVnZW5kLmRpcmVjdGlvbiA9ICJob3Jpem9udGFsIikgKyANCiAgICAgICAgZ2dwbG90Mjo6dGhlbWUobGVnZW5kLmJhY2tncm91bmQgPSBlbGVtZW50X2JsYW5rKCkpICsgDQogICAgICAgIGdncGxvdDI6OmxhYnMoeCA9IGxhYmVsLCB5ID0gIiIsIHNpemUgPSBsZWdlbmQpICsgDQogICAgICAgIGdncGxvdDI6OnRoZW1lKGF4aXMudGV4dC55ID0gZWxlbWVudF90ZXh0KHNpemUgPSAxMCwgDQogICAgICAgICAgICBjb2xvdXIgPSAiYmxhY2siLCBoanVzdCA9IDAuNSwgYW5nbGUgPSBhbmdsZSkpDQogICAgaWYgKGNiID09IFRSVUUpIHsNCiAgICAgICAgcGxvdCA8LSBwbG90ICsgc2NhbGVfZmlsbF9tYW51YWwodmFsdWVzID0gY2JwbCkgKyBzY2FsZV9jb2xvdXJfbWFudWFsKHZhbHVlcyA9IGNicGwpDQogICAgfQ0KICAgIGlmIChrID09IFRSVUUpIHsNCiAgICAgICAgcGxvdCA8LSBwbG90ICsgZ2dwbG90Mjo6YW5ub3RhdGUoInRleHQiLCB4ID0gKG1heChkYXRhJHlpKSArIA0KICAgICAgICAgICAgKG1heChkYXRhJHlpKSAqIDAuMSkpLCB5ID0gKHNlcSgxLCBncm91cF9ubywgMSkgKyANCiAgICAgICAgICAgIDAuMyksIGxhYmVsID0gcGFzdGUoIml0YWxpYyhOKT09IiwgbW9kX3RhYmxlJEspLCANCiAgICAgICAgICAgIHBhcnNlID0gVFJVRSwgaGp1c3QgPSAicmlnaHQiLCBzaXplID0gMy41KQ0KICAgIH0NCiAgICByZXR1cm4ocGxvdCkNCn0NCg0KDQoNCnBsMzwtb3JjaGFyZF9wbG90KG1lYW5fcGx1X3VyLCBtb2QgPSAicGx1IiwgYWxwaGEgPSAwLjA4LCB4bGFiID0gImxuUlIgLSB1bnJlc3RvcmVkL3Jlc3RvcmVkIikrIHRoZW1lKGxlZ2VuZC5wb3NpdGlvbiA9ICJub25lIikNCnBsMjwtb3JjaGFyZF9wbG90KHZyX3BsdV91ciwgbW9kID0gInBsdSIsIGFscGhhID0gMC4wOCx4bGFiID0gImxuVlIgLSB1bnJlc3RvcmVkL3Jlc3RvcmVkIikrIHRoZW1lKGxlZ2VuZC5wb3NpdGlvbiA9ICJub25lIikNCnBsMTwtb3JjaGFyZF9wbG90KGN2cl9wbHVfdXIsIG1vZCA9ICJwbHUiLCBhbHBoYSA9IDAuMDgseGxhYiA9ICJsbkNWUiAtIHVucmVzdG9yZWQvcmVzdG9yZWQiKSsgdGhlbWUobGVnZW5kLnBvc2l0aW9uID0gIm5vbmUiKQ0KDQpwbDY8LW9yY2hhcmRfcGxvdChtZWFuX3BsdV9yciwgbW9kID0gInBsdSIsIGFscGhhID0gMC4wOCx4bGFiID0gImxuUlIgLSByZWZlcmVuY2UvcmVzdG9yZWQiKSsgdGhlbWUobGVnZW5kLnBvc2l0aW9uID0gIm5vbmUiKQ0KcGw1PC1vcmNoYXJkX3Bsb3QodnJfcGx1X3JyLCBtb2QgPSAicGx1IiwgYWxwaGEgPSAwLjA4LHhsYWIgPSAibG5WUiAtIHJlZmVyZW5jZS9yZXN0b3JlZCIpKyB0aGVtZShsZWdlbmQucG9zaXRpb24gPSAibm9uZSIpDQpwbDQ8LW9yY2hhcmRfcGxvdChjdnJfcGx1X3JyLCBtb2QgPSAicGx1IiwgYWxwaGEgPSAwLjA4LHhsYWIgPSAibG5DVlIgLSByZWZlcmVuY2UvcmVzdG9yZWQiKSsgdGhlbWUobGVnZW5kLnBvc2l0aW9uID0gIm5vbmUiKQ0KDQoNCihwbDEgfCBwbDIgfCBwbDMpIC8gKHBsNCB8IHBsNSB8IHBsNikgK3Bsb3RfYW5ub3RhdGlvbih0YWdfbGV2ZWxzID0gIkEiKQ0KDQoNCg0KcGwxPC1wbDErIHNjYWxlX2ZpbGxfbWFudWFsKHZhbHVlcyA9IHJlcCgiZ3JlZW40IiwgNSkpICsgc2NhbGVfY29sb3VyX21hbnVhbCh2YWx1ZXMgPSByZXAoImdyZWVuNCIsIDUpKSsNCiAgICAgICB0aGVtZShheGlzLnRpdGxlLnkgPSBlbGVtZW50X2JsYW5rKCksDQogICAgICAgcGFuZWwuZ3JpZC5tYWpvciA9IGVsZW1lbnRfYmxhbmsoKSwNCiAgICAgICBwYW5lbC5ncmlkLm1pbm9yID0gZWxlbWVudF9ibGFuaygpLA0KICAgICAgIGF4aXMudGV4dC55ID0gZWxlbWVudF90ZXh0KGFuZ2xlPTApLA0KICAgICAgICkreGxhYigiTG9nIENWIHJhdGlvIChyZWxhdGl2ZSB0byB1bnJlc3RvcmVkKSIpDQoNCnBsMzwtcGwzKyBzY2FsZV9maWxsX21hbnVhbCh2YWx1ZXMgPSByZXAoInB1cnBsZSIsIDUpKSArIHNjYWxlX2NvbG91cl9tYW51YWwodmFsdWVzID0gcmVwKCJwdXJwbGUiLCA1KSkgKw0KICAgICAgIHRoZW1lKGF4aXMudGl0bGUueSA9IGVsZW1lbnRfYmxhbmsoKSwNCiAgICAgICBwYW5lbC5ncmlkLm1ham9yID0gZWxlbWVudF9ibGFuaygpLA0KICAgICAgIHBhbmVsLmdyaWQubWlub3IgPSBlbGVtZW50X2JsYW5rKCksDQogICAgICAgYXhpcy50ZXh0LnkgPSBlbGVtZW50X3RleHQoYW5nbGU9MCksDQogICAgICAgKSt4bGFiKCJMb2cgcmVzcG9uc2UgcmF0aW8gKHJlbGF0aXZlIHRvIHVucmVzdG9yZWQpIikNCg0KcGw2PC1wbDYrIHNjYWxlX2ZpbGxfbWFudWFsKHZhbHVlcyA9IHJlcCgicHVycGxlIiwgNSkpICsgc2NhbGVfY29sb3VyX21hbnVhbCh2YWx1ZXMgPSByZXAoInB1cnBsZSIsIDUpKSArDQogICAgICAgdGhlbWUoYXhpcy50aXRsZS55ID0gZWxlbWVudF9ibGFuaygpLA0KICAgICAgIHBhbmVsLmdyaWQubWFqb3IgPSBlbGVtZW50X2JsYW5rKCksDQogICAgICAgcGFuZWwuZ3JpZC5taW5vciA9IGVsZW1lbnRfYmxhbmsoKSwNCiAgICAgICBheGlzLnRleHQueSA9IGVsZW1lbnRfYmxhbmsoKSwNCiAgICAgICApK3hsYWIoIkxvZyByZXNwb25zZSByYXRpbyAocmVsYXRpdmUgdG8gcmVmZXJlbmNlKSIpDQoNCnBsNDwtcGw0KyBzY2FsZV9maWxsX21hbnVhbCh2YWx1ZXMgPSByZXAoImdyZWVuNCIsIDUpKSArIHNjYWxlX2NvbG91cl9tYW51YWwodmFsdWVzID0gcmVwKCJncmVlbjQiLCA1KSkrDQogICAgICAgdGhlbWUoYXhpcy50aXRsZS55ID0gZWxlbWVudF9ibGFuaygpLA0KICAgICAgIHBhbmVsLmdyaWQubWFqb3IgPSBlbGVtZW50X2JsYW5rKCksDQogICAgICAgcGFuZWwuZ3JpZC5taW5vciA9IGVsZW1lbnRfYmxhbmsoKSwNCiAgICAgICBheGlzLnRleHQueSA9IGVsZW1lbnRfYmxhbmsoKSwNCiAgICAgICApK3hsYWIoIkxvZyBDViByYXRpbyAocmVsYXRpdmUgdG8gcmVmZXJlbmNlKSIpDQoNCkZpZ3VyZTU8LSgocGwzICsgcGw2KSAvIChwbDEgKyBwbDQpKSArcGxvdF9hbm5vdGF0aW9uKHRhZ19wcmVmaXggPSAiKCIsIHRhZ19sZXZlbHMgPSAiYSIsIHRhZ19zdWZmaXggPSAiKSIpDQojZ2dzYXZlKCJGaWd1cmVfNS5wZGYiLCBGaWd1cmU1LCBoZWlnaHQgPSA4LjUsIHdpZHRoID0gMTEpDQoNCmBgYA0KDQoNCioqRmlndXJlIFM3KiogT3JjaGFyZCBwbG90cyBzaG93aW5nIHRoZSByZWxhdGlvbnNoaXAgYmV0d2VlbiByZXN0b3JhdGlvbiBzaXRlIHBhc3QgbGFuZCB1c2UgYW5kIHRoZSBiaW9kaXZlcnNpdHkgY2hhbmdlIGZvbGxvd2luZyByZXN0b3JhdGlvbiBjb21wYXJlZCB0byB1bnJlc3RvcmVkIGFuZCByZWZlcmVuY2UgbGV2ZWxzLg0KDQoNCioqVGFibGUgUzQuKiogRWZmZWN0IG9mIGFnZSBvZiByZXN0b3JhdGlvbiBzaXRlIG9uIHZhcmlhYmlsaXR5IG9mIGJpb2RpdmVyc2l0eSAobG5DVlIsIGxuVlIpIGFuZCBtZWFuIGJpb2RpdmVyc2l0eSAobG5SUikgY29tcGFyZWQgdG8gdW5yZXN0b3JlZCBhbmQgcmVmZXJlbmNlIGxldmVscw0KDQoNCmBgYHtyIH0NCg0KYWdlX2xpc3Q8LWxpc3QoY3ZyX2FnZV91ciwgY3ZyX2FnZV91cl9xLCB2cl9hZ2VfdXIsIHZyX2FnZV91cl9xLCBtZWFuX2FnZV91ciwgbWVhbl9hZ2VfdXJfcSwgY3ZyX2FnZV9yciwgY3ZyX2FnZV9ycl9xLCB2cl9hZ2VfcnIsIHZyX2FnZV9ycl9xLCBtZWFuX2FnZV9yciwgbWVhbl9hZ2VfcnJfcSkNCmFnZTwtbWFwKC54ID0gYWdlX2xpc3QsIC5mID0gdGlkeSkgDQphZ2VfcmVzPC1iaW5kX3Jvd3MoYWdlKQ0KbmFtZXM8LWMoImxuQ1ZSIHJlc3RvcmVkL3VucmVzdG9yZWQiLCAibG5DVlIgcmVzdG9yZWQvdW5yZXN0b3JlZCAtIHdpdGggcXVhZHJhdCBzaXplIiwibG5WUiByZXN0b3JlZC91bnJlc3RvcmVkIiwibG5WUiByZXN0b3JlZC91bnJlc3RvcmVkIC0gd2l0aCBxdWFkcmF0IHNpemUiLCJsblJSIHJlc3RvcmVkL3VucmVzdG9yZWQiLCJsblJSIHJlc3RvcmVkL3VucmVzdG9yZWQgLSB3aXRoIHF1YWRyYXQgc2l6ZSIsICJsbkNWUiByZXN0b3JlZC9yZWZlcmVuY2UiLCAibG5DVlIgcmVzdG9yZWQvcmVmZXJlbmNlIC0gd2l0aCBxdWFkcmF0IHNpemUiLCJsblZSIHJlc3RvcmVkL3JlZmVyZW5jZSIsImxuVlIgcmVzdG9yZWQvcmVmZXJlbmNlIC0gd2l0aCBxdWFkcmF0IHNpemUiLCJsblJSIHJlc3RvcmVkL3JlZmVyZW5jZSIsImxuUlIgcmVzdG9yZWQvcmVmZXJlbmNlIC0gd2l0aCBxdWFkcmF0IHNpemUiKQ0KYWdlX3JlczwtYWdlX3JlcyAlPiUgbXV0YXRlKG1vZGVsID0gcmVwKG5hbWVzLCB0aW1lcyA9IGMoMiwzLDIsMywyLDMsMiwzLDIsMywyLDMpKSkgJT4lIHNlbGVjdChtb2RlbCwgZXZlcnl0aGluZygpKQ0Ka2FibGUoYWdlX3JlcykgJT4lIGthYmxlX3N0eWxpbmcoKSU+JQ0KICAgIHNjcm9sbF9ib3god2lkdGggPSAiODAwcHgiLCBoZWlnaHQgPSAiMzAwcHgiKQ0KDQoNCmBgYA0KDQoqKlRhYmxlIFM1LioqIEVmZmVjdCBvZiBzaXplIChoYSkgb2YgcmVzdG9yYXRpb24gc2l0ZSBvbiB2YXJpYWJpbGl0eSBvZiBiaW9kaXZlcnNpdHkgKGxuQ1ZSLCBsblZSKSBhbmQgbWVhbiBiaW9kaXZlcnNpdHkgKGxuUlIpIGNvbXBhcmVkIHRvIHVucmVzdG9yZWQgYW5kIHJlZmVyZW5jZSBsZXZlbHMNCg0KDQpgYGB7ciB9DQoNCg0Kc2l6ZV9saXN0PC1saXN0KGN2cl9zaXplX3VyLCBjdnJfc2l6ZV91cl9xLCB2cl9zaXplX3VyLCB2cl9zaXplX3VyX3EsIG1lYW5fc2l6ZV91cixtZWFuX3NpemVfdXJfcSwgY3ZyX3NpemVfcnIsY3ZyX3NpemVfcnJfcSwgdnJfc2l6ZV9yciwgdnJfc2l6ZV9ycl9xLCBtZWFuX3NpemVfcnIsIG1lYW5fc2l6ZV9ycl9xKQ0KDQpzaXplPC1tYXAoLnggPSBzaXplX2xpc3QsIC5mID0gdGlkeSkgDQpzaXplX3JlczwtYmluZF9yb3dzKHNpemUpDQpuYW1lczwtYygibG5DVlIgcmVzdG9yZWQvdW5yZXN0b3JlZCIsICJsbkNWUiByZXN0b3JlZC91bnJlc3RvcmVkIC0gd2l0aCBxdWFkcmF0IHNpemUiLCJsblZSIHJlc3RvcmVkL3VucmVzdG9yZWQiLCJsblZSIHJlc3RvcmVkL3VucmVzdG9yZWQgLSB3aXRoIHF1YWRyYXQgc2l6ZSIsImxuUlIgcmVzdG9yZWQvdW5yZXN0b3JlZCIsImxuUlIgcmVzdG9yZWQvdW5yZXN0b3JlZCAtIHdpdGggcXVhZHJhdCBzaXplIiwgImxuQ1ZSIHJlc3RvcmVkL3JlZmVyZW5jZSIsICJsbkNWUiByZXN0b3JlZC9yZWZlcmVuY2UgLSB3aXRoIHF1YWRyYXQgc2l6ZSIsImxuVlIgcmVzdG9yZWQvcmVmZXJlbmNlIiwibG5WUiByZXN0b3JlZC9yZWZlcmVuY2UgLSB3aXRoIHF1YWRyYXQgc2l6ZSIsImxuUlIgcmVzdG9yZWQvcmVmZXJlbmNlIiwibG5SUiByZXN0b3JlZC9yZWZlcmVuY2UgLSB3aXRoIHF1YWRyYXQgc2l6ZSIpDQpzaXplX3Jlczwtc2l6ZV9yZXMgJT4lIG11dGF0ZShtb2RlbCA9IHJlcChuYW1lcywgdGltZXMgPSBjKDIsMywyLDMsMiwzLDIsMywyLDMsMiwzKSkpICU+JSBzZWxlY3QobW9kZWwsIGV2ZXJ5dGhpbmcoKSkNCmthYmxlKHNpemVfcmVzKSAlPiUga2FibGVfc3R5bGluZygpJT4lDQogICAgc2Nyb2xsX2JveCh3aWR0aCA9ICI4MDBweCIsIGhlaWdodCA9ICIzMDBweCIpDQoNCg0KYGBgDQoNCioqVGFibGUgUzYuKiogRWZmZWN0IG9mIHBhc3QgbGFuZCBzdGF0dXMgb2YgcmVzdG9yYXRpb24gc2l0ZSBvbiB2YXJpYWJpbGl0eSBvZiBiaW9kaXZlcnNpdHkgKGxuQ1ZSLCBsblZSKSBhbmQgbWVhbiBiaW9kaXZlcnNpdHkgKGxuUlIpIGNvbXBhcmVkIHRvIHVucmVzdG9yZWQgYW5kIHJlZmVyZW5jZSBsZXZlbHMNCg0KYGBge3IgfQ0KDQogDQpwbHVfbGlzdDwtbGlzdChjdnJfcGx1X3VyLCBjdnJfcGx1X3VyX3EsIHZyX3BsdV91ciwgdnJfcGx1X3VyX3EsIG1lYW5fcGx1X3VyLCBtZWFuX3BsdV91cl9xLCBjdnJfcGx1X3JyLCBjdnJfcGx1X3JyX3EsIHZyX3BsdV9yciwgdnJfcGx1X3JyX3EsIG1lYW5fcGx1X3JyLCBtZWFuX3BsdV9ycl9xKQ0KDQpwbHU8LW1hcCgueCA9IHBsdV9saXN0LCAuZiA9IHRpZHkpIA0KcGx1X3JlczwtYmluZF9yb3dzKHBsdSkNCm5hbWVzPC1jKCJsbkNWUiByZXN0b3JlZC91bnJlc3RvcmVkIiwgImxuQ1ZSIHJlc3RvcmVkL3VucmVzdG9yZWQgLSB3aXRoIHF1YWRyYXQgc2l6ZSIsImxuVlIgcmVzdG9yZWQvdW5yZXN0b3JlZCIsImxuVlIgcmVzdG9yZWQvdW5yZXN0b3JlZCAtIHdpdGggcXVhZHJhdCBzaXplIiwibG5SUiByZXN0b3JlZC91bnJlc3RvcmVkIiwibG5SUiByZXN0b3JlZC91bnJlc3RvcmVkIC0gd2l0aCBxdWFkcmF0IHNpemUiLCAibG5DVlIgcmVzdG9yZWQvcmVmZXJlbmNlIiwgImxuQ1ZSIHJlc3RvcmVkL3JlZmVyZW5jZSAtIHdpdGggcXVhZHJhdCBzaXplIiwibG5WUiByZXN0b3JlZC9yZWZlcmVuY2UiLCJsblZSIHJlc3RvcmVkL3JlZmVyZW5jZSAtIHdpdGggcXVhZHJhdCBzaXplIiwibG5SUiByZXN0b3JlZC9yZWZlcmVuY2UiLCJsblJSIHJlc3RvcmVkL3JlZmVyZW5jZSAtIHdpdGggcXVhZHJhdCBzaXplIikNCnBsdV9yZXM8LXBsdV9yZXMgJT4lIG11dGF0ZShtb2RlbCA9IHJlcChuYW1lcywgdGltZXMgPSBjKDUsNSw1LDUsNSw1LDUsNiw1LDYsNSw2KSkpICMlPiUgc2VsZWN0KG1vZGVsLCBldmVyeXRoaW5nKCkpDQprYWJsZShwbHVfcmVzKSAlPiUga2FibGVfc3R5bGluZygpJT4lDQogICAgc2Nyb2xsX2JveCh3aWR0aCA9ICI4MDBweCIsIGhlaWdodCA9ICIzMDBweCIpDQoNCg0KYGBgDQoNCg0KIyMgUHVibGljYXRpb24gYmlhcw0KDQpXZSB1c2VkIG11bHRpcGxlIGFwcHJvYWNoZXMgdG8gYXNzZXNzIHRoZSBlZmZlY3Qgb2YgcHVibGljYXRpb24gYmlhcyBvbiBvdXIgcmVzdWx0cy4gV2UgdXNlZCBhIG11bHRpbGV2ZWwgdmVyc2lvbiBvZiBFZ2dlcidzIHJlZ3Jlc3Npb24gd2l0aCB0aGUgc3F1YXJlLXJvb3Qgb2YgdGhlIHNhbXBsaW5nIHZhcmlhbmNlcyBhcyBhIG1vZGVyYXRvciB0byB0ZXN0IGZvciBhc3ltbWV0cnkgaW4gZnVubmVsIHBsb3RzIG9mIG1ldGEtYW5hbHl0aWMgbW9kZWxzIChOYWthZ2F3YSAmIFBvdWxpbiAyMDEyKS4gV2UgYWxzbyB1c2VkIHRyYWRpdGlvbmFsIGZ1bm5lbCBwbG90cyB0byB2aXN1YWxseSBhc3Nlc3MgYXN5bW1ldHJ5LiBJbnRlcmNlcHRzIGluIEVnZ2VyJ3MgcmVncmVzc2lvbnMgd2VyZSBkaWZmZXJlbnQgZnJvbSB6ZXJvIGluIGFsbCBtb2RlbHMsIHByb3ZpZGluZyBldmlkZW5jZSBmb3IgcHVibGljYXRpb24gYmlhcy4gV2UgYWRkZWQgdGhlIHB1YmxpY2F0aW9uIHllYXIgdG8gbWV0YS1yZWdyZXNzaW9ucyB0byB0ZXN0IGZvciB0aGUgZWZmZWN0IG9mIGEgdGltZS1sYWcgYmlhcyBvbiBvdXIgcmVzdWx0cyAoQXBwZW5kaXggUzIsIFRhYmxlIFM0LVM3KS4gVGhlIGVmZmVjdCBvZiBwdWJsaWNhdGlvbiB5ZWFyIHdhcyBub3QgcmVsYXRlZCB0byBlZmZlY3Qgc2l6ZXMgaW4gYW55IG1vZGVsIChBcHBlbmRpeCBTMiwgVGFibGUgUzQtUzcpLg0KDQoNCmBgYHtyIH0NCg0KDQojIyMjIyMjIyMjIyMjIyMjIyMjIyMjIyMjIyMjIyMjIyMjIyMjIyMjIyMjIyMjIyMjIyMjIyMjIyMjIyMNCiMjI0FORCBtYWpvcml0eSBvZiBjb2RlIGxpZnRlZCBmcm9tIGZyb20gSm9obnNvbiBldCBhbCAyMDIwIFwjIyMjDQojIyNTaWxpY29uIGlzIGEgZ2xvYmFsIHBsYW50IGRlZmVuY2UgYnV0IGVmZmVjdGl2ZW5lc3MjIyMjIyMgDQojIyNkZXBlbmRzIG9uIGhlcmJpdm9yZSBmZWVkaW5nIHN0cmF0ZWd5OiBhIG1ldGEtYW5hbHlzaXMiIyMNCg0KDQpsaWJyYXJ5KG1ldGFmb3IpDQpsaWJyYXJ5KGthYmxlRXh0cmEpDQpsaWJyYXJ5KGtuaXRyKQ0KbGlicmFyeShnZ3Bsb3QyKQ0KDQojdW5pdmFyaWF0ZSBlZ2dlciByZWdyZXNzb2luDQoNCmVnZ2VyX3VuaV91cl9jdnIgPC0gcm1hLm12KHlpID0geWlfY3ZyLCBWID0gdmlfY3ZyLCBtb2RzID0gfnNxcnQodmlfY3ZyKSwgdGVzdCA9ICJ0IiwgcmFuZG9tID0gbGlzdCh+MSB8IGlkLCB+MSB8IHBsb3RfaWQsIH4xIHwgdW5pdCksIG1ldGhvZCA9ICJSRU1MIiwgZGF0YSA9IHVuX3JlKQ0KZWdnZXJfdW5pX3VyX3ZyIDwtIHJtYS5tdih5aSA9IHlpX3ZyLCBWID0gdmlfdnIsIG1vZHMgPSB+c3FydCh2aV92ciksIHRlc3QgPSAidCIsIHJhbmRvbSA9IGxpc3QofjEgfCBpZCwgfjEgfCBwbG90X2lkLCB+MSB8IHVuaXQpLCBtZXRob2QgPSAiUkVNTCIsIGRhdGEgPSB1bl9yZSkNCmVnZ2VyX3VuaV91cl9tZWFuIDwtIHJtYS5tdih5aSA9IHlpX21lYW4sIFYgPSB2aV9tZWFuLCBtb2RzID0gfnNxcnQodmlfbWVhbiksIHRlc3QgPSAidCIsIHJhbmRvbSA9IGxpc3QofjEgfCBpZCwgfjEgfCBwbG90X2lkLCB+MSB8IHVuaXQpLCBtZXRob2QgPSAiUkVNTCIsIGRhdGEgPSB1bl9yZSkNCmVnZ2VyX3VuaV9ycl9jdnIgPC0gcm1hLm12KHlpID0geWlfY3ZyLCBWID0gdmlfY3ZyLCBtb2RzID0gfnNxcnQodmlfY3ZyKSwgdGVzdCA9ICJ0IiwgcmFuZG9tID0gbGlzdCh+MSB8IGlkLCB+MSB8IHBsb3RfaWQsIH4xIHwgdW5pdCksIG1ldGhvZCA9ICJSRU1MIiwgZGF0YSA9IHJlX3JlZikNCmVnZ2VyX3VuaV9ycl92ciA8LSBybWEubXYoeWkgPSB5aV92ciwgViA9IHZpX3ZyLCBtb2RzID0gfnNxcnQodmlfdnIpLCB0ZXN0ID0gInQiLCByYW5kb20gPSBsaXN0KH4xIHwgaWQsIH4xIHwgcGxvdF9pZCwgfjEgfCB1bml0KSwgbWV0aG9kID0gIlJFTUwiLCBkYXRhID0gcmVfcmVmKQ0KZWdnZXJfdW5pX3JyX21lYW4gPC0gcm1hLm12KHlpID0geWlfbWVhbiwgViA9IHZpX21lYW4sIG1vZHMgPSB+c3FydCh2aV9tZWFuKSwgdGVzdCA9ICJ0IiwgcmFuZG9tID0gbGlzdCh+MSB8IGlkLCB+MSB8IHBsb3RfaWQsIH4xIHwgdW5pdCksIG1ldGhvZCA9ICJSRU1MIiwgZGF0YSA9IHJlX3JlZikNCg0KDQpgYGANCg0KIyMjIFRyYWRpdGlvbmFsIGZ1bm5lbCBwbG90cw0KDQpgYGB7ciwgZmlnLmhlaWdodD0gMTAsIGZpZy53aWR0aD0xMH0NCiMjI1B1YmxpY2F0aW9uIGJpYXMgYW5hbHlzaXMgLSB1bnNjYWxlZCBtb2RlbHMNCiMgY2FuJ3QgZ2V0IHRoaXMgdG8gcHJpbnQgbmVhdGx5LCBmb3Igbm93PyBzb21ldGhpbmcgdG8gZG8gd2l0aCBtZXRhZm9yOjpmdW5uZWwgPyBudm0gdGhpcyB3b3JrczoNCnBhcihtZnJvdz1jKDMsMikpIA0KDQpmdW5uZWwoZWdnZXJfdW5pX3VyX2N2ciwgeWF4aXMgPSAic2VpbnYiLCBsZXZlbCA9IGMoOTAsIDk1LCA5OSksIHNoYWRlID0gYygid2hpdGUiLCAiZ3JheTU1IiwgImdyYXk3NSIpLCByZWZsaW5lID0gMCwgbGVnZW5kID0gVFJVRSkNCmZ1bm5lbChlZ2dlcl91bmlfdXJfdnIsIHlheGlzID0gInNlaW52IiwgbGV2ZWwgPSBjKDkwLCA5NSwgOTkpLCBzaGFkZSA9IGMoIndoaXRlIiwgImdyYXk1NSIsICJncmF5NzUiKSwgcmVmbGluZSA9IDAsIGxlZ2VuZCA9IFRSVUUpDQpmdW5uZWwoZWdnZXJfdW5pX3VyX21lYW4sIHlheGlzID0gInNlaW52IiwgbGV2ZWwgPSBjKDkwLCA5NSwgOTkpLCBzaGFkZSA9IGMoIndoaXRlIiwgImdyYXk1NSIsICJncmF5NzUiKSwgcmVmbGluZSA9IDAsIGxlZ2VuZCA9IFRSVUUpDQpmdW5uZWwoZWdnZXJfdW5pX3JyX2N2ciwgeWF4aXMgPSAic2VpbnYiLCBsZXZlbCA9IGMoOTAsIDk1LCA5OSksIHNoYWRlID0gYygid2hpdGUiLCAiZ3JheTU1IiwgImdyYXk3NSIpLCByZWZsaW5lID0gMCwgbGVnZW5kID0gVFJVRSkNCmZ1bm5lbChlZ2dlcl91bmlfcnJfdnIsIHlheGlzID0gInNlaW52IiwgbGV2ZWwgPSBjKDkwLCA5NSwgOTkpLCBzaGFkZSA9IGMoIndoaXRlIiwgImdyYXk1NSIsICJncmF5NzUiKSwgcmVmbGluZSA9IDAsIGxlZ2VuZCA9IFRSVUUpDQpmdW5uZWwoZWdnZXJfdW5pX3JyX21lYW4sIHlheGlzID0gInNlaW52IiwgbGV2ZWwgPSBjKDkwLCA5NSwgOTkpLCBzaGFkZSA9IGMoIndoaXRlIiwgImdyYXk1NSIsICJncmF5NzUiKSwgcmVmbGluZSA9IDAsIGxlZ2VuZCA9IFRSVUUpDQoNCg0KYGBgDQoNCioqRmlndXJlIFM4KiogRnVubmVsIHBsb3RzIHRlc3RpbmcgZm9yIGFzeW1tZXRyeSBmb3IgYWxsIG1vZGVscyBhbmQgZWZmZWN0IHNpemVzLiBBID0gcmVzdG9yZWQvdW5yZXN0b3JlZCBsbkNWUiwgQiA9IHJlc3RvcmVkL3VucmVzdG9yZWQgbG5WUiwgQyA9IHJlc3RvcmVkL3VucmVzdG9yZWQgbG5SUiwgRCA9IHJlc3RvcmVkL3JlZmVyZW5jZSBsbkNWUiwgRSA9IHJlc3RvcmVkL3JlZmVyZW5jZSBsblZSLCBGID0gcmVzdG9yZWQvcmVmZXJlbmNlIGxuUlINCg0KIyMjIFBsb3Qgb2YgdW5pdmFyaWF0ZSBlZ2dlciByZWdyZXNzaW9ucw0KDQpgYGB7ciwgZmlnLmhlaWdodD0gMTAsIGZpZy53aWR0aD0xMH0NCnVuaTE8LXVuaV9lZ2dlcl9wbG90X2N2cihlZ2dlcl91bmlfdXJfY3ZyLCBkYXRhID0gdW5fcmUpK3lsYWIoImxuQ1ZSIChlZmZlY3Qgc2l6ZSIpDQp1bmkyPC11bmlfZWdnZXJfcGxvdF92cihlZ2dlcl91bmlfdXJfdnIsIGRhdGEgPSB1bl9yZSkreWxhYigibG5WUiAoZWZmZWN0IHNpemUiKQ0KdW5pMzwtdW5pX2VnZ2VyX3Bsb3RfbWVhbihlZ2dlcl91bmlfdXJfbWVhbiwgZGF0YSA9IHVuX3JlKQ0KdW5pNDwtdW5pX2VnZ2VyX3Bsb3RfY3ZyKGVnZ2VyX3VuaV9ycl9jdnIsIGRhdGEgPSByZV9yZWYpK3lsYWIoImxuQ1ZSIChlZmZlY3Qgc2l6ZSIpDQp1bmk1PC11bmlfZWdnZXJfcGxvdF92cihlZ2dlcl91bmlfcnJfdnIsIGRhdGEgPSByZV9yZWYpK3lsYWIoImxuVlIgKGVmZmVjdCBzaXplIikNCnVuaTY8LXVuaV9lZ2dlcl9wbG90X21lYW4oZWdnZXJfdW5pX3JyX21lYW4sIGRhdGEgPSByZV9yZWYpDQoNCih1bmkxICsgdW5pMiArIHVuaTMpIC8gKHVuaTQgKyB1bmk1ICsgdW5pNikgKyBwbG90X2Fubm90YXRpb24odGFnX2xldmVscyA9ICJBIikNCg0KYGBgDQoNCioqRmlndXJlIFM5LioqIFBsb3Qgb2YgdW5pdmFyaWF0ZSBlZ2dlciByZWdyZXNzaW9uIGZvciBhbGwgbW9kZWxzIGFuZCBlZmZlY3Qgc2l6ZXMNCg0KIyMgVGltZS1sYWcgYmlhcw0KDQoqKlRhYmxlIFM3LioqIFJlbGF0aW9uc2hpcCBiZXR3ZWVuIHB1YmxpY2F0aW9uIHllYXIgYW5kIGVmZmVjdCBzaXplIChsblJSIC0gcmVzdG9yZWQvdW5yZXN0b3JlZCkNCg0KYGBge3J9DQoNCiMjIyMjIyMjIyMjIyMjIyMjIw0KDQp0aW1lX2xhZ19lZmZlY3RfdW5pX2xuUlIgPC0gcm1hLm12KHlpID0geWlfbWVhbiwgViA9IHZjdl9tZWFuLCBtb2RzID0gflllYXIsIHRlc3QgPSAidCIsIA0KICAgICAgICAgICAgICAgICAgICAgICAgICAgICAgICAgICByYW5kb20gPSBsaXN0KH4xIHwgaWQsIH4xIHwgc2hhcmVkX2N0cmwsIH4xIHwgdW5pdCksIG1ldGhvZCA9ICJSRU1MIiwgZGF0YSA9IHVuX3JlKQ0KIyBnZXR0aW5nIG1hcmdpbmFsIFIyDQpyMl90aW1lX2xhZ19lZmZlY3RfdW5pX2xuUlIgPC0gcjJfbWwodGltZV9sYWdfZWZmZWN0X3VuaV9sblJSKQ0KIyBnZXR0aW5nIGVzdGltYXRlczogbmFtZSBkb2VzIG5vdCB3b3JrIGZvciBzbG9wZXMNCnJlc190aW1lX2xhZ19lZmZlY3RfdW5pX2xuUlIgPC0gZ2V0X2VzdCh0aW1lX2xhZ19lZmZlY3RfdW5pX2xuUlIsIG1vZCA9ICJZZWFyIikNCiMgY3JlYXRpbmcgYSB0YWJsZQ0KdGliYmxlKGBGaXhlZCBlZmZlY3RgID0gcm93Lm5hbWVzKHRpbWVfbGFnX2VmZmVjdF91bmlfbG5SUiRiZXRhKSwgRXN0aW1hdGUgPSBjKHJlc190aW1lX2xhZ19lZmZlY3RfdW5pX2xuUlIkZXN0aW1hdGUpLCANCiAgICAgICBgTG93ZXIgQ0kgWzAuMDI1XWAgPSBjKHJlc190aW1lX2xhZ19lZmZlY3RfdW5pX2xuUlIkbG93ZXJDTCksIGBVcHBlciBDSSAgWzAuOTc1XWAgPSBjKHJlc190aW1lX2xhZ19lZmZlY3RfdW5pX2xuUlIkdXBwZXJDTCksIA0KICAgICAgIGBQIHZhbHVlYCA9IHRpbWVfbGFnX2VmZmVjdF91bmlfbG5SUiRwdmFsLCBSMiA9IGMocjJfdGltZV9sYWdfZWZmZWN0X3VuaV9sblJSWzFdLCANCiAgICAgICAgICAgICAgICAgICAgICAgICAgICAgICAgICAgICAgICAgICAgICAgICAgICAgICAgIE5BKSkgJT4lIGthYmxlKCJodG1sIiwgZGlnaXRzID0gMykgJT4lIGthYmxlX3N0eWxpbmcoInN0cmlwZWQiLCBwb3NpdGlvbiA9ICJsZWZ0IikNCmBgYA0KDQoqKlRhYmxlIFM4LioqIFJlbGF0aW9uc2hpcCBiZXR3ZWVuIHB1YmxpY2F0aW9uIHllYXIgYW5kIGVmZmVjdCBzaXplIChsbkNWUiAtIHJlc3RvcmVkL3VucmVzdG9yZWQpDQoNCmBgYHtyfQ0KDQojIyMjIyMjIyMjIyMjIyMjIyMNCg0KdGltZV9sYWdfZWZmZWN0X3VuaV9sbkNWUiA8LSBybWEubXYoeWkgPSB5aV9jdnIsIFYgPSB2Y3ZfY3ZyLCBtb2RzID0gflllYXIsIHRlc3QgPSAidCIsIA0KICAgICAgICAgICAgICAgICAgICAgICAgICAgICAgICAgICByYW5kb20gPSBsaXN0KH4xIHwgaWQsIH4xIHwgc2hhcmVkX2N0cmwsIH4xIHwgdW5pdCksIG1ldGhvZCA9ICJSRU1MIiwgZGF0YSA9IHVuX3JlKQ0KIyBnZXR0aW5nIG1hcmdpbmFsIFIyDQpyMl90aW1lX2xhZ19lZmZlY3RfdW5pX2xuQ1ZSIDwtIHIyX21sKHRpbWVfbGFnX2VmZmVjdF91bmlfbG5DVlIpDQojIGdldHRpbmcgZXN0aW1hdGVzOiBuYW1lIGRvZXMgbm90IHdvcmsgZm9yIHNsb3Blcw0KcmVzX3RpbWVfbGFnX2VmZmVjdF91bmlfbG5DVlIgPC0gZ2V0X2VzdCh0aW1lX2xhZ19lZmZlY3RfdW5pX2xuQ1ZSLCBtb2QgPSAiWWVhciIpDQojIGNyZWF0aW5nIGEgdGFibGUNCnRpYmJsZShgRml4ZWQgZWZmZWN0YCA9IHJvdy5uYW1lcyh0aW1lX2xhZ19lZmZlY3RfdW5pX2xuQ1ZSJGJldGEpLCBFc3RpbWF0ZSA9IGMocmVzX3RpbWVfbGFnX2VmZmVjdF91bmlfbG5DVlIkZXN0aW1hdGUpLCANCiAgICAgICBgTG93ZXIgQ0kgWzAuMDI1XWAgPSBjKHJlc190aW1lX2xhZ19lZmZlY3RfdW5pX2xuQ1ZSJGxvd2VyQ0wpLCBgVXBwZXIgQ0kgIFswLjk3NV1gID0gYyhyZXNfdGltZV9sYWdfZWZmZWN0X3VuaV9sbkNWUiR1cHBlckNMKSwgDQogICAgICAgYFAgdmFsdWVgID0gdGltZV9sYWdfZWZmZWN0X3VuaV9sbkNWUiRwdmFsLCBSMiA9IGMocjJfdGltZV9sYWdfZWZmZWN0X3VuaV9sbkNWUlsxXSwgDQogICAgICAgICAgICAgICAgICAgICAgICAgICAgICAgICAgICAgICAgICAgICAgICAgICAgICAgICBOQSkpICU+JSBrYWJsZSgiaHRtbCIsIGRpZ2l0cyA9IDMpICU+JSBrYWJsZV9zdHlsaW5nKCJzdHJpcGVkIiwgcG9zaXRpb24gPSAibGVmdCIpDQpgYGANCg0KKipUYWJsZSBTOS4qKiBSZWxhdGlvbnNoaXAgYmV0d2VlbiBwdWJsaWNhdGlvbiB5ZWFyIGFuZCBlZmZlY3Qgc2l6ZSAobG5SUiAtIHJlc3RvcmVkL3JlZmVyZW5jZSkNCg0KYGBge3J9DQogDQojIyMjIyMjIyMjIyMjIyMjIyMNCg0KdGltZV9sYWdfZWZmZWN0X3VuaV9sblJScmVyZWYgPC0gcm1hLm12KHlpID0geWlfbWVhbiwgViA9IHZjdl9tZWFuX3JyLCBtb2RzID0gflllYXIsIHRlc3QgPSAidCIsIA0KICAgICAgICAgICAgICAgICAgICAgICAgICAgICAgICAgICByYW5kb20gPSBsaXN0KH4xIHwgaWQsIH4xIHwgc2hhcmVkX2N0cmwsIH4xIHwgdW5pdCksIG1ldGhvZCA9ICJSRU1MIiwgZGF0YSA9IHJlX3JlZikNCiMgZ2V0dGluZyBtYXJnaW5hbCBSMg0KcjJfdGltZV9sYWdfZWZmZWN0X3VuaV9sblJScmVyZWYgPC0gcjJfbWwodGltZV9sYWdfZWZmZWN0X3VuaV9sblJScmVyZWYpDQojIGdldHRpbmcgZXN0aW1hdGVzOiBuYW1lIGRvZXMgbm90IHdvcmsgZm9yIHNsb3Blcw0KcmVzX3RpbWVfbGFnX2VmZmVjdF91bmlfbG5SUnJlcmVmIDwtIGdldF9lc3QodGltZV9sYWdfZWZmZWN0X3VuaV9sblJScmVyZWYsIG1vZCA9ICJZZWFyIikNCiMgY3JlYXRpbmcgYSB0YWJsZQ0KdGliYmxlKGBGaXhlZCBlZmZlY3RgID0gcm93Lm5hbWVzKHRpbWVfbGFnX2VmZmVjdF91bmlfbG5SUnJlcmVmJGJldGEpLCBFc3RpbWF0ZSA9IGMocmVzX3RpbWVfbGFnX2VmZmVjdF91bmlfbG5SUnJlcmVmJGVzdGltYXRlKSwgDQogICAgICAgYExvd2VyIENJIFswLjAyNV1gID0gYyhyZXNfdGltZV9sYWdfZWZmZWN0X3VuaV9sblJScmVyZWYkbG93ZXJDTCksIGBVcHBlciBDSSAgWzAuOTc1XWAgPSBjKHJlc190aW1lX2xhZ19lZmZlY3RfdW5pX2xuUlJyZXJlZiR1cHBlckNMKSwgDQogICAgICAgYFAgdmFsdWVgID0gdGltZV9sYWdfZWZmZWN0X3VuaV9sblJScmVyZWYkcHZhbCwgUjIgPSBjKHIyX3RpbWVfbGFnX2VmZmVjdF91bmlfbG5SUnJlcmVmWzFdLCANCiAgICAgICAgICAgICAgICAgICAgICAgICAgICAgICAgICAgICAgICAgICAgICAgICAgICAgICAgIE5BKSkgJT4lIGthYmxlKCJodG1sIiwgZGlnaXRzID0gMykgJT4lIGthYmxlX3N0eWxpbmcoInN0cmlwZWQiLCBwb3NpdGlvbiA9ICJsZWZ0IikNCmBgYA0KDQoqKlRhYmxlIFMxMC4qKiBSZWxhdGlvbnNoaXAgYmV0d2VlbiBwdWJsaWNhdGlvbiB5ZWFyIGFuZCBlZmZlY3Qgc2l6ZSAobG5SUiAtIHJlc3RvcmVkL3JlZmVyZW5jZSkNCg0KYGBge3J9DQoNCiMjIyMjIyMjIyMjIyMjIyMjIw0KDQp0aW1lX2xhZ19lZmZlY3RfdW5pX2xuQ1ZScmVyZWYgPC0gcm1hLm12KHlpID0geWlfY3ZyLCBWID0gdmN2X2N2cl9yciwgbW9kcyA9IH5ZZWFyLCB0ZXN0ID0gInQiLCANCiAgICAgICAgICAgICAgICAgICAgICAgICAgICAgICAgICAgcmFuZG9tID0gbGlzdCh+MSB8IGlkLCB+MSB8IHNoYXJlZF9jdHJsLCB+MSB8IHVuaXQpLCBtZXRob2QgPSAiUkVNTCIsIGRhdGEgPSByZV9yZWYpDQojIGdldHRpbmcgbWFyZ2luYWwgUjINCnIyX3RpbWVfbGFnX2VmZmVjdF91bmlfbG5SUnJlcmVmIDwtIHIyX21sKHRpbWVfbGFnX2VmZmVjdF91bmlfbG5SUnJlcmVmKQ0KIyBnZXR0aW5nIGVzdGltYXRlczogbmFtZSBkb2VzIG5vdCB3b3JrIGZvciBzbG9wZXMNCnJlc190aW1lX2xhZ19lZmZlY3RfdW5pX2xuUlJyZXJlZiA8LSBnZXRfZXN0KHRpbWVfbGFnX2VmZmVjdF91bmlfbG5SUnJlcmVmLCBtb2QgPSAiWWVhciIpDQojIGNyZWF0aW5nIGEgdGFibGUNCnRpYmJsZShgRml4ZWQgZWZmZWN0YCA9IHJvdy5uYW1lcyh0aW1lX2xhZ19lZmZlY3RfdW5pX2xuUlJyZXJlZiRiZXRhKSwgRXN0aW1hdGUgPSBjKHJlc190aW1lX2xhZ19lZmZlY3RfdW5pX2xuUlJyZXJlZiRlc3RpbWF0ZSksIA0KICAgICAgIGBMb3dlciBDSSBbMC4wMjVdYCA9IGMocmVzX3RpbWVfbGFnX2VmZmVjdF91bmlfbG5SUnJlcmVmJGxvd2VyQ0wpLCBgVXBwZXIgQ0kgIFswLjk3NV1gID0gYyhyZXNfdGltZV9sYWdfZWZmZWN0X3VuaV9sblJScmVyZWYkdXBwZXJDTCksIA0KICAgICAgIGBQIHZhbHVlYCA9IHRpbWVfbGFnX2VmZmVjdF91bmlfbG5SUnJlcmVmJHB2YWwsIFIyID0gYyhyMl90aW1lX2xhZ19lZmZlY3RfdW5pX2xuUlJyZXJlZlsxXSwgDQogICAgICAgICAgICAgICAgICAgICAgICAgICAgICAgICAgICAgICAgICAgICAgICAgICAgICAgICBOQSkpICU+JSBrYWJsZSgiaHRtbCIsIGRpZ2l0cyA9IDMpICU+JSBrYWJsZV9zdHlsaW5nKCJzdHJpcGVkIiwgcG9zaXRpb24gPSAibGVmdCIpDQpgYGANCg0KIyMgU2NhbGUgZGVwZW5kZW5jeQ0KDQpXZSBmb2xsb3cgcmVjb21tZW5kYXRpb25zIGJ5IFNwYWtlIGV0IGFsLiAoMjAyMCkgd2hlcmUgcG9zc2libGUgdG8gcmVkdWNlIHRoZSBzY2FsZSBiaWFzIGluIG91ciBtZXRhLWFuYWx5c2VzLiBGaXJzdGx5LCB3ZSB1c2UgdGhlIGxvZyByZXNwb25zZSByYXRpbyB3aGljaCBoYXMgYmVlbiBzaG93biB0byBiZSBtb3JlIHJvYnVzdCBpbiBzY2VuYXJpb3Mgb2YgY3Jvc3Mtc3R1ZHkgc3ludGhlc2VzIGF0IHZhcnlpbmcgc3BhdGlhbCBncmFpbiwgY29tcGFyZWQgd2l0aCBIZWRnZXMnIGcgKFNwYWtlIGV0IGFsLiAyMDIwKSAuIE91ciBkYXRhIGRvZXMgbm90IGFsbG93IHRoZSB1c2Ugb2YgYXN5bXB0b3RpYyBtZWFzdXJlcyBvZiByaWNobmVzcyAoZS5nLiByYXJlZmllZCByaWNobmVzcykgc2luY2Ugb25seSBhIHZlcnkgc21hbGwgbWlub3JpdHkgb2Ygc3R1ZGllcyByZXBvcnQgYmlvZGl2ZXJzaXR5IGluIHRoaXMgbWFubmVyLiBIb3dldmVyLCB3ZSBydW4gYWxsIG1vZGVscyBpbmNsdWRpbmcgdGhlIHF1YWRyYXQgc2l6ZSBhcyBhIHRlcm0gaW4gYWxsIG91ciBtb2RlbHMgYW5kIGNvbXBhcmUgdG8gdGhlIHJlc3VsdHMgd2l0aG91dCB0aGlzIHRlcm0uIFRoZSBzaWduaWZpY2FuY2Ugb3Igbm9uLXNpZ25pZmljYW5jZSBvZiBhbGwgcmVzdWx0cyByZXBvcnRlZCB3ZXJlIG5vdCBjaGFuZ2VkIHdoZW4gbW9kZWxzIHdlcmUgcnVuIG9uIHRoZSByZWR1Y2VkIGRhdGFzZXQgbW9kZWxzIGluY2x1ZGluZyBxdWFkcmF0IHNpemVzIHdoZXJlIGF2YWlsYWJsZS4gVGhvdWdoLCB0aGUgbmF0dXJlIG9mIG1hbnkgYmlvZGl2ZXJzaXR5IHNhbXBsaW5nIG1ldGhvZHMgKGUuZy4gcGl0ZmFsbCB0cmFwcywgYnV0dGVyZmx5IG5ldHMsIHRyYW5zZWN0cykgYXJlIG5vdCBjb21wYXJhYmxlIGluIG0yIG1lYW5pbmcgdGhhdCB0aGVzZSBkaWFnbm9zdGljIG1vZGVscyBleGNsdWRlIDIwLTMwJSBvZiB0aGUgZGF0YSBwb2ludHMuIFRoZXJlZm9yZSwgdGhlIG1vZGVsIHJlc3VsdHMgcHJlc2VudGVkIGluIHRoZSBtYWluIHRleHQgYXJlIGV4Y2x1ZGluZyBxdWFkcmF0IHNpemUuDQoNClRoZSBwbG90cyBiZWxvdyBzaG93IHRoYXQgb3VyIGVmZmVjdCBzaXplcyB3b3VsZCBoYXZlIGJlZW4gc2Vuc2l0aXZlIHRvIHNpemUgaGFkIHdlIHVzZWQgSGVkZ2VzICpnKiwgaG93ZXZlciB0aGV5IHNob3cgbGl0dGxlIHJlc3BvbnNlIHRvIHNjYWxlIHdoZW4gdXNpbmcgdGhlIGxvZyByZXNwb25zZSByYXRpbyBvciBsb2cgQ1YgcmF0aW8uDQoNCmBgYHtyIH0NCg0KZHVnIDwtIHJlYWQuY3N2KCJEYXRhL3ZhcmlhdGlvbl9kYXRhLmNzdiIpI3B1dCBkYXRhIGZpbGUgaGVyZQ0KDQpkdWdsciA8LSBtZXRhZm9yOjplc2NhbGMobWVhc3VyZT0iUk9NIixtMWk9ZHVnJHRfbWVhbiwgbTJpPWR1ZyRjX21lYW4sIHNkMWk9ZHVnJHRfc2QsIHNkMmk9Y19zZCwgbjFpPWR1ZyR0X3F1YWRfbiwgbjJpPWR1ZyRjX3F1YWRfbiwgYXBwZW5kPVQsIGRhdGE9ZHVnKSAjbm9uLWVxdWFsIHZhcmlhbmNlcyANCg0KI2xuQ1ZSDQpkdWdjdnIgPC0gbWV0YWZvcjo6ZXNjYWxjKG1lYXN1cmU9IkNWUiIsbTFpPWR1ZyR0X21lYW4sIG0yaT1kdWckY19tZWFuLCBzZDFpPWR1ZyR0X3NkLCBzZDJpPWNfc2QsIG4xaT1kdWckdF9xdWFkX24sIG4yaT1kdWckY19xdWFkX24sIGFwcGVuZD1ULCBkYXRhPWR1ZykgDQoNCiNIZWRnZXMnIGcNCmR1Z2hnIDwtIG1ldGFmb3I6OmVzY2FsYyhtZWFzdXJlPSJTTUQiLG0xaT1kdWckdF9tZWFuLCBtMmk9ZHVnJGNfbWVhbiwgc2QxaT1kdWckdF9zZCwgc2QyaT1jX3NkLCBuMWk9ZHVnJHRfcXVhZF9uLCBuMmk9ZHVnJGNfcXVhZF9uLCBhcHBlbmQ9VCwgZGF0YT1kdWcpIA0KDQojYWx0ZXJuYXRpdmUgdmFyaWFuY2UgZXN0aW1hdGUgZm9yIGcNCm4xPWFzLm51bWVyaWMoZHVnJHRfcXVhZF9uKTsgbjI9YXMubnVtZXJpYyhkdWckY19xdWFkX24pDQpuX3RpbGRlPW4yKm4xLyhuMituMSkNCnZhcl9kX24uREVOUz0oKDEtMy8oNCoobjIrbjEtMiktMSkpXjIpKihuMituMS0yKS8obl90aWxkZSoobjIrbjEtNCkpICNIZWRnZXMgdmFyaWFuY2UgdGhhdCBkb2VzIG5vdCBjb250YWluIGQgaHR0cHM6Ly9lc2Fqb3VybmFscy5vbmxpbmVsaWJyYXJ5LndpbGV5LmNvbS9kb2kvZnVsbC8xMC4xMDAyL2VjczIuMjQxOQ0KDQpkdWdoZyR2aTI8LSB2YXJfZF9uLkRFTlMNCg0KdW5pdCA8LSBmYWN0b3IoMTpsZW5ndGgoZHVnbHIkeWkpKQ0KZHVnbHIkdW5pdCA8LSB1bml0DQoNCnVuaXQgPC0gZmFjdG9yKDE6bGVuZ3RoKGR1Z2N2ciR5aSkpDQpkdWdjdnIkdW5pdCA8LSB1bml0DQoNCnVuaXQgPC0gZmFjdG9yKDE6bGVuZ3RoKGR1Z2hnJHlpKSkNCmR1Z2hnJHVuaXQgPC0gdW5pdA0KDQpkdWdscjwtZHVnbHIgJT4lIGRyb3BfbmEoYyhpZCwgcGxvdF9pZCwgdW5pdCkpDQpkdWdjdnI8LWR1Z2xyICU+JSBkcm9wX25hKGMoaWQsIHBsb3RfaWQsIHVuaXQpKQ0KZHVnaGc8LWR1Z2hnICU+JSBkcm9wX25hKGMoaWQsIHBsb3RfaWQsIHVuaXQpKQ0KDQpkdWdscjwtZHVnbHIgJT4lIGRyb3BfbmEodF9xc2l6ZV9tMikNCmR1Z2N2cjwtZHVnbHIgJT4lIGRyb3BfbmEodF9xc2l6ZV9tMikNCmR1Z2hnPC1kdWdoZyAlPiUgZHJvcF9uYSh0X3FzaXplX20yKQ0KDQojcmFuZG9tLWVmZmVjdHMsIGNvbnZlbnRpb25hbCB3ZWlnaHRlZCBtZXRhLWFuYWx5c2lzDQpsci5tYS5yYW4gPC0gcm1hLm12KHlpPXlpLCBWPXZpLCBkYXRhPWR1Z2xyLCBtZXRob2Q9IlJFTUwiLCByYW5kb20gPSBsaXN0KH4xIHwgaWQsIH4xIHwgcGxvdF9pZCwgfjEgfCB1bml0KSkNCiN1bndlaWdodGVkIA0KbHIubWEudW4gPC0gcm1hLm12KHlpPXlpLCBWPXZpLCBkYXRhPWR1Z2xyLCBtZXRob2Q9IlJFTUwiLCByYW5kb209IGxpc3QofjEgfCBpZCwgfjEgfCBwbG90X2lkLCB+MSB8IHVuaXQpLCBXPTEpDQojZml4ZWQtZWZmZWN0cywgY29udmVudGlvbmFsIHdlaWdodGVkIG1ldGEtYW5hbHlzaXMNCmxyLm1hLmZpeCA8LSBybWEubXYoeWk9eWksIFY9dmksIGRhdGE9ZHVnbHIsIG1ldGhvZD0iUkVNTCIsIHJhbmRvbT0gbGlzdCh+MSB8IGlkLCB+MSB8IHBsb3RfaWQsIH4xIHwgdW5pdCksIFcgPSAxL3ZpKQ0KDQojcmFuZG9tLWVmZmVjdHMsIGNvbnZlbnRpb25hbCB3ZWlnaHRlZCBtZXRhLWFuYWx5c2lzDQpjdnIubWEucmFuIDwtIHJtYS5tdih5aT15aSwgVj12aSwgZGF0YT1kdWdjdnIsIG1ldGhvZD0iUkVNTCIsIHJhbmRvbSA9IGxpc3QofjEgfCBpZCwgfjEgfCBwbG90X2lkLCB+MSB8IHVuaXQpKQ0KI3Vud2VpZ2h0ZWQgDQpjdnIubWEudW4gPC0gcm1hLm12KHlpPXlpLCBWPXZpLCBkYXRhPWR1Z2N2ciwgbWV0aG9kPSJSRU1MIiwgcmFuZG9tPSBsaXN0KH4xIHwgaWQsIH4xIHwgcGxvdF9pZCwgfjEgfCB1bml0KSwgVz0xKQ0KI2ZpeGVkLWVmZmVjdHMsIGNvbnZlbnRpb25hbCB3ZWlnaHRlZCBtZXRhLWFuYWx5c2lzDQpjdnIubWEuZml4IDwtIHJtYS5tdih5aT15aSwgVj12aSwgZGF0YT1kdWdjdnIsIG1ldGhvZD0iUkVNTCIsIHJhbmRvbT0gbGlzdCh+MSB8IGlkLCB+MSB8IHBsb3RfaWQsIH4xIHwgdW5pdCksIFcgPSAxL3ZpKQ0KDQojcmFuZG9tLWVmZmVjdHMsIGNvbnZlbnRpb25hbCB3ZWlnaHRlZCBtZXRhLWFuYWx5c2lzDQpoZy5tYS5yYW4gPC0gcm1hLm12KHlpPXlpLCBWPXZpLCBkYXRhPWR1Z2hnLCBtZXRob2Q9IlJFTUwiLCByYW5kb209IGxpc3QofjEgfCBpZCwgfjEgfCBwbG90X2lkLCB+MSB8IHVuaXQpKQ0KI3Vud2VpZ2h0ZWQgDQpoZy5tYS51biA8LSBybWEubXYoeWk9eWksIFY9dmksIGRhdGE9ZHVnaGcsIG1ldGhvZD0iUkVNTCIsIHJhbmRvbT0gbGlzdCh+MSB8IGlkLCB+MSB8IHBsb3RfaWQsIH4xIHwgdW5pdCksIFc9MSkNCiNmaXhlZC1lZmZlY3RzLCBjb252ZW50aW9uYWwgd2VpZ2h0ZWQgbWV0YS1hbmFseXNpcw0KaGcubWEuZml4IDwtIHJtYS5tdih5aT15aSwgVj12aSwgZGF0YT1kdWdoZywgbWV0aG9kPSJSRU1MIiwgcmFuZG9tPSBsaXN0KH4xIHwgaWQsIH4xIHwgcGxvdF9pZCwgfjEgfCB1bml0KSwgVyA9IDEvdmkpDQoNCmhnLm1hLnJhbi5kX2FsdCA8LSBybWEubXYoeWk9eWksIFY9dmkyLCBkYXRhPWR1Z2hnLCBtZXRob2Q9IlJFTUwiLCByYW5kb209IGxpc3QofjEgfCBpZCwgfjEgfCBwbG90X2lkLCB+MSB8IHVuaXQpKQ0KDQpoZy5tYS5maXguZGFsdCA8LSBybWEubXYoeWk9eWksIFY9dmkyLCBkYXRhPWR1Z2hnLCBtZXRob2Q9IlJFTUwiLCByYW5kb209IGxpc3QofjEgfCBpZCwgfjEgfCBwbG90X2lkLCB+MSB8IHVuaXQpLCBXID0gMS92aTIpDQoNCmBgYA0KDQojIyMgRXhwbG9yYXRvcnkgYnViYmxlIHBsb3RzDQoNCmBgYHtyLCBmaWcuaGVpZ2h0PTgsIGZpZy53aWR0aD04fQ0KDQpkdWdscjwtZHVnbHIgJT4lIG11dGF0ZShpZCA9IGFzLmZhY3RvcihpZCkpICMgY3VycmVudGx5IHRoaW5rcyBpZCBpcyBudW1lcmljIC0gaXMgdGhhdCBhbiBpc3N1ZT8NCmR1Z2N2cjwtZHVnY3ZyICU+JSBtdXRhdGUoaWQgPSBhcy5mYWN0b3IoaWQpKSAjIGN1cnJlbnRseSB0aGlua3MgaWQgaXMgbnVtZXJpYyAtIGlzIHRoYXQgYW4gaXNzdWU/DQpkdWdoZzwtZHVnaGcgJT4lIG11dGF0ZShpZCA9IGFzLmZhY3RvcihpZCkpICMgY3VycmVudGx5IHRoaW5rcyBpZCBpcyBudW1lcmljIC0gaXMgdGhhdCBhbiBpc3N1ZT8NCg0KTnZzQS5MUiA8LSBnZ3Bsb3QoZHVnbHIpICsgZ2VvbV9wb2ludChhZXMoeD1sb2codF9xc2l6ZV9tMi8xMDAwMCksIHk9dF9xdWFkX24sIHNpemU9MS92aSxjb2w9aWQsYWxwaGE9MC4zKSkrIHRoZW1lX2J3KCkgKyB0aGVtZShwYW5lbC5ncmlkLm1ham9yID0gZWxlbWVudF9ibGFuaygpLA0KcGFuZWwuZ3JpZC5taW5vciA9IGVsZW1lbnRfYmxhbmsoKSwgYXhpcy5saW5lID0gZWxlbWVudF9saW5lKGNvbG91ciA9ICJibGFjayIpKSsgeWxhYihleHByZXNzaW9uKGl0YWxpYyhOKSkpK3hsYWIoIkxvZyhQbG90IHNpemUgKGhhKSkiKStnZW9tX2hsaW5lKHlpbnRlcmNlcHQgPSAwLCBsaW5ldHlwZSA9ICJkYXNoZWQiKSArIHNjYWxlX3NpemVfY29udGludW91cyhuYW1lID0gZXhwcmVzc2lvbihwYXN0ZSgiMS8iLGl0YWxpYyhWKSkpLGxhYmVscz1OVUxMKStzY2FsZV9jb2xvcl9kaXNjcmV0ZSgiIiwgZ3VpZGU9Rikrc2NhbGVfYWxwaGEoIiIsZ3VpZGU9RikrDQogICAgdGhlbWUocGxvdC50aXRsZSA9IGVsZW1lbnRfdGV4dChzaXplID0gOCkpK3RoZW1lKGF4aXMudGV4dC54PWVsZW1lbnRfdGV4dChzaXplPXJlbCgwLjcpKSkNCg0KTnZzQS5DVlIgPC0gZ2dwbG90KGR1Z2N2cikgKyBnZW9tX3BvaW50KGFlcyh4PWxvZyh0X3FzaXplX20yLzEwMDAwKSwgeT10X3F1YWRfbiwgc2l6ZT0xL3ZpLGNvbD1pZCxhbHBoYT0wLjMpKSsgdGhlbWVfYncoKSArIHRoZW1lKHBhbmVsLmdyaWQubWFqb3IgPSBlbGVtZW50X2JsYW5rKCksDQpwYW5lbC5ncmlkLm1pbm9yID0gZWxlbWVudF9ibGFuaygpLCBheGlzLmxpbmUgPSBlbGVtZW50X2xpbmUoY29sb3VyID0gImJsYWNrIikpKyB5bGFiKGV4cHJlc3Npb24oaXRhbGljKE4pKSkreGxhYigiTG9nKFBsb3Qgc2l6ZSAoaGEpKSIpK2dlb21faGxpbmUoeWludGVyY2VwdCA9IDAsIGxpbmV0eXBlID0gImRhc2hlZCIpICsgc2NhbGVfc2l6ZV9jb250aW51b3VzKG5hbWUgPSBleHByZXNzaW9uKHBhc3RlKCIxLyIsaXRhbGljKFYpKSksbGFiZWxzPU5VTEwpK3NjYWxlX2NvbG9yX2Rpc2NyZXRlKCIiLCBndWlkZT1GKStzY2FsZV9hbHBoYSgiIixndWlkZT1GKSsNCiAgICB0aGVtZShwbG90LnRpdGxlID0gZWxlbWVudF90ZXh0KHNpemUgPSA4KSkrdGhlbWUoYXhpcy50ZXh0Lng9ZWxlbWVudF90ZXh0KHNpemU9cmVsKDAuNykpKQ0KDQpOdnNBLmcgPC0gZ2dwbG90KGR1Z2hnKSArIGdlb21fcG9pbnQoYWVzKHg9bG9nKHRfcXNpemVfbTIvMTAwMDApLCB5PXRfcXVhZF9uLCBzaXplPTEvdmksIGNvbG9yPWlkLGFscGhhPTAuMykpKyB0aGVtZV9idygpICsgdGhlbWUocGFuZWwuZ3JpZC5tYWpvciA9IGVsZW1lbnRfYmxhbmsoKSwNCnBhbmVsLmdyaWQubWlub3IgPSBlbGVtZW50X2JsYW5rKCksIGF4aXMubGluZSA9IGVsZW1lbnRfbGluZShjb2xvdXIgPSAiYmxhY2siKSkrIHlsYWIoZXhwcmVzc2lvbihpdGFsaWMoTikpKSt4bGFiKCJMb2coUGxvdCBzaXplIChoYSkpIikrZ2VvbV9obGluZSh5aW50ZXJjZXB0ID0gMCwgbGluZXR5cGUgPSAiZGFzaGVkIikgKyBzY2FsZV9zaXplX2NvbnRpbnVvdXMobmFtZSA9IGV4cHJlc3Npb24ocGFzdGUoIjEvIixpdGFsaWMoVikpKSxsYWJlbHM9TlVMTCkrc2NhbGVfY29sb3JfZGlzY3JldGUoIiIsIGd1aWRlPUYpK3NjYWxlX2FscGhhKCIiLGd1aWRlPUYpKyANCiAgICB0aGVtZShwbG90LnRpdGxlID0gZWxlbWVudF90ZXh0KHNpemUgPSA4KSkrdGhlbWUoYXhpcy50ZXh0Lng9ZWxlbWVudF90ZXh0KHNpemU9cmVsKDAuNykpKQ0KDQoNCkxSdnNBIDwtIGdncGxvdChkdWdscikgKyBnZW9tX3BvaW50KGFlcyh4PWxvZyh0X3FzaXplX20yLzEwMDAwKSwgeT15aSwgc2l6ZT0xL3ZpLCBjb2xvcj1pZCxhbHBoYT0wLjMpKSsgdGhlbWVfYncoKSArIHRoZW1lKHBhbmVsLmdyaWQubWFqb3IgPSBlbGVtZW50X2JsYW5rKCksDQpwYW5lbC5ncmlkLm1pbm9yID0gZWxlbWVudF9ibGFuaygpLCBheGlzLmxpbmUgPSBlbGVtZW50X2xpbmUoY29sb3VyID0gImJsYWNrIikpKyB5bGFiKGV4cHJlc3Npb24oaXRhbGljKCJMUiIpKSkreGxhYigiTG9nKFBsb3Qgc2l6ZSAoaGEpKSIpK2dlb21faGxpbmUoeWludGVyY2VwdCA9IDAsIGxpbmV0eXBlID0gImRhc2hlZCIpICsgc2NhbGVfc2l6ZV9jb250aW51b3VzKG5hbWUgPSBleHByZXNzaW9uKHBhc3RlKCIxLyIsaXRhbGljKFYpKSksbGFiZWxzPU5VTEwpK3NjYWxlX2NvbG9yX2Rpc2NyZXRlKCJzdHVkeSIpK3NjYWxlX2NvbG9yX2Rpc2NyZXRlKCIiLCBndWlkZT1GKStzY2FsZV9hbHBoYSgiIixndWlkZT1GKSsgDQogICAgdGhlbWUocGxvdC50aXRsZSA9IGVsZW1lbnRfdGV4dChzaXplID0gOCkpK3RoZW1lKGF4aXMudGV4dC54PWVsZW1lbnRfdGV4dChzaXplPXJlbCgwLjcpKSkNCg0KQ1ZSdnNBIDwtIGdncGxvdChkdWdjdnIpICsgZ2VvbV9wb2ludChhZXMoeD1sb2codF9xc2l6ZV9tMi8xMDAwMCksIHk9eWksIHNpemU9MS92aSwgY29sb3I9aWQsYWxwaGE9MC4zKSkrIHRoZW1lX2J3KCkgKyB0aGVtZShwYW5lbC5ncmlkLm1ham9yID0gZWxlbWVudF9ibGFuaygpLA0KcGFuZWwuZ3JpZC5taW5vciA9IGVsZW1lbnRfYmxhbmsoKSwgYXhpcy5saW5lID0gZWxlbWVudF9saW5lKGNvbG91ciA9ICJibGFjayIpKSsgeWxhYihleHByZXNzaW9uKGl0YWxpYygiQ1ZSIikpKSt4bGFiKCJMb2coUGxvdCBzaXplIChoYSkpIikrZ2VvbV9obGluZSh5aW50ZXJjZXB0ID0gMCwgbGluZXR5cGUgPSAiZGFzaGVkIikgKyBzY2FsZV9zaXplX2NvbnRpbnVvdXMobmFtZSA9IGV4cHJlc3Npb24ocGFzdGUoIjEvIixpdGFsaWMoVikpKSxsYWJlbHM9TlVMTCkrc2NhbGVfY29sb3JfZGlzY3JldGUoInN0dWR5Iikrc2NhbGVfY29sb3JfZGlzY3JldGUoIiIsIGd1aWRlPUYpK3NjYWxlX2FscGhhKCIiLGd1aWRlPUYpKyANCiAgICB0aGVtZShwbG90LnRpdGxlID0gZWxlbWVudF90ZXh0KHNpemUgPSA4KSkrdGhlbWUoYXhpcy50ZXh0Lng9ZWxlbWVudF90ZXh0KHNpemU9cmVsKDAuNykpKQ0KDQoNCg0KZ3ZzQSA8LSAgZ2dwbG90KGR1Z2hnKSArIGdlb21fcG9pbnQoYWVzKHg9bG9nKHRfcXNpemVfbTIvMTAwMDApLCB5PXlpLCBzaXplPTEvdmksIGNvbG9yPWlkLGFscGhhPTAuMykpKyB0aGVtZV9idygpICsgdGhlbWUocGFuZWwuZ3JpZC5tYWpvciA9IGVsZW1lbnRfYmxhbmsoKSwNCnBhbmVsLmdyaWQubWlub3IgPSBlbGVtZW50X2JsYW5rKCksIGF4aXMubGluZSA9IGVsZW1lbnRfbGluZShjb2xvdXIgPSAiYmxhY2siKSkrIHlsYWIoZXhwcmVzc2lvbihpdGFsaWMoZykpKSt4bGFiKCJMb2coUGxvdCBzaXplIChoYSkpIikrZ2VvbV9obGluZSh5aW50ZXJjZXB0ID0gMCwgbGluZXR5cGUgPSAiZGFzaGVkIikgK3NjYWxlX3NpemVfY29udGludW91cyhuYW1lID0gZXhwcmVzc2lvbihwYXN0ZSgiMS8iLGl0YWxpYyhWKSkpLGxhYmVscz1OVUxMKStzY2FsZV9jb2xvcl9kaXNjcmV0ZSgiIiwgZ3VpZGU9Rikrc2NhbGVfYWxwaGEoIiIsZ3VpZGU9RikrIA0KICAgIHRoZW1lKHBsb3QudGl0bGUgPSBlbGVtZW50X3RleHQoc2l6ZSA9IDgpKSt0aGVtZShheGlzLnRleHQueD1lbGVtZW50X3RleHQoc2l6ZT1yZWwoMC43KSkpDQoNCg0KTFIuVmFydnNBIDwtIGdncGxvdChkdWdscikgKyBnZW9tX3BvaW50KGFlcyh4PWxvZyh0X3FzaXplX20yLzEwMDAwKSwgeT12aSwgc2l6ZT10X3F1YWRfbiwgY29sb3I9aWQsYWxwaGE9MC4zKSkrIHRoZW1lX2J3KCkgKyB0aGVtZShwYW5lbC5ncmlkLm1ham9yID0gZWxlbWVudF9ibGFuaygpLA0KcGFuZWwuZ3JpZC5taW5vciA9IGVsZW1lbnRfYmxhbmsoKSwgYXhpcy5saW5lID0gZWxlbWVudF9saW5lKGNvbG91ciA9ICJibGFjayIpKSsgeWxhYihleHByZXNzaW9uKHBhc3RlKCJWYXJpYW5jZSBvZiAiLGl0YWxpYyhMUikpKSkreGxhYigiTG9nKFBsb3Qgc2l6ZSAoaGEpKSIpK2dlb21faGxpbmUoeWludGVyY2VwdCA9IDAsIGxpbmV0eXBlID0gImRhc2hlZCIpICtzY2FsZV9zaXplX2NvbnRpbnVvdXMobmFtZSA9IGV4cHJlc3Npb24oaXRhbGljKE4pKSxsYWJlbHM9TlVMTCkrc2NhbGVfY29sb3JfZGlzY3JldGUoIiIsIGd1aWRlPUYpK3NjYWxlX2FscGhhKCIiLGd1aWRlPUYpKyANCiAgICB0aGVtZShwbG90LnRpdGxlID0gZWxlbWVudF90ZXh0KHNpemUgPSA4KSkrdGhlbWUoYXhpcy50ZXh0Lng9ZWxlbWVudF90ZXh0KHNpemU9cmVsKDAuNykpKQ0KDQpDVlIuVmFydnNBIDwtIGdncGxvdChkdWdjdnIpICsgZ2VvbV9wb2ludChhZXMoeD1sb2codF9xc2l6ZV9tMi8xMDAwMCksIHk9dmksIHNpemU9dF9xdWFkX24sIGNvbG9yPWlkLGFscGhhPTAuMykpKyB0aGVtZV9idygpICsgdGhlbWUocGFuZWwuZ3JpZC5tYWpvciA9IGVsZW1lbnRfYmxhbmsoKSwNCnBhbmVsLmdyaWQubWlub3IgPSBlbGVtZW50X2JsYW5rKCksIGF4aXMubGluZSA9IGVsZW1lbnRfbGluZShjb2xvdXIgPSAiYmxhY2siKSkrIHlsYWIoZXhwcmVzc2lvbihwYXN0ZSgiVmFyaWFuY2Ugb2YgIixpdGFsaWMoQ1ZSKSkpKSt4bGFiKCJMb2coUGxvdCBzaXplIChoYSkpIikrZ2VvbV9obGluZSh5aW50ZXJjZXB0ID0gMCwgbGluZXR5cGUgPSAiZGFzaGVkIikgK3NjYWxlX3NpemVfY29udGludW91cyhuYW1lID0gZXhwcmVzc2lvbihpdGFsaWMoTikpLGxhYmVscz1OVUxMKStzY2FsZV9jb2xvcl9kaXNjcmV0ZSgiIiwgZ3VpZGU9Rikrc2NhbGVfYWxwaGEoIiIsZ3VpZGU9RikrIA0KICAgIHRoZW1lKHBsb3QudGl0bGUgPSBlbGVtZW50X3RleHQoc2l6ZSA9IDgpKSt0aGVtZShheGlzLnRleHQueD1lbGVtZW50X3RleHQoc2l6ZT1yZWwoMC43KSkpDQoNCmcuVmFydnNBIDwtIGdncGxvdChkdWdoZykgKyBnZW9tX3BvaW50KGFlcyh4PWxvZyh0X3FzaXplX20yLzEwMDAwKSwgeT12aSwgc2l6ZT10X3F1YWRfbiwgY29sb3I9aWQsYWxwaGE9MC4zKSkrIHRoZW1lX2J3KCkgKyB0aGVtZShwYW5lbC5ncmlkLm1ham9yID0gZWxlbWVudF9ibGFuaygpLA0KcGFuZWwuZ3JpZC5taW5vciA9IGVsZW1lbnRfYmxhbmsoKSwgYXhpcy5saW5lID0gZWxlbWVudF9saW5lKGNvbG91ciA9ICJibGFjayIpKSsgeWxhYihleHByZXNzaW9uKHBhc3RlKCJWYXJpYW5jZSBvZiAiLGl0YWxpYyhnKSkpKSt4bGFiKCJMb2coUGxvdCBzaXplIChoYSkpIikrZ2VvbV9obGluZSh5aW50ZXJjZXB0ID0gMCwgbGluZXR5cGUgPSAiZGFzaGVkIikgK3NjYWxlX3NpemVfY29udGludW91cyhuYW1lID0gZXhwcmVzc2lvbihpdGFsaWMoTikpLGxhYmVscz1OVUxMKStzY2FsZV9jb2xvcl9kaXNjcmV0ZSgiIiwgZ3VpZGU9Rikrc2NhbGVfYWxwaGEoIiIsZ3VpZGU9RikrIA0KICAgIHRoZW1lKHBsb3QudGl0bGUgPSBlbGVtZW50X3RleHQoc2l6ZSA9IDgpKSt0aGVtZShheGlzLnRleHQ9ZWxlbWVudF90ZXh0KHNpemU9cmVsKDAuNykpKQ0KDQpsaWJyYXJ5KGdyaWRFeHRyYSkNCg0KZ3JpZEV4dHJhOjpncmlkLmFycmFuZ2UoTnZzQS5nLCBndnNBLCBnLlZhcnZzQSxOdnNBLkxSLCAgTFJ2c0EsTFIuVmFydnNBLCBOdnNBLkNWUiwgIENWUnZzQSxDVlIuVmFydnNBLCBuY29sPTMpDQoNCmBgYA0KDQoqKkZpZ3VyZSBTMTAgQnViYmxlIHBsb3Qgb2YgcGxvdCBzaXplIGFnYWluc3QgdmFyaW91cyBlZmZlY3Qgc2l6ZXMgKGxhYmVsbGVkKSoqDQoNCmBgYHtyLCBmaWcuaGVpZ2h0PSAzLCBmaWcud2lkdGg9IDUsIGV2YWw9RkFMU0V9DQpoZGVmZiA8LSBkYXRhLmZyYW1lKGVzdGltYXRlPWMoaGcubWEucmFuJGIsaGcubWEudW4kYixoZy5tYS5maXgkYixoZy5tYS5yYW4uZF9hbHQkYixoZy5tYS5maXguZGFsdCRiKSxjaS51cD1jKGhnLm1hLnJhbiRjaS51YixoZy5tYS51biRjaS51YixoZy5tYS5maXgkY2kudWIsIGhnLm1hLnJhbi5kX2FsdCRjaS51YixoZy5tYS5maXguZGFsdCRjaS51YiksIGNpLmxvPWMoaGcubWEucmFuJGNpLmxiLGhnLm1hLnVuJGNpLmxiLGhnLm1hLmZpeCRjaS5sYixoZy5tYS5yYW4uZF9hbHQkY2kubGIsaGcubWEuZml4LmRhbHQkY2kubGIpLCB3ZWlnaHRpbmc9YygiUiIsICJVIiwgIkYiLCAiUiIsICJGIiksIHZ0eXBlPWMoImQiLCJkIiwiZCIsImRfYWx0IiwiZF9hbHQiKSkNCg0KaGRlZmYgPC0gaGRlZmYgJT4lDQphcnJhbmdlKHdlaWdodGluZykgJT4lICAgICMgRmlyc3Qgc29ydCBieSB2YWwuIFRoaXMgc29ydCB0aGUgZGF0YWZyYW1lIGJ1dCBOT1QgdGhlIGZhY3RvciBsZXZlbHMNCiAgbXV0YXRlKHdlaWdodGluZz1mYWN0b3Iod2VpZ2h0aW5nLCBsZXZlbHM9DQogICAgICAgICAgICAgICAgICAgICAgICAgICAgYygiUiIsICJGIiwgIlUiKSkpICAgICMgVGhpcyB0cmljayB1cGRhdGUgdGhlIGZhY3RvciBsZXZlbHMNCg0KaGRlZmYucC5hIDwtZ2dwbG90KGhkZWZmKSArIGdlb21fcG9pbnQoYWVzKHkgPSBlc3RpbWF0ZSwgeCA9IHdlaWdodGluZywgY29sb3VyPXZ0eXBlKSwgcG9zaXRpb249cG9zaXRpb25fZG9kZ2Uod2lkdGggPSAwLjUpKSArIGdlb21fZXJyb3JiYXIoYWVzKHg9d2VpZ2h0aW5nLHltaW49Y2kubG8sIHltYXg9Y2kudXAsIHdpZHRoID0gMC4xLCBjb2xvdXI9dnR5cGUpLCwgcG9zaXRpb249cG9zaXRpb25fZG9kZ2Uod2lkdGggPSAwLjUpKSsNCiAgIGdlb21fYWJsaW5lKGludGVyY2VwdCA9IDAsIHNsb3BlPTAsIGNvbG91ciA9ICJkYXJrZ3JheSIsIGxpbmV0eXBlPSJkYXNoZWQiLCkgKw0KICB4bGFiKE5VTEwpICsNCiAgeWxhYihleHByZXNzaW9uKGl0YWxpYyhnKSkpKw0KICB0aGVtZV9idygpICsgDQogIHRoZW1lKHBsb3QudGl0bGUgPSBlbGVtZW50X3RleHQoc2l6ZT0xMCksYXhpcy50aXRsZSA9IGVsZW1lbnRfdGV4dChzaXplPTEwKSxheGlzLnRleHQgPSBlbGVtZW50X3RleHQoc2l6ZT05KSApDQpoZGVmZi5wLmEgPC1oZGVmZi5wLmEgK3RoZW1lKGF4aXMudGV4dC54ID0gZWxlbWVudF90ZXh0KGFuZ2xlPTAsZmFjZSA9IGMocmVwKCdwbGFpbicsMTYpLCAnYm9sZCcsICdib2xkJykpKSArc2NhbGVfY29sb3JfbWFudWFsKHZhbHVlcz1jKCJibGFjayIsICJkYXJrZ3JheSIpKQ0KDQpscmVmZiA8LSBkYXRhLmZyYW1lKGVzdGltYXRlPWMobHIubWEucmFuJGIsbHIubWEudW4kYixsci5tYS5maXgkYiksY2kudXA9Yyhsci5tYS5yYW4kY2kudWIsbHIubWEudW4kY2kudWIsbHIubWEuZml4JGNpLnViKSwgY2kubG89Yyhsci5tYS5yYW4kY2kubGIsbHIubWEudW4kY2kubGIsbHIubWEuZml4JGNpLmxiKSwgd2VpZ2h0aW5nPWMoIlIiLCAiVSIsICJGIikpDQpscmVmZiA8LSBscmVmZiAlPiUNCmFycmFuZ2Uod2VpZ2h0aW5nKSAlPiUgICAgIyBGaXJzdCBzb3J0IGJ5IHZhbC4gVGhpcyBzb3J0IHRoZSBkYXRhZnJhbWUgYnV0IE5PVCB0aGUgZmFjdG9yIGxldmVscw0KICBtdXRhdGUod2VpZ2h0aW5nPWZhY3Rvcih3ZWlnaHRpbmcsIGxldmVscz0NCiAgICAgICAgICAgICAgICAgICAgICAgICAgICBjKCJSIiwgIkYiLCAiVSIpKSkgICAgIyBUaGlzIHRyaWNrIHVwZGF0ZSB0aGUgZmFjdG9yIGxldmVscw0KDQoNCmxyZWZmLnAuYSA8LWdncGxvdChscmVmZikgKyBnZW9tX3BvaW50KGFlcyh5ID0gZXN0aW1hdGUsIHggPSB3ZWlnaHRpbmcpKSArIGdlb21fZXJyb3JiYXIoYWVzKHg9d2VpZ2h0aW5nLHltaW49Y2kubG8sIHltYXg9Y2kudXAsIHdpZHRoID0gMC4xKSkrDQogIGdlb21fYWJsaW5lKGludGVyY2VwdCA9IDAsIHNsb3BlPTAsIGNvbG91ciA9ICJkYXJrZ3JheSIsIGxpbmV0eXBlPSJkYXNoZWQiKSArDQogIHhsYWIoTlVMTCkgKw0KIHlsYWIoZXhwcmVzc2lvbihpdGFsaWMoTFIpKSkrDQogIHRoZW1lX2J3KCkgKyANCiAgdGhlbWUocGxvdC50aXRsZSA9IGVsZW1lbnRfdGV4dChzaXplPTEwKSxheGlzLnRpdGxlID0gZWxlbWVudF90ZXh0KHNpemU9MTApLGF4aXMudGV4dCA9IGVsZW1lbnRfdGV4dChzaXplPTkpICkNCmxyZWZmLnAuYSA8LWxyZWZmLnAuYSArdGhlbWUoYXhpcy50ZXh0LnggPSBlbGVtZW50X3RleHQoYW5nbGU9MCxmYWNlID0gYyhyZXAoJ3BsYWluJywxNiksICdib2xkJywgJ2JvbGQnKSkpDQoNCmN2cmVmZiA8LSBkYXRhLmZyYW1lKGVzdGltYXRlPWMoY3ZyLm1hLnJhbiRiLGN2ci5tYS51biRiLGN2ci5tYS5maXgkYiksY2kudXA9YyhjdnIubWEucmFuJGNpLnViLGN2ci5tYS51biRjaS51YixjdnIubWEuZml4JGNpLnViKSwgY2kubG89YyhjdnIubWEucmFuJGNpLmxiLGN2ci5tYS51biRjaS5sYixjdnIubWEuZml4JGNpLmxiKSwgd2VpZ2h0aW5nPWMoIlIiLCAiVSIsICJGIikpDQpjdnJlZmYgPC0gY3ZyZWZmICU+JQ0KYXJyYW5nZSh3ZWlnaHRpbmcpICU+JSAgICAjIEZpcnN0IHNvcnQgYnkgdmFsLiBUaGlzIHNvcnQgdGhlIGRhdGFmcmFtZSBidXQgTk9UIHRoZSBmYWN0b3IgbGV2ZWxzDQogIG11dGF0ZSh3ZWlnaHRpbmc9ZmFjdG9yKHdlaWdodGluZywgbGV2ZWxzPQ0KICAgICAgICAgICAgICAgICAgICAgICAgICAgIGMoIlIiLCAiRiIsICJVIikpKSAgICAjIFRoaXMgdHJpY2sgdXBkYXRlIHRoZSBmYWN0b3IgbGV2ZWxzDQoNCg0KY3ZyZWZmLnAuYSA8LWdncGxvdChjdnJlZmYpICsgZ2VvbV9wb2ludChhZXMoeSA9IGVzdGltYXRlLCB4ID0gd2VpZ2h0aW5nKSkgKyBnZW9tX2Vycm9yYmFyKGFlcyh4PXdlaWdodGluZyx5bWluPWNpLmxvLCB5bWF4PWNpLnVwLCB3aWR0aCA9IDAuMSkpKw0KICBnZW9tX2FibGluZShpbnRlcmNlcHQgPSAwLCBzbG9wZT0wLCBjb2xvdXIgPSAiZGFya2dyYXkiLCBsaW5ldHlwZT0iZGFzaGVkIikgKw0KICB4bGFiKE5VTEwpICsNCiB5bGFiKGV4cHJlc3Npb24oaXRhbGljKGN2cikpKSsNCiAgdGhlbWVfYncoKSArIA0KICB0aGVtZShwbG90LnRpdGxlID0gZWxlbWVudF90ZXh0KHNpemU9MTApLGF4aXMudGl0bGUgPSBlbGVtZW50X3RleHQoc2l6ZT0xMCksYXhpcy50ZXh0ID0gZWxlbWVudF90ZXh0KHNpemU9OSkgKQ0KY3ZyZWZmLnAuYSA8LWN2cmVmZi5wLmEgK3RoZW1lKGF4aXMudGV4dC54ID0gZWxlbWVudF90ZXh0KGFuZ2xlPTAsZmFjZSA9IGMocmVwKCdwbGFpbicsMTYpLCAnYm9sZCcsICdib2xkJykpKQ0KDQoNCg0KZ3JpZEV4dHJhOjpncmlkLmFycmFuZ2UoaGRlZmYucC5hLCBscmVmZi5wLmEsIGN2cmVmZi5wLmEsIG5jb2w9Mywgd2lkdGhzID0gYygzLCAyLjMsIDIuMykpDQpgYGANCg0KDQoNCmBgYHtyLCBmaWcuaGVpZ2h0PSAzLCBmaWcud2lkdGg9IDV9DQojTFINCiNyYW5kb20sIGNvbnZlbnRpb25hbGx5IHdlaWdodGVkDQpsci5tYS5yYW4gPC0gcm1hLm12KHlpPXNjYWxlKHlpKSwgVj12aSwgbW9kcz1sb2codF9xc2l6ZV9tMiksIGRhdGE9ZHVnbHIsIG1ldGhvZD0iUkVNTCIsIHJhbmRvbT0gbGlzdCh+MSB8IGlkLCB+MSB8IHBsb3RfaWQsIH4xIHwgdW5pdCkpDQojIHVud2VpZ2h0ZWQgDQpsci5tYS51biA8LSBybWEubXYoeWk9c2NhbGUoeWkpLCBWPXZpLCBtb2RzPWxvZyh0X3FzaXplX20yKSwgZGF0YT1kdWdsciwgbWV0aG9kPSJSRU1MIiwgcmFuZG9tPSBsaXN0KH4xIHwgaWQsIH4xIHwgcGxvdF9pZCwgfjEgfCB1bml0KSwgVz0xKQ0KI2ZpeGVkIGVmZmVjdCANCmxyLm1hLmZpeCA8LSBybWEubXYoeWk9c2NhbGUoeWkpLCBWPXZpLCBtb2RzPWxvZyh0X3FzaXplX20yKSwgZGF0YT1kdWdsciwgbWV0aG9kPSJSRU1MIiwgcmFuZG9tPSBsaXN0KH4xIHwgaWQsIH4xIHwgcGxvdF9pZCwgfjEgfCB1bml0KSwgVyA9IDEvdmkpDQoNCg0KI2N2cg0KI3JhbmRvbSwgY29udmVudGlvbmFsbHkgd2VpZ2h0ZWQNCmN2ci5tYS5yYW4gPC0gcm1hLm12KHlpPXNjYWxlKHlpKSwgVj12aSwgbW9kcz1sb2codF9xc2l6ZV9tMiksIGRhdGE9ZHVnY3ZyLCBtZXRob2Q9IlJFTUwiLCByYW5kb209IGxpc3QofjEgfCBpZCwgfjEgfCBwbG90X2lkLCB+MSB8IHVuaXQpKQ0KIyB1bndlaWdodGVkIA0KY3ZyLm1hLnVuIDwtIHJtYS5tdih5aT1zY2FsZSh5aSksIFY9dmksIG1vZHM9bG9nKHRfcXNpemVfbTIpLCBkYXRhPWR1Z2N2ciwgbWV0aG9kPSJSRU1MIiwgcmFuZG9tPSBsaXN0KH4xIHwgaWQsIH4xIHwgcGxvdF9pZCwgfjEgfCB1bml0KSwgVz0xKQ0KI2ZpeGVkIGVmZmVjdCANCmN2ci5tYS5maXggPC0gcm1hLm12KHlpPXNjYWxlKHlpKSwgVj12aSwgbW9kcz1sb2codF9xc2l6ZV9tMiksIGRhdGE9ZHVnY3ZyLCBtZXRob2Q9IlJFTUwiLCByYW5kb209IGxpc3QofjEgfCBpZCwgfjEgfCBwbG90X2lkLCB+MSB8IHVuaXQpLCBXID0gMS92aSkNCg0KI0hHDQojcmFuZG9tLCBjb252ZW50aW9uYWxseSB3ZWlnaHRlZA0KaGcubWEucmFuIDwtIHJtYS5tdih5aT1zY2FsZSh5aSksIFY9dmksIG1vZHM9bG9nKHRfcXNpemVfbTIpLCBkYXRhPWR1Z2hnLCBtZXRob2Q9IlJFTUwiLCByYW5kb209IGxpc3QofjEgfCBpZCwgfjEgfCBwbG90X2lkLCB+MSB8IHVuaXQpKQ0KIyB1bndlaWdodGVkIA0KaGcubWEudW4gPC0gcm1hLm12KHlpPXNjYWxlKHlpKSwgVj12aSwgbW9kcz1sb2codF9xc2l6ZV9tMiksIGRhdGE9ZHVnaGcsIG1ldGhvZD0iUkVNTCIsIHJhbmRvbT0gbGlzdCh+MSB8IGlkLCB+MSB8IHBsb3RfaWQsIH4xIHwgdW5pdCksIFc9MSkNCiNmaXhlZCBlZmZlY3RzIA0KaGcubWEuZml4IDwtIHJtYS5tdih5aT1zY2FsZSh5aSksIFY9dmksIG1vZHM9bG9nKHRfcXNpemVfbTIpLCBkYXRhPWR1Z2hnLCBtZXRob2Q9IlJFTUwiLCByYW5kb209IGxpc3QofjEgfCBpZCwgfjEgfCBwbG90X2lkLCB+MSB8IHVuaXQpLCBXID0gMS92aSkNCg0KaGcubWEucmFuLmRfYWx0IDwtIHJtYS5tdih5aT1zY2FsZSh5aSksIFY9dmkyLCBtb2RzPWxvZyh0X3FzaXplX20yKSwgZGF0YT1kdWdoZywgbWV0aG9kPSJSRU1MIiwgcmFuZG9tPSBsaXN0KH4xIHwgaWQsIH4xIHwgcGxvdF9pZCwgfjEgfCB1bml0KSkNCg0KaGcubWEuZml4LmRhbHQgPC0gcm1hLm12KHlpPXNjYWxlKHlpKSwgVj12aTIsIG1vZHM9bG9nKHRfcXNpemVfbTIpLCBkYXRhPWR1Z2hnLCBtZXRob2Q9IlJFTUwiLCByYW5kb209IGxpc3QofjEgfCBpZCwgfjEgfCBwbG90X2lkLCB+MSB8IHVuaXQpLCBXID0gMS92aTIpDQpgYGANCg0KYGBge3IsIGZpZy5oZWlnaHQ9IDMsIGZpZy53aWR0aD0gNSwgZXZhbCA9IEZBTFNFfQ0KaGRlZmYgPC0gZGF0YS5mcmFtZShlc3RpbWF0ZT1jKGhnLm1hLnJhbiRiWzJdLGhnLm1hLnVuJGJbMl0saGcubWEuZml4JGJbMl0saGcubWEucmFuLmRfYWx0JGJbMl0saGcubWEuZml4LmRhbHQkYlsyXSksY2kudXA9YyhoZy5tYS5yYW4kY2kudWJbMl0saGcubWEudW4kY2kudWJbMl0saGcubWEuZml4JGNpLnViWzJdLCBoZy5tYS5yYW4uZF9hbHQkY2kudWJbMl0saGcubWEuZml4LmRhbHQkY2kudWJbMl0pLCBjaS5sbz1jKGhnLm1hLnJhbiRjaS5sYlsyXSxoZy5tYS51biRjaS5sYlsyXSxoZy5tYS5maXgkY2kubGJbMl0saGcubWEucmFuLmRfYWx0JGNpLmxiWzJdLGhnLm1hLmZpeC5kYWx0JGNpLmxiWzJdKSwgd2VpZ2h0aW5nPWMoIlIiLCAiVSIsICJGIiwgIlIiLCAiRiIpLCB2dHlwZT1jKCJkIiwiZCIsImQiLCJkX2FsdCIsImRfYWx0IikpDQoNCmhkZWZmIDwtIGhkZWZmICU+JQ0KYXJyYW5nZSh3ZWlnaHRpbmcpICU+JSAgICAjIEZpcnN0IHNvcnQgYnkgdmFsLiBUaGlzIHNvcnQgdGhlIGRhdGFmcmFtZSBidXQgTk9UIHRoZSBmYWN0b3IgbGV2ZWxzDQogIG11dGF0ZSh3ZWlnaHRpbmc9ZmFjdG9yKHdlaWdodGluZywgbGV2ZWxzPQ0KICAgICAgICAgICAgICAgICAgICAgICAgICAgIGMoIlIiLCAiRiIsICJVIikpKSAgICAjIFRoaXMgdHJpY2sgdXBkYXRlIHRoZSBmYWN0b3IgbGV2ZWxzDQoNCmhkZWZmLnAuYSA8LWdncGxvdChoZGVmZikgKyBnZW9tX3BvaW50KGFlcyh5ID0gZXN0aW1hdGUsIHggPSB3ZWlnaHRpbmcsIGNvbG91cj12dHlwZSksIHBvc2l0aW9uPXBvc2l0aW9uX2RvZGdlKHdpZHRoID0gMC41KSkgKyBnZW9tX2Vycm9yYmFyKGFlcyh4PXdlaWdodGluZyx5bWluPWNpLmxvLCB5bWF4PWNpLnVwLCB3aWR0aCA9IDAuMSwgY29sb3VyPXZ0eXBlKSwsIHBvc2l0aW9uPXBvc2l0aW9uX2RvZGdlKHdpZHRoID0gMC41KSkrDQogICBnZW9tX2FibGluZShpbnRlcmNlcHQgPSAwLCBzbG9wZT0wLCBjb2xvdXIgPSAiZGFya2dyYXkiLCBsaW5ldHlwZT0iZGFzaGVkIiwpICsNCiAgeGxhYihOVUxMKSArDQogIHlsYWIoIkVmZmVjdCBvZiBwbG90IHNpemUgKHJlZ3Jlc3Npb24gY29lZmZpY2llbnQpIikrDQogIHRoZW1lX2J3KCkgKyANCiAgdGhlbWUocGxvdC50aXRsZSA9IGVsZW1lbnRfdGV4dChzaXplPTEwKSxheGlzLnRpdGxlID0gZWxlbWVudF90ZXh0KHNpemU9MTApLGF4aXMudGV4dCA9IGVsZW1lbnRfdGV4dChzaXplPTkpICkNCmhkZWZmLnAuYSA8LWhkZWZmLnAuYSArdGhlbWUoYXhpcy50ZXh0LnggPSBlbGVtZW50X3RleHQoYW5nbGU9MCxmYWNlID0gYyhyZXAoJ3BsYWluJywxNiksICdib2xkJywgJ2JvbGQnKSkpK3lsaW0oLTEsMS4yKSArc2NhbGVfY29sb3JfbWFudWFsKHZhbHVlcz1jKCJibGFjayIsICJkYXJrZ3JheSIpLCBndWlkZT1GKQ0KDQpscmVmZiA8LSBkYXRhLmZyYW1lKGVzdGltYXRlPWMobHIubWEucmFuJGJbMl0sbHIubWEudW4kYlsyXSxsci5tYS5maXgkYlsyXSksY2kudXA9Yyhsci5tYS5yYW4kY2kudWJbMl0sbHIubWEudW4kY2kudWJbMl0sbHIubWEuZml4JGNpLnViWzJdKSwgY2kubG89Yyhsci5tYS5yYW4kY2kubGJbMl0sbHIubWEudW4kY2kubGJbMl0sbHIubWEuZml4JGNpLmxiWzJdKSwgd2VpZ2h0aW5nPWMoIlIiLCAiVSIsICJGIikpDQpscmVmZiA8LSBscmVmZiAlPiUNCmFycmFuZ2Uod2VpZ2h0aW5nKSAlPiUgICAgIyBGaXJzdCBzb3J0IGJ5IHZhbC4gVGhpcyBzb3J0IHRoZSBkYXRhZnJhbWUgYnV0IE5PVCB0aGUgZmFjdG9yIGxldmVscw0KICBtdXRhdGUod2VpZ2h0aW5nPWZhY3Rvcih3ZWlnaHRpbmcsIGxldmVscz0NCiAgICAgICAgICAgICAgICAgICAgICAgICAgICBjKCJSIiwgIkYiLCAiVSIpKSkgICAgIyBUaGlzIHRyaWNrIHVwZGF0ZSB0aGUgZmFjdG9yIGxldmVscw0KDQoNCmxyZWZmLnAuYSA8LWdncGxvdChscmVmZikgKyBnZW9tX3BvaW50KGFlcyh5ID0gZXN0aW1hdGUsIHggPSB3ZWlnaHRpbmcpKSArIGdlb21fZXJyb3JiYXIoYWVzKHg9d2VpZ2h0aW5nLHltaW49Y2kubG8sIHltYXg9Y2kudXAsIHdpZHRoID0gMC4xKSkrDQogIGdlb21fYWJsaW5lKGludGVyY2VwdCA9IDAsIHNsb3BlPTAsIGNvbG91ciA9ICJkYXJrZ3JheSIsIGxpbmV0eXBlPSJkYXNoZWQiKSArDQogIHhsYWIoTlVMTCkgKw0KICB5bGFiKCJFZmZlY3Qgb2YgcGxvdCBzaXplIChyZWdyZXNzaW9uIGNvZWZmaWNpZW50KSIpKw0KICB0aGVtZV9idygpICsgDQogIHRoZW1lKHBsb3QudGl0bGUgPSBlbGVtZW50X3RleHQoc2l6ZT0xMCksYXhpcy50aXRsZSA9IGVsZW1lbnRfdGV4dChzaXplPTEwKSxheGlzLnRleHQgPSBlbGVtZW50X3RleHQoc2l6ZT05KSApDQpscmVmZi5wLmEgPC1scmVmZi5wLmEgK3RoZW1lKGF4aXMudGV4dC54ID0gZWxlbWVudF90ZXh0KGFuZ2xlPTAsZmFjZSA9IGMocmVwKCdwbGFpbicsMTYpLCAnYm9sZCcsICdib2xkJykpKSt5bGltKC0xLjIsMS41KQ0KDQoNCmN2cmVmZiA8LSBkYXRhLmZyYW1lKGVzdGltYXRlPWMoY3ZyLm1hLnJhbiRiWzJdLGN2ci5tYS51biRiWzJdLGN2ci5tYS5maXgkYlsyXSksY2kudXA9YyhjdnIubWEucmFuJGNpLnViWzJdLGN2ci5tYS51biRjaS51YlsyXSxjdnIubWEuZml4JGNpLnViWzJdKSwgY2kubG89YyhjdnIubWEucmFuJGNpLmxiWzJdLGN2ci5tYS51biRjaS5sYlsyXSxjdnIubWEuZml4JGNpLmxiWzJdKSwgd2VpZ2h0aW5nPWMoIlIiLCAiVSIsICJGIikpDQpjdnJlZmYgPC0gY3ZyZWZmICU+JQ0KYXJyYW5nZSh3ZWlnaHRpbmcpICU+JSAgICAjIEZpcnN0IHNvcnQgYnkgdmFsLiBUaGlzIHNvcnQgdGhlIGRhdGFmcmFtZSBidXQgTk9UIHRoZSBmYWN0b3IgbGV2ZWxzDQogIG11dGF0ZSh3ZWlnaHRpbmc9ZmFjdG9yKHdlaWdodGluZywgbGV2ZWxzPQ0KICAgICAgICAgICAgICAgICAgICAgICAgICAgIGMoIlIiLCAiRiIsICJVIikpKSAgICAjIFRoaXMgdHJpY2sgdXBkYXRlIHRoZSBmYWN0b3IgbGV2ZWxzDQoNCg0KY3ZyZWZmLnAuYSA8LWdncGxvdChjdnJlZmYpICsgZ2VvbV9wb2ludChhZXMoeSA9IGVzdGltYXRlLCB4ID0gd2VpZ2h0aW5nKSkgKyBnZW9tX2Vycm9yYmFyKGFlcyh4PXdlaWdodGluZyx5bWluPWNpLmxvLCB5bWF4PWNpLnVwLCB3aWR0aCA9IDAuMSkpKw0KICBnZW9tX2FibGluZShpbnRlcmNlcHQgPSAwLCBzbG9wZT0wLCBjb2xvdXIgPSAiZGFya2dyYXkiLCBsaW5ldHlwZT0iZGFzaGVkIikgKw0KICB4bGFiKE5VTEwpICsNCiAgeWxhYigiRWZmZWN0IG9mIHBsb3Qgc2l6ZSAocmVncmVzc2lvbiBjb2VmZmljaWVudCkiKSsNCiAgdGhlbWVfYncoKSArIA0KICB0aGVtZShwbG90LnRpdGxlID0gZWxlbWVudF90ZXh0KHNpemU9MTApLGF4aXMudGl0bGUgPSBlbGVtZW50X3RleHQoc2l6ZT0xMCksYXhpcy50ZXh0ID0gZWxlbWVudF90ZXh0KHNpemU9OSkgKQ0KY3ZyZWZmLnAuYSA8LWN2cmVmZi5wLmEgK3RoZW1lKGF4aXMudGV4dC54ID0gZWxlbWVudF90ZXh0KGFuZ2xlPTAsZmFjZSA9IGMocmVwKCdwbGFpbicsMTYpLCAnYm9sZCcsICdib2xkJykpKSt5bGltKC0xLjIsMS41KQ0KDQpncmlkRXh0cmE6OmdyaWQuYXJyYW5nZShoZGVmZi5wLmEsIGxyZWZmLnAuYSwgY3ZyZWZmLnAuYSwgbmNvbD0zLCB3aWR0aHM9YygzLDIuMywgMi4zKSkNCmBgYA0KDQpSZS1ydW4gbWV0YS1hbmFseXNlcywgYnV0IG9uIHVuc2NhbGVkIHJlc3BvbnNlIHZhcmlhYmxlcyBzbyBjYW4gbWFrZSBwcmVkaWN0aW9ucyBvZiB1bnNjYWxlZCBlZmZlY3Qgc2l6ZXMuDQoNCmBgYHtyICwgZmlnLmhlaWdodD0gMywgZmlnLndpZHRoPSA1LCBldmFsID0gRkFMU0V9DQpoZy5tYS5yYW4gPC0gcm1hLm12KHlpPXlpLCBWPXZpLCBtb2RzPWxvZyh0X3FzaXplX20yKSwgZGF0YT1kdWdoZywgbWV0aG9kPSJSRU1MIiwgcmFuZG9tPSBsaXN0KH4xIHwgaWQsIH4xIHwgcGxvdF9pZCwgfjEgfCB1bml0KSkNCiNyYW5kb20gdW53ZWlnaHRlZCAoc2FtZSBtZXRhLWVzdCBpZiBmaXhlZCB1bndlaWdodGVkLCBidXQgZGlmZiBzZSkNCmhnLm1hLnVuIDwtIHJtYS5tdih5aT15aSwgVj12aSwgbW9kcz1sb2codF9xc2l6ZV9tMiksIGRhdGE9ZHVnaGcsIG1ldGhvZD0iUkVNTCIsIHJhbmRvbT0gbGlzdCh+MSB8IGlkLCB+MSB8IHBsb3RfaWQsIH4xIHwgdW5pdCksIFc9MSkNCiNyYW5kb20gZWZmZWN0IGJ1dCBjb250cm9sIHRoZSB3ZWlnaHRzLCBkbyAxL3YuIHNhbWUgZXN0IGFzIGEgZml4ZWQgZWZmZWN0DQpoZy5tYS5maXggPC0gcm1hLm12KHlpPXlpLCBWPXZpLCBtb2RzPWxvZyh0X3FzaXplX20yKSwgZGF0YT1kdWdoZywgbWV0aG9kPSJSRU1MIiwgcmFuZG9tPSBsaXN0KH4xIHwgaWQsIH4xIHwgcGxvdF9pZCwgfjEgfCB1bml0KSwgVyA9IDEvdmkpDQoNCmhnLm1hLnJhbi5kX2FsdCA8LSBybWEubXYoeWk9eWksIFY9dmkyLCBtb2RzPWxvZyh0X3FzaXplX20yKSwgZGF0YT1kdWdoZywgbWV0aG9kPSJSRU1MIiwgcmFuZG9tPSBsaXN0KH4xIHwgaWQsIH4xIHwgcGxvdF9pZCwgfjEgfCB1bml0KSkNCg0KaGcubWEuZml4LmRhbHQgPC0gcm1hLm12KHlpPXlpLCBWPXZpMiwgbW9kcz1sb2codF9xc2l6ZV9tMiksIGRhdGE9ZHVnaGcsIG1ldGhvZD0iUkVNTCIsIHJhbmRvbT0gbGlzdCh+MSB8IGlkLCB+MSB8IHBsb3RfaWQsIH4xIHwgdW5pdCksIFcgPSAxL3ZpMikNCg0KDQpsci5tYS5yYW4gPC0gcm1hLm12KHlpPXlpLCBWPXZpLCBtb2RzPWxvZyh0X3FzaXplX20yKSwgZGF0YT1kdWdsciwgbWV0aG9kPSJSRU1MIiwgcmFuZG9tPSBsaXN0KH4xIHwgaWQsIH4xIHwgcGxvdF9pZCwgfjEgfCB1bml0KSkNCiNyYW5kb20gdW53ZWlnaHRlZCAoc2FtZSBtZXRhLWVzdCBpZiBmaXhlZCB1bndlaWdodGVkLCBidXQgZGlmZiBzZSkNCmxyLm1hLnVuIDwtIHJtYS5tdih5aT15aSwgVj12aSwgbW9kcz1sb2codF9xc2l6ZV9tMiksIGRhdGE9ZHVnbHIsIG1ldGhvZD0iUkVNTCIsIHJhbmRvbT0gbGlzdCh+MSB8IGlkLCB+MSB8IHBsb3RfaWQsIH4xIHwgdW5pdCksIFc9MSkNCiNyYW5kb20gZWZmZWN0IGJ1dCBjb250cm9sIHRoZSB3ZWlnaHRzLCBkbyAxL3YuIHNhbWUgZXN0IGFzIGEgZml4ZWQgZWZmZWN0DQpsci5tYS5maXggPC0gcm1hLm12KHlpPXlpLCBWPXZpLCBtb2RzPWxvZyh0X3FzaXplX20yKSwgZGF0YT1kdWdsciwgbWV0aG9kPSJSRU1MIiwgcmFuZG9tPSBsaXN0KH4xIHwgaWQsIH4xIHwgcGxvdF9pZCwgfjEgfCB1bml0KSwgVyA9IDEvdmkpDQoNCg0KY3ZyLm1hLnJhbiA8LSBybWEubXYoeWk9eWksIFY9dmksIG1vZHM9bG9nKHRfcXNpemVfbTIpLCBkYXRhPWR1Z2N2ciwgbWV0aG9kPSJSRU1MIiwgcmFuZG9tPSBsaXN0KH4xIHwgaWQsIH4xIHwgcGxvdF9pZCwgfjEgfCB1bml0KSkNCiNyYW5kb20gdW53ZWlnaHRlZCAoc2FtZSBtZXRhLWVzdCBpZiBmaXhlZCB1bndlaWdodGVkLCBidXQgZGlmZiBzZSkNCmN2ci5tYS51biA8LSBybWEubXYoeWk9eWksIFY9dmksIG1vZHM9bG9nKHRfcXNpemVfbTIpLCBkYXRhPWR1Z2N2ciwgbWV0aG9kPSJSRU1MIiwgcmFuZG9tPSBsaXN0KH4xIHwgaWQsIH4xIHwgcGxvdF9pZCwgfjEgfCB1bml0KSwgVz0xKQ0KI3JhbmRvbSBlZmZlY3QgYnV0IGNvbnRyb2wgdGhlIHdlaWdodHMsIGRvIDEvdi4gc2FtZSBlc3QgYXMgYSBmaXhlZCBlZmZlY3QNCmN2ci5tYS5maXggPC0gcm1hLm12KHlpPXlpLCBWPXZpLCBtb2RzPWxvZyh0X3FzaXplX20yKSwgZGF0YT1kdWdjdnIsIG1ldGhvZD0iUkVNTCIsIHJhbmRvbT0gbGlzdCh+MSB8IGlkLCB+MSB8IHBsb3RfaWQsIH4xIHwgdW5pdCksIFcgPSAxL3ZpKQ0KDQpgYGANCg0KTm93IHByZWRpY3QgdGhlIGVmZmVjdCBzaXplcyBhY3Jvc3MgYWxsIGludGVycG9sYXRlZCB2YWx1ZXMgb2YgQSwgcGxvdCB0aGUgbWV0YS1yZWdyZXNzaW9uIHNsb3BlczoNCg0KYGBge3IgY2FjaGU9VFJVRSwgZmlnLmhlaWdodD04LCBmaWcud2lkdGg9OH0NCg0KbmV3bW9kcz1kYXRhLmZyYW1lKGludGVyY2VwdD1oZy5tYS5yYW4kYlsxXSwgdF9xc2l6ZV9tMj1zZXEobWluKGR1Z2hnJHRfcXNpemVfbTIpLCBtYXgoZHVnaGckdF9xc2l6ZV9tMiksIDAuMSkgKQ0KbmV3bW9kcz1hcy5tYXRyaXgobmV3bW9kcykNCiNoZWFkKG5ld21vZHMpDQpoZy5tYS5yYW4ucHJlZHM9ZGF0YS5mcmFtZShwcmVkaWN0KGhnLm1hLnJhbiwgIGFkZHg9VFJVRSkpDQpoZy5tYS5maXgucHJlZHM9ZGF0YS5mcmFtZShwcmVkaWN0KGhnLm1hLmZpeCwgIGFkZHg9VFJVRSkpDQpoZy5tYS51bi5wcmVkcz1kYXRhLmZyYW1lKHByZWRpY3QoaGcubWEudW4sICBhZGR4PVRSVUUpKQ0KI3dlaWdodHMoaGcubWEucmFuKQ0KaGcubWEucmFuLnA9Z2dwbG90KCkrZ2VvbV9wb2ludChkYXRhPWR1Z2hnLGFlcyh4PWxvZyh0X3FzaXplX20yKSwgeT15aSxjb2xvdXI9aWQpLCANCiAgICAgICAgICAgICAgICAgICAgICAgICAgICAgICAgICAgICAgICAgICAgICAgI3NpemU9d2VpZ2h0cyhoZy5tYS5yYW4pKSwgDQogICAgICAgICAgICAgICAgICAgICAgICAgICAgICAgIGFscGhhPTAuMykgK2dlb21fbGluZShhZXMoeD1oZy5tYS5yYW4ucHJlZHMkWC5tb2RzLCB5PWhnLm1hLnJhbi5wcmVkcyRwcmVkKSkrZ2VvbV9saW5lKGFlcyh4PWhnLm1hLnJhbi5wcmVkcyRYLm1vZHMsIHk9aGcubWEucmFuLnByZWRzJHByZWQpKStnZW9tX3JpYmJvbihhZXMoeD1oZy5tYS5yYW4ucHJlZHMkWC5tb2RzLHltaW49aGcubWEucmFuLnByZWRzJGNpLmxiLCB5bWF4PWhnLm1hLnJhbi5wcmVkcyRjaS51YiApLGFscGhhPTAuMikrc2NhbGVfc2l6ZV9jb250aW51b3VzKGd1aWRlPUZBTFNFKStzY2FsZV9jb2xvcl9kaXNjcmV0ZShndWlkZT1GKSt0aGVtZV9idygpK3lsYWIoZXhwcmVzc2lvbihpdGFsaWMoZykpKSt4bGFiKE5VTEwpK2dlb21faGxpbmUoeWludGVyY2VwdCA9IDAsIGxpbmV0eXBlID0gImRhc2hlZCIpICtnZ3RpdGxlKGV4cHJlc3Npb24ocGFzdGUoIlJhbmRvbS1lZmZlY3RzIG1ldGEtYW5hbHlzaXMsIHd0ID0gMS8oIiwgaXRhbGljKCJWIiksIisiLHRhdV4yLCIpIikpKSt0aGVtZShwbG90LnRpdGxlID0gZWxlbWVudF90ZXh0KHNpemUgPSA4KSkreWxpbSgtMTIsNy4yKQ0KDQoNCmhnLm1hLmZpeC5wPWdncGxvdCgpK2dlb21fcG9pbnQoZGF0YT1kdWdoZyxhZXMoeD1sb2codF9xc2l6ZV9tMiksIHk9eWksY29sb3VyPWlkKSwgI3NpemU9d2VpZ2h0cyhoZy5tYS5maXgpKSwNCiAgICAgICAgICAgICAgICAgICAgICAgICAgICAgICAgICAgICAgICAgICAgICAgYWxwaGE9MC4zKSArZ2VvbV9saW5lKGFlcyh4PWhnLm1hLmZpeC5wcmVkcyRYLm1vZHMsIHk9aGcubWEuZml4LnByZWRzJHByZWQpKStnZW9tX2xpbmUoYWVzKHg9aGcubWEuZml4LnByZWRzJFgubW9kcywgeT1oZy5tYS5maXgucHJlZHMkcHJlZCkpK2dlb21fcmliYm9uKGFlcyh4PWhnLm1hLmZpeC5wcmVkcyRYLm1vZHMseW1pbj1oZy5tYS5maXgucHJlZHMkY2kubGIsIHltYXg9aGcubWEuZml4LnByZWRzJGNpLnViICksYWxwaGE9MC4yKStzY2FsZV9zaXplX2NvbnRpbnVvdXMoZ3VpZGU9RkFMU0UpK3NjYWxlX2NvbG9yX2Rpc2NyZXRlKGd1aWRlPUYpK3RoZW1lX2J3KCkreWxhYihleHByZXNzaW9uKGl0YWxpYyhnKSkpK3hsYWIoTlVMTCkrZ2VvbV9obGluZSh5aW50ZXJjZXB0ID0gMCwgbGluZXR5cGUgPSAiZGFzaGVkIikrZ2d0aXRsZShleHByZXNzaW9uKHBhc3RlKCJGaXhlZC1lZmZlY3RzIG1ldGEtYW5hbHlzaXMsIHd0ID0gMS8oIiwgaXRhbGljKCJWIiksIikiKSkpK3RoZW1lKHBsb3QudGl0bGUgPSBlbGVtZW50X3RleHQoc2l6ZSA9IDgpKSt5bGltKC0xMiw3LjIpDQoNCmhnLm1hLnVuLnA9Z2dwbG90KCkrZ2VvbV9wb2ludChkYXRhPWR1Z2hnLGFlcyh4PWxvZyh0X3FzaXplX20yKSwgeT15aSxjb2xvdXI9aWQsIHNpemU9MSksIGFscGhhPTAuMykgK2dlb21fbGluZShhZXMoeD1oZy5tYS5maXgucHJlZHMkWC5tb2RzLCB5PWhnLm1hLnVuLnByZWRzJHByZWQpKStnZW9tX2xpbmUoYWVzKHg9aGcubWEudW4ucHJlZHMkWC5tb2RzLCB5PWhnLm1hLnVuLnByZWRzJHByZWQpKStnZW9tX3JpYmJvbihhZXMoeD1oZy5tYS51bi5wcmVkcyRYLm1vZHMseW1pbj1oZy5tYS51bi5wcmVkcyRjaS5sYiwgeW1heD1oZy5tYS51bi5wcmVkcyRjaS51YiApLGFscGhhPTAuMikrc2NhbGVfY29sb3JfZGlzY3JldGUoZ3VpZGU9RikrdGhlbWVfYncoKSt5bGFiKGV4cHJlc3Npb24oaXRhbGljKGcpKSkreGxhYigiTG9nKFBsb3Qgc2l6ZSAoaGEpKSIpK2dlb21faGxpbmUoeWludGVyY2VwdCA9IDAsIGxpbmV0eXBlID0gImRhc2hlZCIpK3NjYWxlX3NpemVfY29udGludW91cyhndWlkZT1GQUxTRSkrZ2d0aXRsZSgiVW53ZWlnaHRlZCBtZXRhLWFuYWx5c2lzLCB3dCA9IDEiKSt0aGVtZShwbG90LnRpdGxlID0gZWxlbWVudF90ZXh0KHNpemUgPSA4KSkreWxpbSgtMTIsNy4yKQ0KDQoNCg0KDQpuZXdtb2RzPWRhdGEuZnJhbWUoaW50ZXJjZXB0PWxyLm1hLnJhbiRiWzFdLCB0X3FzaXplX20yPXNlcShtaW4oZHVnbHIkdF9xc2l6ZV9tMiksIG1heChkdWdsciR0X3FzaXplX20yKSwgMC4xKSApDQpuZXdtb2RzPWFzLm1hdHJpeChuZXdtb2RzKQ0KI2hlYWQobmV3bW9kcykNCmxyLm1hLnJhbi5wcmVkcz1kYXRhLmZyYW1lKHByZWRpY3QobHIubWEucmFuLCAgYWRkeD1UUlVFKSkNCmxyLm1hLmZpeC5wcmVkcz1kYXRhLmZyYW1lKHByZWRpY3QobHIubWEuZml4LCAgYWRkeD1UUlVFKSkNCmxyLm1hLnVuLnByZWRzPWRhdGEuZnJhbWUocHJlZGljdChsci5tYS51biwgIGFkZHg9VFJVRSkpDQoNCmxyLm1hLnJhbi5wPWdncGxvdCgpK2dlb21fcG9pbnQoZGF0YT1kdWdscixhZXMoeD1sb2codF9xc2l6ZV9tMiksIHk9eWksY29sb3VyPWlkKSwjIHNpemU9d2VpZ2h0cyhsci5tYS5yYW4pKSwNCiAgICAgICAgICAgICAgICAgICAgICAgICAgICAgICAgYWxwaGE9MC4zKSArZ2VvbV9saW5lKGFlcyh4PWxyLm1hLnJhbi5wcmVkcyRYLm1vZHMsIHk9bHIubWEucmFuLnByZWRzJHByZWQpKStnZW9tX2xpbmUoYWVzKHg9bHIubWEucmFuLnByZWRzJFgubW9kcywgeT1sci5tYS5yYW4ucHJlZHMkcHJlZCkpK2dlb21fcmliYm9uKGFlcyh4PWxyLm1hLnJhbi5wcmVkcyRYLm1vZHMseW1pbj1sci5tYS5yYW4ucHJlZHMkY2kubGIsIHltYXg9bHIubWEucmFuLnByZWRzJGNpLnViICksYWxwaGE9MC4yKStzY2FsZV9zaXplX2NvbnRpbnVvdXMoZ3VpZGU9RkFMU0UpK3NjYWxlX2NvbG9yX2Rpc2NyZXRlKGd1aWRlPUYpK3RoZW1lX2J3KCkreWxhYihleHByZXNzaW9uKGl0YWxpYyhMUikpKSt4bGFiKE5VTEwpK2dlb21faGxpbmUoeWludGVyY2VwdCA9IDAsIGxpbmV0eXBlID0gImRhc2hlZCIpICtnZ3RpdGxlKCIiKSt0aGVtZShwbG90LnRpdGxlID0gZWxlbWVudF90ZXh0KHNpemUgPSA4KSkreWxpbSgtMS42LDEuMikNCg0KDQpsci5tYS5maXgucD1nZ3Bsb3QoKStnZW9tX3BvaW50KGRhdGE9ZHVnbHIsYWVzKHg9bG9nKHRfcXNpemVfbTIpLCB5PXlpLGNvbG91cj1pZCksIyBzaXplPXdlaWdodHMobHIubWEuZml4KSksDQogICAgICAgICAgICAgICAgICAgICAgICAgICAgICAgIGFscGhhPTAuMykgK2dlb21fbGluZShhZXMoeD1sci5tYS5maXgucHJlZHMkWC5tb2RzLCB5PWxyLm1hLmZpeC5wcmVkcyRwcmVkKSkrZ2VvbV9saW5lKGFlcyh4PWxyLm1hLmZpeC5wcmVkcyRYLm1vZHMsIHk9bHIubWEuZml4LnByZWRzJHByZWQpKStnZW9tX3JpYmJvbihhZXMoeD1sci5tYS5maXgucHJlZHMkWC5tb2RzLHltaW49bHIubWEuZml4LnByZWRzJGNpLmxiLCB5bWF4PWxyLm1hLmZpeC5wcmVkcyRjaS51YiApLGFscGhhPTAuMikrc2NhbGVfc2l6ZV9jb250aW51b3VzKGd1aWRlPUZBTFNFKStzY2FsZV9jb2xvcl9kaXNjcmV0ZShndWlkZT1GKSt0aGVtZV9idygpK3lsYWIoZXhwcmVzc2lvbihpdGFsaWMoTFIpKSkreGxhYihOVUxMKStnZW9tX2hsaW5lKHlpbnRlcmNlcHQgPSAwLCBsaW5ldHlwZSA9ICJkYXNoZWQiKStnZ3RpdGxlKCIiKSt0aGVtZShwbG90LnRpdGxlID0gZWxlbWVudF90ZXh0KHNpemUgPSA4KSkrY29vcmRfY2FydGVzaWFuKHlsaW0gPSBjKC0xLjYsIDEuMikpIA0KDQoNCg0KbHIubWEudW4ucD1nZ3Bsb3QoKStnZW9tX3BvaW50KGRhdGE9ZHVnbHIsYWVzKHg9bG9nKHRfcXNpemVfbTIpLCB5PXlpLGNvbG91cj1pZCwgc2l6ZT0xKSwgYWxwaGE9MC4zKSArZ2VvbV9saW5lKGFlcyh4PWxyLm1hLmZpeC5wcmVkcyRYLm1vZHMsIHk9bHIubWEudW4ucHJlZHMkcHJlZCkpK2dlb21fbGluZShhZXMoeD1sci5tYS51bi5wcmVkcyRYLm1vZHMsIHk9bHIubWEudW4ucHJlZHMkcHJlZCkpK2dlb21fcmliYm9uKGFlcyh4PWxyLm1hLnVuLnByZWRzJFgubW9kcyx5bWluPWxyLm1hLnVuLnByZWRzJGNpLmxiLCB5bWF4PWxyLm1hLnVuLnByZWRzJGNpLnViICksYWxwaGE9MC4yKStzY2FsZV9jb2xvcl9kaXNjcmV0ZShndWlkZT1GKSt0aGVtZV9idygpK3lsYWIoZXhwcmVzc2lvbihpdGFsaWMoTFIpKSkreGxhYigiTG9nKFBsb3Qgc2l6ZSAoaGEpKSIpK2dlb21faGxpbmUoeWludGVyY2VwdCA9IDAsIGxpbmV0eXBlID0gImRhc2hlZCIpK3NjYWxlX3NpemVfY29udGludW91cyhndWlkZT1GQUxTRSkrZ2d0aXRsZSgiIikrdGhlbWUocGxvdC50aXRsZSA9IGVsZW1lbnRfdGV4dChzaXplID0gOCkpK3lsaW0oLTEuNiwgMS4yKQ0KDQpuZXdtb2RzPWRhdGEuZnJhbWUoaW50ZXJjZXB0PWN2ci5tYS5yYW4kYlsxXSwgdF9xc2l6ZV9tMj1zZXEobWluKGR1Z2N2ciR0X3FzaXplX20yKSwgbWF4KGR1Z2N2ciR0X3FzaXplX20yKSwgMC4xKSApDQpuZXdtb2RzPWFzLm1hdHJpeChuZXdtb2RzKQ0KI2hlYWQobmV3bW9kcykNCmN2ci5tYS5yYW4ucHJlZHM9ZGF0YS5mcmFtZShwcmVkaWN0KGN2ci5tYS5yYW4sICBhZGR4PVRSVUUpKQ0KY3ZyLm1hLmZpeC5wcmVkcz1kYXRhLmZyYW1lKHByZWRpY3QoY3ZyLm1hLmZpeCwgIGFkZHg9VFJVRSkpDQpjdnIubWEudW4ucHJlZHM9ZGF0YS5mcmFtZShwcmVkaWN0KGN2ci5tYS51biwgIGFkZHg9VFJVRSkpDQoNCmN2ci5tYS5yYW4ucD1nZ3Bsb3QoKStnZW9tX3BvaW50KGRhdGE9ZHVnY3ZyLGFlcyh4PWxvZyh0X3FzaXplX20yKSwgeT15aSxjb2xvdXI9aWQpLCMgc2l6ZT13ZWlnaHRzKGN2ci5tYS5yYW4pKSwNCiAgICAgICAgICAgICAgICAgICAgICAgICAgICAgICAgYWxwaGE9MC4zKSArZ2VvbV9saW5lKGFlcyh4PWN2ci5tYS5yYW4ucHJlZHMkWC5tb2RzLCB5PWN2ci5tYS5yYW4ucHJlZHMkcHJlZCkpK2dlb21fbGluZShhZXMoeD1jdnIubWEucmFuLnByZWRzJFgubW9kcywgeT1jdnIubWEucmFuLnByZWRzJHByZWQpKStnZW9tX3JpYmJvbihhZXMoeD1jdnIubWEucmFuLnByZWRzJFgubW9kcyx5bWluPWN2ci5tYS5yYW4ucHJlZHMkY2kubGIsIHltYXg9Y3ZyLm1hLnJhbi5wcmVkcyRjaS51YiApLGFscGhhPTAuMikrc2NhbGVfc2l6ZV9jb250aW51b3VzKGd1aWRlPUZBTFNFKStzY2FsZV9jb2xvcl9kaXNjcmV0ZShndWlkZT1GKSt0aGVtZV9idygpK3lsYWIoZXhwcmVzc2lvbihpdGFsaWMoY3ZyKSkpK3hsYWIoTlVMTCkrZ2VvbV9obGluZSh5aW50ZXJjZXB0ID0gMCwgbGluZXR5cGUgPSAiZGFzaGVkIikgK2dndGl0bGUoIiIpK3RoZW1lKHBsb3QudGl0bGUgPSBlbGVtZW50X3RleHQoc2l6ZSA9IDgpKSt5bGltKC0xLjYsMS4yKQ0KDQoNCmN2ci5tYS5maXgucD1nZ3Bsb3QoKStnZW9tX3BvaW50KGRhdGE9ZHVnY3ZyLGFlcyh4PWxvZyh0X3FzaXplX20yKSwgeT15aSxjb2xvdXI9aWQpLCMgc2l6ZT13ZWlnaHRzKGN2ci5tYS5maXgpKSwNCiAgICAgICAgICAgICAgICAgICAgICAgICAgICAgICAgYWxwaGE9MC4zKSArZ2VvbV9saW5lKGFlcyh4PWN2ci5tYS5maXgucHJlZHMkWC5tb2RzLCB5PWN2ci5tYS5maXgucHJlZHMkcHJlZCkpK2dlb21fbGluZShhZXMoeD1jdnIubWEuZml4LnByZWRzJFgubW9kcywgeT1jdnIubWEuZml4LnByZWRzJHByZWQpKStnZW9tX3JpYmJvbihhZXMoeD1jdnIubWEuZml4LnByZWRzJFgubW9kcyx5bWluPWN2ci5tYS5maXgucHJlZHMkY2kubGIsIHltYXg9Y3ZyLm1hLmZpeC5wcmVkcyRjaS51YiApLGFscGhhPTAuMikrc2NhbGVfc2l6ZV9jb250aW51b3VzKGd1aWRlPUZBTFNFKStzY2FsZV9jb2xvcl9kaXNjcmV0ZShndWlkZT1GKSt0aGVtZV9idygpK3lsYWIoZXhwcmVzc2lvbihpdGFsaWMoY3ZyKSkpK3hsYWIoTlVMTCkrZ2VvbV9obGluZSh5aW50ZXJjZXB0ID0gMCwgbGluZXR5cGUgPSAiZGFzaGVkIikrZ2d0aXRsZSgiIikrdGhlbWUocGxvdC50aXRsZSA9IGVsZW1lbnRfdGV4dChzaXplID0gOCkpK2Nvb3JkX2NhcnRlc2lhbih5bGltID0gYygtMS42LCAxLjIpKSANCg0KDQoNCmN2ci5tYS51bi5wPWdncGxvdCgpK2dlb21fcG9pbnQoZGF0YT1kdWdjdnIsYWVzKHg9bG9nKHRfcXNpemVfbTIpLCB5PXlpLGNvbG91cj1pZCwgc2l6ZT0xKSwgYWxwaGE9MC4zKSArZ2VvbV9saW5lKGFlcyh4PWN2ci5tYS5maXgucHJlZHMkWC5tb2RzLCB5PWN2ci5tYS51bi5wcmVkcyRwcmVkKSkrZ2VvbV9saW5lKGFlcyh4PWN2ci5tYS51bi5wcmVkcyRYLm1vZHMsIHk9Y3ZyLm1hLnVuLnByZWRzJHByZWQpKStnZW9tX3JpYmJvbihhZXMoeD1jdnIubWEudW4ucHJlZHMkWC5tb2RzLHltaW49Y3ZyLm1hLnVuLnByZWRzJGNpLmxiLCB5bWF4PWN2ci5tYS51bi5wcmVkcyRjaS51YiApLGFscGhhPTAuMikrc2NhbGVfY29sb3JfZGlzY3JldGUoZ3VpZGU9RikrdGhlbWVfYncoKSt5bGFiKGV4cHJlc3Npb24oaXRhbGljKGN2cikpKSt4bGFiKCJMb2coUGxvdCBzaXplIChoYSkpIikrZ2VvbV9obGluZSh5aW50ZXJjZXB0ID0gMCwgbGluZXR5cGUgPSAiZGFzaGVkIikrc2NhbGVfc2l6ZV9jb250aW51b3VzKGd1aWRlPUZBTFNFKStnZ3RpdGxlKCIiKSt0aGVtZShwbG90LnRpdGxlID0gZWxlbWVudF90ZXh0KHNpemUgPSA4KSkreWxpbSgtMS42LCAxLjIpDQoNCg0KDQoNCmdyaWRFeHRyYTo6Z3JpZC5hcnJhbmdlKGhnLm1hLnJhbi5wLCBsci5tYS5yYW4ucCxjdnIubWEucmFuLnAsDQogICAgICAgICAgICAgICAgICAgICAgICBoZy5tYS5maXgucCwgbHIubWEuZml4LnAsY3ZyLm1hLmZpeC5wLA0KICAgICAgICAgICAgICAgICAgICAgICAgaGcubWEudW4ucCwgbHIubWEudW4ucCxjdnIubWEuZml4LnAsDQogICAgICAgICAgICAgICAgICAgICAgICBuY29sPTMsIGhlaWdodHM9YygyLDIsMi4yKSkjK3lsaW0oLTMuMiwgMC43KQ0KYGBgDQoNCioqRmlndXJlIFMxMS4gTWV0YS1yZWdyZXNzaW9uIHNsb3BlcyBvZiBtb2RlbHMgdXNpbmcgaGVkZ2VzIGcsIGxuUlIsIGFuZCBsbkNWUiBhZ2FpbnN0IGxvZyhwbG90IHNpemUpLiBFYWNoIG1ldGEtcmVncmVzc2lvbiBpcyBjb25kdWN0ZWQgYXMgYSByYW5kb20tZWZmZWN0LCBmaXhlZC1lZmZlY3RzLCBhbmQgdW53ZWlnaHRlZCBtZXRhLWFuYWx5c2lzKioNCg0KVGhpcyBzaXplLWJpYXMgdGVzdGluZyBoYXMgb25seSBiZWVuIGNvbmR1Y3RlZCBmb3IgdGhlIHJlc3RvcmVkL3VucmVzdG9yZWQgY29tcGFyaXNvbi4gVGhlIGNodW5rIGJlbG93IHdpbGwgcmUtcnVuIHRoZSBzYW1lIHRlc3RzIG9uIHRoZSByZXN0b3JlZC9yZWZlcmVuY2UgY29tcGFyaXNvbiwgaG93ZXZlciB0aGVzZSBwbG90cyB3aWxsIG5vdCBiZSBwcmVzZW50ZWQgYXMgdGhleSBkbyBub3QgZGV2aWF0ZSBzaWduaWZpY2FudGx5IGZyb20gdGhlIHJlc3VsdHMganVzdCBwcmVzZW50ZWQNCg0KYGBge3IsIGZpZy5oZWlnaHQ9OSwgZmlnLndpZHRoPTgsIGV2YWwgPSBGQUxTRX0NCg0KZHVnIDwtIHJlYWQuY3N2KCJEYXRhL3ZhcmlhdGlvbl9kYXRhLmNzdiIpI3B1dCBkYXRhIGZpbGUgaGVyZQ0KZHVnPC1kdWcgJT4lIGZpbHRlcihyX3F1YWRfbiA+PSAxKQ0KZHVnbHIgPC0gbWV0YWZvcjo6ZXNjYWxjKG1lYXN1cmU9IlJPTSIsbTFpPWR1ZyR0X21lYW4sIG0yaT1kdWckcl9tZWFuLCBzZDFpPWR1ZyR0X3NkLCBzZDJpPXJfc2QsIG4xaT1kdWckdF9xdWFkX24sIG4yaT1kdWckcl9xdWFkX24sIGFwcGVuZD1ULCBkYXRhPWR1ZykgI25vbi1lcXVhbCB2YXJpYW5jZXMgDQoNCiNsbkNWUg0KZHVnY3ZyIDwtIG1ldGFmb3I6OmVzY2FsYyhtZWFzdXJlPSJDVlIiLG0xaT1kdWckdF9tZWFuLCBtMmk9ZHVnJHJfbWVhbiwgc2QxaT1kdWckdF9zZCwgc2QyaT1yX3NkLCBuMWk9ZHVnJHRfcXVhZF9uLCBuMmk9ZHVnJHJfcXVhZF9uLCBhcHBlbmQ9VCwgZGF0YT1kdWcpIA0KDQojSGVkZ2VzJyBnDQpkdWdoZyA8LSBtZXRhZm9yOjplc2NhbGMobWVhc3VyZT0iU01EIixtMWk9ZHVnJHRfbWVhbiwgbTJpPWR1ZyRyX21lYW4sIHNkMWk9ZHVnJHRfc2QsIHNkMmk9cl9zZCwgbjFpPWR1ZyR0X3F1YWRfbiwgbjJpPWR1ZyRyX3F1YWRfbiwgYXBwZW5kPVQsIGRhdGE9ZHVnKSANCg0KI2FsdGVybmF0aXZlIHZhcmlhbmNlIGVzdGltYXRlIGZvciBnDQpuMT1hcy5udW1lcmljKGR1ZyR0X3F1YWRfbik7IG4yPWFzLm51bWVyaWMoZHVnJGNfcXVhZF9uKQ0Kbl90aWxkZT1uMipuMS8objIrbjEpDQp2YXJfZF9uLkRFTlM9KCgxLTMvKDQqKG4yK24xLTIpLTEpKV4yKSoobjIrbjEtMikvKG5fdGlsZGUqKG4yK24xLTQpKSAjSGVkZ2VzIHZhcmlhbmNlIHRoYXQgZG9lcyBub3QgY29udGFpbiBkIGh0dHBzOi8vZXNham91cm5hbHMub25saW5lbGlicmFyeS53aWxleS5jb20vZG9pL2Z1bGwvMTAuMTAwMi9lY3MyLjI0MTkNCg0KZHVnaGckdmkyPC0gdmFyX2Rfbi5ERU5TDQpkdWdoZzwtZHVnaGcgJT4lIGZpbHRlcih2aTIgPiAwICkgIyBzb21lIFNEID0gMCBtdWNraW5nIHVwIHRoZSBjYWxjcyBiZWxvdz8NCg0KdW5pdCA8LSBmYWN0b3IoMTpsZW5ndGgoZHVnbHIkeWkpKQ0KZHVnbHIkdW5pdCA8LSB1bml0DQoNCnVuaXQgPC0gZmFjdG9yKDE6bGVuZ3RoKGR1Z2N2ciR5aSkpDQpkdWdjdnIkdW5pdCA8LSB1bml0DQoNCnVuaXQgPC0gZmFjdG9yKDE6bGVuZ3RoKGR1Z2hnJHlpKSkNCmR1Z2hnJHVuaXQgPC0gdW5pdA0KDQpkdWdscjwtZHVnbHIgJT4lIGRyb3BfbmEoYyhpZCwgcGxvdF9pZCwgdW5pdCkpDQpkdWdjdnI8LWR1Z2xyICU+JSBkcm9wX25hKGMoaWQsIHBsb3RfaWQsIHVuaXQpKQ0KZHVnaGc8LWR1Z2hnICU+JSBkcm9wX25hKGMoaWQsIHBsb3RfaWQsIHVuaXQpKQ0KDQpkdWdscjwtZHVnbHIgJT4lIGRyb3BfbmEodF9xc2l6ZV9tMikNCmR1Z2N2cjwtZHVnbHIgJT4lIGRyb3BfbmEodF9xc2l6ZV9tMikNCmR1Z2hnPC1kdWdoZyAlPiUgZHJvcF9uYSh0X3FzaXplX20yKQ0KDQojcmFuZG9tLWVmZmVjdHMsIGNvbnZlbnRpb25hbCB3ZWlnaHRlZCBtZXRhLWFuYWx5c2lzDQpsci5tYS5yYW4gPC0gcm1hLm12KHlpPXlpLCBWPXZpLCBkYXRhPWR1Z2xyLCBtZXRob2Q9IlJFTUwiLCByYW5kb20gPSBsaXN0KH4xIHwgaWQsIH4xIHwgcGxvdF9pZCwgfjEgfCB1bml0KSkNCiN1bndlaWdodGVkIA0KbHIubWEudW4gPC0gcm1hLm12KHlpPXlpLCBWPXZpLCBkYXRhPWR1Z2xyLCBtZXRob2Q9IlJFTUwiLCByYW5kb209IGxpc3QofjEgfCBpZCwgfjEgfCBwbG90X2lkLCB+MSB8IHVuaXQpLCBXPTEpDQojZml4ZWQtZWZmZWN0cywgY29udmVudGlvbmFsIHdlaWdodGVkIG1ldGEtYW5hbHlzaXMNCmxyLm1hLmZpeCA8LSBybWEubXYoeWk9eWksIFY9dmksIGRhdGE9ZHVnbHIsIG1ldGhvZD0iUkVNTCIsIHJhbmRvbT0gbGlzdCh+MSB8IGlkLCB+MSB8IHBsb3RfaWQsIH4xIHwgdW5pdCksIFcgPSAxL3ZpKQ0KDQojcmFuZG9tLWVmZmVjdHMsIGNvbnZlbnRpb25hbCB3ZWlnaHRlZCBtZXRhLWFuYWx5c2lzDQpjdnIubWEucmFuIDwtIHJtYS5tdih5aT15aSwgVj12aSwgZGF0YT1kdWdjdnIsIG1ldGhvZD0iUkVNTCIsIHJhbmRvbSA9IGxpc3QofjEgfCBpZCwgfjEgfCBwbG90X2lkLCB+MSB8IHVuaXQpKQ0KI3Vud2VpZ2h0ZWQgDQpjdnIubWEudW4gPC0gcm1hLm12KHlpPXlpLCBWPXZpLCBkYXRhPWR1Z2N2ciwgbWV0aG9kPSJSRU1MIiwgcmFuZG9tPSBsaXN0KH4xIHwgaWQsIH4xIHwgcGxvdF9pZCwgfjEgfCB1bml0KSwgVz0xKQ0KI2ZpeGVkLWVmZmVjdHMsIGNvbnZlbnRpb25hbCB3ZWlnaHRlZCBtZXRhLWFuYWx5c2lzDQpjdnIubWEuZml4IDwtIHJtYS5tdih5aT15aSwgVj12aSwgZGF0YT1kdWdjdnIsIG1ldGhvZD0iUkVNTCIsIHJhbmRvbT0gbGlzdCh+MSB8IGlkLCB+MSB8IHBsb3RfaWQsIH4xIHwgdW5pdCksIFcgPSAxL3ZpKQ0KDQojcmFuZG9tLWVmZmVjdHMsIGNvbnZlbnRpb25hbCB3ZWlnaHRlZCBtZXRhLWFuYWx5c2lzDQpoZy5tYS5yYW4gPC0gcm1hLm12KHlpPXlpLCBWPXZpLCBkYXRhPWR1Z2hnLCBtZXRob2Q9IlJFTUwiLCByYW5kb209IGxpc3QofjEgfCBpZCwgfjEgfCBwbG90X2lkLCB+MSB8IHVuaXQpKQ0KI3Vud2VpZ2h0ZWQgDQpoZy5tYS51biA8LSBybWEubXYoeWk9eWksIFY9dmksIGRhdGE9ZHVnaGcsIG1ldGhvZD0iUkVNTCIsIHJhbmRvbT0gbGlzdCh+MSB8IGlkLCB+MSB8IHBsb3RfaWQsIH4xIHwgdW5pdCksIFc9MSkNCiNmaXhlZC1lZmZlY3RzLCBjb252ZW50aW9uYWwgd2VpZ2h0ZWQgbWV0YS1hbmFseXNpcw0KaGcubWEuZml4IDwtIHJtYS5tdih5aT15aSwgVj12aSwgZGF0YT1kdWdoZywgbWV0aG9kPSJSRU1MIiwgcmFuZG9tPSBsaXN0KH4xIHwgaWQsIH4xIHwgcGxvdF9pZCwgfjEgfCB1bml0KSwgVyA9IDEvdmkpDQoNCmhnLm1hLnJhbi5kX2FsdCA8LSBybWEubXYoeWk9eWksIFY9dmkyLCBkYXRhPWR1Z2hnLCBtZXRob2Q9IlJFTUwiLCByYW5kb209IGxpc3QofjEgfCBpZCwgfjEgfCBwbG90X2lkLCB+MSB8IHVuaXQpKQ0KDQpoZy5tYS5maXguZGFsdCA8LSBybWEubXYoeWk9eWksIFY9dmkyLCBkYXRhPWR1Z2hnLCBtZXRob2Q9IlJFTUwiLCByYW5kb209IGxpc3QofjEgfCBpZCwgfjEgfCBwbG90X2lkLCB+MSB8IHVuaXQpLCBXID0gMS92aTIpDQoNCg0KDQpkdWdscjwtZHVnbHIgJT4lIG11dGF0ZShpZCA9IGFzLmZhY3RvcihpZCkpICMgY3VycmVudGx5IHRoaW5rcyBpZCBpcyBudW1lcmljIC0gaXMgdGhhdCBhbiBpc3N1ZT8NCmR1Z2N2cjwtZHVnY3ZyICU+JSBtdXRhdGUoaWQgPSBhcy5mYWN0b3IoaWQpKSAjIGN1cnJlbnRseSB0aGlua3MgaWQgaXMgbnVtZXJpYyAtIGlzIHRoYXQgYW4gaXNzdWU/DQpkdWdoZzwtZHVnaGcgJT4lIG11dGF0ZShpZCA9IGFzLmZhY3RvcihpZCkpICMgY3VycmVudGx5IHRoaW5rcyBpZCBpcyBudW1lcmljIC0gaXMgdGhhdCBhbiBpc3N1ZT8NCg0KTnZzQS5MUiA8LSBnZ3Bsb3QoZHVnbHIpICsgZ2VvbV9wb2ludChhZXMoeD1sb2codF9xc2l6ZV9tMi8xMDAwMCksIHk9dF9xdWFkX24sIHNpemU9MS92aSxjb2w9aWQsYWxwaGE9MC4zKSkrIHRoZW1lX2J3KCkgKyB0aGVtZShwYW5lbC5ncmlkLm1ham9yID0gZWxlbWVudF9ibGFuaygpLA0KcGFuZWwuZ3JpZC5taW5vciA9IGVsZW1lbnRfYmxhbmsoKSwgYXhpcy5saW5lID0gZWxlbWVudF9saW5lKGNvbG91ciA9ICJibGFjayIpKSsgeWxhYihleHByZXNzaW9uKGl0YWxpYyhOKSkpK3hsYWIoIkxvZyhQbG90IHNpemUgKGhhKSkiKStnZW9tX2hsaW5lKHlpbnRlcmNlcHQgPSAwLCBsaW5ldHlwZSA9ICJkYXNoZWQiKSArIHNjYWxlX3NpemVfY29udGludW91cyhuYW1lID0gZXhwcmVzc2lvbihwYXN0ZSgiMS8iLGl0YWxpYyhWKSkpLGxhYmVscz1OVUxMKStzY2FsZV9jb2xvcl9kaXNjcmV0ZSgiIiwgZ3VpZGU9Rikrc2NhbGVfYWxwaGEoIiIsZ3VpZGU9RikrDQogICAgdGhlbWUocGxvdC50aXRsZSA9IGVsZW1lbnRfdGV4dChzaXplID0gOCkpK3RoZW1lKGF4aXMudGV4dC54PWVsZW1lbnRfdGV4dChzaXplPXJlbCgwLjcpKSkNCg0KTnZzQS5DVlIgPC0gZ2dwbG90KGR1Z2N2cikgKyBnZW9tX3BvaW50KGFlcyh4PWxvZyh0X3FzaXplX20yLzEwMDAwKSwgeT10X3F1YWRfbiwgc2l6ZT0xL3ZpLGNvbD1pZCxhbHBoYT0wLjMpKSsgdGhlbWVfYncoKSArIHRoZW1lKHBhbmVsLmdyaWQubWFqb3IgPSBlbGVtZW50X2JsYW5rKCksDQpwYW5lbC5ncmlkLm1pbm9yID0gZWxlbWVudF9ibGFuaygpLCBheGlzLmxpbmUgPSBlbGVtZW50X2xpbmUoY29sb3VyID0gImJsYWNrIikpKyB5bGFiKGV4cHJlc3Npb24oaXRhbGljKE4pKSkreGxhYigiTG9nKFBsb3Qgc2l6ZSAoaGEpKSIpK2dlb21faGxpbmUoeWludGVyY2VwdCA9IDAsIGxpbmV0eXBlID0gImRhc2hlZCIpICsgc2NhbGVfc2l6ZV9jb250aW51b3VzKG5hbWUgPSBleHByZXNzaW9uKHBhc3RlKCIxLyIsaXRhbGljKFYpKSksbGFiZWxzPU5VTEwpK3NjYWxlX2NvbG9yX2Rpc2NyZXRlKCIiLCBndWlkZT1GKStzY2FsZV9hbHBoYSgiIixndWlkZT1GKSsNCiAgICB0aGVtZShwbG90LnRpdGxlID0gZWxlbWVudF90ZXh0KHNpemUgPSA4KSkrdGhlbWUoYXhpcy50ZXh0Lng9ZWxlbWVudF90ZXh0KHNpemU9cmVsKDAuNykpKQ0KDQpOdnNBLmcgPC0gZ2dwbG90KGR1Z2hnKSArIGdlb21fcG9pbnQoYWVzKHg9bG9nKHRfcXNpemVfbTIvMTAwMDApLCB5PXRfcXVhZF9uLCBzaXplPTEvdmksIGNvbG9yPWlkLGFscGhhPTAuMykpKyB0aGVtZV9idygpICsgdGhlbWUocGFuZWwuZ3JpZC5tYWpvciA9IGVsZW1lbnRfYmxhbmsoKSwNCnBhbmVsLmdyaWQubWlub3IgPSBlbGVtZW50X2JsYW5rKCksIGF4aXMubGluZSA9IGVsZW1lbnRfbGluZShjb2xvdXIgPSAiYmxhY2siKSkrIHlsYWIoZXhwcmVzc2lvbihpdGFsaWMoTikpKSt4bGFiKCJMb2coUGxvdCBzaXplIChoYSkpIikrZ2VvbV9obGluZSh5aW50ZXJjZXB0ID0gMCwgbGluZXR5cGUgPSAiZGFzaGVkIikgKyBzY2FsZV9zaXplX2NvbnRpbnVvdXMobmFtZSA9IGV4cHJlc3Npb24ocGFzdGUoIjEvIixpdGFsaWMoVikpKSxsYWJlbHM9TlVMTCkrc2NhbGVfY29sb3JfZGlzY3JldGUoIiIsIGd1aWRlPUYpK3NjYWxlX2FscGhhKCIiLGd1aWRlPUYpKyANCiAgICB0aGVtZShwbG90LnRpdGxlID0gZWxlbWVudF90ZXh0KHNpemUgPSA4KSkrdGhlbWUoYXhpcy50ZXh0Lng9ZWxlbWVudF90ZXh0KHNpemU9cmVsKDAuNykpKQ0KDQoNCkxSdnNBIDwtIGdncGxvdChkdWdscikgKyBnZW9tX3BvaW50KGFlcyh4PWxvZyh0X3FzaXplX20yLzEwMDAwKSwgeT15aSwgc2l6ZT0xL3ZpLCBjb2xvcj1pZCxhbHBoYT0wLjMpKSsgdGhlbWVfYncoKSArIHRoZW1lKHBhbmVsLmdyaWQubWFqb3IgPSBlbGVtZW50X2JsYW5rKCksDQpwYW5lbC5ncmlkLm1pbm9yID0gZWxlbWVudF9ibGFuaygpLCBheGlzLmxpbmUgPSBlbGVtZW50X2xpbmUoY29sb3VyID0gImJsYWNrIikpKyB5bGFiKGV4cHJlc3Npb24oaXRhbGljKCJMUiIpKSkreGxhYigiTG9nKFBsb3Qgc2l6ZSAoaGEpKSIpK2dlb21faGxpbmUoeWludGVyY2VwdCA9IDAsIGxpbmV0eXBlID0gImRhc2hlZCIpICsgc2NhbGVfc2l6ZV9jb250aW51b3VzKG5hbWUgPSBleHByZXNzaW9uKHBhc3RlKCIxLyIsaXRhbGljKFYpKSksbGFiZWxzPU5VTEwpK3NjYWxlX2NvbG9yX2Rpc2NyZXRlKCJzdHVkeSIpK3NjYWxlX2NvbG9yX2Rpc2NyZXRlKCIiLCBndWlkZT1GKStzY2FsZV9hbHBoYSgiIixndWlkZT1GKSsgDQogICAgdGhlbWUocGxvdC50aXRsZSA9IGVsZW1lbnRfdGV4dChzaXplID0gOCkpK3RoZW1lKGF4aXMudGV4dC54PWVsZW1lbnRfdGV4dChzaXplPXJlbCgwLjcpKSkNCg0KQ1ZSdnNBIDwtIGdncGxvdChkdWdjdnIpICsgZ2VvbV9wb2ludChhZXMoeD1sb2codF9xc2l6ZV9tMi8xMDAwMCksIHk9eWksIHNpemU9MS92aSwgY29sb3I9aWQsYWxwaGE9MC4zKSkrIHRoZW1lX2J3KCkgKyB0aGVtZShwYW5lbC5ncmlkLm1ham9yID0gZWxlbWVudF9ibGFuaygpLA0KcGFuZWwuZ3JpZC5taW5vciA9IGVsZW1lbnRfYmxhbmsoKSwgYXhpcy5saW5lID0gZWxlbWVudF9saW5lKGNvbG91ciA9ICJibGFjayIpKSsgeWxhYihleHByZXNzaW9uKGl0YWxpYygiQ1ZSIikpKSt4bGFiKCJMb2coUGxvdCBzaXplIChoYSkpIikrZ2VvbV9obGluZSh5aW50ZXJjZXB0ID0gMCwgbGluZXR5cGUgPSAiZGFzaGVkIikgKyBzY2FsZV9zaXplX2NvbnRpbnVvdXMobmFtZSA9IGV4cHJlc3Npb24ocGFzdGUoIjEvIixpdGFsaWMoVikpKSxsYWJlbHM9TlVMTCkrc2NhbGVfY29sb3JfZGlzY3JldGUoInN0dWR5Iikrc2NhbGVfY29sb3JfZGlzY3JldGUoIiIsIGd1aWRlPUYpK3NjYWxlX2FscGhhKCIiLGd1aWRlPUYpKyANCiAgICB0aGVtZShwbG90LnRpdGxlID0gZWxlbWVudF90ZXh0KHNpemUgPSA4KSkrdGhlbWUoYXhpcy50ZXh0Lng9ZWxlbWVudF90ZXh0KHNpemU9cmVsKDAuNykpKQ0KDQoNCg0KZ3ZzQSA8LSAgZ2dwbG90KGR1Z2hnKSArIGdlb21fcG9pbnQoYWVzKHg9bG9nKHRfcXNpemVfbTIvMTAwMDApLCB5PXlpLCBzaXplPTEvdmksIGNvbG9yPWlkLGFscGhhPTAuMykpKyB0aGVtZV9idygpICsgdGhlbWUocGFuZWwuZ3JpZC5tYWpvciA9IGVsZW1lbnRfYmxhbmsoKSwNCnBhbmVsLmdyaWQubWlub3IgPSBlbGVtZW50X2JsYW5rKCksIGF4aXMubGluZSA9IGVsZW1lbnRfbGluZShjb2xvdXIgPSAiYmxhY2siKSkrIHlsYWIoZXhwcmVzc2lvbihpdGFsaWMoZykpKSt4bGFiKCJMb2coUGxvdCBzaXplIChoYSkpIikrZ2VvbV9obGluZSh5aW50ZXJjZXB0ID0gMCwgbGluZXR5cGUgPSAiZGFzaGVkIikgK3NjYWxlX3NpemVfY29udGludW91cyhuYW1lID0gZXhwcmVzc2lvbihwYXN0ZSgiMS8iLGl0YWxpYyhWKSkpLGxhYmVscz1OVUxMKStzY2FsZV9jb2xvcl9kaXNjcmV0ZSgiIiwgZ3VpZGU9Rikrc2NhbGVfYWxwaGEoIiIsZ3VpZGU9RikrIA0KICAgIHRoZW1lKHBsb3QudGl0bGUgPSBlbGVtZW50X3RleHQoc2l6ZSA9IDgpKSt0aGVtZShheGlzLnRleHQueD1lbGVtZW50X3RleHQoc2l6ZT1yZWwoMC43KSkpDQoNCg0KTFIuVmFydnNBIDwtIGdncGxvdChkdWdscikgKyBnZW9tX3BvaW50KGFlcyh4PWxvZyh0X3FzaXplX20yLzEwMDAwKSwgeT12aSwgc2l6ZT10X3F1YWRfbiwgY29sb3I9aWQsYWxwaGE9MC4zKSkrIHRoZW1lX2J3KCkgKyB0aGVtZShwYW5lbC5ncmlkLm1ham9yID0gZWxlbWVudF9ibGFuaygpLA0KcGFuZWwuZ3JpZC5taW5vciA9IGVsZW1lbnRfYmxhbmsoKSwgYXhpcy5saW5lID0gZWxlbWVudF9saW5lKGNvbG91ciA9ICJibGFjayIpKSsgeWxhYihleHByZXNzaW9uKHBhc3RlKCJWYXJpYW5jZSBvZiAiLGl0YWxpYyhMUikpKSkreGxhYigiTG9nKFBsb3Qgc2l6ZSAoaGEpKSIpK2dlb21faGxpbmUoeWludGVyY2VwdCA9IDAsIGxpbmV0eXBlID0gImRhc2hlZCIpICtzY2FsZV9zaXplX2NvbnRpbnVvdXMobmFtZSA9IGV4cHJlc3Npb24oaXRhbGljKE4pKSxsYWJlbHM9TlVMTCkrc2NhbGVfY29sb3JfZGlzY3JldGUoIiIsIGd1aWRlPUYpK3NjYWxlX2FscGhhKCIiLGd1aWRlPUYpKyANCiAgICB0aGVtZShwbG90LnRpdGxlID0gZWxlbWVudF90ZXh0KHNpemUgPSA4KSkrdGhlbWUoYXhpcy50ZXh0Lng9ZWxlbWVudF90ZXh0KHNpemU9cmVsKDAuNykpKQ0KDQpDVlIuVmFydnNBIDwtIGdncGxvdChkdWdjdnIpICsgZ2VvbV9wb2ludChhZXMoeD1sb2codF9xc2l6ZV9tMi8xMDAwMCksIHk9dmksIHNpemU9dF9xdWFkX24sIGNvbG9yPWlkLGFscGhhPTAuMykpKyB0aGVtZV9idygpICsgdGhlbWUocGFuZWwuZ3JpZC5tYWpvciA9IGVsZW1lbnRfYmxhbmsoKSwNCnBhbmVsLmdyaWQubWlub3IgPSBlbGVtZW50X2JsYW5rKCksIGF4aXMubGluZSA9IGVsZW1lbnRfbGluZShjb2xvdXIgPSAiYmxhY2siKSkrIHlsYWIoZXhwcmVzc2lvbihwYXN0ZSgiVmFyaWFuY2Ugb2YgIixpdGFsaWMoQ1ZSKSkpKSt4bGFiKCJMb2coUGxvdCBzaXplIChoYSkpIikrZ2VvbV9obGluZSh5aW50ZXJjZXB0ID0gMCwgbGluZXR5cGUgPSAiZGFzaGVkIikgK3NjYWxlX3NpemVfY29udGludW91cyhuYW1lID0gZXhwcmVzc2lvbihpdGFsaWMoTikpLGxhYmVscz1OVUxMKStzY2FsZV9jb2xvcl9kaXNjcmV0ZSgiIiwgZ3VpZGU9Rikrc2NhbGVfYWxwaGEoIiIsZ3VpZGU9RikrIA0KICAgIHRoZW1lKHBsb3QudGl0bGUgPSBlbGVtZW50X3RleHQoc2l6ZSA9IDgpKSt0aGVtZShheGlzLnRleHQueD1lbGVtZW50X3RleHQoc2l6ZT1yZWwoMC43KSkpDQoNCmcuVmFydnNBIDwtIGdncGxvdChkdWdoZykgKyBnZW9tX3BvaW50KGFlcyh4PWxvZyh0X3FzaXplX20yLzEwMDAwKSwgeT12aSwgc2l6ZT10X3F1YWRfbiwgY29sb3I9aWQsYWxwaGE9MC4zKSkrIHRoZW1lX2J3KCkgKyB0aGVtZShwYW5lbC5ncmlkLm1ham9yID0gZWxlbWVudF9ibGFuaygpLA0KcGFuZWwuZ3JpZC5taW5vciA9IGVsZW1lbnRfYmxhbmsoKSwgYXhpcy5saW5lID0gZWxlbWVudF9saW5lKGNvbG91ciA9ICJibGFjayIpKSsgeWxhYihleHByZXNzaW9uKHBhc3RlKCJWYXJpYW5jZSBvZiAiLGl0YWxpYyhnKSkpKSt4bGFiKCJMb2coUGxvdCBzaXplIChoYSkpIikrZ2VvbV9obGluZSh5aW50ZXJjZXB0ID0gMCwgbGluZXR5cGUgPSAiZGFzaGVkIikgK3NjYWxlX3NpemVfY29udGludW91cyhuYW1lID0gZXhwcmVzc2lvbihpdGFsaWMoTikpLGxhYmVscz1OVUxMKStzY2FsZV9jb2xvcl9kaXNjcmV0ZSgiIiwgZ3VpZGU9Rikrc2NhbGVfYWxwaGEoIiIsZ3VpZGU9RikrIA0KICAgIHRoZW1lKHBsb3QudGl0bGUgPSBlbGVtZW50X3RleHQoc2l6ZSA9IDgpKSt0aGVtZShheGlzLnRleHQ9ZWxlbWVudF90ZXh0KHNpemU9cmVsKDAuNykpKQ0KDQoNCg0KZ3JpZEV4dHJhOjpncmlkLmFycmFuZ2UoTnZzQS5nLCBndnNBLCBnLlZhcnZzQSxOdnNBLkxSLCAgTFJ2c0EsTFIuVmFydnNBLCBOdnNBLkNWUiwgIENWUnZzQSxDVlIuVmFydnNBLCBuY29sPTMpDQoNCg0KaGRlZmYgPC0gZGF0YS5mcmFtZShlc3RpbWF0ZT1jKGhnLm1hLnJhbiRiLGhnLm1hLnVuJGIsaGcubWEuZml4JGIsaGcubWEucmFuLmRfYWx0JGIsaGcubWEuZml4LmRhbHQkYiksY2kudXA9YyhoZy5tYS5yYW4kY2kudWIsaGcubWEudW4kY2kudWIsaGcubWEuZml4JGNpLnViLCBoZy5tYS5yYW4uZF9hbHQkY2kudWIsaGcubWEuZml4LmRhbHQkY2kudWIpLCBjaS5sbz1jKGhnLm1hLnJhbiRjaS5sYixoZy5tYS51biRjaS5sYixoZy5tYS5maXgkY2kubGIsaGcubWEucmFuLmRfYWx0JGNpLmxiLGhnLm1hLmZpeC5kYWx0JGNpLmxiKSwgd2VpZ2h0aW5nPWMoIlIiLCAiVSIsICJGIiwgIlIiLCAiRiIpLCB2dHlwZT1jKCJkIiwiZCIsImQiLCJkX2FsdCIsImRfYWx0IikpDQoNCmhkZWZmIDwtIGhkZWZmICU+JQ0KYXJyYW5nZSh3ZWlnaHRpbmcpICU+JSAgICAjIEZpcnN0IHNvcnQgYnkgdmFsLiBUaGlzIHNvcnQgdGhlIGRhdGFmcmFtZSBidXQgTk9UIHRoZSBmYWN0b3IgbGV2ZWxzDQogIG11dGF0ZSh3ZWlnaHRpbmc9ZmFjdG9yKHdlaWdodGluZywgbGV2ZWxzPQ0KICAgICAgICAgICAgICAgICAgICAgICAgICAgIGMoIlIiLCAiRiIsICJVIikpKSAgICAjIFRoaXMgdHJpY2sgdXBkYXRlIHRoZSBmYWN0b3IgbGV2ZWxzDQoNCmhkZWZmLnAuYSA8LWdncGxvdChoZGVmZikgKyBnZW9tX3BvaW50KGFlcyh5ID0gZXN0aW1hdGUsIHggPSB3ZWlnaHRpbmcsIGNvbG91cj12dHlwZSksIHBvc2l0aW9uPXBvc2l0aW9uX2RvZGdlKHdpZHRoID0gMC41KSkgKyBnZW9tX2Vycm9yYmFyKGFlcyh4PXdlaWdodGluZyx5bWluPWNpLmxvLCB5bWF4PWNpLnVwLCB3aWR0aCA9IDAuMSwgY29sb3VyPXZ0eXBlKSwsIHBvc2l0aW9uPXBvc2l0aW9uX2RvZGdlKHdpZHRoID0gMC41KSkrDQogICBnZW9tX2FibGluZShpbnRlcmNlcHQgPSAwLCBzbG9wZT0wLCBjb2xvdXIgPSAiZGFya2dyYXkiLCBsaW5ldHlwZT0iZGFzaGVkIiwpICsNCiAgeGxhYihOVUxMKSArDQogIHlsYWIoZXhwcmVzc2lvbihpdGFsaWMoZykpKSsNCiAgdGhlbWVfYncoKSArIA0KICB0aGVtZShwbG90LnRpdGxlID0gZWxlbWVudF90ZXh0KHNpemU9MTApLGF4aXMudGl0bGUgPSBlbGVtZW50X3RleHQoc2l6ZT0xMCksYXhpcy50ZXh0ID0gZWxlbWVudF90ZXh0KHNpemU9OSkgKQ0KaGRlZmYucC5hIDwtaGRlZmYucC5hICt0aGVtZShheGlzLnRleHQueCA9IGVsZW1lbnRfdGV4dChhbmdsZT0wLGZhY2UgPSBjKHJlcCgncGxhaW4nLDE2KSwgJ2JvbGQnLCAnYm9sZCcpKSkgK3NjYWxlX2NvbG9yX21hbnVhbCh2YWx1ZXM9YygiYmxhY2siLCAiZGFya2dyYXkiKSkNCg0KbHJlZmYgPC0gZGF0YS5mcmFtZShlc3RpbWF0ZT1jKGxyLm1hLnJhbiRiLGxyLm1hLnVuJGIsbHIubWEuZml4JGIpLGNpLnVwPWMobHIubWEucmFuJGNpLnViLGxyLm1hLnVuJGNpLnViLGxyLm1hLmZpeCRjaS51YiksIGNpLmxvPWMobHIubWEucmFuJGNpLmxiLGxyLm1hLnVuJGNpLmxiLGxyLm1hLmZpeCRjaS5sYiksIHdlaWdodGluZz1jKCJSIiwgIlUiLCAiRiIpKQ0KbHJlZmYgPC0gbHJlZmYgJT4lDQphcnJhbmdlKHdlaWdodGluZykgJT4lICAgICMgRmlyc3Qgc29ydCBieSB2YWwuIFRoaXMgc29ydCB0aGUgZGF0YWZyYW1lIGJ1dCBOT1QgdGhlIGZhY3RvciBsZXZlbHMNCiAgbXV0YXRlKHdlaWdodGluZz1mYWN0b3Iod2VpZ2h0aW5nLCBsZXZlbHM9DQogICAgICAgICAgICAgICAgICAgICAgICAgICAgYygiUiIsICJGIiwgIlUiKSkpICAgICMgVGhpcyB0cmljayB1cGRhdGUgdGhlIGZhY3RvciBsZXZlbHMNCg0KDQpscmVmZi5wLmEgPC1nZ3Bsb3QobHJlZmYpICsgZ2VvbV9wb2ludChhZXMoeSA9IGVzdGltYXRlLCB4ID0gd2VpZ2h0aW5nKSkgKyBnZW9tX2Vycm9yYmFyKGFlcyh4PXdlaWdodGluZyx5bWluPWNpLmxvLCB5bWF4PWNpLnVwLCB3aWR0aCA9IDAuMSkpKw0KICBnZW9tX2FibGluZShpbnRlcmNlcHQgPSAwLCBzbG9wZT0wLCBjb2xvdXIgPSAiZGFya2dyYXkiLCBsaW5ldHlwZT0iZGFzaGVkIikgKw0KICB4bGFiKE5VTEwpICsNCiB5bGFiKGV4cHJlc3Npb24oaXRhbGljKExSKSkpKw0KICB0aGVtZV9idygpICsgDQogIHRoZW1lKHBsb3QudGl0bGUgPSBlbGVtZW50X3RleHQoc2l6ZT0xMCksYXhpcy50aXRsZSA9IGVsZW1lbnRfdGV4dChzaXplPTEwKSxheGlzLnRleHQgPSBlbGVtZW50X3RleHQoc2l6ZT05KSApDQpscmVmZi5wLmEgPC1scmVmZi5wLmEgK3RoZW1lKGF4aXMudGV4dC54ID0gZWxlbWVudF90ZXh0KGFuZ2xlPTAsZmFjZSA9IGMocmVwKCdwbGFpbicsMTYpLCAnYm9sZCcsICdib2xkJykpKQ0KDQpjdnJlZmYgPC0gZGF0YS5mcmFtZShlc3RpbWF0ZT1jKGN2ci5tYS5yYW4kYixjdnIubWEudW4kYixjdnIubWEuZml4JGIpLGNpLnVwPWMoY3ZyLm1hLnJhbiRjaS51YixjdnIubWEudW4kY2kudWIsY3ZyLm1hLmZpeCRjaS51YiksIGNpLmxvPWMoY3ZyLm1hLnJhbiRjaS5sYixjdnIubWEudW4kY2kubGIsY3ZyLm1hLmZpeCRjaS5sYiksIHdlaWdodGluZz1jKCJSIiwgIlUiLCAiRiIpKQ0KY3ZyZWZmIDwtIGN2cmVmZiAlPiUNCmFycmFuZ2Uod2VpZ2h0aW5nKSAlPiUgICAgIyBGaXJzdCBzb3J0IGJ5IHZhbC4gVGhpcyBzb3J0IHRoZSBkYXRhZnJhbWUgYnV0IE5PVCB0aGUgZmFjdG9yIGxldmVscw0KICBtdXRhdGUod2VpZ2h0aW5nPWZhY3Rvcih3ZWlnaHRpbmcsIGxldmVscz0NCiAgICAgICAgICAgICAgICAgICAgICAgICAgICBjKCJSIiwgIkYiLCAiVSIpKSkgICAgIyBUaGlzIHRyaWNrIHVwZGF0ZSB0aGUgZmFjdG9yIGxldmVscw0KDQoNCmN2cmVmZi5wLmEgPC1nZ3Bsb3QoY3ZyZWZmKSArIGdlb21fcG9pbnQoYWVzKHkgPSBlc3RpbWF0ZSwgeCA9IHdlaWdodGluZykpICsgZ2VvbV9lcnJvcmJhcihhZXMoeD13ZWlnaHRpbmcseW1pbj1jaS5sbywgeW1heD1jaS51cCwgd2lkdGggPSAwLjEpKSsNCiAgZ2VvbV9hYmxpbmUoaW50ZXJjZXB0ID0gMCwgc2xvcGU9MCwgY29sb3VyID0gImRhcmtncmF5IiwgbGluZXR5cGU9ImRhc2hlZCIpICsNCiAgeGxhYihOVUxMKSArDQogeWxhYihleHByZXNzaW9uKGl0YWxpYyhjdnIpKSkrDQogIHRoZW1lX2J3KCkgKyANCiAgdGhlbWUocGxvdC50aXRsZSA9IGVsZW1lbnRfdGV4dChzaXplPTEwKSxheGlzLnRpdGxlID0gZWxlbWVudF90ZXh0KHNpemU9MTApLGF4aXMudGV4dCA9IGVsZW1lbnRfdGV4dChzaXplPTkpICkNCmN2cmVmZi5wLmEgPC1jdnJlZmYucC5hICt0aGVtZShheGlzLnRleHQueCA9IGVsZW1lbnRfdGV4dChhbmdsZT0wLGZhY2UgPSBjKHJlcCgncGxhaW4nLDE2KSwgJ2JvbGQnLCAnYm9sZCcpKSkNCg0KDQoNCmdyaWRFeHRyYTo6Z3JpZC5hcnJhbmdlKGhkZWZmLnAuYSwgbHJlZmYucC5hLCBjdnJlZmYucC5hLCBuY29sPTMsIHdpZHRocyA9IGMoMywgMi4zLCAyLjMpKQ0KDQoNCiNMUg0KI3JhbmRvbSwgY29udmVudGlvbmFsbHkgd2VpZ2h0ZWQNCmxyLm1hLnJhbiA8LSBybWEubXYoeWk9c2NhbGUoeWkpLCBWPXZpLCBtb2RzPWxvZyh0X3FzaXplX20yKSwgZGF0YT1kdWdsciwgbWV0aG9kPSJSRU1MIiwgcmFuZG9tPSBsaXN0KH4xIHwgaWQsIH4xIHwgcGxvdF9pZCwgfjEgfCB1bml0KSkNCiMgdW53ZWlnaHRlZCANCmxyLm1hLnVuIDwtIHJtYS5tdih5aT1zY2FsZSh5aSksIFY9dmksIG1vZHM9bG9nKHRfcXNpemVfbTIpLCBkYXRhPWR1Z2xyLCBtZXRob2Q9IlJFTUwiLCByYW5kb209IGxpc3QofjEgfCBpZCwgfjEgfCBwbG90X2lkLCB+MSB8IHVuaXQpLCBXPTEpDQojZml4ZWQgZWZmZWN0IA0KbHIubWEuZml4IDwtIHJtYS5tdih5aT1zY2FsZSh5aSksIFY9dmksIG1vZHM9bG9nKHRfcXNpemVfbTIpLCBkYXRhPWR1Z2xyLCBtZXRob2Q9IlJFTUwiLCByYW5kb209IGxpc3QofjEgfCBpZCwgfjEgfCBwbG90X2lkLCB+MSB8IHVuaXQpLCBXID0gMS92aSkNCg0KDQojY3ZyDQojcmFuZG9tLCBjb252ZW50aW9uYWxseSB3ZWlnaHRlZA0KY3ZyLm1hLnJhbiA8LSBybWEubXYoeWk9c2NhbGUoeWkpLCBWPXZpLCBtb2RzPWxvZyh0X3FzaXplX20yKSwgZGF0YT1kdWdjdnIsIG1ldGhvZD0iUkVNTCIsIHJhbmRvbT0gbGlzdCh+MSB8IGlkLCB+MSB8IHBsb3RfaWQsIH4xIHwgdW5pdCkpDQojIHVud2VpZ2h0ZWQgDQpjdnIubWEudW4gPC0gcm1hLm12KHlpPXNjYWxlKHlpKSwgVj12aSwgbW9kcz1sb2codF9xc2l6ZV9tMiksIGRhdGE9ZHVnY3ZyLCBtZXRob2Q9IlJFTUwiLCByYW5kb209IGxpc3QofjEgfCBpZCwgfjEgfCBwbG90X2lkLCB+MSB8IHVuaXQpLCBXPTEpDQojZml4ZWQgZWZmZWN0IA0KY3ZyLm1hLmZpeCA8LSBybWEubXYoeWk9c2NhbGUoeWkpLCBWPXZpLCBtb2RzPWxvZyh0X3FzaXplX20yKSwgZGF0YT1kdWdjdnIsIG1ldGhvZD0iUkVNTCIsIHJhbmRvbT0gbGlzdCh+MSB8IGlkLCB+MSB8IHBsb3RfaWQsIH4xIHwgdW5pdCksIFcgPSAxL3ZpKQ0KDQojSEcNCiNyYW5kb20sIGNvbnZlbnRpb25hbGx5IHdlaWdodGVkDQpoZy5tYS5yYW4gPC0gcm1hLm12KHlpPXNjYWxlKHlpKSwgVj12aSwgbW9kcz1sb2codF9xc2l6ZV9tMiksIGRhdGE9ZHVnaGcsIG1ldGhvZD0iUkVNTCIsIHJhbmRvbT0gbGlzdCh+MSB8IGlkLCB+MSB8IHBsb3RfaWQsIH4xIHwgdW5pdCkpDQojIHVud2VpZ2h0ZWQgDQpoZy5tYS51biA8LSBybWEubXYoeWk9c2NhbGUoeWkpLCBWPXZpLCBtb2RzPWxvZyh0X3FzaXplX20yKSwgZGF0YT1kdWdoZywgbWV0aG9kPSJSRU1MIiwgcmFuZG9tPSBsaXN0KH4xIHwgaWQsIH4xIHwgcGxvdF9pZCwgfjEgfCB1bml0KSwgVz0xKQ0KI2ZpeGVkIGVmZmVjdHMgDQpoZy5tYS5maXggPC0gcm1hLm12KHlpPXNjYWxlKHlpKSwgVj12aSwgbW9kcz1sb2codF9xc2l6ZV9tMiksIGRhdGE9ZHVnaGcsIG1ldGhvZD0iUkVNTCIsIHJhbmRvbT0gbGlzdCh+MSB8IGlkLCB+MSB8IHBsb3RfaWQsIH4xIHwgdW5pdCksIFcgPSAxL3ZpKQ0KDQpoZy5tYS5yYW4uZF9hbHQgPC0gcm1hLm12KHlpPXNjYWxlKHlpKSwgVj12aTIsIG1vZHM9bG9nKHRfcXNpemVfbTIpLCBkYXRhPWR1Z2hnLCBtZXRob2Q9IlJFTUwiLCByYW5kb209IGxpc3QofjEgfCBpZCwgfjEgfCBwbG90X2lkLCB+MSB8IHVuaXQpKQ0KDQpoZy5tYS5maXguZGFsdCA8LSBybWEubXYoeWk9c2NhbGUoeWkpLCBWPXZpMiwgbW9kcz1sb2codF9xc2l6ZV9tMiksIGRhdGE9ZHVnaGcsIG1ldGhvZD0iUkVNTCIsIHJhbmRvbT0gbGlzdCh+MSB8IGlkLCB+MSB8IHBsb3RfaWQsIH4xIHwgdW5pdCksIFcgPSAxL3ZpMikNCg0KaGRlZmYgPC0gZGF0YS5mcmFtZShlc3RpbWF0ZT1jKGhnLm1hLnJhbiRiWzJdLGhnLm1hLnVuJGJbMl0saGcubWEuZml4JGJbMl0saGcubWEucmFuLmRfYWx0JGJbMl0saGcubWEuZml4LmRhbHQkYlsyXSksY2kudXA9YyhoZy5tYS5yYW4kY2kudWJbMl0saGcubWEudW4kY2kudWJbMl0saGcubWEuZml4JGNpLnViWzJdLCBoZy5tYS5yYW4uZF9hbHQkY2kudWJbMl0saGcubWEuZml4LmRhbHQkY2kudWJbMl0pLCBjaS5sbz1jKGhnLm1hLnJhbiRjaS5sYlsyXSxoZy5tYS51biRjaS5sYlsyXSxoZy5tYS5maXgkY2kubGJbMl0saGcubWEucmFuLmRfYWx0JGNpLmxiWzJdLGhnLm1hLmZpeC5kYWx0JGNpLmxiWzJdKSwgd2VpZ2h0aW5nPWMoIlIiLCAiVSIsICJGIiwgIlIiLCAiRiIpLCB2dHlwZT1jKCJkIiwiZCIsImQiLCJkX2FsdCIsImRfYWx0IikpDQoNCmhkZWZmIDwtIGhkZWZmICU+JQ0KYXJyYW5nZSh3ZWlnaHRpbmcpICU+JSAgICAjIEZpcnN0IHNvcnQgYnkgdmFsLiBUaGlzIHNvcnQgdGhlIGRhdGFmcmFtZSBidXQgTk9UIHRoZSBmYWN0b3IgbGV2ZWxzDQogIG11dGF0ZSh3ZWlnaHRpbmc9ZmFjdG9yKHdlaWdodGluZywgbGV2ZWxzPQ0KICAgICAgICAgICAgICAgICAgICAgICAgICAgIGMoIlIiLCAiRiIsICJVIikpKSAgICAjIFRoaXMgdHJpY2sgdXBkYXRlIHRoZSBmYWN0b3IgbGV2ZWxzDQoNCmhkZWZmLnAuYSA8LWdncGxvdChoZGVmZikgKyBnZW9tX3BvaW50KGFlcyh5ID0gZXN0aW1hdGUsIHggPSB3ZWlnaHRpbmcsIGNvbG91cj12dHlwZSksIHBvc2l0aW9uPXBvc2l0aW9uX2RvZGdlKHdpZHRoID0gMC41KSkgKyBnZW9tX2Vycm9yYmFyKGFlcyh4PXdlaWdodGluZyx5bWluPWNpLmxvLCB5bWF4PWNpLnVwLCB3aWR0aCA9IDAuMSwgY29sb3VyPXZ0eXBlKSwsIHBvc2l0aW9uPXBvc2l0aW9uX2RvZGdlKHdpZHRoID0gMC41KSkrDQogICBnZW9tX2FibGluZShpbnRlcmNlcHQgPSAwLCBzbG9wZT0wLCBjb2xvdXIgPSAiZGFya2dyYXkiLCBsaW5ldHlwZT0iZGFzaGVkIiwpICsNCiAgeGxhYihOVUxMKSArDQogIHlsYWIoIkVmZmVjdCBvZiBwbG90IHNpemUgKHJlZ3Jlc3Npb24gY29lZmZpY2llbnQpIikrDQogIHRoZW1lX2J3KCkgKyANCiAgdGhlbWUocGxvdC50aXRsZSA9IGVsZW1lbnRfdGV4dChzaXplPTEwKSxheGlzLnRpdGxlID0gZWxlbWVudF90ZXh0KHNpemU9MTApLGF4aXMudGV4dCA9IGVsZW1lbnRfdGV4dChzaXplPTkpICkNCmhkZWZmLnAuYSA8LWhkZWZmLnAuYSArdGhlbWUoYXhpcy50ZXh0LnggPSBlbGVtZW50X3RleHQoYW5nbGU9MCxmYWNlID0gYyhyZXAoJ3BsYWluJywxNiksICdib2xkJywgJ2JvbGQnKSkpK3lsaW0oLTEsMS4yKSArc2NhbGVfY29sb3JfbWFudWFsKHZhbHVlcz1jKCJibGFjayIsICJkYXJrZ3JheSIpLCBndWlkZT1GKQ0KDQpscmVmZiA8LSBkYXRhLmZyYW1lKGVzdGltYXRlPWMobHIubWEucmFuJGJbMl0sbHIubWEudW4kYlsyXSxsci5tYS5maXgkYlsyXSksY2kudXA9Yyhsci5tYS5yYW4kY2kudWJbMl0sbHIubWEudW4kY2kudWJbMl0sbHIubWEuZml4JGNpLnViWzJdKSwgY2kubG89Yyhsci5tYS5yYW4kY2kubGJbMl0sbHIubWEudW4kY2kubGJbMl0sbHIubWEuZml4JGNpLmxiWzJdKSwgd2VpZ2h0aW5nPWMoIlIiLCAiVSIsICJGIikpDQpscmVmZiA8LSBscmVmZiAlPiUNCmFycmFuZ2Uod2VpZ2h0aW5nKSAlPiUgICAgIyBGaXJzdCBzb3J0IGJ5IHZhbC4gVGhpcyBzb3J0IHRoZSBkYXRhZnJhbWUgYnV0IE5PVCB0aGUgZmFjdG9yIGxldmVscw0KICBtdXRhdGUod2VpZ2h0aW5nPWZhY3Rvcih3ZWlnaHRpbmcsIGxldmVscz0NCiAgICAgICAgICAgICAgICAgICAgICAgICAgICBjKCJSIiwgIkYiLCAiVSIpKSkgICAgIyBUaGlzIHRyaWNrIHVwZGF0ZSB0aGUgZmFjdG9yIGxldmVscw0KDQoNCmxyZWZmLnAuYSA8LWdncGxvdChscmVmZikgKyBnZW9tX3BvaW50KGFlcyh5ID0gZXN0aW1hdGUsIHggPSB3ZWlnaHRpbmcpKSArIGdlb21fZXJyb3JiYXIoYWVzKHg9d2VpZ2h0aW5nLHltaW49Y2kubG8sIHltYXg9Y2kudXAsIHdpZHRoID0gMC4xKSkrDQogIGdlb21fYWJsaW5lKGludGVyY2VwdCA9IDAsIHNsb3BlPTAsIGNvbG91ciA9ICJkYXJrZ3JheSIsIGxpbmV0eXBlPSJkYXNoZWQiKSArDQogIHhsYWIoTlVMTCkgKw0KICB5bGFiKCJFZmZlY3Qgb2YgcGxvdCBzaXplIChyZWdyZXNzaW9uIGNvZWZmaWNpZW50KSIpKw0KICB0aGVtZV9idygpICsgDQogIHRoZW1lKHBsb3QudGl0bGUgPSBlbGVtZW50X3RleHQoc2l6ZT0xMCksYXhpcy50aXRsZSA9IGVsZW1lbnRfdGV4dChzaXplPTEwKSxheGlzLnRleHQgPSBlbGVtZW50X3RleHQoc2l6ZT05KSApDQpscmVmZi5wLmEgPC1scmVmZi5wLmEgK3RoZW1lKGF4aXMudGV4dC54ID0gZWxlbWVudF90ZXh0KGFuZ2xlPTAsZmFjZSA9IGMocmVwKCdwbGFpbicsMTYpLCAnYm9sZCcsICdib2xkJykpKSt5bGltKC0xLjIsMS41KQ0KDQoNCmN2cmVmZiA8LSBkYXRhLmZyYW1lKGVzdGltYXRlPWMoY3ZyLm1hLnJhbiRiWzJdLGN2ci5tYS51biRiWzJdLGN2ci5tYS5maXgkYlsyXSksY2kudXA9YyhjdnIubWEucmFuJGNpLnViWzJdLGN2ci5tYS51biRjaS51YlsyXSxjdnIubWEuZml4JGNpLnViWzJdKSwgY2kubG89YyhjdnIubWEucmFuJGNpLmxiWzJdLGN2ci5tYS51biRjaS5sYlsyXSxjdnIubWEuZml4JGNpLmxiWzJdKSwgd2VpZ2h0aW5nPWMoIlIiLCAiVSIsICJGIikpDQpjdnJlZmYgPC0gY3ZyZWZmICU+JQ0KYXJyYW5nZSh3ZWlnaHRpbmcpICU+JSAgICAjIEZpcnN0IHNvcnQgYnkgdmFsLiBUaGlzIHNvcnQgdGhlIGRhdGFmcmFtZSBidXQgTk9UIHRoZSBmYWN0b3IgbGV2ZWxzDQogIG11dGF0ZSh3ZWlnaHRpbmc9ZmFjdG9yKHdlaWdodGluZywgbGV2ZWxzPQ0KICAgICAgICAgICAgICAgICAgICAgICAgICAgIGMoIlIiLCAiRiIsICJVIikpKSAgICAjIFRoaXMgdHJpY2sgdXBkYXRlIHRoZSBmYWN0b3IgbGV2ZWxzDQoNCg0KY3ZyZWZmLnAuYSA8LWdncGxvdChjdnJlZmYpICsgZ2VvbV9wb2ludChhZXMoeSA9IGVzdGltYXRlLCB4ID0gd2VpZ2h0aW5nKSkgKyBnZW9tX2Vycm9yYmFyKGFlcyh4PXdlaWdodGluZyx5bWluPWNpLmxvLCB5bWF4PWNpLnVwLCB3aWR0aCA9IDAuMSkpKw0KICBnZW9tX2FibGluZShpbnRlcmNlcHQgPSAwLCBzbG9wZT0wLCBjb2xvdXIgPSAiZGFya2dyYXkiLCBsaW5ldHlwZT0iZGFzaGVkIikgKw0KICB4bGFiKE5VTEwpICsNCiAgeWxhYigiRWZmZWN0IG9mIHBsb3Qgc2l6ZSAocmVncmVzc2lvbiBjb2VmZmljaWVudCkiKSsNCiAgdGhlbWVfYncoKSArIA0KICB0aGVtZShwbG90LnRpdGxlID0gZWxlbWVudF90ZXh0KHNpemU9MTApLGF4aXMudGl0bGUgPSBlbGVtZW50X3RleHQoc2l6ZT0xMCksYXhpcy50ZXh0ID0gZWxlbWVudF90ZXh0KHNpemU9OSkgKQ0KY3ZyZWZmLnAuYSA8LWN2cmVmZi5wLmEgK3RoZW1lKGF4aXMudGV4dC54ID0gZWxlbWVudF90ZXh0KGFuZ2xlPTAsZmFjZSA9IGMocmVwKCdwbGFpbicsMTYpLCAnYm9sZCcsICdib2xkJykpKSt5bGltKC0xLjIsMS41KQ0KDQpncmlkRXh0cmE6OmdyaWQuYXJyYW5nZShoZGVmZi5wLmEsIGxyZWZmLnAuYSwgY3ZyZWZmLnAuYSwgbmNvbD0zLCB3aWR0aHM9YygzLDIuMywgMi4zKSkNCg0KaGcubWEucmFuIDwtIHJtYS5tdih5aT15aSwgVj12aSwgbW9kcz1sb2codF9xc2l6ZV9tMiksIGRhdGE9ZHVnaGcsIG1ldGhvZD0iUkVNTCIsIHJhbmRvbT0gbGlzdCh+MSB8IGlkLCB+MSB8IHBsb3RfaWQsIH4xIHwgdW5pdCkpDQojcmFuZG9tIHVud2VpZ2h0ZWQgKHNhbWUgbWV0YS1lc3QgaWYgZml4ZWQgdW53ZWlnaHRlZCwgYnV0IGRpZmYgc2UpDQpoZy5tYS51biA8LSBybWEubXYoeWk9eWksIFY9dmksIG1vZHM9bG9nKHRfcXNpemVfbTIpLCBkYXRhPWR1Z2hnLCBtZXRob2Q9IlJFTUwiLCByYW5kb209IGxpc3QofjEgfCBpZCwgfjEgfCBwbG90X2lkLCB+MSB8IHVuaXQpLCBXPTEpDQojcmFuZG9tIGVmZmVjdCBidXQgY29udHJvbCB0aGUgd2VpZ2h0cywgZG8gMS92LiBzYW1lIGVzdCBhcyBhIGZpeGVkIGVmZmVjdA0KaGcubWEuZml4IDwtIHJtYS5tdih5aT15aSwgVj12aSwgbW9kcz1sb2codF9xc2l6ZV9tMiksIGRhdGE9ZHVnaGcsIG1ldGhvZD0iUkVNTCIsIHJhbmRvbT0gbGlzdCh+MSB8IGlkLCB+MSB8IHBsb3RfaWQsIH4xIHwgdW5pdCksIFcgPSAxL3ZpKQ0KDQpoZy5tYS5yYW4uZF9hbHQgPC0gcm1hLm12KHlpPXlpLCBWPXZpMiwgbW9kcz1sb2codF9xc2l6ZV9tMiksIGRhdGE9ZHVnaGcsIG1ldGhvZD0iUkVNTCIsIHJhbmRvbT0gbGlzdCh+MSB8IGlkLCB+MSB8IHBsb3RfaWQsIH4xIHwgdW5pdCkpDQoNCmhnLm1hLmZpeC5kYWx0IDwtIHJtYS5tdih5aT15aSwgVj12aTIsIG1vZHM9bG9nKHRfcXNpemVfbTIpLCBkYXRhPWR1Z2hnLCBtZXRob2Q9IlJFTUwiLCByYW5kb209IGxpc3QofjEgfCBpZCwgfjEgfCBwbG90X2lkLCB+MSB8IHVuaXQpLCBXID0gMS92aTIpDQoNCg0KbHIubWEucmFuIDwtIHJtYS5tdih5aT15aSwgVj12aSwgbW9kcz1sb2codF9xc2l6ZV9tMiksIGRhdGE9ZHVnbHIsIG1ldGhvZD0iUkVNTCIsIHJhbmRvbT0gbGlzdCh+MSB8IGlkLCB+MSB8IHBsb3RfaWQsIH4xIHwgdW5pdCkpDQojcmFuZG9tIHVud2VpZ2h0ZWQgKHNhbWUgbWV0YS1lc3QgaWYgZml4ZWQgdW53ZWlnaHRlZCwgYnV0IGRpZmYgc2UpDQpsci5tYS51biA8LSBybWEubXYoeWk9eWksIFY9dmksIG1vZHM9bG9nKHRfcXNpemVfbTIpLCBkYXRhPWR1Z2xyLCBtZXRob2Q9IlJFTUwiLCByYW5kb209IGxpc3QofjEgfCBpZCwgfjEgfCBwbG90X2lkLCB+MSB8IHVuaXQpLCBXPTEpDQojcmFuZG9tIGVmZmVjdCBidXQgY29udHJvbCB0aGUgd2VpZ2h0cywgZG8gMS92LiBzYW1lIGVzdCBhcyBhIGZpeGVkIGVmZmVjdA0KbHIubWEuZml4IDwtIHJtYS5tdih5aT15aSwgVj12aSwgbW9kcz1sb2codF9xc2l6ZV9tMiksIGRhdGE9ZHVnbHIsIG1ldGhvZD0iUkVNTCIsIHJhbmRvbT0gbGlzdCh+MSB8IGlkLCB+MSB8IHBsb3RfaWQsIH4xIHwgdW5pdCksIFcgPSAxL3ZpKQ0KDQoNCmN2ci5tYS5yYW4gPC0gcm1hLm12KHlpPXlpLCBWPXZpLCBtb2RzPWxvZyh0X3FzaXplX20yKSwgZGF0YT1kdWdjdnIsIG1ldGhvZD0iUkVNTCIsIHJhbmRvbT0gbGlzdCh+MSB8IGlkLCB+MSB8IHBsb3RfaWQsIH4xIHwgdW5pdCkpDQojcmFuZG9tIHVud2VpZ2h0ZWQgKHNhbWUgbWV0YS1lc3QgaWYgZml4ZWQgdW53ZWlnaHRlZCwgYnV0IGRpZmYgc2UpDQpjdnIubWEudW4gPC0gcm1hLm12KHlpPXlpLCBWPXZpLCBtb2RzPWxvZyh0X3FzaXplX20yKSwgZGF0YT1kdWdjdnIsIG1ldGhvZD0iUkVNTCIsIHJhbmRvbT0gbGlzdCh+MSB8IGlkLCB+MSB8IHBsb3RfaWQsIH4xIHwgdW5pdCksIFc9MSkNCiNyYW5kb20gZWZmZWN0IGJ1dCBjb250cm9sIHRoZSB3ZWlnaHRzLCBkbyAxL3YuIHNhbWUgZXN0IGFzIGEgZml4ZWQgZWZmZWN0DQpjdnIubWEuZml4IDwtIHJtYS5tdih5aT15aSwgVj12aSwgbW9kcz1sb2codF9xc2l6ZV9tMiksIGRhdGE9ZHVnY3ZyLCBtZXRob2Q9IlJFTUwiLCByYW5kb209IGxpc3QofjEgfCBpZCwgfjEgfCBwbG90X2lkLCB+MSB8IHVuaXQpLCBXID0gMS92aSkNCg0KDQoNCg0KDQpuZXdtb2RzPWRhdGEuZnJhbWUoaW50ZXJjZXB0PWhnLm1hLnJhbiRiWzFdLCB0X3FzaXplX20yPXNlcShtaW4oZHVnaGckdF9xc2l6ZV9tMiksIG1heChkdWdoZyR0X3FzaXplX20yKSwgMC4xKSApDQpuZXdtb2RzPWFzLm1hdHJpeChuZXdtb2RzKQ0KDQpoZy5tYS5yYW4ucHJlZHM9ZGF0YS5mcmFtZShwcmVkaWN0KGhnLm1hLnJhbiwgIGFkZHg9VFJVRSkpDQpoZy5tYS5maXgucHJlZHM9ZGF0YS5mcmFtZShwcmVkaWN0KGhnLm1hLmZpeCwgIGFkZHg9VFJVRSkpDQpoZy5tYS51bi5wcmVkcz1kYXRhLmZyYW1lKHByZWRpY3QoaGcubWEudW4sICBhZGR4PVRSVUUpKQ0KDQpoZy5tYS5yYW4ucD1nZ3Bsb3QoKStnZW9tX3BvaW50KGRhdGE9ZHVnaGcsYWVzKHg9bG9nKHRfcXNpemVfbTIpLCB5PXlpLGNvbG91cj1pZCksIA0KICAgICAgICAgICAgICAgICAgICAgICAgICAgICAgICAgICAgICAgICAgICAgICAjc2l6ZT13ZWlnaHRzKGhnLm1hLnJhbikpLCANCiAgICAgICAgICAgICAgICAgICAgICAgICAgICAgICAgYWxwaGE9MC4zKSArZ2VvbV9saW5lKGFlcyh4PWhnLm1hLnJhbi5wcmVkcyRYLm1vZHMsIHk9aGcubWEucmFuLnByZWRzJHByZWQpKStnZW9tX2xpbmUoYWVzKHg9aGcubWEucmFuLnByZWRzJFgubW9kcywgeT1oZy5tYS5yYW4ucHJlZHMkcHJlZCkpK2dlb21fcmliYm9uKGFlcyh4PWhnLm1hLnJhbi5wcmVkcyRYLm1vZHMseW1pbj1oZy5tYS5yYW4ucHJlZHMkY2kubGIsIHltYXg9aGcubWEucmFuLnByZWRzJGNpLnViICksYWxwaGE9MC4yKStzY2FsZV9zaXplX2NvbnRpbnVvdXMoZ3VpZGU9RkFMU0UpK3NjYWxlX2NvbG9yX2Rpc2NyZXRlKGd1aWRlPUYpK3RoZW1lX2J3KCkreWxhYihleHByZXNzaW9uKGl0YWxpYyhnKSkpK3hsYWIoTlVMTCkrZ2VvbV9obGluZSh5aW50ZXJjZXB0ID0gMCwgbGluZXR5cGUgPSAiZGFzaGVkIikgK2dndGl0bGUoZXhwcmVzc2lvbihwYXN0ZSgiUmFuZG9tLWVmZmVjdHMgbWV0YS1hbmFseXNpcywgd3QgPSAxLygiLCBpdGFsaWMoIlYiKSwiKyIsdGF1XjIsIikiKSkpK3RoZW1lKHBsb3QudGl0bGUgPSBlbGVtZW50X3RleHQoc2l6ZSA9IDgpKSt5bGltKC0xMiw3LjIpDQoNCg0KaGcubWEuZml4LnA9Z2dwbG90KCkrZ2VvbV9wb2ludChkYXRhPWR1Z2hnLGFlcyh4PWxvZyh0X3FzaXplX20yKSwgeT15aSxjb2xvdXI9aWQpLCAjc2l6ZT13ZWlnaHRzKGhnLm1hLmZpeCkpLA0KICAgICAgICAgICAgICAgICAgICAgICAgICAgICAgICAgICAgICAgICAgICAgICBhbHBoYT0wLjMpICtnZW9tX2xpbmUoYWVzKHg9aGcubWEuZml4LnByZWRzJFgubW9kcywgeT1oZy5tYS5maXgucHJlZHMkcHJlZCkpK2dlb21fbGluZShhZXMoeD1oZy5tYS5maXgucHJlZHMkWC5tb2RzLCB5PWhnLm1hLmZpeC5wcmVkcyRwcmVkKSkrZ2VvbV9yaWJib24oYWVzKHg9aGcubWEuZml4LnByZWRzJFgubW9kcyx5bWluPWhnLm1hLmZpeC5wcmVkcyRjaS5sYiwgeW1heD1oZy5tYS5maXgucHJlZHMkY2kudWIgKSxhbHBoYT0wLjIpK3NjYWxlX3NpemVfY29udGludW91cyhndWlkZT1GQUxTRSkrc2NhbGVfY29sb3JfZGlzY3JldGUoZ3VpZGU9RikrdGhlbWVfYncoKSt5bGFiKGV4cHJlc3Npb24oaXRhbGljKGcpKSkreGxhYihOVUxMKStnZW9tX2hsaW5lKHlpbnRlcmNlcHQgPSAwLCBsaW5ldHlwZSA9ICJkYXNoZWQiKStnZ3RpdGxlKGV4cHJlc3Npb24ocGFzdGUoIkZpeGVkLWVmZmVjdHMgbWV0YS1hbmFseXNpcywgd3QgPSAxLygiLCBpdGFsaWMoIlYiKSwiKSIpKSkrdGhlbWUocGxvdC50aXRsZSA9IGVsZW1lbnRfdGV4dChzaXplID0gOCkpK3lsaW0oLTEyLDcuMikNCg0KaGcubWEudW4ucD1nZ3Bsb3QoKStnZW9tX3BvaW50KGRhdGE9ZHVnaGcsYWVzKHg9bG9nKHRfcXNpemVfbTIpLCB5PXlpLGNvbG91cj1pZCwgc2l6ZT0xKSwgYWxwaGE9MC4zKSArZ2VvbV9saW5lKGFlcyh4PWhnLm1hLmZpeC5wcmVkcyRYLm1vZHMsIHk9aGcubWEudW4ucHJlZHMkcHJlZCkpK2dlb21fbGluZShhZXMoeD1oZy5tYS51bi5wcmVkcyRYLm1vZHMsIHk9aGcubWEudW4ucHJlZHMkcHJlZCkpK2dlb21fcmliYm9uKGFlcyh4PWhnLm1hLnVuLnByZWRzJFgubW9kcyx5bWluPWhnLm1hLnVuLnByZWRzJGNpLmxiLCB5bWF4PWhnLm1hLnVuLnByZWRzJGNpLnViICksYWxwaGE9MC4yKStzY2FsZV9jb2xvcl9kaXNjcmV0ZShndWlkZT1GKSt0aGVtZV9idygpK3lsYWIoZXhwcmVzc2lvbihpdGFsaWMoZykpKSt4bGFiKCJMb2coUGxvdCBzaXplIChoYSkpIikrZ2VvbV9obGluZSh5aW50ZXJjZXB0ID0gMCwgbGluZXR5cGUgPSAiZGFzaGVkIikrc2NhbGVfc2l6ZV9jb250aW51b3VzKGd1aWRlPUZBTFNFKStnZ3RpdGxlKCJVbndlaWdodGVkIG1ldGEtYW5hbHlzaXMsIHd0ID0gMSIpK3RoZW1lKHBsb3QudGl0bGUgPSBlbGVtZW50X3RleHQoc2l6ZSA9IDgpKSt5bGltKC0xMiw3LjIpDQoNCg0KDQoNCm5ld21vZHM9ZGF0YS5mcmFtZShpbnRlcmNlcHQ9bHIubWEucmFuJGJbMV0sIHRfcXNpemVfbTI9c2VxKG1pbihkdWdsciR0X3FzaXplX20yKSwgbWF4KGR1Z2xyJHRfcXNpemVfbTIpLCAwLjEpICkNCm5ld21vZHM9YXMubWF0cml4KG5ld21vZHMpDQpoZWFkKG5ld21vZHMpDQpsci5tYS5yYW4ucHJlZHM9ZGF0YS5mcmFtZShwcmVkaWN0KGxyLm1hLnJhbiwgIGFkZHg9VFJVRSkpDQpsci5tYS5maXgucHJlZHM9ZGF0YS5mcmFtZShwcmVkaWN0KGxyLm1hLmZpeCwgIGFkZHg9VFJVRSkpDQpsci5tYS51bi5wcmVkcz1kYXRhLmZyYW1lKHByZWRpY3QobHIubWEudW4sICBhZGR4PVRSVUUpKQ0KDQpsci5tYS5yYW4ucD1nZ3Bsb3QoKStnZW9tX3BvaW50KGRhdGE9ZHVnbHIsYWVzKHg9bG9nKHRfcXNpemVfbTIpLCB5PXlpLGNvbG91cj1pZCksIyBzaXplPXdlaWdodHMobHIubWEucmFuKSksDQogICAgICAgICAgICAgICAgICAgICAgICAgICAgICAgIGFscGhhPTAuMykgK2dlb21fbGluZShhZXMoeD1sci5tYS5yYW4ucHJlZHMkWC5tb2RzLCB5PWxyLm1hLnJhbi5wcmVkcyRwcmVkKSkrZ2VvbV9saW5lKGFlcyh4PWxyLm1hLnJhbi5wcmVkcyRYLm1vZHMsIHk9bHIubWEucmFuLnByZWRzJHByZWQpKStnZW9tX3JpYmJvbihhZXMoeD1sci5tYS5yYW4ucHJlZHMkWC5tb2RzLHltaW49bHIubWEucmFuLnByZWRzJGNpLmxiLCB5bWF4PWxyLm1hLnJhbi5wcmVkcyRjaS51YiApLGFscGhhPTAuMikrc2NhbGVfc2l6ZV9jb250aW51b3VzKGd1aWRlPUZBTFNFKStzY2FsZV9jb2xvcl9kaXNjcmV0ZShndWlkZT1GKSt0aGVtZV9idygpK3lsYWIoZXhwcmVzc2lvbihpdGFsaWMoTFIpKSkreGxhYihOVUxMKStnZW9tX2hsaW5lKHlpbnRlcmNlcHQgPSAwLCBsaW5ldHlwZSA9ICJkYXNoZWQiKSArZ2d0aXRsZSgiIikrdGhlbWUocGxvdC50aXRsZSA9IGVsZW1lbnRfdGV4dChzaXplID0gOCkpK3lsaW0oLTEuNiwxLjIpDQoNCg0KbHIubWEuZml4LnA9Z2dwbG90KCkrZ2VvbV9wb2ludChkYXRhPWR1Z2xyLGFlcyh4PWxvZyh0X3FzaXplX20yKSwgeT15aSxjb2xvdXI9aWQpLCMgc2l6ZT13ZWlnaHRzKGxyLm1hLmZpeCkpLA0KICAgICAgICAgICAgICAgICAgICAgICAgICAgICAgICBhbHBoYT0wLjMpICtnZW9tX2xpbmUoYWVzKHg9bHIubWEuZml4LnByZWRzJFgubW9kcywgeT1sci5tYS5maXgucHJlZHMkcHJlZCkpK2dlb21fbGluZShhZXMoeD1sci5tYS5maXgucHJlZHMkWC5tb2RzLCB5PWxyLm1hLmZpeC5wcmVkcyRwcmVkKSkrZ2VvbV9yaWJib24oYWVzKHg9bHIubWEuZml4LnByZWRzJFgubW9kcyx5bWluPWxyLm1hLmZpeC5wcmVkcyRjaS5sYiwgeW1heD1sci5tYS5maXgucHJlZHMkY2kudWIgKSxhbHBoYT0wLjIpK3NjYWxlX3NpemVfY29udGludW91cyhndWlkZT1GQUxTRSkrc2NhbGVfY29sb3JfZGlzY3JldGUoZ3VpZGU9RikrdGhlbWVfYncoKSt5bGFiKGV4cHJlc3Npb24oaXRhbGljKExSKSkpK3hsYWIoTlVMTCkrZ2VvbV9obGluZSh5aW50ZXJjZXB0ID0gMCwgbGluZXR5cGUgPSAiZGFzaGVkIikrZ2d0aXRsZSgiIikrdGhlbWUocGxvdC50aXRsZSA9IGVsZW1lbnRfdGV4dChzaXplID0gOCkpK2Nvb3JkX2NhcnRlc2lhbih5bGltID0gYygtMS42LCAxLjIpKSANCg0KDQoNCmxyLm1hLnVuLnA9Z2dwbG90KCkrZ2VvbV9wb2ludChkYXRhPWR1Z2xyLGFlcyh4PWxvZyh0X3FzaXplX20yKSwgeT15aSxjb2xvdXI9aWQsIHNpemU9MSksIGFscGhhPTAuMykgK2dlb21fbGluZShhZXMoeD1sci5tYS5maXgucHJlZHMkWC5tb2RzLCB5PWxyLm1hLnVuLnByZWRzJHByZWQpKStnZW9tX2xpbmUoYWVzKHg9bHIubWEudW4ucHJlZHMkWC5tb2RzLCB5PWxyLm1hLnVuLnByZWRzJHByZWQpKStnZW9tX3JpYmJvbihhZXMoeD1sci5tYS51bi5wcmVkcyRYLm1vZHMseW1pbj1sci5tYS51bi5wcmVkcyRjaS5sYiwgeW1heD1sci5tYS51bi5wcmVkcyRjaS51YiApLGFscGhhPTAuMikrc2NhbGVfY29sb3JfZGlzY3JldGUoZ3VpZGU9RikrdGhlbWVfYncoKSt5bGFiKGV4cHJlc3Npb24oaXRhbGljKExSKSkpK3hsYWIoIkxvZyhQbG90IHNpemUgKGhhKSkiKStnZW9tX2hsaW5lKHlpbnRlcmNlcHQgPSAwLCBsaW5ldHlwZSA9ICJkYXNoZWQiKStzY2FsZV9zaXplX2NvbnRpbnVvdXMoZ3VpZGU9RkFMU0UpK2dndGl0bGUoIiIpK3RoZW1lKHBsb3QudGl0bGUgPSBlbGVtZW50X3RleHQoc2l6ZSA9IDgpKSt5bGltKC0xLjYsIDEuMikNCg0KbmV3bW9kcz1kYXRhLmZyYW1lKGludGVyY2VwdD1jdnIubWEucmFuJGJbMV0sIHRfcXNpemVfbTI9c2VxKG1pbihkdWdjdnIkdF9xc2l6ZV9tMiksIG1heChkdWdjdnIkdF9xc2l6ZV9tMiksIDAuMSkgKQ0KbmV3bW9kcz1hcy5tYXRyaXgobmV3bW9kcykNCg0KY3ZyLm1hLnJhbi5wcmVkcz1kYXRhLmZyYW1lKHByZWRpY3QoY3ZyLm1hLnJhbiwgIGFkZHg9VFJVRSkpDQpjdnIubWEuZml4LnByZWRzPWRhdGEuZnJhbWUocHJlZGljdChjdnIubWEuZml4LCAgYWRkeD1UUlVFKSkNCmN2ci5tYS51bi5wcmVkcz1kYXRhLmZyYW1lKHByZWRpY3QoY3ZyLm1hLnVuLCAgYWRkeD1UUlVFKSkNCg0KY3ZyLm1hLnJhbi5wPWdncGxvdCgpK2dlb21fcG9pbnQoZGF0YT1kdWdjdnIsYWVzKHg9bG9nKHRfcXNpemVfbTIpLCB5PXlpLGNvbG91cj1pZCksIyBzaXplPXdlaWdodHMoY3ZyLm1hLnJhbikpLA0KICAgICAgICAgICAgICAgICAgICAgICAgICAgICAgICBhbHBoYT0wLjMpICtnZW9tX2xpbmUoYWVzKHg9Y3ZyLm1hLnJhbi5wcmVkcyRYLm1vZHMsIHk9Y3ZyLm1hLnJhbi5wcmVkcyRwcmVkKSkrZ2VvbV9saW5lKGFlcyh4PWN2ci5tYS5yYW4ucHJlZHMkWC5tb2RzLCB5PWN2ci5tYS5yYW4ucHJlZHMkcHJlZCkpK2dlb21fcmliYm9uKGFlcyh4PWN2ci5tYS5yYW4ucHJlZHMkWC5tb2RzLHltaW49Y3ZyLm1hLnJhbi5wcmVkcyRjaS5sYiwgeW1heD1jdnIubWEucmFuLnByZWRzJGNpLnViICksYWxwaGE9MC4yKStzY2FsZV9zaXplX2NvbnRpbnVvdXMoZ3VpZGU9RkFMU0UpK3NjYWxlX2NvbG9yX2Rpc2NyZXRlKGd1aWRlPUYpK3RoZW1lX2J3KCkreWxhYihleHByZXNzaW9uKGl0YWxpYyhjdnIpKSkreGxhYihOVUxMKStnZW9tX2hsaW5lKHlpbnRlcmNlcHQgPSAwLCBsaW5ldHlwZSA9ICJkYXNoZWQiKSArZ2d0aXRsZSgiIikrdGhlbWUocGxvdC50aXRsZSA9IGVsZW1lbnRfdGV4dChzaXplID0gOCkpK3lsaW0oLTEuNiwxLjIpDQoNCg0KY3ZyLm1hLmZpeC5wPWdncGxvdCgpK2dlb21fcG9pbnQoZGF0YT1kdWdjdnIsYWVzKHg9bG9nKHRfcXNpemVfbTIpLCB5PXlpLGNvbG91cj1pZCksIyBzaXplPXdlaWdodHMoY3ZyLm1hLmZpeCkpLA0KICAgICAgICAgICAgICAgICAgICAgICAgICAgICAgICBhbHBoYT0wLjMpICtnZW9tX2xpbmUoYWVzKHg9Y3ZyLm1hLmZpeC5wcmVkcyRYLm1vZHMsIHk9Y3ZyLm1hLmZpeC5wcmVkcyRwcmVkKSkrZ2VvbV9saW5lKGFlcyh4PWN2ci5tYS5maXgucHJlZHMkWC5tb2RzLCB5PWN2ci5tYS5maXgucHJlZHMkcHJlZCkpK2dlb21fcmliYm9uKGFlcyh4PWN2ci5tYS5maXgucHJlZHMkWC5tb2RzLHltaW49Y3ZyLm1hLmZpeC5wcmVkcyRjaS5sYiwgeW1heD1jdnIubWEuZml4LnByZWRzJGNpLnViICksYWxwaGE9MC4yKStzY2FsZV9zaXplX2NvbnRpbnVvdXMoZ3VpZGU9RkFMU0UpK3NjYWxlX2NvbG9yX2Rpc2NyZXRlKGd1aWRlPUYpK3RoZW1lX2J3KCkreWxhYihleHByZXNzaW9uKGl0YWxpYyhjdnIpKSkreGxhYihOVUxMKStnZW9tX2hsaW5lKHlpbnRlcmNlcHQgPSAwLCBsaW5ldHlwZSA9ICJkYXNoZWQiKStnZ3RpdGxlKCIiKSt0aGVtZShwbG90LnRpdGxlID0gZWxlbWVudF90ZXh0KHNpemUgPSA4KSkrY29vcmRfY2FydGVzaWFuKHlsaW0gPSBjKC0xLjYsIDEuMikpIA0KDQoNCg0KY3ZyLm1hLnVuLnA9Z2dwbG90KCkrZ2VvbV9wb2ludChkYXRhPWR1Z2N2cixhZXMoeD1sb2codF9xc2l6ZV9tMiksIHk9eWksY29sb3VyPWlkLCBzaXplPTEpLCBhbHBoYT0wLjMpICtnZW9tX2xpbmUoYWVzKHg9Y3ZyLm1hLmZpeC5wcmVkcyRYLm1vZHMsIHk9Y3ZyLm1hLnVuLnByZWRzJHByZWQpKStnZW9tX2xpbmUoYWVzKHg9Y3ZyLm1hLnVuLnByZWRzJFgubW9kcywgeT1jdnIubWEudW4ucHJlZHMkcHJlZCkpK2dlb21fcmliYm9uKGFlcyh4PWN2ci5tYS51bi5wcmVkcyRYLm1vZHMseW1pbj1jdnIubWEudW4ucHJlZHMkY2kubGIsIHltYXg9Y3ZyLm1hLnVuLnByZWRzJGNpLnViICksYWxwaGE9MC4yKStzY2FsZV9jb2xvcl9kaXNjcmV0ZShndWlkZT1GKSt0aGVtZV9idygpK3lsYWIoZXhwcmVzc2lvbihpdGFsaWMoY3ZyKSkpK3hsYWIoIkxvZyhQbG90IHNpemUgKGhhKSkiKStnZW9tX2hsaW5lKHlpbnRlcmNlcHQgPSAwLCBsaW5ldHlwZSA9ICJkYXNoZWQiKStzY2FsZV9zaXplX2NvbnRpbnVvdXMoZ3VpZGU9RkFMU0UpK2dndGl0bGUoIiIpK3RoZW1lKHBsb3QudGl0bGUgPSBlbGVtZW50X3RleHQoc2l6ZSA9IDgpKSt5bGltKC0xLjYsIDEuMikNCg0KDQoNCg0KZ3JpZEV4dHJhOjpncmlkLmFycmFuZ2UoaGcubWEucmFuLnAsIGxyLm1hLnJhbi5wLGN2ci5tYS5yYW4ucCwNCiAgICAgICAgICAgICAgICAgICAgICAgIGhnLm1hLmZpeC5wLCBsci5tYS5maXgucCxjdnIubWEuZml4LnAsDQogICAgICAgICAgICAgICAgICAgICAgICBoZy5tYS51bi5wLCBsci5tYS51bi5wLGN2ci5tYS5maXgucCwNCiAgICAgICAgICAgICAgICAgICAgICAgIG5jb2w9MywgaGVpZ2h0cz1jKDIsMiwyLjIpKSMreWxpbSgtMy4yLCAwLjcpDQpgYGANCg0KDQpgYGB7ciwgZXZhbD1GQUxTRX0NCg0KIyBzdHVkaWVzIHdpdGggYWxsIHRocmVlIGNvbXBhcmlzb25zDQpyZV9yZWYgJT4lIGRyb3BfbmEoY19tZWFuLCByX21lYW4pICU+JSBkcGx5cjo6c2VsZWN0KGlkKSAlPiUgZGlzdGluY3QoKQ0KDQojIHRheG9uIGJyZWFrZG93bg0KZnVsbF9kYXRhICU+JSBncm91cF9ieSguJHRheG9uKSAlPiUgc3VtbWFyaXNlKG4oKSkNCg0KIyBtZXRyaWMgYnJlYWtkb3duDQpmdWxsX2RhdGEgJT4lIGdyb3VwX2J5KC4kbWVhc3VyZV90eXBlKSAlPiUgc3VtbWFyaXNlKG4oKSkNCg0KIyBtZXRyaWMgYnJlYWtkb3duDQpmdWxsX2RhdGEgJT4lIGdyb3VwX2J5KC4kcGx1KSAlPiUgc3VtbWFyaXNlKG4oKSkNCg0KIyBzaXplIGJyZWFrZG93bg0KZnVsbF9kYXRhICU+JSBkcm9wX25hKHNpdGVfc2l6ZSwgcl9tZWFuKSAlPiUgZHBseXI6OnN1bW1hcmlzZShuKCkpDQoNCiNmaW5hbCB0cmlwbGUgY2hlY2sgb2YgbW9kZWwgdmFsdWVzIGFnYWluc3QgaW4tdGV4dCB2YWx1ZXMNCnN1bW1hcnkobWVhbl91cikNCmV4cCguMTgxNSkNCnN1bW1hcnkoY3ZyX3VyKQ0KZXhwKC0wLjE1MTkpDQpzdW1tYXJ5KG1lYW5fcnIpDQpleHAoLTAuMTM5NSkNCnN1bW1hcnkoY3ZyX3JyKQ0KZXhwKDAuMTgzKQ0Kc3VtbWFyeShtZWFuX2FnZV91cikNCmV4cCgwLjAwNikNCg0Kc3VtbWFyeShjdnJfYWdlX3VyKQ0Kc3VtbWFyeShtZWFuX2FnZV9ycikNCnN1bW1hcnkoY3ZyX2FnZV9ycikNCnN1bW1hcnkobWVhbl9zaXplX3VyKQ0Kc3VtbWFyeShjdnJfc2l6ZV91cikNCnN1bW1hcnkobWVhbl9zaXplX3JyKQ0Kc3VtbWFyeShjdnJfc2l6ZV9ycikNCg0KIyByZXNvdHJhdGlvbiBtZXRob2QgYnJlYWtkb3duDQpmdWxsX2RhdGEgJT4lIHNlbGVjdChpZCwgcmVzdG9yYXRpb25fbWV0aG9kKSAlPiUgZGlzdGluY3QoKSAlPiUgIGdyb3VwX2J5KHJlc3RvcmF0aW9uX21ldGhvZCkgJT4lIHN1bW1hcmlzZShuKCkpDQoNCiMgbnVtYmVyIG9mIHN0dWRpZXMgZm9yIGVhY2ggc2VwYXJhdGUgTUENCmZ1bGxfZGF0YSAlPiUgZHJvcF9uYShjX21lYW4pICU+JSBkcGx5cjo6c2VsZWN0KGlkKSAlPiUgIGRpc3RpbmN0KCkgJT4lICBzdW1tYXJpc2UobigpKQ0KZnVsbF9kYXRhICU+JSBkcm9wX25hKHJfbWVhbikgJT4lIGRwbHlyOjpzZWxlY3QoaWQpICU+JSAgZGlzdGluY3QoKSAlPiUgIHN1bW1hcmlzZShuKCkpDQpmdWxsX2RhdGEgJT4lIGRyb3BfbmEocl9tZWFuLCBjX21lYW4pICU+JSBkcGx5cjo6c2VsZWN0KGlkKSAlPiUgIGRpc3RpbmN0KCkgJT4lICBzdW1tYXJpc2UobigpKQ0KZnVsbF9kYXRhICU+JSBzZWxlY3QoaWQpICU+JSAgZGlzdGluY3QoKSAlPiUgIHN1bW1hcmlzZShuKCkpDQpgYGANCg0KIyMjIExvYWRpbmcgZXh0cmEgcGFja2FnZXMgZm9yIHRheG9uIGFuYWx5c2lzDQoNCmBgYHtyfQ0KDQpsaWJyYXJ5KHB1cnJyKQ0KbGlicmFyeShtdWx0Y29tcCkNCmBgYA0KDQojIyMgQ3VzdG9tIGZ1bmN0aW9ucw0KDQoNCmBgYHtyfQ0KDQoNCmdldF9wcmVkMSA8LSBmdW5jdGlvbihtb2RlbCwgbW9kID0gIiAiKSB7DQogIG5hbWUgPC0gbmFtZSA8LSBmaXJzdHVwKGFzLmNoYXJhY3RlcihzdHJpbmdyOjpzdHJfcmVwbGFjZShyb3cubmFtZXMobW9kZWwkYmV0YSksIA0KICAgICAgICAgICAgICAgICAgICAgICAgICAgICAgICAgICAgICAgICAgICAgICAgICAgICAgICAgICAgbW9kLCAiIikpKQ0KICBsZW4gPC0gbGVuZ3RoKG5hbWUpDQogIA0KICBpZiAobGVuICE9IDEpIHsNCiAgICBuZXdkYXRhIDwtIG1hdHJpeChOQSwgbmNvbCA9IGxlbiwgbnJvdyA9IGxlbikNCiAgICBmb3IgKGkgaW4gMTpsZW4pIHsNCiAgICAgIHBvcyA8LSB3aGljaChtb2RlbCRYWywgaV0gPT0gMSlbWzFdXQ0KICAgICAgbmV3ZGF0YVssIGldIDwtIG1vZGVsJFhbcG9zLCBdDQogICAgfQ0KICAgIHByZWQgPC0gbWV0YWZvcjo6cHJlZGljdC5ybWEobW9kZWwsIG5ld21vZHMgPSBuZXdkYXRhKQ0KICB9IGVsc2Ugew0KICAgIHByZWQgPC0gbWV0YWZvcjo6cHJlZGljdC5ybWEobW9kZWwpDQogIH0NCiAgZXN0aW1hdGUgPC0gcHJlZCRwcmVkDQogIGxvd2VyQ0wgPC0gcHJlZCRjaS5sYg0KICB1cHBlckNMIDwtIHByZWQkY2kudWINCiAgbG93ZXJQUiA8LSBwcmVkJGNyLmxiDQogIHVwcGVyUFIgPC0gcHJlZCRjci51Yg0KICANCiAgdGFibGUgPC0gdGliYmxlKG5hbWUgPSBmYWN0b3IobmFtZSwgbGV2ZWxzID0gbmFtZSwgbGFiZWxzID0gbmFtZSksIGVzdGltYXRlID0gZXN0aW1hdGUsIA0KICAgICAgICAgICAgICAgICAgbG93ZXJDTCA9IGxvd2VyQ0wsIHVwcGVyQ0wgPSB1cHBlckNMLCBwdmFsID0gbW9kZWwkcHZhbCwgbG93ZXJQUiA9IGxvd2VyUFIsIA0KICAgICAgICAgICAgICAgICAgdXBwZXJQUiA9IHVwcGVyUFIpDQp9DQoNCmdldF9wcmVkMiA8LSBmdW5jdGlvbihtb2RlbCwgbW9kID0gIiAiKSB7DQogIG5hbWUgPC0gYXMuZmFjdG9yKHN0cl9yZXBsYWNlKHJvdy5uYW1lcyhtb2RlbCRiZXRhKSwgcGFzdGUwKCJyZWxldmVsIiwgIlxcKCIsIA0KICAgICAgICAgICAgICAgICAgICAgICAgICAgICAgICAgICAgICAgICAgICAgICAgICAgICAgICAgICAgICBtb2QsICIsIHJlZiA9IG5hbWUiLCAiXFwpIiksICIiKSkNCiAgbGVuIDwtIGxlbmd0aChuYW1lKQ0KICANCiAgaWYgKGxlbiAhPSAxKSB7DQogICAgbmV3ZGF0YSA8LSBkaWFnKGxlbikNCiAgICBwcmVkIDwtIHByZWRpY3Qucm1hKG1vZGVsLCBpbnRlcmNlcHQgPSBGQUxTRSwgbmV3bW9kcyA9IG5ld2RhdGFbLCAtMV0pDQogIH0gZWxzZSB7DQogICAgcHJlZCA8LSBwcmVkaWN0LnJtYShtb2RlbCkNCiAgfQ0KICBlc3RpbWF0ZSA8LSBwcmVkJHByZWQNCiAgbG93ZXJDTCA8LSBwcmVkJGNpLmxiDQogIHVwcGVyQ0wgPC0gcHJlZCRjaS51Yg0KICBsb3dlclBSIDwtIHByZWQkY3IubGINCiAgdXBwZXJQUiA8LSBwcmVkJGNyLnViDQogIA0KICB0YWJsZSA8LSB0aWJibGUobmFtZSA9IGZhY3RvcihuYW1lLCBsZXZlbHMgPSBuYW1lLCBsYWJlbHMgPSBuYW1lKSwgZXN0aW1hdGUgPSBlc3RpbWF0ZSwgDQogICAgICAgICAgICAgICAgICBsb3dlckNMID0gbG93ZXJDTCwgdXBwZXJDTCA9IHVwcGVyQ0wsIHB2YWwgPSBtb2RlbCRwdmFsLCBsb3dlclBSID0gbG93ZXJQUiwgDQogICAgICAgICAgICAgICAgICB1cHBlclBSID0gdXBwZXJQUikNCn0NCg0KDQp1bmlfbW9kX3Bsb3Q8LWZ1bmN0aW9uKG0sIGRmLCBsb2dfcmF0aW8sIHJlc3BvbnNlLCB2YXJpYW5jZSl7DQpwIDwtIHByZWRpY3Qucm1hKG0pDQpkZiAlPiUgbXV0YXRlKHltaW4gPSBwJGNpLmxiLCANCiAgICAgICAgICAgICAgICAgICAgICAgICAgICAgICAgICAgICAgICAgICAgICAgICAgeW1heCA9IHAkY2kudWIsIHltaW4yID0gcCRjci5sYiwgDQogICAgICAgICAgICAgICAgICAgICAgICAgICAgICAgICAgICAgICAgICAgICAgICAgIHltYXgyID0gcCRjci51YiwgcHJlZCA9IHAkcHJlZCkgJT4lIA0KICBnZ3Bsb3QoYWVzKHggPSByZXNwb25zZSwgeSA9IGxvZ19yYXRpbywgc2l6ZSA9IHNxcnQoMS92YXJpYW5jZSkpKSArIGdlb21fcG9pbnQoc2hhcGUgPSAyMSwgYWxwaGE9IDAuMiwNCiAgICAgICAgICAgICAgICAgICAgICAgICAgICAgICAgICAgICAgICAgICAgICAgICAgICAgICAgICAgICAgICAgICAgIGZpbGwgPSAiZ3JleTkwIikgKyANCiAgZ2VvbV9obGluZSh5aW50ZXJjZXB0ID0gMCwgc2l6ZSA9IC41LCBjb2xvdXIgPSAiZ3JheTcwIikrDQogIGdlb21fc21vb3RoKGFlcyh5ID0geW1pbjIpLCBtZXRob2QgPSAibG0iLCBzZSA9IEZBTFNFLCBsdHkgPSAic29saWQiLCBsd2QgPSAwLjc1LCANCiAgICAgICAgICAgICAgY29sb3VyID0gIiMwMDcyQjIiKSArIGdlb21fc21vb3RoKGFlcyh5ID0geW1heDIpLCBtZXRob2QgPSAibG0iLCBzZSA9IEZBTFNFLCANCiAgICAgICAgICAgICAgICAgICAgICAgICAgICAgICAgICAgICAgICAgICAgICAgIGx0eSA9ICJzb2xpZCIsIGx3ZCA9IDAuNzUsIGNvbG91ciA9ICIjMDA3MkIyIikgKyBnZW9tX3Ntb290aChhZXMoeSA9IHltaW4pLCANCiAgICAgICAgICAgICAgICAgICAgICAgICAgICAgICAgICAgICAgICAgICAgICAgICAgICAgICAgICAgICAgICAgICAgICAgICAgICAgICAgICAgICAgICAgICAgICAgICAgICAgICAgICAgICAgbWV0aG9kID0gImxtIiwgc2UgPSBGQUxTRSwgbHR5ID0gImRhc2hlZCIsIGx3ZCA9IDAuNzUsIGNvbG91ciA9ICIjRDU1RTAwIikgKyANCiAgZ2VvbV9zbW9vdGgoYWVzKHkgPSB5bWF4KSwgbWV0aG9kID0gImxtIiwgc2UgPSBGQUxTRSwgbHR5ID0gInNvbGlkIiwgbHdkID0gMC43NSwgDQogICAgICAgICAgICAgIGNvbG91ciA9ICIjRDU1RTAwIikgKyBnZW9tX3Ntb290aChhZXMoeSA9IHByZWQpLCBtZXRob2QgPSAibG0iLCBzZSA9IEZBTFNFLCANCiAgICAgICAgICAgICAgICAgICAgICAgICAgICAgICAgICAgICAgICAgICAgICAgIGx0eSA9ICJzb2xpZCIsIGx3ZCA9IDEsIGNvbG91ciA9ICJibGFjayIpICsgDQogIGxhYnMoeCA9ICJcbiBsbihyZXN0b3JhdGlvbiBzaXRlIGFnZSkiLCB5ID0gImxuKHJlc3RvcmVkL3VucmVzdG9yZWQpIC0gbWVhbiBiaW9kaXZlcnNpdHkiLCBzaXplID0gIlByZWNpc2lvbiAoMS9TRSkiKSArIGd1aWRlcyhmaWxsID0gIm5vbmUiLCANCiAgICAgICAgICAgICAgICAgICAgICAgICAgICAgICAgICAgICAgICAgICAgICAgICAgICAgICAgICAgICAgICAgICAgICAgICAgICAgICAgICAgICAgICAgICAgICAgICAgICAgICAgICAgICAgICAgIGNvbG91ciA9ICJub25lIikgKyAjIHRoZW1zZXMNCiAgdGhlbWVfY2xhc3NpYygpICsgdGhlbWUobGVnZW5kLnBvc2l0aW9uID0gYygwLCAxKSwgbGVnZW5kLmp1c3RpZmljYXRpb24gPSBjKDAsIDEpKSArIHRoZW1lKGxlZ2VuZC5kaXJlY3Rpb24gPSAiaG9yaXpvbnRhbCIpICsgDQogIHRoZW1lKGxlZ2VuZC5iYWNrZ3JvdW5kID0gZWxlbWVudF9ibGFuaygpKSArIHRoZW1lKGF4aXMudGV4dC55ID0gZWxlbWVudF90ZXh0KHNpemUgPSA4LCANCiAgICAgICAgICAgICAgICAgICAgICAgICAgICAgICAgICAgICAgICAgICAgICAgICAgICAgICAgICAgICAgICAgICAgICAgICAgICAgICAgY29sb3VyID0gImJsYWNrIiwgaGp1c3QgPSAwLjUsIGFuZ2xlID0gOTApKSsNCiAgY29vcmRfY2FydGVzaWFuKHlsaW0gPSBjKC0yLjUsIDIuNSkpKw0KICBzY2FsZV95X2NvbnRpbnVvdXMobGltaXRzID0gYygtMi41LCAyLjUpLA0KICAgICAgICAgICAgICAgICAgICAgYnJlYWtzID0gYygtMiwgLTEsIDAsIDEsIDIpLA0KICAgICAgICAgICAgICAgICAgICAgbGFiZWxzID0gYygiXG4gXG4gLTIiLCAiMTAwJSBkZWNyZWFzZSBcbiBcbiAtMSIsICJcbiBcbiAwLjAiLCAiMTAwJSBpbmNyZWFzZSBcbiBcbiAxIiwgIlxuIFxuMiIpKSArDQogIHRoZW1lKGxlZ2VuZC5wb3NpdGlvbiA9ICJub25lIikgDQp9DQoNCg0KdW5pX21vZF9wbG90X25zPC1mdW5jdGlvbihtLCBkZiwgbG9nX3JhdGlvLCByZXNwb25zZSwgdmFyaWFuY2Upew0KcCA8LSBwcmVkaWN0LnJtYShtKQ0KZGYgJT4lIG11dGF0ZSh5bWluID0gcCRjaS5sYiwgDQogICAgICAgICAgICAgICAgICAgICAgICAgICAgICAgICAgICAgICAgICAgICAgICAgIHltYXggPSBwJGNpLnViLCB5bWluMiA9IHAkY3IubGIsIA0KICAgICAgICAgICAgICAgICAgICAgICAgICAgICAgICAgICAgICAgICAgICAgICAgICB5bWF4MiA9IHAkY3IudWIsIHByZWQgPSBwJHByZWQpICU+JSANCiAgZ2dwbG90KGFlcyh4ID0gcmVzcG9uc2UsIHkgPSBsb2dfcmF0aW8sIHNpemUgPSBzcXJ0KDEvdmFyaWFuY2UpKSkgKyBnZW9tX3BvaW50KHNoYXBlID0gMjEsIGFscGhhPSAwLjIsDQogICAgICAgICAgICAgICAgICAgICAgICAgICAgICAgICAgICAgICAgICAgICAgICAgICAgICAgICAgICAgICAgICAgICBmaWxsID0gImdyZXk5MCIpICsgDQogIGdlb21faGxpbmUoeWludGVyY2VwdCA9IDAsIHNpemUgPSAuNSwgY29sb3VyID0gImdyYXk3MCIpKw0KICBnZW9tX3Ntb290aChhZXMoeSA9IHltaW4yKSwgbWV0aG9kID0gImxtIiwgc2UgPSBGQUxTRSwgbHR5ID0gImRhc2hlZCIsIGx3ZCA9IDAuNzUsIA0KICAgICAgICAgICAgICBjb2xvdXIgPSAiIzAwNzJCMiIpICsgZ2VvbV9zbW9vdGgoYWVzKHkgPSB5bWF4MiksIG1ldGhvZCA9ICJsbSIsIHNlID0gRkFMU0UsIA0KICAgICAgICAgICAgICAgICAgICAgICAgICAgICAgICAgICAgICAgICAgICAgICAgbHR5ID0gImRhc2hlZCIsIGx3ZCA9IDAuNzUsIGNvbG91ciA9ICIjMDA3MkIyIikgKyBnZW9tX3Ntb290aChhZXMoeSA9IHltaW4pLCANCiAgICAgICAgICAgICAgICAgICAgICAgICAgICAgICAgICAgICAgICAgICAgICAgICAgICAgICAgICAgICAgICAgICAgICAgICAgICAgICAgICAgICAgICAgICAgICAgICAgICAgICAgICAgICAgbWV0aG9kID0gImxtIiwgc2UgPSBGQUxTRSwgbHR5ID0gImRhc2hlZCIsIGx3ZCA9IDAuNzUsIGNvbG91ciA9ICIjRDU1RTAwIikgKyANCiAgZ2VvbV9zbW9vdGgoYWVzKHkgPSB5bWF4KSwgbWV0aG9kID0gImxtIiwgc2UgPSBGQUxTRSwgbHR5ID0gImRhc2hlZCIsIGx3ZCA9IDAuNzUsIA0KICAgICAgICAgICAgICBjb2xvdXIgPSAiI0Q1NUUwMCIpICsgZ2VvbV9zbW9vdGgoYWVzKHkgPSBwcmVkKSwgbWV0aG9kID0gImxtIiwgc2UgPSBGQUxTRSwgDQogICAgICAgICAgICAgICAgICAgICAgICAgICAgICAgICAgICAgICAgICAgICAgICBsdHkgPSAiZGFzaGVkIiwgbHdkID0gMSwgY29sb3VyID0gImJsYWNrIikgKyANCiAgbGFicyh4ID0gIlxuIGxuKHJlc3RvcmF0aW9uIHNpdGUgYWdlKSIsIHkgPSAibG4ocmVzdG9yZWQvdW5yZXN0b3JlZCkgLSBtZWFuIGJpb2RpdmVyc2l0eSIsIHNpemUgPSAiUHJlY2lzaW9uICgxL1NFKSIpICsgZ3VpZGVzKGZpbGwgPSAibm9uZSIsIA0KICAgICAgICAgICAgICAgICAgICAgICAgICAgICAgICAgICAgICAgICAgICAgICAgICAgICAgICAgICAgICAgICAgICAgICAgICAgICAgICAgICAgICAgICAgICAgICAgICAgICAgICAgICAgICAgICAgY29sb3VyID0gIm5vbmUiKSArICMgdGhlbXNlcw0KICB0aGVtZV9jbGFzc2ljKCkgKyB0aGVtZShsZWdlbmQucG9zaXRpb24gPSBjKDAsIDEpLCBsZWdlbmQuanVzdGlmaWNhdGlvbiA9IGMoMCwgMSkpICsgdGhlbWUobGVnZW5kLmRpcmVjdGlvbiA9ICJob3Jpem9udGFsIikgKyANCiAgdGhlbWUobGVnZW5kLmJhY2tncm91bmQgPSBlbGVtZW50X2JsYW5rKCkpICsgdGhlbWUoYXhpcy50ZXh0LnkgPSBlbGVtZW50X3RleHQoc2l6ZSA9IDgsIA0KICAgICAgICAgICAgICAgICAgICAgICAgICAgICAgICAgICAgICAgICAgICAgICAgICAgICAgICAgICAgICAgICAgICAgICAgICAgICAgICBjb2xvdXIgPSAiYmxhY2siLCBoanVzdCA9IDAuNSwgYW5nbGUgPSA5MCkpKw0KICBjb29yZF9jYXJ0ZXNpYW4oeWxpbSA9IGMoLTIuNSwgMi41KSkrDQogIHNjYWxlX3lfY29udGludW91cyhsaW1pdHMgPSBjKC0yLjUsIDIuNSksDQogICAgICAgICAgICAgICAgICAgICBicmVha3MgPSBjKC0yLCAtMSwgMCwgMSwgMiksDQogICAgICAgICAgICAgICAgICAgICBsYWJlbHMgPSBjKCJcbiBcbiAtMiIsICIxMDAlIGRlY3JlYXNlIFxuIFxuIC0xIiwgIlxuIFxuIDAuMCIsICIxMDAlIGluY3JlYXNlIFxuIFxuIDEiLCAiXG4gXG4yIikpICsNCiAgdGhlbWUobGVnZW5kLnBvc2l0aW9uID0gIm5vbmUiKSANCn0NCg0KDQpgYGANCg0KYGBge3J9DQoNCg0KZGF0PC1yZWFkLmNzdigiRGF0YS92YXJpYXRpb25fZGF0YS5jc3YiKQ0KDQoNCmBgYA0KDQojIyBWZWdldGF0aW9uIHR5cGVzDQoNCg0KQmVsb3cgYXJlIHRocmVlIHRhYmxlcyB0aGF0IHN1bW1hcmlzZSB2YXJpb3VzIHN1Ymdyb3VwcyB3aXRoaW4gdGhlIGRhdGEgaW5jbHVkaW5nOiB0aGUgYnJlYWtkb3duIG9mIGJyb2FkIHZlZ2V0YXRpb24gdHlwZXMsIGNhdGVnb3Jpc2VkIGludG8gd29vZHkvbm9uLXdvb2R5LiBUaGVzZSBhcmUgdmVyeSBjb2Fyc2UgY2F0ZWdvcmllcyBhbmQgIndvb2R5IiBlbmNvbXBhc3NlcyBhIGxhcmdlIHJhbmdlIG9mIHZlZ2V0YXRpb24gdHlwZXMgZnJvbSB3b29kbGFuZCB0byBzaHJ1YmxhbmQgdG8gcmFpbmZvcmVzdC4gTm9uLXdvb2R5IGlzIGEgY2F0Y2gtYWxsIGZvciBoZXJiYWNlb3VzIHZlZ2V0YXRpb24gY29tbXVuaXRpZXMsIGUuZy4gcHJhaXJpZSwgZm9yYi0gb3IgaGVyYi1kb21pbmF0ZWQgZWNvc3lzdGVtcy4gDQoNCg0KKipUYWJsZSBTMTEqKiBOdW1iZXIgb2YgZWZmZWN0IHNpemVzIGluY2x1ZGVkIGluIHRoZSBtZXRhLWFuYWx5c2lzIGJ5IGRvbWluYW50IHZlZ2V0YXRpb24NCg0KYGBge3J9DQoNCmRmPC1kYXQNCmRmICU+JSBncm91cF9ieSh3b29keV9ub253b29keSkgJT4lIHN1bW1hcmlzZShuKCkpICU+JSByZW5hbWUoYERvbWluYW50IHZlZ2V0YXRpb24gdHlwZWAgPSB3b29keV9ub253b29keSwgYE51bWJlciBvZiBlZmZlY3Qgc2l6ZXNgID0gYG4oKWApICU+JSBrYWJsZSgiaHRtbCIpICAlPiUgDQogIGthYmxlX3N0eWxpbmcoInN0cmlwZWQiLCBwb3NpdGlvbiA9ICJsZWZ0IikNCg0KYGBgDQoNCg0KaGlsZSBicm9hZCB0YXhvbm9taWMgZ3JvdXBzIHdlcmUgdXNlZCBmb3Igc3ViZ3JvdXAgYW5hbHlzZXMgaW4gdGhlIG1haW4tdGV4dCwgd2UgaW5jbHVkZSBoZXJlIHRoZSBtb3JlIGRldGFpbGVkIG5vdGVzIG9uIHRheG9uIGNvbGxlY3RlZCBkdXJpbmcgdGhlIGxpdGVyYXR1cmUgc2VhcmNoIHdoaWNoIG1heSBiZSBvZiBhZGRpdGlvbmFsIGludGVyZXN0Lg0KDQoNCioqVGFibGUgUzEyKiogTnVtYmVyIG9mIGVmZmVjdCBzaXplcyBpbmNsdWRlZCBpbiB0aGUgbWV0YS1hbmFseXNpcyBieSB0YXhvbg0KYGBge3J9DQoNCmRmICU+JSBncm91cF9ieSh0YXhvbiwgdGF4b25fZGV0YWlsKSAlPiUgc3VtbWFyaXNlKG4oKSkgJT4lIHJlbmFtZShUYXhvbiA9IHRheG9uLCBUYXhvbl9kZXRhaWwgPSB0YXhvbl9kZXRhaWwsIGBOdW1iZXIgb2YgZWZmZWN0IHNpemVzYCA9IGBuKClgKSAlPiUga2FibGUoImh0bWwiKSAgJT4lIA0KICBrYWJsZV9zdHlsaW5nKCJzdHJpcGVkIiwgcG9zaXRpb24gPSAibGVmdCIpICU+JQ0KICAgIHNjcm9sbF9ib3god2lkdGggPSAiODAwcHgiLCBoZWlnaHQgPSAiMzAwcHgiKQ0KDQpgYGANCg0KDQpXaGlsZSB3ZSBjb25kdWN0ZWQgYWxsIGFuYWx5c2VzIHRocm91Z2ggdGhlIGxlbnMgb2YgbWVhbiAiYmlvZGl2ZXJzaXR5IiBhbmQgdmFyaWFiaWxpdHkgb2YgImJpb2RpdmVyc2l0eSIsIGJpb2RpdmVyc2l0eSBjYW4gYmUgbWVhc3VyZWQgaW4gbWFueSBkaWZmZXJlbnQgd2F5cy4gQmVsb3cgd2UgZGV0YWlsIHRoZSB2YXJpZXR5IG9mIG1lYXN1cmVzIG9mIGJpb2RpdmVyc2l0eSBpbmNsdWRlZCBpbiB0aGUgbWV0YS1hbmFseXNpcy4NCg0KDQoqKlRhYmxlIFMxMyoqIE51bWJlciBvZiBlZmZlY3Qgc2l6ZXMgaW5jbHVkZWQgaW4gdGhlIG1ldGEtYW5hbHlzaXMgYnkgbWVhc3VyZSBvZiBiaW9kaXZlcnNpdHkNCmBgYHtyfQ0KDQpkZiAlPiUgZ3JvdXBfYnkobWVhc3VyZV90eXBlLCBtZWFzdXJlKSAlPiUgc3VtbWFyaXNlKG4oKSkgJT4lIHJlbmFtZShNZWFzdXJlID0gbWVhc3VyZSwgTWVhc3VyZV9kZXRhaWwgPSBtZWFzdXJlX3R5cGUsIGBOdW1iZXIgb2YgZWZmZWN0IHNpemVzYCA9IGBuKClgKSAlPiUga2FibGUoImh0bWwiKSAgJT4lIA0KICBrYWJsZV9zdHlsaW5nKCJzdHJpcGVkIiwgcG9zaXRpb24gPSAibGVmdCIpJT4lDQogICAgc2Nyb2xsX2JveCh3aWR0aCA9ICI4MDBweCIsIGhlaWdodCA9ICIzMDBweCIpDQoNCmBgYA0KKipUYWJsZSBTMTQqKiBOdW1iZXIgb2YgZWZmZWN0IHNpemVzIGluY2x1ZGVkIGluIHRoZSBtZXRhLWFuYWx5c2lzIGJ5IG1lYXN1cmUgb2YgYmlvZGl2ZXJzaXR5DQpgYGB7cn0NCg0KZGYgJT4lIGdyb3VwX2J5KHRheG9uLCBtZWFzdXJlKSAlPiUgc3VtbWFyaXNlKG4oKSkgJT4lIHJlbmFtZShNZWFzdXJlID0gbWVhc3VyZSwgVGF4b24gPSB0YXhvbiwgYE51bWJlciBvZiBlZmZlY3Qgc2l6ZXNgID0gYG4oKWApICU+JSBrYWJsZSgiaHRtbCIpICAlPiUgDQogIGthYmxlX3N0eWxpbmcoInN0cmlwZWQiLCBwb3NpdGlvbiA9ICJsZWZ0IiklPiUNCiAgICBzY3JvbGxfYm94KHdpZHRoID0gIjgwMHB4IiwgaGVpZ2h0ID0gIjMwMHB4IikNCg0KYGBgDQoNCg0KYGBge3J9DQoNCiMjIyMjIyMjIyMjIyMjIyMjIyMjIyMjIyMjIw0KIyMgVU5SRVNUT1JFRCAvIFJFU1RPUkVEICMjDQojIyMjIyMjIyMjIyMjIyMjIyMjIyMjIyMjIyMNCg0KIyB1bl9yZSA9IHVucmVzdG9yZWQgLyByZXN0b3JlZCANCg0KdW5fcmU8LXJlYWQuY3N2KCJEYXRhL3ZhcmlhdGlvbl9kYXRhLmNzdiIsIHN0cmluZ3NBc0ZhY3RvcnMgPSBGKQ0KDQp1bl9yZSRjX3F1YWRfbiA9IGFzLm51bWVyaWModW5fcmUkY19xdWFkX24pDQp1bl9yZSRjX21lYW4gPSAgYXMubnVtZXJpYyh1bl9yZSRjX21lYW4pDQp1bl9yZSRjX3NkID0gYXMubnVtZXJpYyh1bl9yZSRjX3NkKQ0KDQojcmVtb3ZlIHN0dWRpZXMgd2l0aCBvbmx5IGEgcmVzdG9yZWQgc2l0ZXMgY29tcGFyaXNvbg0KdW5fcmU8LXVuX3JlWyFpcy5uYSh1bl9yZSRjX21lYW4pLF0NCiN1bl9yZSAlPiUgZ3JvdXBfYnkoaWQsIGNfbWVhbiwgY19zZCkgJT4lIGRpc3RpbmN0KHNoYXJlZF9jdHJsKSAlPiUgZmlsdGVyKG4oKT4xKSAjIGNoZWNraW5nIHNoYXJlZCBjb250cm9scyBpcyBhY2N1cmF0ZQ0KDQojY2FsY3VsYXRlIHRoZSBsbkNWUiBhbmQgbG5SUiBhbmQgbG5WUiBlZmZlY3Qgc2l6ZSBhbmQgdW5fcmVpYW5jZSB3aXRoIGVzY2FsYw0KQ1ZSPC1lc2NhbGMobWVhc3VyZSA9ICJDVlIiLCBuMWkgPSB1bl9yZSR0X3F1YWRfbiwgbjJpID0gdW5fcmUkY19xdWFkX24sIG0xaSA9IHVuX3JlJHRfbWVhbiwgbTJpID0gdW5fcmUkY19tZWFuLCBzZDFpID0gdW5fcmUkdF9zZCwgc2QyaSA9IHVuX3JlJGNfc2QpDQpsblJSPC1lc2NhbGMobWVhc3VyZSA9ICJST00iLCBuMWkgPSB1bl9yZSR0X3F1YWRfbiwgbjJpID0gdW5fcmUkY19xdWFkX24sIG0xaSA9IHVuX3JlJHRfbWVhbiwgbTJpID0gdW5fcmUkY19tZWFuLCBzZDFpID0gdW5fcmUkdF9zZCwgc2QyaSA9IHVuX3JlJGNfc2QpDQpsblZSPC1lc2NhbGMobWVhc3VyZSA9ICJWUiIsIG4xaSA9IHVuX3JlJHRfcXVhZF9uLCBuMmkgPSB1bl9yZSRjX3F1YWRfbiwgbTFpID0gdW5fcmUkdF9tZWFuLCBtMmkgPSB1bl9yZSRjX21lYW4sIHNkMWkgPSB1bl9yZSR0X3NkLCBzZDJpID0gdW5fcmUkY19zZCkNCg0KI2NvbWJpbmVkIGVmZmVjdCBzaXplcyB3aXRoIHJlbGV2YW50IHVuX3JlYSBmcmFtZXMNCnVuX3JlIDwtYmluZF9jb2xzKHVuX3JlLCBsblJSLCBsblZSLCBDVlIpDQoNCiMgbmFtZSB0aGUgdW5fcmVhIHNvbWV0aGluZyBtZWFuaW5nZnVsIGFuZCByZW1vdmUgYWxsIHRoZSBjb2x1bW5zIHVubmVlZGVkDQp1bl9yZTwtdW5fcmUgJT4lIHJlbmFtZSh5aV9tZWFuID0geWkuLi4zNiwgdmlfbWVhbiA9IHZpLi4uMzcsIHlpX3ZyID0geWkuLi4zOCwgdmlfdnIgPSB2aS4uLjM5LCB5aV9jdnIgPSB5aS4uLjQwLCB2aV9jdnIgPSB2aS4uLjQxKQ0KDQojcmVtb3ZlIHN0dWRpZXMgdGhhdCBoYXZlIHZpPU5BIC0gdXN1YWxseSB3aGVyZSBjb250cm9sIFNEID0gMA0KdW5fcmU8LXVuX3JlWyFpcy5uYSh1bl9yZSR2aV92ciksXQ0KDQp1bl9yZSRwbHU8LWFzLmZhY3Rvcih1bl9yZSRwbHUpDQp1bl9yZSRwbHU8LXJlbGV2ZWwodW5fcmUkcGx1LCAic2VtaS1uYXR1cmFsIikNCg0KI25lZWQgYW5vdGhlciByYW5kb20gZmFjdG9yIGZvciAndW5pdCcNCg0KdW5pdCA8LSBmYWN0b3IoMTpsZW5ndGgodW5fcmUkeWlfbWVhbikpDQp1bl9yZSR1bml0IDwtIHVuaXQNCg0KdmN2X2N2cjwtbWFrZV9WQ1ZfbWF0cml4KHVuX3JlLCBWID0idmlfY3ZyIiwgInNoYXJlZF9jdHJsIiwgInVuaXQiLCByaG89MC41KQ0KdmN2X21lYW48LW1ha2VfVkNWX21hdHJpeCh1bl9yZSwgViA9InZpX21lYW4iLCAic2hhcmVkX2N0cmwiLCAidW5pdCIsIHJobz0wLjUpDQp2Y3ZfdnI8LW1ha2VfVkNWX21hdHJpeCh1bl9yZSwgViA9InZpX3ZyIiwgInNoYXJlZF9jdHJsIiwgInVuaXQiLCByaG89MC41KQ0KDQoNCg0KIyMjIyMjIyMjIyMjIyMjIyMjIyMjIyMjIyMNCiMjIFJFU1RPUkVEIC8gUkVGRVJFTkNFICMjDQojIyMjIyMjIyMjIyMjIyMjIyMjIyMjIyMjIw0KDQojIHJlX3JlZiA9IHJlc3RvcmVkIC8gcmVmZXJlbmNlIA0KDQpyZV9yZWY8LXJlYWQuY3N2KCJEYXRhL3ZhcmlhdGlvbl9kYXRhLmNzdiIsIHN0cmluZ3NBc0ZhY3RvcnMgPSBGKQ0KDQojcmVtb3ZlIHN0dWRpZXMgd2l0aCBvbmx5IGEgZGVncmFkZWQgc2l0ZSBjb21wYXJpc29uDQpyZV9yZWY8LXJlX3JlZlshaXMubmEocmVfcmVmJHJfbWVhbiksXQ0KDQojcmVtb3ZlIHN0dWRpZXMgdGhhdCBoYXZlIHZpPU5BIC0gdXN1YWxseSB3aGVyZSBjb250cm9sIFNEID0gMA0KcmVfcmVmPC1yZV9yZWYgJT4lIGZpbHRlcihyX3NkICE9IDApDQpyZV9yZWY8LXJlX3JlZiAlPiUgZmlsdGVyKCFpcy5uYShyX3NkKSkNCg0KIyB0aGVyZSBpcyBhIGZldyBzaXRlcyB3aGVyZSB0aGUgcmVmZXJlbmNlIGNvbnRyb2wgaXMgc2hhcmVkLCBidXQgdGhlIGRlZ3JhZGVkIG9uZSBpcyBub3QsIG5lZWQgdG8gYWRkIGEgInJlZl9zaGFyZWRfY3RybCIgdG8gY29ycmVjdCB0aGlzDQpyZV9yZWY8LXJlX3JlZiAlPiUgZ3JvdXBfYnkoaWQsIHJfbWVhbiwgcl9zZCkgJT4lIG11dGF0ZShyZWZfc2hhcmVkX2N0cmwgPSBjdXJfZ3JvdXBfaWQoKSkNCiNyZV9yZWYgJT4lIGdyb3VwX2J5KGlkLCByX21lYW4sIHJfc2QpICU+JSBkaXN0aW5jdChyZWZfc2hhcmVkX2N0cmwpICU+JSBmaWx0ZXIobigpPjEpICMgdG8gY2hlY2sgYW55IGVycm9ycyBpbiB0aGUgc2hhcmVkX2NvbnRyb2wgdGFnZ2luZw0KDQpyZV9yZWYkcl9xdWFkX24gPSBhcy5udW1lcmljKHJlX3JlZiRyX3F1YWRfbikNCnJlX3JlZiRyX21lYW4gPSAgYXMubnVtZXJpYyhyZV9yZWYkcl9tZWFuKQ0KcmVfcmVmJHJfc2QgPSBhcy5udW1lcmljKHJlX3JlZiRyX3NkKQ0KDQpyZV9yZWY8LXJlX3JlZiAlPiUgZmlsdGVyKHJfcXVhZF9uID4gMSkgIyBhIGZldyBzYW1wbGUgc2l6ZXMgb2YgMSBvciAwPw0KDQoNCiNjYWxjdWxhdGUgdGhlIGxuQ1ZSIGFuZCBsblJSIGVmZmVjdCBzaXplIGFuZCByZV9yZWZpYW5jZSB3aXRoIGVzY2FsYw0KQ1ZSPC1lc2NhbGMobWVhc3VyZSA9ICJDVlIiLCBuMWkgPSByZV9yZWYkdF9xdWFkX24sIG4yaSA9IHJlX3JlZiRyX3F1YWRfbiwgbTFpID0gcmVfcmVmJHRfbWVhbiwgbTJpID0gcmVfcmVmJHJfbWVhbiwgc2QxaSA9IHJlX3JlZiR0X3NkLCBzZDJpID0gcmVfcmVmJHJfc2QpDQpsblJSPC1lc2NhbGMobWVhc3VyZSA9ICJST00iLCBuMWkgPSByZV9yZWYkdF9xdWFkX24sIG4yaSA9IHJlX3JlZiRyX3F1YWRfbiwgbTFpID0gcmVfcmVmJHRfbWVhbiwgbTJpID0gcmVfcmVmJHJfbWVhbiwgc2QxaSA9IHJlX3JlZiR0X3NkLCBzZDJpID0gcmVfcmVmJHJfc2QpDQpsblZSPC1lc2NhbGMobWVhc3VyZSA9ICJWUiIsIG4xaSA9IHJlX3JlZiR0X3F1YWRfbiwgbjJpID0gcmVfcmVmJHJfcXVhZF9uLCBtMWkgPSByZV9yZWYkdF9tZWFuLCBtMmkgPSByZV9yZWYkcl9tZWFuLCBzZDFpID0gcmVfcmVmJHRfc2QsIHNkMmkgPSByZV9yZWYkcl9zZCkNCg0KDQojY29tYmluZWQgZWZmZWN0IHNpemVzIHdpdGggcmVsZXZhbnQgZGF0YSBmcmFtZXMNCnJlX3JlZiA8LWJpbmRfY29scyhyZV9yZWYsIGxuUlIsIGxuVlIsIENWUikNCiMgbmFtZSB0aGUgZGF0YSBzb21ldGhpbmcgbWVhbmluZ2Z1bCBhbmQgcmVtb3ZlIGFsbCB0aGUgY29sdW1ucyB1bm5lZWRlZA0KcmVfcmVmPC1yZV9yZWYgJT4lIHJlbmFtZSh5aV9tZWFuID0geWkuLi4zNywgdmlfbWVhbiA9IHZpLi4uMzgsIHlpX3ZyID0geWkuLi4zOSwgdmlfdnIgPSB2aS4uLjQwLCB5aV9jdnIgPSB5aS4uLjQxLCB2aV9jdnIgPSB2aS4uLjQyKQ0KDQpyZV9yZWYkcGx1PC1hcy5mYWN0b3IocmVfcmVmJHBsdSkNCnJlX3JlZiRwbHU8LXJlbGV2ZWwocmVfcmVmJHBsdSwgInNlbWktbmF0dXJhbCIpDQoNCiNuZWVkIGFub3RoZXIgcmFuZG9tIGZhY3RvciBmb3IgJ3VuaXQnDQoNCnVuaXQgPC0gZmFjdG9yKDE6bGVuZ3RoKHJlX3JlZiR5aV9tZWFuKSkNCnJlX3JlZiR1bml0IDwtIHVuaXQNCg0KcmVfcmVmPC1hcy5kYXRhLmZyYW1lKHJlX3JlZikgIyB0aGUgZ3JvdXBfYnkgdG8gZG8gdGhlIHNoYXJlZCBjb250cm9sIGNoZWNrIGFib3ZlIHR1cm5zIHRoaXMgYmFkIGJveSBpbnRvIGEgdGliYmxlLCBuZWVkcyB0byBiZSBhIGRhdGFmcmFtZSBmb3IgdGhlIGJlbG93IGZ1bmN0aW9uDQoNCnZjdl9jdnJfcnI8LW1ha2VfVkNWX21hdHJpeChkYXRhID0gcmVfcmVmLCBWID0idmlfY3ZyIiwgY2x1c3RlciA9ICJyZWZfc2hhcmVkX2N0cmwiLCBvYnMgPSAidW5pdCIsIHJobz0wLjUpDQp2Y3ZfbWVhbl9ycjwtbWFrZV9WQ1ZfbWF0cml4KHJlX3JlZiwgViA9InZpX21lYW4iLCAicmVmX3NoYXJlZF9jdHJsIiwgInVuaXQiLCByaG89MC41KQ0KdmN2X3ZyX3JyPC1tYWtlX1ZDVl9tYXRyaXgocmVfcmVmLCBWID0idmlfdnIiLCAicmVmX3NoYXJlZF9jdHJsIiwgInVuaXQiLCByaG89MC41KQ0KDQoNCmBgYA0KDQojIyBNZXRhLWFuYWx5dGljIG1vZGVscyByZS1ydW4gaW5jbHVkaW5nIHRheG9uIGFzIGEgbW9kZXJhdG9yDQoNCkFkZGl0aW9uYWxseSwgd2UgdGVzdCBmb3IgYW55IGRpZmZlcmVuY2VzIG9mIGJvdGggbWFpbiBlZmZlY3RzIGFuZCBhZ2UgZWZmZWN0cyBmb3IgZWFjaCBicm9hZCB0YXhvbiBjYXRlZ29yeSAoJ3BsYW50cycsICdpbnZlcnRlYnJhdGVzJywgJ3ZlcnRlYnJhdGVzJywgJ3NvaWwgbWljcm9iZXMnLCAnYW1vZWJhJywgYW5kICdmdW5naScpLiBGaXJzdCB3ZSBydW4gbWV0YS1hbmFseXRpYyBtb2RlbHMgb2YgTG5DVlIgYW5kIExuUlIgZm9yIGJvdGggcmVzdG9yZWQvdW5yZXN0b3JlZCBhbmQgcmVzdG9yZWQvcmVmZXJlbmNlIGNvbXBhcmlzb25zLiBJbiBUYWJsZSBTNCwgd2UgcHJpbnQgdGhlIFFtIHN0YXRpc3RpYyBmb3IgdGhlc2Ugb3IgdGhlIHNvLWNhbGxlZCAib21uaWJ1cyB0ZXN0IiB3aGljaCB0byBwYXJhcGhyYXNlIFdvbGZnYW5nIFZpZWNodGJhdWVyIChzZWUgaHR0cDovL3d3dy5tZXRhZm9yLXByb2plY3Qub3JnL2Rva3UucGhwL3RpcHM6dGVzdGluZ19mYWN0b3JzX2xpbmNvbXMgKSBmb3IgZXh0ZW5kZWQgZGlzY3Vzc2lvbikgaXMgdG8gdGVzdCBpZiBhdCBsZWFzdCBwYXJ0IG9mIHRoZSBoZXRlcm9nZW5laXR5IGluIHRoZSB0cnVlIGVmZmVjdHMgaXMgcmVsYXRlZCB0byBzb21lIG9mIHRoZSB2YXJpYWJsZXMgaW4gdGhlIG1vZGVsIChpbiB0aGlzIGNhc2UsIHRheG9uKS4gDQoNCmBgYHtyfQ0KDQpjdnJfdXIgPC0gcm1hLm12KHlpX2N2ciwgdmN2X2N2ciwgbW9kcz1+dGF4b24sIHJhbmRvbSA9IGxpc3QofjEgfCBpZCwgfjEgfCBwbG90X2lkLCB+MSB8IHVuaXQpLCBtZXRob2QgPSAiUkVNTCIsIGRhdGEgPSB1bl9yZSkgIyBjYW4ndCByZW1vdmUgaW50ZXJjZXB0LCBvciBlbHNlIHdlIGFyZSB0ZXN0aW5nIHdoZXRoZXIgdGhlIGF2ZXJhZ2UgdHJ1ZSBvdXRjb21lIGlzIGVxdWFsIHRvIDAgZm9yIGFsbCBsZXZlbHMsIG5vdCBpZiB0aGVyZSBhcmUgYmV0d2Vlbi1ncm91cCBkaWZmZXJlbmNlcy4gZ3JhbmRlIGRpZmZlcmVuemEhIQ0KDQptZWFuX3VyIDwtIHJtYS5tdih5aV9tZWFuLCB2Y3ZfbWVhbixtb2RzPX50YXhvbiwgcmFuZG9tID0gbGlzdCh+MSB8IGlkLCB+MSB8IHBsb3RfaWQsIH4xIHwgdW5pdCksIG1ldGhvZCA9ICJSRU1MIiwgZGF0YSA9IHVuX3JlKQ0KY3ZyX3JyIDwtIHJtYS5tdih5aV9jdnIsIHZjdl9jdnJfcnIsIG1vZHM9fnRheG9uLHJhbmRvbSA9IGxpc3QofjEgfCBpZCwgfjEgfCBwbG90X2lkLCB+MSB8IHVuaXQpLCBtZXRob2QgPSAiUkVNTCIsIGRhdGEgPSByZV9yZWYpDQptZWFuX3JyIDwtIHJtYS5tdih5aV9tZWFuLCB2Y3ZfbWVhbl9yciwgbW9kcz1+dGF4b24scmFuZG9tID0gbGlzdCh+MSB8IGlkLCB+MSB8IHBsb3RfaWQsIH4xIHwgdW5pdCksIG1ldGhvZCA9ICJSRU1MIiwgZGF0YSA9IHJlX3JlZikNCg0KYGBgDQoNCg0KKipUYWJsZSBTMTU6KiogUU0gc3RhdGlzdGljcywgZGVncmVlcyBvZiBmcmVlZG9tLCBhbmQgcC12YWx1ZXMgZm9yIHRoZSB0ZXN0IG9mIG1vZGVyYXRvcnMgZm9yIGVhY2ggbWV0YS1hbmFseXRpYyBtb2RlbC4NCg0KYGBge3J9DQpvcHRpb25zKHNjaXBlbiA9IDk5OSkNCg0KeDwtY2JpbmQoYygiTG9nIENWIC0gcmVzdG9yZWQvdW5yZXN0b3JlZCIsICJMb2cgcmVzcG9uc2UgcmF0aW8gLSByZXN0b3JlZC91bnJlc3RvcmVkIiwgIkxvZyBDViAtIHJlc3RvcmVkL3JlZmVyZW5jZSIsICJMb2cgcmVzcG9uc2UgcmF0aW8gLSByZXN0b3JlZC9yZWZlcmVuY2UiKSwgYyhjdnJfdXIkUU0sIG1lYW5fdXIkUU0sIGN2cl9yciRRTSwgbWVhbl9yciRRTSksDQpjKGN2cl91ciRRTWRmWzFdLCBtZWFuX3VyJFFNZGZbMV0sIGN2cl9yciRRTWRmWzFdLCBtZWFuX3JyJFFNZGZbMV0pLA0KYyhjdnJfdXIkUU1wLCBtZWFuX3VyJFFNcCwgY3ZyX3JyJFFNcCwgbWVhbl9yciRRTXApKQ0KDQp4PC1gY29sbmFtZXM8LWAoeCwgYygiTW9kZWwiLCAiUU0iLCAiUU0gZGYiLCAiUU0gcCB2YWx1ZSIpKQ0KDQp4PC1hcy5kYXRhLmZyYW1lKHgpDQoNCng8LXggJT4lIG11dGF0ZShRTSA9ICBhcy5udW1lcmljKFFNKSwNCiAgICAgICAgICAgICBgUU0gZGZgID0gIGFzLm51bWVyaWMoYFFNIGRmYCksDQogICAgICAgICAgICAgYFFNIHAgdmFsdWVgID0gIGFzLm51bWVyaWMoYFFNIHAgdmFsdWVgKSkNCg0KDQoNCg0KeCAlPiUga2FibGUoImh0bWwiLCBkaWdpdHMgPSAzKSAlPiUgDQogIGthYmxlX3N0eWxpbmcoInN0cmlwZWQiLCBwb3NpdGlvbiA9ICJsZWZ0IikNCg0KYGBgDQoNCldlIHNlZSBubyBldmlkZW5jZSBmb3IgdGhpcyBpbiBhbnkgb2YgdGhlIG1vZGVscywgdGhlcmVmb3JlIGRvIG5vdCBwcm9jZWVkIHRvIHBvc3Rob2MgdGVzdHMgdG8gYWRqdXN0IGZvciB0aGUgbXVsdGlwbGUgY29tcGFyaXNvbnMgYW5kIGdldCBwYWlyd2lzZSBkaWZmZXJlbmNlcyBiZXR3ZWVuIGdyb3Vwcy4NCg0KDQoNCmBgYHtyLCBmaWcud2lkdGg9OCwgZmlnLmhlaWdodD0xMH0NCg0KIyB0aGlzIHdhcyB0byBtYWtlIHBsb3RzLCB3aGljaCBhcmUgY3VycmVudCBub3QgcHJpbnRpbmcgYmVjYXVzZSB1bmVjZXNzYXJ5IA0KDQojIGN2cm9yZ3VyPC1vcmNoYXJkX3Bsb3QoY3ZyX3VyLCBtb2Q9InRheG9uIiwgeGxhYiA9ICJsb2cgQ1YgcmF0aW8gLSB1bnJlc3RvcmVkL3Jlc3RvcmVkIiwgYWxwaGEgPSAwLjEsIGs9VCkrdGhlbWVfY2xhc3NpYygpDQojIG1lYW5vcmd1cjwtb3JjaGFyZF9wbG90KG1lYW5fdXIsIG1vZD0idGF4b24iLCB4bGFiID0gImxvZyByZXNwb25zZSByYXRpbyAtIHVucmVzdG9yZWQvcmVzdG9yZWQiLCBhbHBoYSA9IDAuMSwgaz1UKSt0aGVtZV9jbGFzc2ljKCkNCiMgY3Zyb3JncnI8LW9yY2hhcmRfcGxvdChjdnJfcnIsIG1vZD0idGF4b24iLCB4bGFiID0gImxvZyBDViByYXRpbyAtIHJlZmVyZW5jZS9yZXN0b3JlZCIsIGFscGhhID0gMC4xLCBrPVQpK3RoZW1lX2NsYXNzaWMoKQ0KIyBtZWFub3JncnI8LW9yY2hhcmRfcGxvdChtZWFuX3JyLCBtb2Q9InRheG9uIiwgeGxhYiA9ICJsb2cgcmVzcG9uc2UgcmF0aW8gLSByZWZlcmVuY2UvcmVzdG9yZWQiLCBhbHBoYSA9IDAuMSwgaz1UKSt0aGVtZV9jbGFzc2ljKCkNCiMgDQojIA0KIyAoY3Zyb3JndXIvbWVhbm9yZ3VyKStwbG90X2Fubm90YXRpb24odGFnX2xldmVscyA9ICJhIiwgdGFnX3N1ZmZpeCA9ICIpIikNCg0KYGBgDQoNCg0KYGBge3IsIGZpZy5oZWlnaHQ9MTAsIGZpZy53aWR0aD04fQ0KIyBhcyBhYm92ZQ0KDQojIChjdnJvcmdyci9tZWFub3JncnIpK3Bsb3RfYW5ub3RhdGlvbih0YWdfbGV2ZWxzID0gImEiLCB0YWdfc3VmZml4ID0gIikiKQ0KDQpgYGANCg0KIyMgQWdlIGVmZmVjdHMgYnkgdGF4b24NCg0KTmV4dCwgd2UgZG8gdGhlIHNhbWUgdGhpbmcgdG8gdGVzdCBpZiB0aGVyZSBpcyBzaWduaWZpY2FudCB2YXJpYXRpb24gaW4gdGhlIGludGVyYWN0aW9uIGJldHdlZW4gYHRheG9uYCBhbmQgYGFnZWAuDQoNCmBgYHtyfQ0KY3ZyX3VyX2FnZSA8LSBybWEubXYoeWlfY3ZyLCB2Y3ZfY3ZyLCBtb2RzID0gfmFnZS5yZXN0Ljp0YXhvbiwgcmFuZG9tID0gbGlzdCh+MSB8IGlkLCB+MSB8IHBsb3RfaWQsIH4xIHwgdW5pdCksIG1ldGhvZCA9ICJSRU1MIiwgZGF0YSA9IHVuX3JlKQ0KbWVhbl91cl9hZ2UgPC0gcm1hLm12KHlpX21lYW4sIHZjdl9tZWFuLCBtb2RzID0gfmFnZS5yZXN0Ljp0YXhvbiwgcmFuZG9tID0gbGlzdCh+MSB8IGlkLCB+MSB8IHBsb3RfaWQsIH4xIHwgdW5pdCksIG1ldGhvZCA9ICJSRU1MIiwgZGF0YSA9IHVuX3JlKQ0KDQpjdnJfcnJfYWdlIDwtIHJtYS5tdih5aV9jdnIsIHZjdl9jdnJfcnIsIG1vZHMgPSB+YWdlLnJlc3QuOnRheG9uLCByYW5kb20gPSBsaXN0KH4xIHwgaWQsIH4xIHwgcGxvdF9pZCwgfjEgfCB1bml0KSwgbWV0aG9kID0gIlJFTUwiLCBkYXRhID0gcmVfcmVmKQ0KbWVhbl9ycl9hZ2UgPC0gcm1hLm12KHlpX21lYW4sIHZjdl9tZWFuX3JyLCBtb2RzID0gfmFnZS5yZXN0Ljp0YXhvbiwgcmFuZG9tID0gbGlzdCh+MSB8IGlkLCB+MSB8IHBsb3RfaWQsIH4xIHwgdW5pdCksIG1ldGhvZCA9ICJSRU1MIiwgZGF0YSA9IHJlX3JlZikNCg0KDQojIGN2cnVyYWdlPC1vcmNoYXJkX3Bsb3QoY3ZyX3VyX2FnZSwgbW9kPSJ0YXhvbiIsIHhsYWIgPSAibG9nKHZhcmlhYmlsaXR5IHJhdGlvKSAtIHVucmVzdG9yZWQvcmVzdG9yZWQiLCBhbHBoYSA9IDAuMSwgaz1UKSt0aGVtZV9jbGFzc2ljKCkNCiMgbWVhbnVyYWdlPC1vcmNoYXJkX3Bsb3QobWVhbl91cl9hZ2UsIG1vZD0idGF4b24iLCB4bGFiID0gImxvZyh2YXJpYWJpbGl0eSByYXRpbykgLSB1bnJlc3RvcmVkL3Jlc3RvcmVkIiwgYWxwaGEgPSAwLjEsIGs9VCkrdGhlbWVfY2xhc3NpYygpDQojIGN2cnJyYWdlPC1vcmNoYXJkX3Bsb3QoY3ZyX3JyX2FnZSwgbW9kPSJ0YXhvbiIsIHhsYWIgPSAibG9nKHZhcmlhYmlsaXR5IHJhdGlvKSAtIHVucmVzdG9yZWQvcmVzdG9yZWQiLCBhbHBoYSA9IDAuMSwgaz1UKSt0aGVtZV9jbGFzc2ljKCkNCiMgbWVhbnJyYWdlPC1vcmNoYXJkX3Bsb3QobWVhbl9ycl9hZ2UsIG1vZD0idGF4b24iLCB4bGFiID0gImxvZyh2YXJpYWJpbGl0eSByYXRpbykgLSB1bnJlc3RvcmVkL3Jlc3RvcmVkIiwgYWxwaGEgPSAwLjEsIGs9VCkrdGhlbWVfY2xhc3NpYygpDQpgYGANCg0KKipUYWJsZSBTMTY6KiogUU0gc3RhdGlzdGljcywgZGVncmVlcyBvZiBmcmVlZG9tLCBhbmQgcC12YWx1ZXMgZm9yIHRoZSB0ZXN0IG9mIG1vZGVyYXRvcnMgZm9yIGVhY2ggbWV0YS1hbmFseXRpYyBtb2RlbC4NCg0KYGBge3J9DQoNCng8LWNiaW5kKGMoIkxvZyBDViAtIHJlc3RvcmVkL3VucmVzdG9yZWQgKGFnZTp0YXhvbiBpbnRlcmFjdGlvbikiLCAiTG9nIHJlc3BvbnNlIHJhdGlvIC0gcmVzdG9yZWQvdW5yZXN0b3JlZCAoYWdlOnRheG9uIGludGVyYWN0aW9uKSIsICJMb2cgQ1YgLSByZXN0b3JlZC9yZWZlcmVuY2UgKGFnZTp0YXhvbiBpbnRlcmFjdGlvbikiLCAiTG9nIHJlc3BvbnNlIHJhdGlvIC0gcmVzdG9yZWQvcmVmZXJlbmNlIChhZ2U6dGF4b24gaW50ZXJhY3Rpb24pIiksIGMoY3ZyX3VyX2FnZSRRTSwgbWVhbl91cl9hZ2UkUU0sIGN2cl9ycl9hZ2UkUU0sIG1lYW5fcnJfYWdlJFFNKSwNCmMoY3ZyX3VyX2FnZSRRTWRmWzFdLCBtZWFuX3VyX2FnZSRRTWRmWzFdLCBjdnJfcnJfYWdlJFFNZGZbMV0sIG1lYW5fcnJfYWdlJFFNZGZbMV0pLA0KYyhjdnJfdXJfYWdlJFFNcCwgbWVhbl91cl9hZ2UkUU1wLCBjdnJfcnJfYWdlJFFNcCwgbWVhbl9ycl9hZ2UkUU1wKSkNCg0KeDwtYGNvbG5hbWVzPC1gKHgsIGMoIk1vZGVsIiwgIlFNIiwgIlFNIGRmIiwgIlFNIHAgdmFsdWUiKSkNCg0KeDwtYXMuZGF0YS5mcmFtZSh4KQ0KDQp4PC14ICU+JSBtdXRhdGUoUU0gPSAgYXMubnVtZXJpYyhRTSksDQogICAgICAgICAgICAgYFFNIGRmYCA9ICBhcy5udW1lcmljKGBRTSBkZmApLA0KICAgICAgICAgICAgIGBRTSBwIHZhbHVlYCA9ICBhcy5udW1lcmljKGBRTSBwIHZhbHVlYCkpDQoNCg0KDQoNCnggJT4lIGthYmxlKCJodG1sIiwgZGlnaXRzID0gMykgJT4lIA0KICBrYWJsZV9zdHlsaW5nKCJzdHJpcGVkIiwgcG9zaXRpb24gPSAibGVmdCIpDQoNCmBgYA0KSGVyZSwgd2UgY2FuIHNlZSB0aGF0IHRoZXJlIGlzIGV2aWRlbmNlIHRoYXQgc29tZSBvZiB0aGUgaGV0ZXJvZ2VuZWl0eSBpbiB0aGUgInRydWUgZWZmZWN0IiBpcyBkdWUgdG8gdGhlIGluY2x1ZGVkIG1vZGVyYXRvcnMNCg0KDQojIyBTdWJncm91cCBhbmFseXNlcyBiZXR3ZWVuIHRheG9uIGFuZCBpbnRlcmFjdGluZyB3aXRoIGFnZQ0KDQpMYXN0LCBzaW5jZSB3ZSBoYWQgYSBzaWduaWZpY2FudCBRTSBzdGF0aXN0aWMgaW4gdGhlIExuUlIgbW9kZWwgY29tcGFyaW5nIHJlc3RvcmVkL3VucmVzdG9yZWQgbW9kZWwsIHdlIGNvbmR1Y3QgYSBwb3N0aG9jIHRlc3QgYmVsb3cgdG8gdGVzdCBmb3IgZGlmZmVyZW5jZXMgYmV0d2VlbiBlYWNoIGdyb3VwIChlYWNoIGdyb3VwIGJlaW5nIHRoZSBpbnRlcmFjdGlvbiBiZXR3ZWVuIHRheG9uIGFuZCBhZ2Ugb2YgcmVzdG9yZWQgc2l0ZSkuIFdlIGhhdmUgdG8gcnVuIGEgbmV3IG1vZGVsIHdpdGhvdXQgdGhlIGludGVyY2VwdCwgYmVjYXVzZSBvdGhlcndpc2Ugd2UgYXJlIGluY2x1ZGluZyB0aGF0IGluIHRoZSBjb21iaW5hdGlvbnMgb2YgcGFpcnMgKHNlZSBkaXNjdXNzaW9uIGhlcmU6IGh0dHBzOi8vc3RhdHMuc3RhY2tleGNoYW5nZS5jb20vcXVlc3Rpb25zLzMyNDg4NS9lYXN5LXBvc3QtaG9jLXRlc3RzLXdoZW4tbWV0YS1hbmFseXppbmctd2l0aC10aGUtbWV0YWZvci1wYWNrYWdlLWluLXIgYW5kIFdvbGZnYW5nIFZpZWNodGJhdWVyJ3MgZGlzY3Vzc2lvbiBvZiB0aGlzIGhlcmUgaHR0cDovL3d3dy5tZXRhZm9yLXByb2plY3Qub3JnL2Rva3UucGhwL3RpcHM6dGVzdGluZ19mYWN0b3JzX2xpbmNvbXMpLg0KDQpJbiB0aGUgY29kZSwgYnV0IG5vdCBwcmludGVkIGluIHRoZSBvdXRwdXQsIHdlIHJ1biBhIHNlY29uZCBwb3N0aG9jIHRlc3QgYWRqdXN0ZWQgZm9yIG11bHRpcGxpY2l0eSBqdXN0IHRvIGJlIHRob3JvdWdoLCBhbHNvIGZpbmRpbmcgbm8gZGlmZmVyZW5jZXMuDQoNCg0KKipUYWJsZSBTMTcuKiogUGFpcndpc2UgY29tcGFyaXNvbiBiZXR3ZWVuIHN1Ymdyb3VwcyAodGF4b246YWdlIGludGVyYWN0aW9uKSBmb3IgbG9nIHJlc3BvbnNlIHJhdGlvIGJldHdlZW4gcmVzdG9yZWQgYW5kIHVucmVzdG9yZWQgc2l0ZXMuDQoNCg0KDQpgYGB7cn0NCg0KbWVhbl91cl9hZ2UgPC0gcm1hLm12KHlpX21lYW4sIHZjdl9tZWFuLCBtb2RzID0gfmFnZS5yZXN0Ljp0YXhvbi0xLCByYW5kb20gPSBsaXN0KH4xIHwgaWQsIH4xIHwgcGxvdF9pZCwgfjEgfCB1bml0KSwgbWV0aG9kID0gIlJFTUwiLCBkYXRhID0gdW5fcmUpDQoNCnJybWVhbl9hZ2U8LXN1bW1hcnkoZ2xodChtZWFuX3VyX2FnZSwgbGluZmN0PWNiaW5kKGNvbnRyTWF0KHJlcCgxLDYpLCB0eXBlPSJUdWtleSIpKSksIHRlc3Q9YWRqdXN0ZWQoIm5vbmUiKSkNCiNycm1lYW5fYWdlPC1zdW1tYXJ5KGdsaHQobWVhbl91cl9hZ2UsIGxpbmZjdD1jYmluZChjb250ck1hdChyZXAoMSw2KSwgdHlwZT0iVHVrZXkiKSkpLCB0ZXN0PWFkanVzdGVkKCJXZXN0ZmFsbCIpKSAjIGNoZWNrIHVzaW5nIGEgY29ycmVjdGlvbiBmb3IgbXVsdGlwbGljaXR5LCBubyBkaWZmZXJlbnQgc28gbGVhdmluZyBhbG9uZyB0byBrZWVwIGNvbnNpc3RlbnQgd2l0aCB0aGUgYWJvdmUgbW9kZWxzDQoNCiMgcGx1cywgb24gcmVhZGluZyBXZXN0ZmFsbC9TaGFmZmVyLCBpdCBzZWVtcyB0aGF0IGNvcnJlY3Rpb25zIHJlZHVjZXMgdGhlIFR5cGUgSSBlcnJvciByYXRlLCBhbmQgdGhlbiBub3QgY29ycmVjdGluZyByZWR1Y2VzIHRoZSBUeXBlIElJIGVycm9yIC0gd2hpY2ggc2luY2UgdGhyZSBpcyBuLnMuIHJlc3VsdHMgZWl0aGVyIHdheSB0aGlzIGlzIG5vdCB0aGF0IGltcG9ydGFudCBoZXJlPyANCg0KDQpycm1lYW5hZ2U8LWFzLmRhdGEuZnJhbWUoY2JpbmQocnJtZWFuX2FnZSR0ZXN0JGNvZWZmaWNpZW50cywgcnJtZWFuX2FnZSR0ZXN0JHNpZ21hLCBycm1lYW5fYWdlJHRlc3QkdHN0YXQsIHJybWVhbl9hZ2UkdGVzdCRwdmFsdWVzKSkgJT4lDQogIHJlbmFtZShjb2VmZmljaWVudCA9IFYxLA0KICAgICAgICAgc2lnbWEgPSBWMiwNCiAgICAgICAgIHRzdGF0ID0gVjMsDQogICAgICAgICBwID0gVjQpDQoNCmE8LWJpbmRfY29scyhjKDE6NiksIGMoImFtb2ViYWUiLCAiZnVuZ2kiLCAiaW52ZXJ0ZWJyYXRlcyIsICJwbGFudHMiLCAic29pbCBtaWNyb2JlcyIsICJ2ZXJ0ZWJyYXRlcyIpKSAlPiUgcmVuYW1lKG51bSA9IGAuLi4xYCwgdGF4ID0gYC4uLjJgKQ0KDQpybWVhbmFnZTwtYXMuZGF0YS5mcmFtZShyb3duYW1lcyhycm1lYW5hZ2UpKSAlPiUgcmVuYW1lKGNvbXAgPSBgcm93bmFtZXMocnJtZWFuYWdlKWApICU+JSBtdXRhdGUocmVmID0gYXMubnVtZXJpYyh3b3JkKGNvbXAsIDIsIHNlcCA9ICItIikpLCBjb21wYXJpc29uID0gYXMubnVtZXJpYyh3b3JkKGNvbXAsIDEsIHNlcCA9ICItIikpKSAlPiUgbGVmdF9qb2luKC4sIGEsIGJ5ID0gYygicmVmIiA9ICJudW0iKSkgJT4lIHJlbmFtZShyZWZlcmVuY2VfdGF4YSA9IHRheCkgJT4lDQogIGxlZnRfam9pbiguLCBhLCBieSA9IGMoImNvbXBhcmlzb24iID0gIm51bSIpKSAlPiUgcmVuYW1lKGNvbXBhcmlzb25fdGF4YSA9IHRheCkgJT4lIGJpbmRfY29scyguLCBycm1lYW5hZ2UpDQoNCnJvd25hbWVzKHJtZWFuYWdlKSA8LSBOVUxMDQoNCnJtZWFuYWdlICU+JSBkcGx5cjo6c2VsZWN0KC1jKDE6MykpICU+JSBrYWJsZShkaWdpdHMgPSAzKSAlPiUga2FibGVfc3R5bGluZygpJT4lDQogICAgc2Nyb2xsX2JveCh3aWR0aCA9ICI4MDBweCIsIGhlaWdodCA9ICIzNTBweCIpDQpgYGANCg0KDQoNCiMjIFIgU2Vzc2lvbiBJbmZvcm1hdGlvbg0KDQpgYGB7cn0NCmxpYnJhcnkocGFuZGVyKQ0Kc2Vzc2lvbkluZm8oKSAlPiUgcGFuZGVyKCkNCmBgYA0K
